# Genomic binding of PAX8-PPARG fusion protein regulates cancer-related pathways and alters the immune landscape of thyroid cancer

**DOI:** 10.18632/oncotarget.14050

**Published:** 2016-12-20

**Authors:** Yanxiao Zhang, Jingcheng Yu, Vladimir Grachtchouk, Tingting Qin, Carey N Lumeng, Maureen A Sartor, Ronald J Koenig

**Affiliations:** ^1^ Department of Computational Medicine and Bioinformatics, University of Michigan, Ann Arbor, MI, 48109, USA; ^2^ Division of Metabolism, Endocrinology and Diabetes, Department of Internal Medicine, University of Michigan, Ann Arbor, MI 48109, USA; ^3^ Department of Pediatrics and Communicable Diseases, University of Michigan, Ann Arbor, MI 48109, USA; ^4^ Current address: Ludwig Institute for Cancer Research, La Jolla, CA 92093-0653, USA

**Keywords:** peroxisome proliferator-activated receptor gamma, follicular thyroid cancer, pioglitazone, differentiation, gene fusion

## Abstract

PAX8-PPARG fusion protein (PPFP) results from a t(2;3)(q13;p25) chromosomal translocation, is found in 30% of follicular thyroid carcinomas, and demonstrates oncogenic capacity in transgenic mice. A PPARG ligand, pioglitazone, is highly therapeutic in mice with PPFP thyroid cancer. However, only limited data exist to characterize the binding sites and oncogenic function of PPFP, or to explain the observed therapeutic effect of pioglitazone. Here we used our previously characterized transgenic mouse model of PPFP follicular thyroid carcinoma to identify PPFP binding sites *in vivo* using ChIP-seq, and to distinguish genes and pathways regulated directly or indirectly by PPFP with and without pioglitazone treatment via integration with RNA-seq data. PPFP bound to DNA regions containing the PAX8 and/or the PPARG motif, near genes involved in lipid metabolism, the cell cycle, apoptosis, and cell motility; the binding site distribution was highly concordant with our previous study in a rat PCCL3 cell line. Most strikingly, pioglitazone induced an immune cell infiltration including macrophages and T cells only in the presence of PPFP, which may be central to its therapeutic effect.

## INTRODUCTION

A chromosomal translocation fusing the genes *PAX8* and *PPARG* is found in 30% of follicular thyroid carcinomas [1]. The resulting PAX8-PPARG fusion protein (PPFP) is oncogenic, but the underlying mechanisms are not well understood. PAX8 is a member of the paired box family of transcription factors. It is required for normal thyroid development and in the mature thyroid, it induces the expression of thyroid-specific genes [2, 3]. PPARG is a member of the nuclear receptor family of transcription factors. It is expressed at very low levels in the normal thyroid and has no known function in that organ. PPARG is the master regulator of adipogenesis [4], among other functions. Synthetic agonist ligands for PPARG such as pioglitazone are insulin sensitizing and hence are used to treat type 2 diabetes mellitus. PPFP contains all but the very carboxyl terminal section of PAX8 fused to the entirety of PPARG1. Therefore, PPFP contains both the PAX8 and the PPARG DNA binding domains (DBDs), and PPARG ligands also function as PPFP ligands.

We previously created a transgenic mouse model of PPFP follicular thyroid carcinoma that includes both thyroid-specific expression of PPFP and thyroid-specific deletion of *Pten* (PPFP^Thy^;Pten^Thy−/−^ mice) [5]. Mice with just thyroid-specific expression of PPFP (PPFP^Thy^) only develop mild thyroid hyperplasia [5]. However, human follicular carcinomas in general, including PPFP carcinomas, also have increased activation of AKT (increased phosphoAKT) [6, 7]. Since PTEN is a negative regulator of AKT, we created mice with the combined PPFP^Thy^;Pten^Thy−/−^ genotype to better mimic the human condition. These mice develop thyroid carcinomas with local invasion and lung metastases [5]. Mice with just *Pten* deletion (Pten^Thy−/−^) develop thyroid hyperplasia but no carcinomas [5].

Pioglitazone has a remarkable therapeutic effect in PPFP^Thy^;Pten^Thy−/−^ mice, greatly shrinking thyroid size and completely preventing lung metastases [5]. Furthermore, the remaining thyroid cells take on an adipocyte-like phenotype, accumulating large amounts of intracellular lipid and expressing many PPARG-inducible adipocyte genes. These cells continue to express thyroid genes indicating that they are still thyrocytes, but the results demonstrate that pioglitazone turns PPFP into a strongly PPARG-like transcription factor. In contrast, pioglitazone has no effect on the growth or histology of the thyroid glands in Pten^Thy−/−^ mice.

The mechanisms of action of PPFP as an oncogene and pioglitazone as a therapeutic agent are poorly understood. Here, we report the results of chromatin immunoprecipitation – deep sequencing (ChIP-seq) to identify PPFP binding sites in the mouse thyroid gland genome. In addition, we have used RNA deep sequencing (RNA-seq) to identify mouse thyroid genes regulated by PPFP in the absence or presence of pioglitazone. The results provide insights into the *in vivo* actions of PPFP and the therapeutic effect of pioglitazone in thyroid carcinoma.

## RESULTS

### Overview of the PPFP cistrome

**Figure 1:**
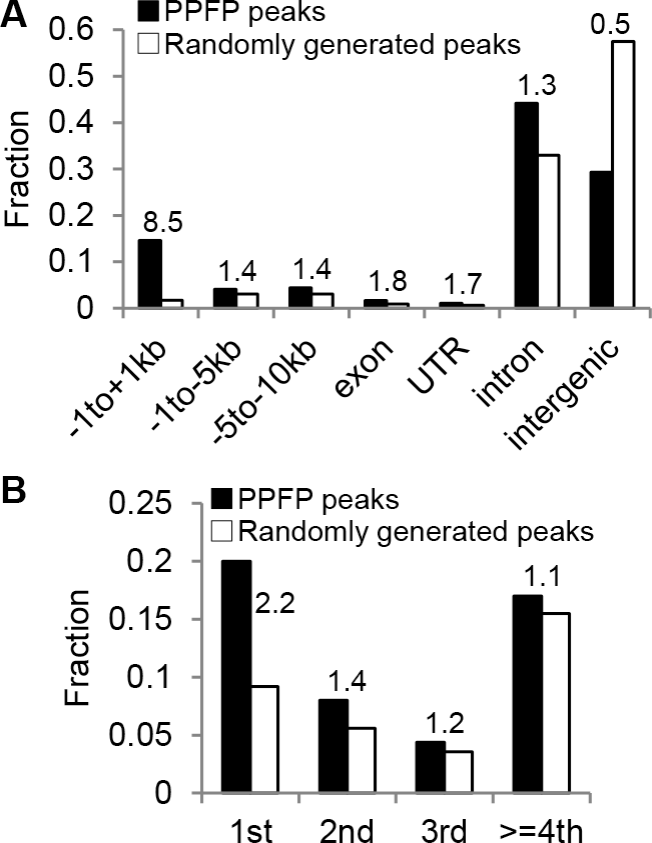
Annotation of PPFP peaks relative to genic and intergenic regions (**A**) Peaks with more than one annotation were assigned to one region only with prioritization going from left to right. (**B**) The intron group of A is divided into individual introns. The numbers above the bars indicate the ratios of PPFP to randomly generated peaks.

A PPFP ChIP-seq analysis was performed on the thyroid glands of PPFP^Thy^;Pten^Thy−/−^ mice to better understand the DNA binding properties of this transcription factor *in vivo* and the genes and processes it is likely to regulate through direct DNA interactions. We identified 20,094 PPFP peaks in the mouse genome. Compared with randomly generated peaks, there was an 8.5-fold enrichment of PPFP peaks within 1 kb of transcription start sites (TSS’s) (Figure 1). PPFP peaks also were enriched 1.4-fold from –1 to –5 kb of TSS’s, and 2.2-fold in first introns. In contrast, PPFP peaks were underrepresented 0.5-fold in intergenic regions.

**Figure 2:**
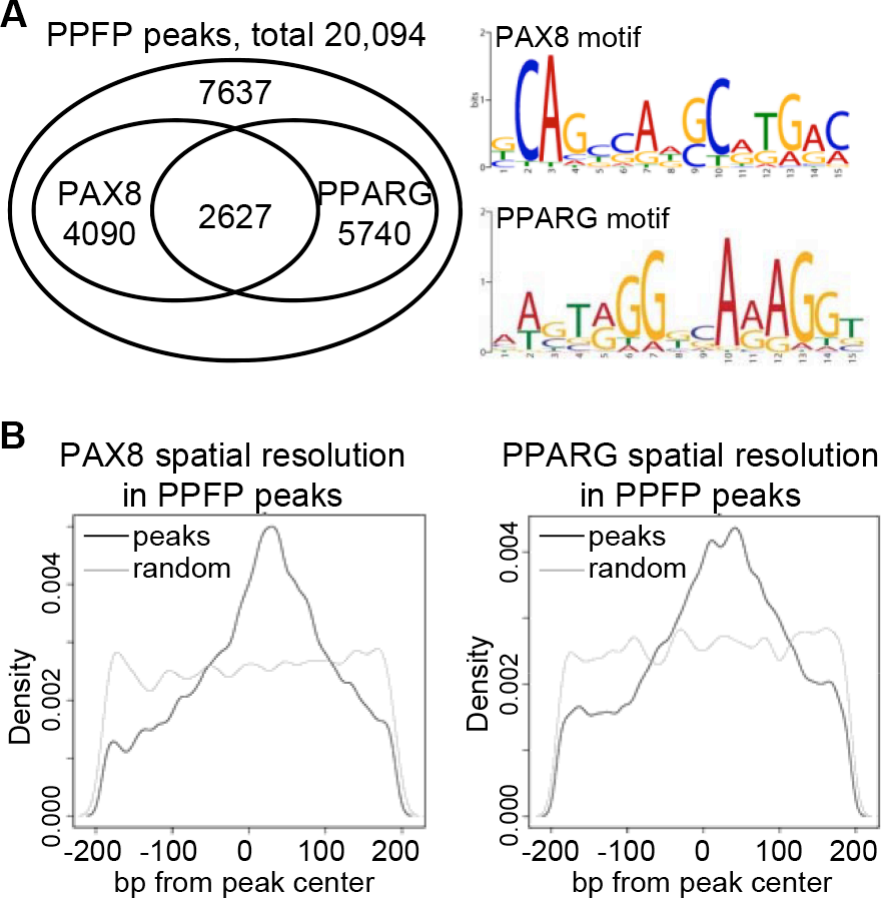
PPFP peaks contain PAX8 and/or PPARG motifs (**A**) Venn diagram showing the overlap of PAX8 and PPARG motifs within PPFP peaks, and the logos for PAX8 and PPARG motifs. (**B**) Spatial resolution analysis of PAX8 and PPARG motifs within PPFP peaks (black lines). The grey lines show the distribution of each motif in randomly generated 400 bp regions across the genome, as negative controls.

PPFP contains the DNA binding domains (DBDs) of both PAX8 and PPARG, and we found that both are functional *in vivo*, with 62% of peaks containing a PAX8 motif and/or a PPARG motif (Figure 2A). The fraction of peaks with either motif or both motifs was essentially identical for peaks < 10 kb versus > 10 kb from a TSS (data not shown). An analysis of motif locations relative to the peak modes indicates that both motifs are centered within the PPFP peaks, as would be expected for functionally relevant motifs (Figure 2B).

### PPFP binds to genes involved in fatty acid metabolism and cancer-related processes

Five hundred twenty two Gene Ontology (GO) terms were identified as enriched with PPFP peaks (FDR < 0.05), after associating peaks with the gene having the nearest TSS (Supplementary Table S1). Numerous GO terms contain the words “fatty acid” or “lipid,” consistent with the PPARG-like activity of PPFP. Examples include the GO terms *fatty acid oxidation*, *lipid metabolic process*, *lipid biosynthetic process*, and *lipid localization*. Additional terms were associated with cancer-related processes such as development, the cell cycle, apoptosis, cell motility, and others (Table 1).

**Table 1:** Examples of Gene Ontology terms related to cancer-associated processes that are associated with PPFP peaks

Word or phrase	Examples of GO terms associated with PPFP peaks	*q*-value
Actin/microtubule	GO:0030029 actin filament-based process	9.56E-06
	GO:0030036 actin cytoskeleton organization	4.83E-05
	GO:0008154 actin polymerization or depolymerization	0.0215
	GO:0030048 actin filament-based movement	0.0380
	GO:0032886 regulation of microtubule-based process	0.0081
	GO:0070507 regulation of microtubule cytoskeleton organization	0.0082
	GO:0031110 regulation of microtubule polymerization or depolymerization	.00155
Adhesion/junction	GO:0016337 cell-cell adhesion	0.0034
	GO:0034330 cell junction organization	0.0010
	GO:0034329 cell junction assembly	0.0115
	GO:0007160 cell-matrix adhesion	0.0160
	GO:0045216 cell-cell junction organization	0.0184
	GO:0031589 cell-substrate adhesion	0.0241
Apoptotic/cell death	GO:0008219 cell death	2.02E-11
	GO:0006915 apoptotic process	5.47E-11
	GO:0010941 regulation of cell death	3.00E-06
Cell cycle/proliferation	GO:0007049 cell cycle	6.32E-06
	GO:0042127 regulation of cell proliferation	2.02E-05
Development	GO:0001701 in utero embryonic development	6.49E-05
	GO:0060284 regulation of cell development	0.0004
	GO:0001944 vasculature development	0.0070
Folding/ubiquitin	GO:0006457 protein folding	0.0015
	GO:0016567 protein ubiquitination	0.0017
	GO:0043161 proteasomal ubiquitin-dependent protein catabolic process	0.0068
	GO:0061077 chaperone-mediated protein folding	0.0125
Glutathione/oxidative stress/reactive oxygen species	GO:0006749 glutathione metabolic process	0.0082
	GO:0034614 cellular response to reactive oxygen species	0.0465
	GO:0034599 cellular response to oxidative stress	0.0324
Glycolysis	GO:0006096 glycolysis	0.0018
GTP/RAS	GO:0007264 small GTPase mediated signal transduction	1.88E-07
	GO:0006184 GTP catabolic process	1.30E-06
	GO:0046039 GTP metabolic process	1.98E-06
	GO:0043547 positive regulation of GTPase activity	0.0001
	GO:0046578 regulation of Ras protein signal transduction	4.19E-05
	GO:0032318 regulation of Ras GTPase activity	0.0017
Migration/motility/locomotion	GO:0030334 regulation of cell migration	0.0017
	GO:2000147 positive regulation of cell motility	0.0111
	GO:0040017 positive regulation of locomotion	0.0351
Wnt	GO:0016055 Wnt receptor signaling pathway	0.0037
	GO:0030111 regulation of Wnt receptor signaling pathway	0.0467

We also performed separate GO term enrichment analyses for genes with PPFP peaks that contain only a PPARG motif, or that contain only a PAX8 motif. Seventy-one GO terms were enriched in the former and 46 in the latter (Supplementary Table S2). All 7 of the GO terms containing the word “lipid” or “fatty acid” are enriched with peaks that contain only the PPARG motif (Table 2), which suggests the PPARG DBD within PPFP is responsible for regulating the associated genes. Genes that play key direct roles in lipid metabolism such as *Cpt1a* and the acyl-coenzyme A dehydrogenases *Acadl*, *Acadm* and *Acads*, as well as genes that are important to the function of PPARs and peroxisome biogenesis such as *Ppargc1a* and *Pex2*, contain a peak with a PPARG motif within 5 kb of the TSS. Similarly, all of the GO terms containing the words “cell cycle” or “cell division,” “locomotion” or “motility,” or “Wnt” also are enriched with peaks that contain only the PPARG motif; examples include the cell cycle gene *Ccnd1* and the locomotion/motility-related genes *Pld1*, *Prkca* and *Vegfa*. In contrast, GO terms containing the word “hypoxia” or “oxygen,” or “junction” (e.g. *cell-substrate junction assembly*) are enriched with peaks that contain only the PAX8 motif, and 8 of the 9 GO terms containing the word “actin” or “cytoskeleton” also are in the PAX8-only motif group. This suggests that the PAX8 DBD within PPFP is responsible for regulating the majority of genes associated with the response to hypoxia, cell junctions, and the cytoskeleton.

**Table 2:** Selected Gene Ontology terms that are enriched in genes with PPFP peaks containing only a PPARG motif, or containing only a PAX8 motif

Word in GO term	GO terms enriched in genes with PPFP peaks that only have a PPARG motif	GO terms enriched in genes with PPFP peaks that only have a PAX8 motif
Cell cycle, cell division	GO:0051301 cell division	none
	GO:0090068 positive regulation of cell cycle process	
Fatty acid, lipid	GO:0044242 cellular lipid catabolic process	none
	GO:0000038 very long-chain fatty acid metabolic process	
	GO:0016042 lipid catabolic process	
	GO:0010883 regulation of lipid storage	
	GO:0043550 regulation of lipid kinase activity	
	GO:0032369 negative regulation of lipid transport	
	GO:0019217 regulation of fatty acid metabolic process	
Locomotion, motility	GO:0040012 regulation of locomotion	none
	GO:0040017 positive regulation of locomotion	
	GO:0048870 cell motility	
Wnt	GO:0016055 Wnt receptor signaling pathway	none
	GO:0030111 regulation of Wnt receptor signaling pathway	
	GO:0060070 canonical Wnt receptor signaling pathway	
Actin, cytoskeleton	GO:0030048 actin filament-based movement	GO:0030833 regulation of actin filament polymerization
		GO:0030838 positive regulation of actin filament polymerization
		GO:0030838 actin filament polymerization
		GO:0030832 regulation of actin filament length
		GO:0008154 actin polymerization or depolymerization
		GO:0008064 regulation of actin polymerization or depolymerization
		GO:0051495 regulation of cytoskeleton organization
		GO:0051495 positive regulation of cytoskeleton organization
Hypoxia, oxygen	none	GO:0001666 response to hypoxia
		GO:0070482 response to oxygen levels
		GO:0036293 response to decreased oxygen levels
Junction	none	GO:0007044 cell-substrate junction assembly
		GO:0034329 cell junction assembly

**Figure 3:**
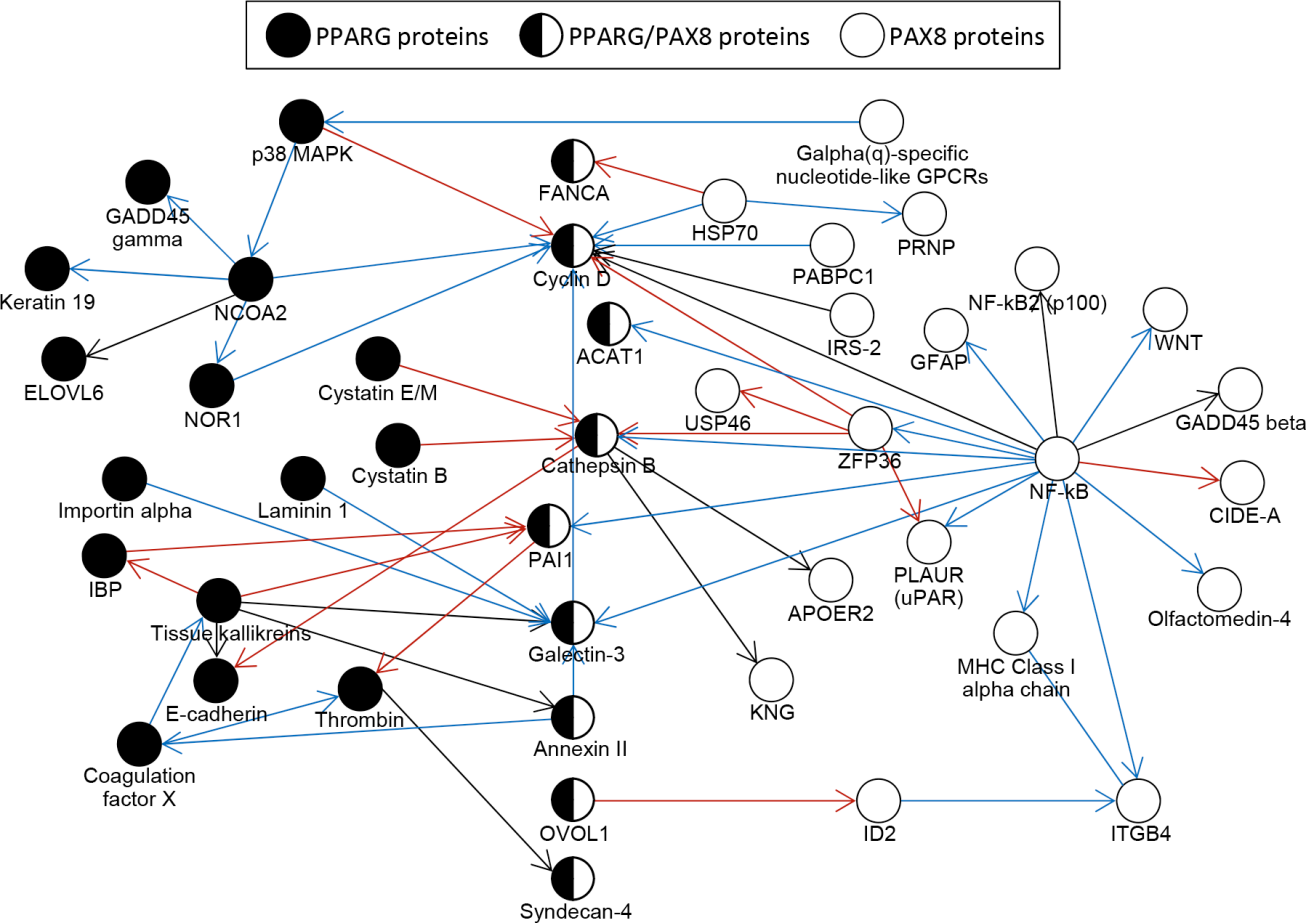
Protein interaction network analysis of genes that contain PPFP peak(s) only with PPARG motifs or only with PAX8 motifs, and that are differentially expressed in PPFPThy;PtenThy−/− mice versus PtenThy−/− mice on a control diet The largest networks are shown. Only genes with PPFP peaks within 10 kb of a TSS were analyzed, since otherwise the networks became too large. Red arrows indicate negative interactions; blue arrows, positive interactions; and black arrows, unspecified interactions. The direction of each arrow indicates the direction of the signaling cascade.

We performed an analysis of protein interaction networks for individual genes targeted by PPFP with only PPARG motifs, or with only PAX8 motifs (see *Materials and Methods* for details). The largest, directly-connected subnetworks overlap and are presented in Figure 3. The network of genes with PPARG motifs has a major node at the transcriptional coactivator NCOA2 and includes proteins involved in the cell cycle (Cyclin D), extracellular matrix (E-cadherin), response to cell stress (GADD45G), cell adhesion (Galectin-3), fatty acid metabolism (ELOVL6), and other processes. The largest subnetwork of genes with PAX8 motifs has a major node at NF-κB and overlaps with the PPARG network at multiple proteins including the previously-mentioned Cyclin D and Galectin-3. Similar to the PPARG network, the PAX8 network includes proteins involved in the extracellular matrix (integrin beta 4), response to cell stress (GADD45 beta), and other processes.

We also were interested to determine whether PPFP may potentially have different functions when binding in a proximal promoter region, which we define as within 10 kb of a TSS, versus at a greater distance from a TSS. We therefore separately identified GO terms enriched in genes that contain PPFP peaks < 10 kb from a TSS, or 10–100 kb from a TSS (Supplementary Table S3). Here we highlight several GO terms that had a strong association with peaks either < 10 kb or 10–100 kb from a TSS (not both). All nine significant GO terms containing the words “cell cycle” or “mitotic” and four GO terms containing the phrase “protein folding” have genes with PPFP peaks only < 10 kb of a TSS. Similarly, 14 GO terms contain the word “cytoskeleton” or “microtubule,” all of which contain a PPFP peak < 10 kb from the TSS and only 3 of which also contain a peak 10–100 kb. In contrast, all 9 GO terms that contain the word “locomotion,” “migration” or “motility” and all 5 GO terms with the word “Wnt”, only contain genes with PPFP peaks 10–100 kb from a TSS. Of the 63 “morphogenesis” or “development” related GO terms, 57 have genes with a peak 10–100 kb from a TSS and only 6 lack such a peak but contain genes with a peak < 10 kb from a TSS. These results suggest that PPFP regulates the cell cycle, protein folding and cytoskeleton changes from the promoter region, while regulating migration and motility, Wnt signaling and developmental processes from enhancer regions.

### Genes differentially expressed in human PPFP thyroid carcinomas are regulated by PPFP in PPFP^Thy^;Pten^Thy−/−^ mice

An RNA-seq differential expression analysis was performed on thyroid RNA from PPFP^Thy^;Pten^Thy−/−^ mice versus Pten^Thy−/−^ control mice. PPFP regulated the expression of 2923 genes (1297 up, 1626 down) using FDR < 0.05 and absolute fold change >2 as cut-offs (Supplementary Table S4). A previous publication identified 275 genes that were differentially expressed in human PPFP thyroid carcinomas versus non-PPFP thyroid carcinomas and normal thyroids [8]. We found that this set of genes also is regulated by PPFP in PPFP^Thy^;Pten^Thy−/−^ mice versus Pten^Thy−/−^ control mice (Fisher's exact test *p* = 0.0019, Table 3), thus supporting the relevance of this mouse model to the human disease. Examples of the overlap in these sets include PPARG target genes involved in fatty acid metabolism such as *Acaa1* and *Acads*, and genes involved in angiogenesis such as *Angptl4* and *Edil3*. A complete list of individual genes is found in Supplementary Table S5.

**Table 3:** Genes differentially expressed in human PPFP thyroid carcinomas have the same trend in differential expression in PPFP^Thy^;Pten^Thy−/−^ mice versus Pten^Thy-/^^-^ control mice

	human PPFP carcinomas repressed	human PPFP carcinomas induced	total
PPFP^Thy^;Pten^Thy−/−^ mice repressed	45	79	124
PPFP^Thy^;Pten^Thy−/−^ mice induced	19	90	109
total	64	169	233

Fisher's exact test, two-tailed *p* = 0.0019.

Of 275 genes differentially expressed in human PPFP thyroid carcinomas versus non-PPFP thyroid carcinomas and normal thyroids [8], 267 have mouse homologs and 34 have negligible expression in mouse thyroid, leaving 233 genes for analysis. We define mouse genes to be induced if log fold change > 0, and repressed if log fold change < 0.

### PPFP and pioglitazone tend to regulate genes in opposite directions

We treated PPFP^Thy^;Pten^Thy−/−^ mice for two weeks with the PPFP/PPARG ligand pioglitazone, and compared thyroid gene expression to that of PPFP^Thy^;Pten^Thy−/−^ mice on a control diet. Pioglitazone regulated the expression of 3047 genes (2012 up, 1035 down) (Supplementary Table S6). Fifty percent of all genes regulated by PPFP in the absence of pioglitazone also were regulated by pioglitazone. Similarly, 48% of all genes regulated by pioglitazone also were regulated by PPFP in the absence of this drug. However, when PPFP and pioglitazone regulated the same gene, they tended to do so in opposite directions (*p* < 0.0001, Fisher's exact test). Thus, 193 genes were induced by both and 116 were repressed by both, whereas 377 genes were induced by PPFP and repressed by pioglitazone, and 771 were repressed by PPFP and induced by pioglitazone.

### PPFP and pioglitazone regulate gene sets related to fatty acid metabolism, immune function and cancer-related processes

Functional enrichment testing of the RNA-seq data using GO terms and KEGG pathways identified 51 induced gene sets and 98 repressed gene sets in PPFP^Thy^;Pten^Thy−/−^ thyroids versus Pten^Thy−/−^ thyroids from mice on a control diet (FDR < 0.05) (Supplementary Table S7). Also shown in Table S7, 35% of the induced gene sets and 21% of the repressed gene sets were enriched in the ChIP-seq (*p* = 0.079 for this difference, two tailed Fisher's exact test). Consistent with the PPARG-like activity of PPFP, the induced gene sets include terms related to lipids and fatty acid metabolism, such as *lipid homeostasis* and *acyl-CoA metabolic process*. Multiple induced gene sets relate to ribosomes, such as *ribosome biogenesis, rRNA processing*, and *rRNA metabolic process*. Several repressed gene sets relate either to immune function, for example *immune response*, *cytokine-cytokine receptor interaction*, and *toll-like receptor signaling pathway*, or to cell junctions, such as *cell junction assembly, cell junction organization*, and *extracellular matrix disassembly*.

Pioglitazone regulated (either directly or via secondary mechanisms) 963 gene sets in PPFP^Thy^;Pten^Thy−/−^ thyroids (Supplementary Table S8). Surprisingly, 92% (889) of these gene sets were induced. Also shown in Supplementary Table S8, 21% of the induced gene sets and 7% of the repressed gene sets were enriched in the ChIP-seq (*p* = 0.0022 for this difference, two tailed Fisher's exact test). Pioglitazone is highly therapeutic in PPFP^Thy^;Pten^Thy−/−^ mice, greatly shrinking thyroid size and preventing metastatic disease [5]. Consistent with this, pioglitazone induced a number of cell death-related processes, such as, *positive regulation of cell death, positive regulation of apoptotic process*, and *intrinsic apoptotic signaling pathway in response to DNA damage*.

Pioglitazone turns PPFP into a strongly PPARG-like transcription factor and results in the trans-differentiation of the thyroid cancer cells into adipocyte-like cells [5]. Consistent with this, pioglitazone induced genes enriched in processes related to lipid and fatty acid metabolism, such as *PPAR signaling pathway, regulation of lipid storage, neutral lipid catabolic process, positive regulation of fat cell differentiation*, and *fatty acid metabolic process*. Also consistent with the thyroid cells becoming more adipocyte-like, pioglitazone repressed *thyroid hormone generation* and *thyroid hormone metabolic process*.

Many of the gene sets induced by pioglitazone are related to immune function. For example, 18 of the top ranked 20 gene sets (by FDR) contain either the word “immune,” “defense,” “leukocyte,” “inflammatory,” “phagocytosis,” or “cytokine.” It is important to note that 17 of these top 18 immune-related gene sets were not enriched with PPFP peaks in the ChIP-seq analysis, suggesting that their induction by pioglitazone is indirect (see below). The one that was also enriched with PPFP binding sites, *immune system process*, includes cytokine genes such as *Csf1*, *Il33*, *Il34* and *Irf1* that contain peaks and were induced. These genes, directly regulated by PPFP, may help explain the mechanism used to stimulate the strong immune response in these tumors.

The above studies did not include pioglitazone-treated Pten^Thy−/−^ mice to compare to control diet Pten^Thy−/−^ mice, which would formally demonstrate that the pioglitazone-associated changes in PPFP^Thy^;Pten^Thy−/−^ mice are dependent on PPFP. However, we previously performed an Affymetrix microarray analysis of PPFP^Thy^;Pten^Thy−/−^ mice and Pten^Thy−/−^ mice, both treated ± pioglitazone (an independent set of mice from those analyzed by RNA-seq). Analysis of the microarray data, previously unpublished, shows results consistent with the RNA-seq data. A similar, strong response to pioglitazone was observed in the PPFP^Thy^;Pten^Thy−/−^ mice (Supplementary Figures S1, S2). However, almost no response was detected by pioglitazone in the Pten^Thy−/−^ thyroids (only 8 out of 45,101 probe sets were differentially regulated). Pioglitazone induced a strong immune-related response in the PPFP^Thy^;Pten^Thy−/−^ thyroids (Supplementary Table S9), results that are very similar to the RNA-seq data. Thus, we conclude that the induction of gene sets by pioglitazone, including the large number of immune-related gene sets, is dependent on the expression of PPFP.

### Pioglitazone induces an immune cell infiltration in PPFP^Thy^;Pten^Thy−/−^ mouse thyroids

**Figure 4:**
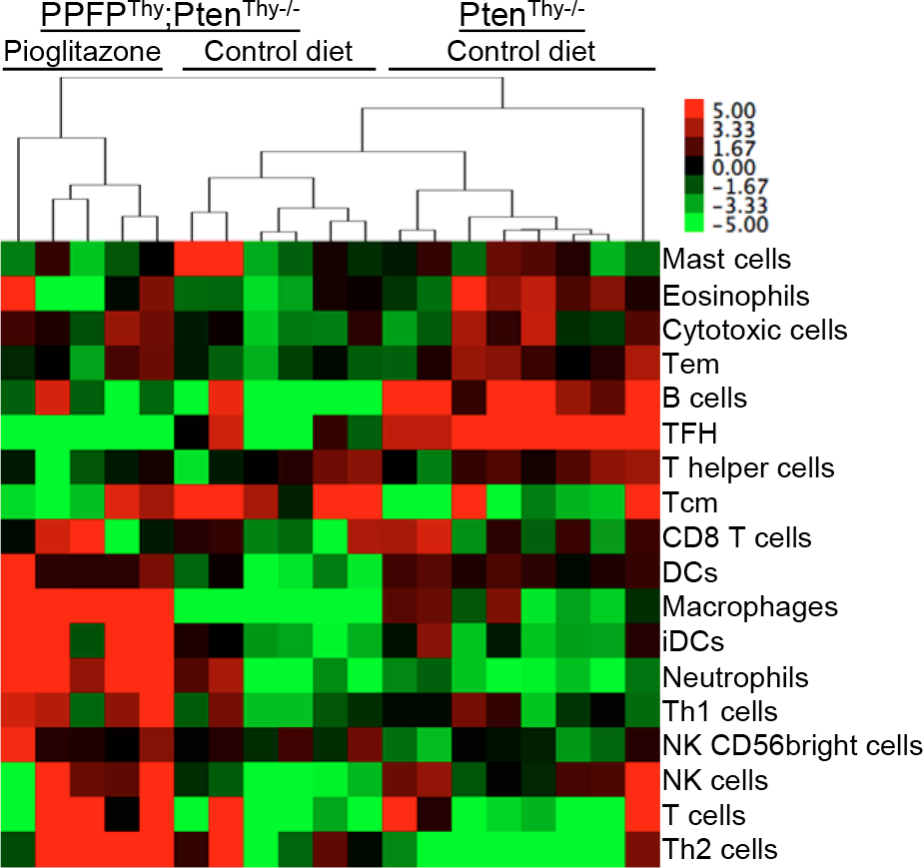
Heat map of overall gene expression scores for types of immune cells in thyroid glands of PPFPThy;PtenThy−/− mice fed pioglitazone or control diet, or PtenThy−/− mice fed control diet A heat map of expression of the individual genes used to determine the overall scores is found in Figure S3. Gene expression was measured by RNA-seq. The samples and cell types were clustered by hierarchical clustering using average linkage and the correlation distance measure. Abbreviations: Tem, T effector memory; TFH, T follicular helper; Tcm, T central memory; DCs, dendritic cells; iDCs, immature DCs; Th1, T helper 1; NK, natural killer; Th2, T helper 2.

**Figure 5:**
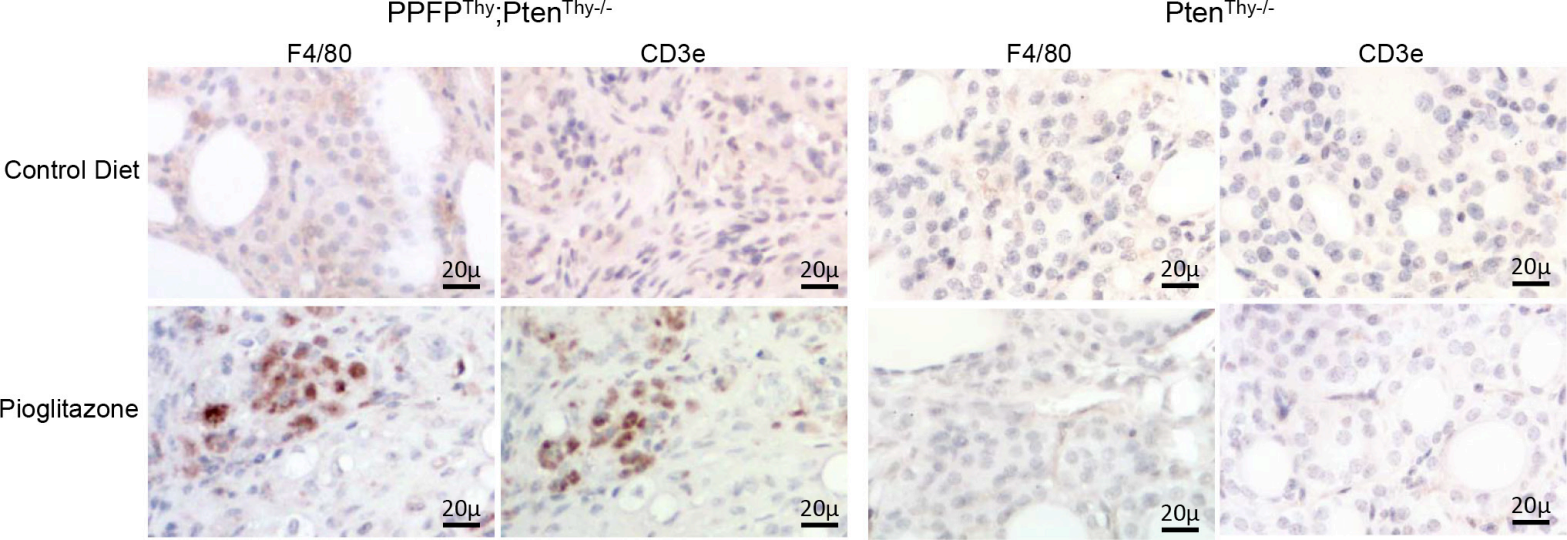
Immunohistochemical staining for F4/80 and CD3e in thyroid glands from PPFPThy;PtenThy−/− and PtenThy−/− mice fed pioglitazone or control diet

The above results suggest that pioglitazone may induce an infiltration of immune cells into the thyroid glands of PPFP^Thy^;Pten^Thy−/−^ mice. A previous publication identified an “immunome,” a set of 577 human genes whose expression pattern differentiates types of intratumoral immune cells from each other [9]. We created a heat map of this immunome from our RNA-seq data (Supplementary Figure S3) and combined the gene-level RNA-seq data to achieve an overall gene expression score for each type of immune cell (Figure 4). The data suggest that pioglitazone may induce the infiltration of immune cells such as macrophages and T cells into PPFP^Thy^;Pten^Thy−/−^ thyroid glands. Analysis of the Affymetrix data yielded similar findings (Supplementary Figure S4). Accordingly, we performed immunohistochemistry using F4/80 as a macrophage marker and CD3e as a T cell marker. The results show infiltration of the pioglitazone-exposed PPFP^Thy^;Pten^Thy−/−^ thyroid glands with both macrophages and T cells, but staining was not observed in control diet PPFP^Thy^;Pten^Thy−/−^ mice, nor in Pten^Thy−/−^ mice ± pioglitazone (Figure 5), indicating that the immune infiltration is dependent on both PPFP and pioglitazone being present.

## DISCUSSION

The majority of thyroid carcinomas contain one of a small number of oncogenic driver mutations such as BRAF V600E or an activating mutation in one of the *RAS* genes (reviewed in [10]). Oncogenic gene fusions also are found such as those involving *RET*, or fusions of *PAX8* to *PPARG*. The *PAX8*-*PPARG* gene fusion is a consequence of a t(2;3)(q13;p25) chromosomal translocation and leads to production of a PAX8-PPARG fusion protein, PPFP [1]. PPFP is found in ~30% of follicular thyroid carcinomas, ~5% of follicular variant papillary carcinomas, and a small fraction of both poorly differentiated thyroid carcinomas and apparently benign adenomas. An unusual feature of PPFP is that it contains the DBDs of both parent proteins, and hence in principle is capable of complex interactions with DNA. The mechanisms that underlie the oncogenic nature of PPFP are incompletely understood. Insight into this question has been provided by the PPFP^Thy^;Pten^Thy−/−^ transgenic mouse model of PPFP thyroid carcinoma, in which the ligand pioglitazone has remarkable therapeutic efficacy [5].

Here we performed a ChIP-seq analysis of PPFP binding to genomic DNA in the thyroid glands of PPFP^Thy^;Pten^Thy−/−^ mice. We identified ~20,000 PPFP peaks and showed that both DBDs are active *in vivo*, by identifying a significant enrichment of both PAX8 and PPARG binding motifs near the center of PPFP peaks. PPFP binds to genes involved in fatty acid metabolism, which is consistent with the PPARG-like activity of the fusion protein. PPFP also binds to genes involved in cancer-related processes such as the cell cycle, consistent with its oncogenic function. It is likely that the PAX8 and PPARG DBDs subserve different functions within PPFP, as certain processes are associated with PPFP peaks that only contain PAX8 motifs, while others are associated with peaks that only contain PPARG motifs. Not surprisingly, all identified fatty acid and lipid- related processes were associated with PPFP peaks that only contain PPARG motifs. The same is the case for the cell cycle and division, locomotion/motility, and genes involved in Wnt signaling. In contrast, genes involved in the response to hypoxia, cell junctions, or the cytoskeleton are enriched in PPFP peaks that only contain PAX8 motifs.

It would be ideal to have ChIP-seq data from human PPFP thyroid carcinomas, but obtaining such data would be extremely difficult since these tumors constitute only a small fraction of all thyroid cancers and a gene mutation analysis is generally not performed pre-operatively in thyroid cancers. However, the results of this mouse thyroid gland ChIP-seq are highly concordant with a ChIP-seq dataset that we previously published on PPFP stably expressed in the PCCL3 rat thyroid cell line [11], which supports the validity of both models. For example, both ChIP-seq studies showed enrichment of PPFP peaks within 1 kb of TSS’s, and both showed that most peaks contain a PAX8 motif and/or a PPARG motif near the peak center, indicating that both DBDs within PPFP are functional. In addition, 122 (73%) of the 167 significantly enriched GO terms in the PCCL3 rat cell line ChIP-seq experiment were also enriched in the current mouse thyroid ChIP-seq data, including 48 of the 50 GO terms with the lowest *q*-values in the rat cell line (Supplementary Table S10). The enriched GO terms in both ChIP-seq studies include terms related to fatty acid metabolism and cancer-related processes such as the cell cycle, motility and development. However, a key difference was that the rat cell line data showed no indication of an immune cell infiltration, illustrating a critical limitation of *in vitro* experiments and the key importance of the tumor microenvironment in understanding the therapeutic mechanisms of candidate treatments.

Although a ChIP-seq study of human PPFP carcinomas has not been performed, a gene expression analysis has [8]. Using Affymetrix GeneChips, a set of 275 genes has been identified that were differentially expressed in PPFP carcinomas versus non-PPFP thyroid carcinomas and normal thyroids. A significant number of these human PPFP-responsive genes were also regulated in the same direction by PPFP in our mouse model of thyroid cancer, supporting the validity of the mouse model.

An unexpected finding in our RNA-seq analysis relates to the relatively large number of genes and gene sets induced by pioglitazone in PPFP^Thy^;Pten^Thy−/−^ mice. A total of 2012 (66%) of the 3047 genes differentially regulated by pioglitazone were induced. PPFP in the absence of pioglitazone differentially regulated a similar number of genes (2923), but only 44% were induced (PPFP^Thy^;Pten^Thy−/−^ versus Pten^Thy−/−^ mice). Consistent with this difference, pioglitazone induced 17 times more gene sets in PPFP^Thy^;Pten^Thy−/−^ mice than did PPFP in the absence of pioglitazone. A striking aspect of the pioglitazone-induced gene sets is that they are heavily related to immune function, including 18 of the 20 top ranked gene sets (by *q*-value). This was not seen in the PCCL3 PPFP rat thyroid cell line RNA-seq data, in which 52 gene sets were induced by pioglitazone but none were directly related to immune function [11], suggesting the strong immune response is due to being *in vivo*. Another striking aspect is that 17 of these top 18 immune-related gene sets were not enriched in PPFP ChIP-seq peaks, implying an indirect relationship to PPFP. Together, these data suggest that, *in vivo*, pioglitazone may induce an immune cell infiltration into PPFP thyroid glands. This hypothesis was confirmed by immunohistochemistry, which showed that both T cells and macrophages are found in PPFP^Thy^;Pten^Thy−/−^ thyroids specifically in response to pioglitazone, but not in Pten^Thy−/−^ thyroids. We speculate that this immune cell infiltration is central to the therapeutic effect of pioglitazone, and future studies will be directed at testing this hypothesis.

## MATERIALS AND METHODS

### Mice

PPFP^Thy^;Pten^Thy−/−^ and Pten^Thy−/−^ mice on a pure FVB/N background have been described and were previously denoted PPFP;PtenFF;Cre and PtenFF;Cre mice, respectively [5]. In these mice, PPFP has a 3xMyc epitope at the amino terminus. Expression of PPFP and deletion of *Pten* are Cre-dependent and are thyroid-specific due to expression of Cre from the *thyroid peroxidase* promoter. Where indicated, mice were fed pioglitazone at a concentration of 200 parts per million in irradiated Purina rodent diet 5001 (Research Diets, Inc., New Brunswick, NJ). Pioglitazone was administered for two weeks prior to sacrifice for RNA-seq studies, and for two days prior to sacrifice for ChIP-seq studies. Mice were 8–10 weeks of age at the time of sacrifice. All procedures were approved by the Institutional Animal Care and Use Committee of the University of Michigan.

### ChIP-seq assay

Thyroid glands (~250 mg) were minced on ice with a scalpel to 1–2 mm pieces. The tissue was crosslinked with 1% formaldehyde in 10 mL phosphate buffered saline for 15 minutes at room temperature, then quenched by adding glycine to 0.125 M. The tissue was subjected to Dounce homogenization in 10 mL of 10 mM Tris pH 8, 85 mM KCl, 0.5% NP-40. The nuclei were pelleted and resuspended in 1.25 mL of 10 mM Tris pH 7.5, 1% SDS, 10 mM EDTA, 1 mM PMSF and Pierce protease inhibitor cocktail (Thermo Fisher Scientific catalog #88666, Waltham, MA). The tissue was sheared by passage through a 20 gauge needle, then a 25 gauge needle, followed by sonication in a Diagenode Bioruptor (Denville, NJ). The material was diluted 10-fold with 50 mM Tris pH 8, 150 mM NaCl, 1.1% Triton X-100, 1 mM EDTA, 1 mM PMSF and protease inhibitor cocktail, and then gently rocked overnight at 4C with 1:100 anti-Myc tag antibody 9B11 (Cell Signaling Technology #2276, Danvers, MA). Antibody complexes were isolated with Protein G Dynabeads (Thermo Fisher Scientific). The beads were then washed twice with 20 mM Tris pH 8, 150 mM NaCl, 0.1% SDS, 1% Triton X-100, 1 mM EDTA, then three times with the same buffer with 0.1% sodium deoxycholate. Protein-DNA complexes were eluted off the beads by incubation for 30 min at 30C with 0.1 mL of 1 mg/mL Myc peptide (Sigma-Aldrich catalog #M2435, St. Louis, MO) in phosphate buffered saline. The eluate and 30 μL of input DNA (material not subjected to immunoprecipitation) were diluted 1:1 with 50 mM Tris pH 8, 10 mM EDTA, 1% SDS, and decrosslinked by incubation overnight at 65C. These materials were digested with 0.25 mg/mL proteinase K, phenol/chloroform extracted, and precipitated with glycogen and ethanol. The samples were dissolved in 25 μL of 10 mM Tris pH 8, 1 mM EDTA and 0.1 μL of Roche DNase-free RNase (stock 0.5 mg/mL, Sigma-Aldrich catalog #11119915001). The ChIP and input DNA samples were used for next generation library construction and DNA sequencing on an Illumina HiSeq 2000 per the manufacturer's protocols using 50 nt single-end reads, performed by the University of Michigan DNA Sequencing Core. Two independent experiments were performed, each using one mouse thyroid gland. For one experiment the library was constructed with WaferGen Biosystems (Fremont, CA) reagent kit #400040 resulting in 83 and 107 million reads sequenced for the PPFP ChIP and input samples, respectively. Library construction for the second experiment was with a Rubicon Genomics (Ann Arbor, MI) ThruPlex kit resulting in 58 and 46 million reads sequenced.

### RNA-seq assay

RNA was isolated from thyroid glands using an RNeasy Mini Kit per the manufacturer's protocol (Qiagen, Valencia, CA). Samples for analysis included thyroid glands from six PPFP^Thy^;Pten^Thy−/−^ mice on control diet and five on pioglitazone, as well as eight Pten^Thy−/−^ mice on control diet. Library construction (Illumina TruSeq RNA kit #RS-122–2001) and sequencing on an Illumina HiSeq2000 using 50 nt paired end reads were per the manufacturer's protocols, performed by the University of Michigan DNA Sequencing Core. The samples were barcoded and run in eight lanes (each sample in two lanes); an average of 88 million reads were sequenced per sample.

### ChIP-seq data analysis

The quality of the reads was assessed using FastQC [12]. ChIP-seq and input reads were aligned to the mouse reference genome (mm9) using BWA (version 0.5.9-r16) with default options. PePr (v1.0.9) [13] was used to identify consistent PPFP peaks across the replicates using *q*-value < 1e-12 as the cutoff. Peaks that intersected with ENCODE blacklisted regions were removed [14]. The median peak width was 2238 bp with a standard deviation of 1470. Motif presence positively correlated with query DNA length, so to remove potential confounding effects of the varying peak widths in the motif analysis, the peak boundaries were refined as 200bp from the peak mode. Over-represented motifs in the peaks were searched for using MEME-CHIP (v4.9.1) [15] with default parameters. The most over-represented motif (shown in Figure 2A) was nearly identical to the PPARG motif previously reported [16, 17] and was used as the position weight matrix (PWM) for the PPARG motif in subsequent analyses. The PAX8 PWM in Figure 2A and used in subsequent analyses is from [11] and is nearly identical to that in [18]. Motif occurrences in the peaks were identified by FIMO [19] using default parameters and the above PWMs.

Peaks were annotated to genomic features (-1 to +1 kb relative to the TSS, -1 to -5 kb, -5 to -10 kb, exon, UTR, intron, and intergenic) using the HOMER annotatePeaks script as described previously [11]. If a peak had two or more annotations, priority was assigned based on the above order from left to right. As a comparison, we also generated the same number of peaks of the same width randomly across the genome and annotated them in the same way (repeated 10 times and the averages were reported). PPFP peak distribution was compared to that of the randomly generated peaks and fold enrichment was reported (Figure 1).

### RNA-seq data analysis

Quality checks were performed on RNA-seq reads using RSeQC (2.3.9) [20]. The reads were aligned to mm9 with Tophat2 (v2.0.11) [21] and gene expression levels were quantified by HTseq (0.6.1p1) [22] with option “-m intersection-strict” and normalized with edgeR (3.2.4) Bioconductor package [23]. Differential expression analysis was performed using edgeR with tagwise dispersion option for pairwise comparison of PPFP^Thy^;Pten^Thy−/−^ mice versus Pten^Thy−/−^ mice with control diet, and PPFP^Thy^;Pten^Thy−/−^ mice with control diet versus with pioglitazone. False discovery rate (FDR) was controlled using the Benjamini-Hochberg method [24].

### Protein interaction network analysis

Genes that were annotated to PPFP peaks with only PAX8, or only PPARG motifs were used for constructing a protein interaction network. To make the network scale manageable, we chose the genes using the following criteria: *i)* the distance between a gene's TSS and PPFP peak was less than 10 kb; *ii)* the genes were differentially expressed in PPFP^Thy^;Pten^Thy−/−^ mice compared with Pten^Thy−/−^ control mice; and *iii)* the genes were associated with at least one enriched GO term. In total, 183 genes with only PPARG motifs and 143 genes with only PAX8 motifs were selected. MetaCore by Thomson Reuters ( http://thomsonreuters.com/metacore/) [25] was used to model interactions among these genes. The shortest path algorithm (maximum step = 1) was applied to generate the network for each of the gene sets respectively, and the two networks were merged. The largest component of the merged network was chosen to illustrate the interactions among the genes of interest.

### Immune cell type scores

Signature genes for each immune cell subtype were obtained from Bindea et al. [9]. The mouse homolog genes were identified using this human gene list and homolog identifiers from NCBI homoloGene. The gene expression levels were normalized across all samples (deducted by mean and divided by standard deviation). The activity score of each cell type was calculated as the sum of the normalized expression of every gene belonging to that cell type. Only subtypes having >5 marker genes were reported.

### Microarray analysis

Microarray analysis was performed using Affymetrix (Santa Clara, CA) mouse 430 2.0 GeneChips. Samples for analysis included thyroid RNA from six mice in each of the following four groups: PPFP^Thy^;Pten^Thy−/−^ mice on control diet or pioglitazone, and Pten^Thy−/−^ mice on control diet or pioglitazone. Samples were processed using the Ambion (Foster City, CA) Premier kit and analyzed by the University of Michigan Microarray/DNA sequencing Core. Differential expression analysis was performed using the limma R package [26]. The data were normalized using RMA with default settings and the eBayes function was applied to test for significant differences between the four groups. FDR was controlled using the Benjamini-Hochberg method [24].

### Gene set enrichment testing

Gene IDs, *p*-values and fold changes from RNA-seq differential expression analyses were used as input in LRpath [27, 28] to perform gene set enrichment testing. We tested GO terms and KEGG pathways with the directional test option, which identifies gene sets enriched with up-regulated or down-regulated genes. Gene sets with FDR ≤ 0.05 were considered significant.

Gene set enrichment testing on ChIP-seq peaks was performed with ChIP-Enrich, a logistic-regression based method that uses a smoothing spline to empirically adjust for gene locus length and mappability [29]. The “Nearest-to-TSS” option for peak to gene assignment was used and Gene Ontology was tested. Multiple testing correction was performed using the Benjamini-Hochberg method [24] and gene sets with FDR ≤ 0.05 were considered significant.

### Immunohistochemistry

CD3e antibody MA5-14524 and F4/80 antibody PA5-32399 were obtained from Thermo Fisher Scientific. Immunohistochemistry was performed on formalin fixed, paraffin embedded thyroids using the CD3e antibody at 1:150 and the F4/80 antibody at 1:100 with Cell Signaling Technology immunohistochemistry kit #13079 per the manufacturer's protocol.

### Data sharing

The ChIP-seq, RNA-seq and microarray data have been deposited in Gene Expression Omnibus (GEO) with the accession ID GSE85650.

## SUPPLEMENTARY MATERIALS FIGURES AND TABLES




