# Supplementary material for: Genomic binding of PAX8-PPARG fusion protein regulates cancer-related pathways and alters the immune landscape of thyroid cancer
Source: Oncotarget. 2016 Dec 20;8(4):5761–73. doi: 10.18632/oncotarget.14050 (PMC5351587; doi:10.18632/oncotarget.14050)
Supplement: Supplementary file 1 [file oncotarget-08-5761-s001.pdf]

Genomic binding of PAX8-PPARG fusion protein regulates cancer-related pathways and alters the immune landscape of thyroid cancer

Supplementary Materials

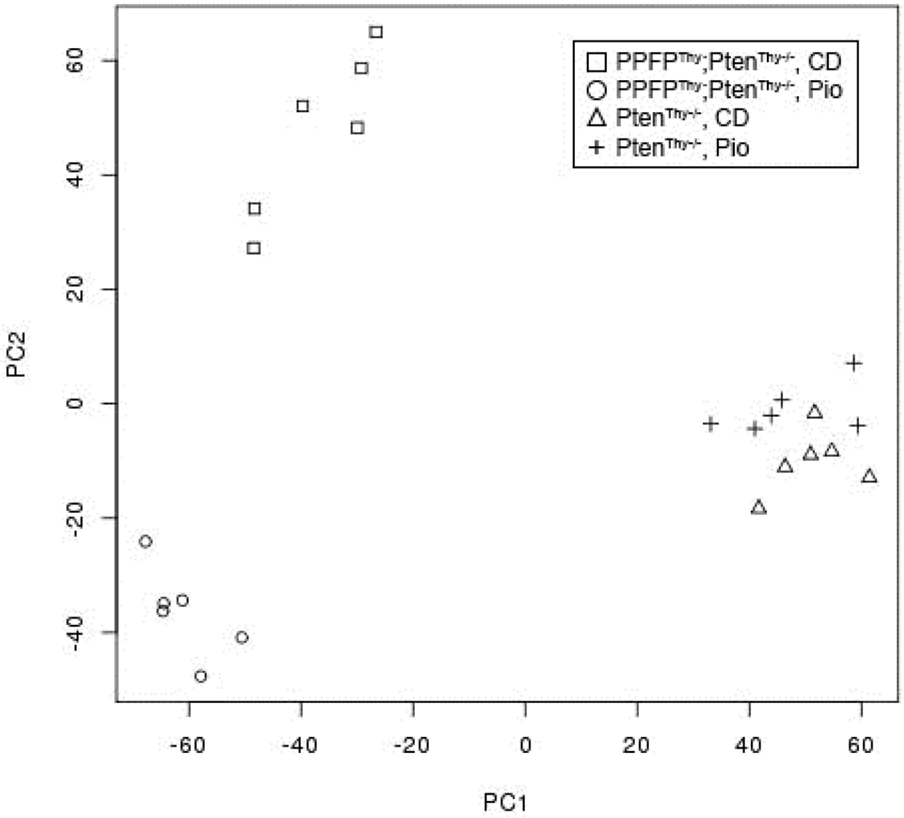

Supplementary Figure S1: Principal component analysis of gene expression in thyroid glands of PPFP^{Thy};Pten$^{Thy-/-}$ and Pten$^{Thy-/-}$ mice fed pioglitazone (Pio) or control diet (CD). The first two principal components are plotted from all probe sets analyzed with Affymetrix mouse 430 2.0 GeneChips. *PC1*, first principal component; *PC2*, second principal component.

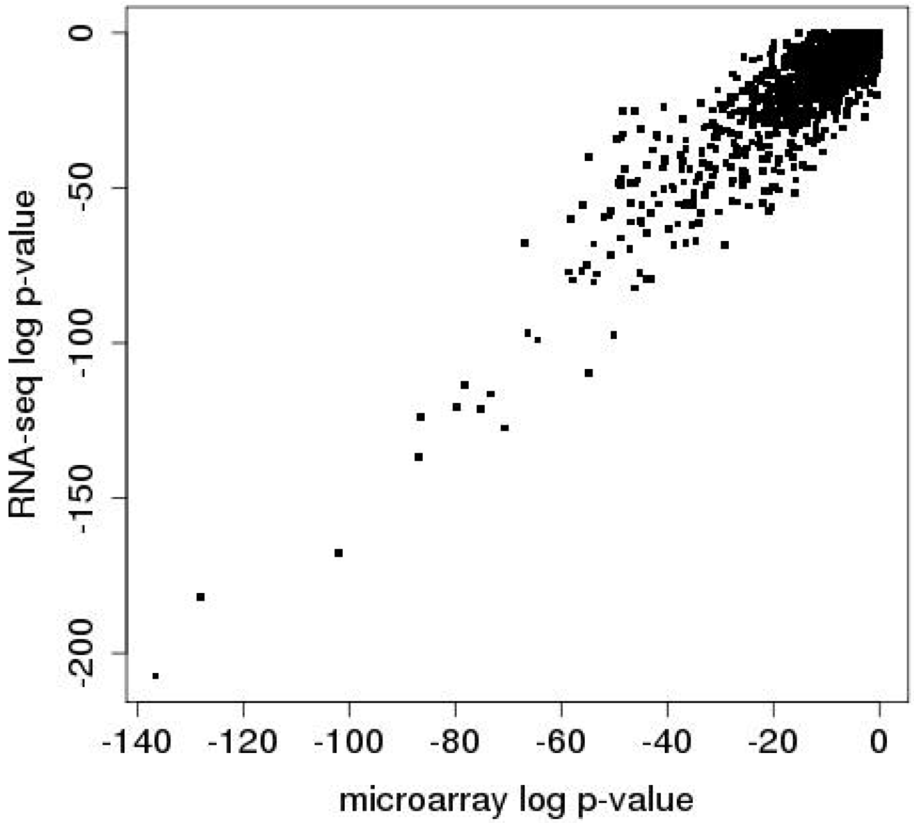

Supplementary Figure S2: Correlation of gene sets regulated by pioglitazone in PPFP^{Thy};Pten$^{Thy-/-}$ mice analyzed by RNA-seq or Affymetrix microarray GeneChips (R = 0.90). The analyses were done on independent sets of mice.

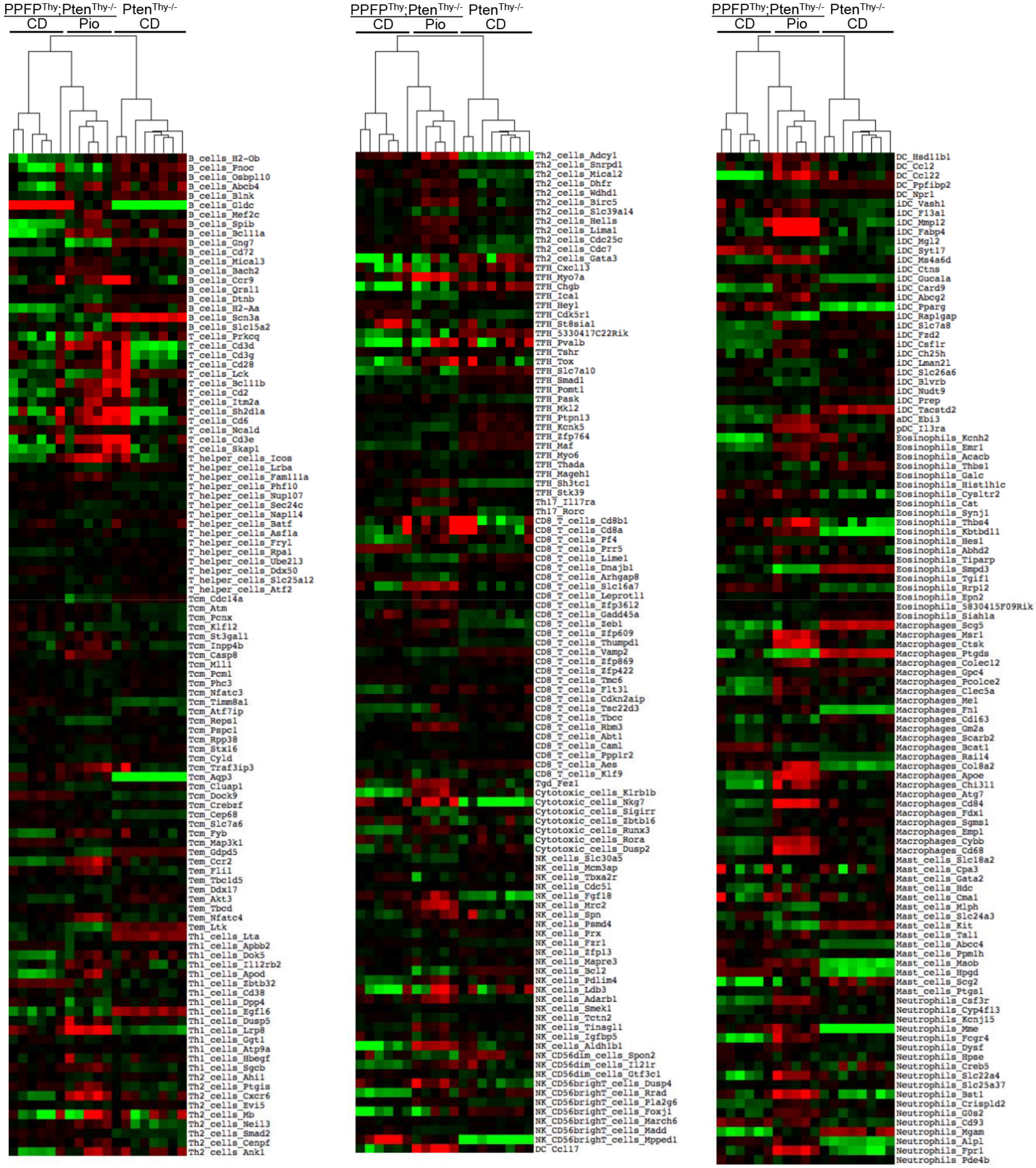

Supplementary Figure S3: RNA-seq analysis of the immunome in thyroid glands of PPFP^{Thy};Pten$^{Thy-/-}$ mice fed control diet (CD) or pioglitazone (Pio), or Pten$^{Thy-/-}$ mice fed CD.

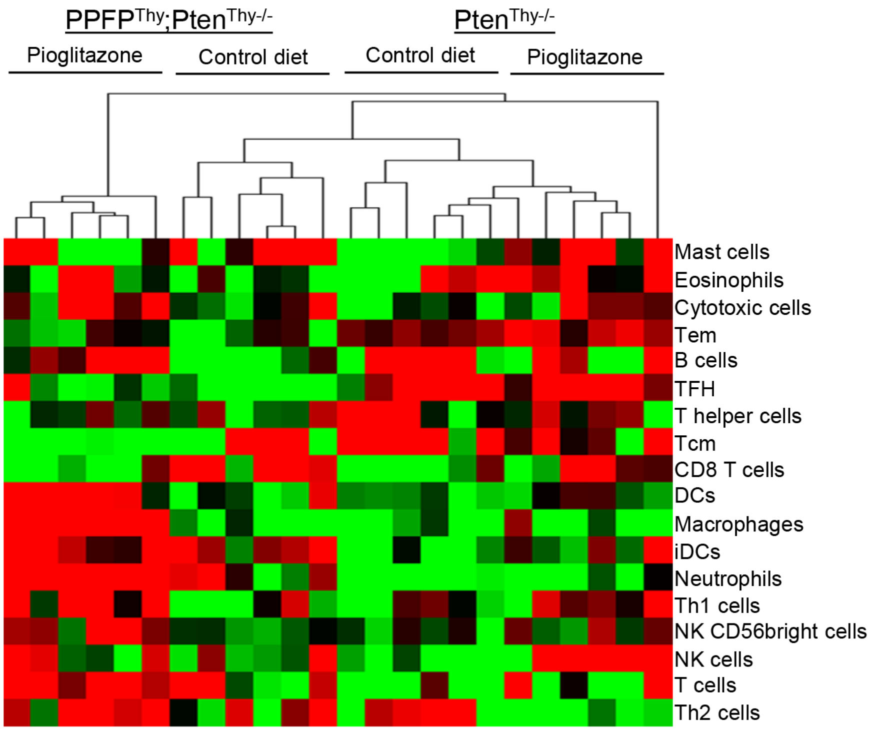

Supplementary Figure S4: Heat map of overall gene expression scores for types of immune cells in thyroid glands of PPFP^{Thy};Pten$^{Thy-/-}$ mice and Pten$^{Thy-/-}$ mice fed pioglitazone or control diet. Gene expression was measured using Affymetrix microarray GeneChips.. The samples were clustered by hierarchical clustering method using average linkage and the correlation distance measure. The cell types follow the same order of the cell types in Figure 3 (RNA-seq data). Abbreviations: Tem, T effector memory; TFH, T follicular helper; Tcm, T central memory; DCs, dendritic cells; iDCs, immature DCs; Th1, T helper 1; NK, natural killer; Th2, T helper 2.

Supplementary Table S1: Gene Ontology terms enriched with PPFP peaks. Peaks were associated with the gene with the nearest transcription start site. See Supplementary_Table_S1

Supplementary Table S2: Gene Ontology terms enriched with PPFP peaks that contain only a PPARG motif, or only a PAX8 motif. See Supplementary_Table_S2

Supplementary Table S3: Gene Ontology terms enriched in genes that contain PPFP peaks < 10 kb from a TSS, or 10-100 kb from a TSS, or both. GO terms are grouped based on key words within the gene set names.. See Supplementary_Table_S3

Supplementary Table S4: Thyroid genes differentially expressed in $PPFP^{Thy}$;$Pten^{Thy-/-}$ mice versus $Pten^{Thy-/-}$ mice on a normal chow diet, using q < 0.05 and absolute fold change >2 as cut-offs for significance. See Supplementary_Table_S4

Supplementary Table S5: Comparison of gene expression changes in human PPFP thyroid carcinomas versus mouse PPFP thyroid carcinomas. Genes previously found to be differentially expressed in human PPFP thyroid carcinomas versus other thyroid neoplasms and normal thyroids [8] were assessed for differential expression in $PPFP^{Thy}$; $Pten^{Thy-/-}$ mice versus $Pten^{Thy-/-}$ control mice fed normal chow (-pio), and in $PPFP^{Thy}$; $Pten^{Thy-/-}$ mice fed pioglitazone versus normal chow. We define mouse genes to be induced if log fold change > 0, and repressed if log fold change < 0. See Supplementary_Table_S5

Supplementary Table S6: Thyroid genes differentially expressed in $PPFP^{Thy}$;$Pten^{Thy-/-}$ mice fed pioglitazone versus control diet, using q < 0.05 and absolute fold change >2 as cut-offs for significance. See Supplementary_Table_S6

Supplementary Table S7: Gene sets induced or repressed in thyroid glands from $PPFP^{Thy}$;$Pten^{Thy-/-}$ mice versus $Pten^{Thy-/-}$ mice on a control diet assessed by RNA-seq, and the enrichment status of these gene sets in the PPFP ChIP-seq. Gene sets are grouped into categories based on key words in the gene set names, and are then sorted by direction of change in RNA-seq, then significance in ChIP-seq, and then RNA-seq q-value. See Supplementary_Table_S7

Supplementary Table S8: Gene sets induced or repressed in thyroid glands from PPFP^{Thy};Pten$^{Thy-/-}$ mice fed pioglitazone versus a control diet assessed by RNA-seq, and the enrichment status of these gene sets in the PPFP ChIP-seq. Gene sets are grouped into categories based on key words in the gene set names, and are then sorted by direction of change in RNA-seq, then significance in ChIP-seq, and then RNA-seq *q*-value. See Supplementary_Table_S8

Supplementary Table S9: Gene sets differentially regulated in the thyroid glands of PPFP^{Thy};Pten$^{Thy-/-}$ mice fed pioglitazone versus control diet. Gene expression was analyzed with Affymetrix microarray GeneChips. See Supplementary_Table_S9

Supplementary Table S10: Gene Ontology terms enriched in a PPFP ChIP-seq from the rat thyroid PCCL3 cell line [11] were tested for enrichment in the mouse thyroid gland PPFP ChIP-seq study. See Supplementary_Table_S10
