# Supplementary material for: Genomic binding of PAX8-PPARG fusion protein regulates cancer-related pathways and alters the immune landscape of thyroid cancer
Source: Oncotarget. 2016 Dec 20;8(4):5761–73. doi: 10.18632/oncotarget.14050 (PMC5351587; doi:10.18632/oncotarget.14050)
Supplement: Supplementary file 2 [file oncotarget-08-5761-s002.docx]

**Table S1: Gene Ontology terms enriched with PPFP peaks. Peaks were associated with the gene with the nearest transcription start site**

| **Geneset.ID** | **Description** | ***p*-value** | ***q*-value** |
| --- | --- | --- | --- |
| GO:0006793 | phosphorus metabolic process | 1.58E-22 | 6.31E-20 |
| GO:0044281 | small molecule metabolic process | 3.91E-22 | 1.42E-19 |
| GO:0033036 | macromolecule localization | 2.36E-20 | 7.87E-18 |
| GO:0016310 | phosphorylation | 8.97E-20 | 2.76E-17 |
| GO:0009056 | catabolic process | 2.26E-19 | 6.44E-17 |
| GO:0051641 | cellular localization | 1.21E-18 | 3.23E-16 |
| GO:0044248 | cellular catabolic process | 7.70E-17 | 1.92E-14 |
| GO:0046907 | intracellular transport | 1.01E-16 | 2.37E-14 |
| GO:0008104 | protein localization | 3.21E-16 | 7.14E-14 |
| GO:0009966 | regulation of signal transduction | 3.49E-16 | 7.34E-14 |
| GO:0035556 | intracellular signal transduction | 4.41E-16 | 8.82E-14 |
| GO:0006629 | lipid metabolic process | 7.31E-16 | 1.39E-13 |
| GO:0010604 | positive regulation of macromolecule metabolic process | 1.44E-15 | 2.62E-13 |
| GO:0006996 | organelle organization | 1.72E-15 | 2.98E-13 |
| GO:0065009 | regulation of molecular function | 1.83E-15 | 3.01E-13 |
| GO:0045184 | establishment of protein localization | 1.88E-15 | 3.01E-13 |
| GO:0051649 | establishment of localization in cell | 2.54E-15 | 3.90E-13 |
| GO:0015031 | protein transport | 4.83E-15 | 7.15E-13 |
| GO:0009893 | positive regulation of metabolic process | 9.75E-15 | 1.39E-12 |
| GO:0034613 | cellular protein localization | 1.24E-14 | 1.71E-12 |
| GO:0070727 | cellular macromolecule localization | 1.50E-14 | 2.00E-12 |
| GO:0006468 | protein phosphorylation | 4.64E-14 | 5.98E-12 |
| GO:0042180 | cellular ketone metabolic process | 5.17E-14 | 6.46E-12 |
| GO:0031325 | positive regulation of cellular metabolic process | 1.23E-13 | 1.49E-11 |
| GO:0016265 | death | 1.45E-13 | 1.71E-11 |
| GO:0023051 | regulation of signaling | 1.66E-13 | 1.90E-11 |
| GO:0008219 | cell death | 1.82E-13 | 2.02E-11 |
| GO:0012501 | programmed cell death | 2.00E-13 | 2.17E-11 |
| GO:0044255 | cellular lipid metabolic process | 2.89E-13 | 3.04E-11 |
| GO:0006915 | apoptotic process | 5.34E-13 | 5.47E-11 |
| GO:0019752 | carboxylic acid metabolic process | 5.89E-13 | 5.88E-11 |
| GO:0044093 | positive regulation of molecular function | 1.20E-12 | 1.17E-10 |
| GO:0048583 | regulation of response to stimulus | 5.44E-12 | 5.17E-10 |
| GO:0006082 | organic acid metabolic process | 5.64E-12 | 5.24E-10 |
| GO:0006886 | intracellular protein transport | 1.34E-11 | 1.22E-09 |
| GO:1901135 | carbohydrate derivative metabolic process | 2.95E-11 | 2.62E-09 |
| GO:0016192 | vesicle-mediated transport | 3.72E-11 | 3.24E-09 |
| GO:0007010 | cytoskeleton organization | 4.56E-11 | 3.88E-09 |
| GO:0071704 | organic substance metabolic process | 6.00E-11 | 4.99E-09 |
| GO:0032268 | regulation of cellular protein metabolic process | 7.83E-11 | 6.39E-09 |
| GO:0032787 | monocarboxylic acid metabolic process | 8.37E-11 | 6.69E-09 |
| GO:0022607 | cellular component assembly | 1.56E-10 | 1.22E-08 |
| GO:0009892 | negative regulation of metabolic process | 1.82E-10 | 1.40E-08 |
| GO:0019637 | organophosphate metabolic process | 3.34E-10 | 2.52E-08 |
| GO:0006366 | transcription from RNA polymerase II promoter | 3.47E-10 | 2.57E-08 |
| GO:0006631 | fatty acid metabolic process | 4.00E-10 | 2.86E-08 |
| GO:0042325 | regulation of phosphorylation | 4.00E-10 | 2.86E-08 |
| GO:0006605 | protein targeting | 4.31E-10 | 3.02E-08 |
| GO:0010605 | negative regulation of macromolecule metabolic process | 4.64E-10 | 3.20E-08 |
| GO:0051338 | regulation of transferase activity | 5.43E-10 | 3.68E-08 |
| GO:0044085 | cellular component biogenesis | 5.55E-10 | 3.70E-08 |
| GO:0010557 | positive regulation of macromolecule biosynthetic process | 5.76E-10 | 3.78E-08 |
| GO:0043549 | regulation of kinase activity | 5.96E-10 | 3.85E-08 |
| GO:0050790 | regulation of catalytic activity | 8.70E-10 | 5.52E-08 |
| GO:0010628 | positive regulation of gene expression | 9.26E-10 | 5.78E-08 |
| GO:0019220 | regulation of phosphate metabolic process | 1.71E-09 | 1.05E-07 |
| GO:0046434 | organophosphate catabolic process | 1.76E-09 | 1.05E-07 |
| GO:0051254 | positive regulation of RNA metabolic process | 1.76E-09 | 1.05E-07 |
| GO:0005975 | carbohydrate metabolic process | 1.78E-09 | 1.05E-07 |
| GO:0006357 | regulation of transcription from RNA polymerase II promoter | 1.83E-09 | 1.06E-07 |
| GO:0043085 | positive regulation of catalytic activity | 1.87E-09 | 1.07E-07 |
| GO:0046483 | heterocycle metabolic process | 2.79E-09 | 1.57E-07 |
| GO:0045893 | positive regulation of transcription, DNA-dependent | 3.07E-09 | 1.70E-07 |
| GO:0007264 | small GTPase mediated signal transduction | 3.43E-09 | 1.88E-07 |
| GO:0031399 | regulation of protein modification process | 3.58E-09 | 1.93E-07 |
| GO:0042278 | purine nucleoside metabolic process | 3.92E-09 | 2.09E-07 |
| GO:0050793 | regulation of developmental process | 4.52E-09 | 2.38E-07 |
| GO:0045859 | regulation of protein kinase activity | 4.99E-09 | 2.59E-07 |
| GO:0031324 | negative regulation of cellular metabolic process | 6.87E-09 | 3.52E-07 |
| GO:0046128 | purine ribonucleoside metabolic process | 7.21E-09 | 3.65E-07 |
| GO:1901292 | nucleoside phosphate catabolic process | 7.94E-09 | 3.97E-07 |
| GO:0009119 | ribonucleoside metabolic process | 9.19E-09 | 4.53E-07 |
| GO:0009116 | nucleoside metabolic process | 1.04E-08 | 5.03E-07 |
| GO:0071822 | protein complex subunit organization | 1.04E-08 | 5.03E-07 |
| GO:0007243 | intracellular protein kinase cascade | 1.29E-08 | 6.15E-07 |
| GO:0001932 | regulation of protein phosphorylation | 1.41E-08 | 6.63E-07 |
| GO:0034655 | nucleobase-containing compound catabolic process | 1.45E-08 | 6.72E-07 |
| GO:1901136 | carbohydrate derivative catabolic process | 1.55E-08 | 7.14E-07 |
| GO:0009141 | nucleoside triphosphate metabolic process | 1.67E-08 | 7.61E-07 |
| GO:0009166 | nucleotide catabolic process | 1.71E-08 | 7.68E-07 |
| GO:0006195 | purine nucleotide catabolic process | 1.74E-08 | 7.72E-07 |
| GO:0051186 | cofactor metabolic process | 1.92E-08 | 8.46E-07 |
| GO:1901068 | guanosine-containing compound metabolic process | 2.25E-08 | 9.79E-07 |
| GO:0045595 | regulation of cell differentiation | 2.37E-08 | 1.02E-06 |
| GO:0044270 | cellular nitrogen compound catabolic process | 2.48E-08 | 1.05E-06 |
| GO:0051128 | regulation of cellular component organization | 2.54E-08 | 1.07E-06 |
| GO:0070271 | protein complex biogenesis | 2.79E-08 | 1.16E-06 |
| GO:0046700 | heterocycle catabolic process | 2.99E-08 | 1.23E-06 |
| GO:0055086 | nucleobase-containing small molecule metabolic process | 3.04E-08 | 1.24E-06 |
| GO:0009144 | purine nucleoside triphosphate metabolic process | 3.08E-08 | 1.25E-06 |
| GO:0006184 | GTP catabolic process | 3.25E-08 | 1.30E-06 |
| GO:0006461 | protein complex assembly | 3.47E-08 | 1.37E-06 |
| GO:0019318 | hexose metabolic process | 3.63E-08 | 1.42E-06 |
| GO:0009259 | ribonucleotide metabolic process | 3.76E-08 | 1.46E-06 |
| GO:0043933 | macromolecular complex subunit organization | 4.24E-08 | 1.62E-06 |
| GO:0009894 | regulation of catabolic process | 4.26E-08 | 1.62E-06 |
| GO:0045935 | positive regulation of nucleobase-containing compound metabolic process | 4.70E-08 | 1.77E-06 |
| GO:0009143 | nucleoside triphosphate catabolic process | 4.97E-08 | 1.86E-06 |
| GO:0051173 | positive regulation of nitrogen compound metabolic process | 5.17E-08 | 1.91E-06 |
| GO:0031401 | positive regulation of protein modification process | 5.39E-08 | 1.98E-06 |
| GO:0046039 | GTP metabolic process | 5.45E-08 | 1.98E-06 |
| GO:0072523 | purine-containing compound catabolic process | 5.50E-08 | 1.98E-06 |
| GO:0006152 | purine nucleoside catabolic process | 5.59E-08 | 2.00E-06 |
| GO:0006753 | nucleoside phosphate metabolic process | 5.67E-08 | 2.01E-06 |
| GO:0042454 | ribonucleoside catabolic process | 6.09E-08 | 2.14E-06 |
| GO:0071844 | cellular component assembly at cellular level | 6.15E-08 | 2.14E-06 |
| GO:0009890 | negative regulation of biosynthetic process | 6.61E-08 | 2.28E-06 |
| GO:1901069 | guanosine-containing compound catabolic process | 6.76E-08 | 2.31E-06 |
| GO:0009146 | purine nucleoside triphosphate catabolic process | 6.82E-08 | 2.31E-06 |
| GO:0009164 | nucleoside catabolic process | 7.38E-08 | 2.48E-06 |
| GO:0009891 | positive regulation of biosynthetic process | 7.50E-08 | 2.50E-06 |
| GO:0009199 | ribonucleoside triphosphate metabolic process | 7.90E-08 | 2.61E-06 |
| GO:0009150 | purine ribonucleotide metabolic process | 8.82E-08 | 2.89E-06 |
| GO:0051239 | regulation of multicellular organismal process | 8.95E-08 | 2.91E-06 |
| GO:0009117 | nucleotide metabolic process | 9.09E-08 | 2.93E-06 |
| GO:0010941 | regulation of cell death | 9.38E-08 | 3.00E-06 |
| GO:0031329 | regulation of cellular catabolic process | 9.65E-08 | 3.06E-06 |
| GO:0010629 | negative regulation of gene expression | 9.71E-08 | 3.06E-06 |
| GO:0009203 | ribonucleoside triphosphate catabolic process | 1.00E-07 | 3.11E-06 |
| GO:0051246 | regulation of protein metabolic process | 1.01E-07 | 3.11E-06 |
| GO:0009261 | ribonucleotide catabolic process | 1.01E-07 | 3.11E-06 |
| GO:0009888 | tissue development | 1.02E-07 | 3.12E-06 |
| GO:0031327 | negative regulation of cellular biosynthetic process | 1.04E-07 | 3.15E-06 |
| GO:0065003 | macromolecular complex assembly | 1.06E-07 | 3.18E-06 |
| GO:0033365 | protein localization to organelle | 1.09E-07 | 3.23E-06 |
| GO:0009205 | purine ribonucleoside triphosphate metabolic process | 1.09E-07 | 3.23E-06 |
| GO:0051056 | regulation of small GTPase mediated signal transduction | 1.13E-07 | 3.34E-06 |
| GO:0009154 | purine ribonucleotide catabolic process | 1.28E-07 | 3.73E-06 |
| GO:0009207 | purine ribonucleoside triphosphate catabolic process | 1.31E-07 | 3.79E-06 |
| GO:0033554 | cellular response to stress | 1.47E-07 | 4.24E-06 |
| GO:0010558 | negative regulation of macromolecule biosynthetic process | 1.65E-07 | 4.72E-06 |
| GO:0032270 | positive regulation of cellular protein metabolic process | 1.87E-07 | 5.30E-06 |
| GO:0006732 | coenzyme metabolic process | 2.03E-07 | 5.71E-06 |
| GO:0007049 | cell cycle | 2.26E-07 | 6.32E-06 |
| GO:0005996 | monosaccharide metabolic process | 2.52E-07 | 7.00E-06 |
| GO:0010627 | regulation of intracellular protein kinase cascade | 2.60E-07 | 7.17E-06 |
| GO:0031328 | positive regulation of cellular biosynthetic process | 2.98E-07 | 8.16E-06 |
| GO:2000113 | negative regulation of cellular macromolecule biosynthetic process | 3.45E-07 | 9.37E-06 |
| GO:0030029 | actin filament-based process | 3.54E-07 | 9.56E-06 |
| GO:0010562 | positive regulation of phosphorus metabolic process | 3.64E-07 | 9.77E-06 |
| GO:0043067 | regulation of programmed cell death | 3.73E-07 | 9.95E-06 |
| GO:0070887 | cellular response to chemical stimulus | 4.28E-07 | 1.13E-05 |
| GO:0010646 | regulation of cell communication | 4.82E-07 | 1.27E-05 |
| GO:0051726 | regulation of cell cycle | 5.39E-07 | 1.41E-05 |
| GO:0009967 | positive regulation of signal transduction | 5.75E-07 | 1.49E-05 |
| GO:0042981 | regulation of apoptotic process | 6.58E-07 | 1.70E-05 |
| GO:0042327 | positive regulation of phosphorylation | 7.38E-07 | 1.89E-05 |
| GO:0042127 | regulation of cell proliferation | 7.92E-07 | 2.02E-05 |
| GO:0008610 | lipid biosynthetic process | 9.41E-07 | 2.37E-05 |
| GO:0072521 | purine-containing compound metabolic process | 9.55E-07 | 2.39E-05 |
| GO:0001934 | positive regulation of protein phosphorylation | 1.21E-06 | 2.99E-05 |
| GO:0032879 | regulation of localization | 1.21E-06 | 2.99E-05 |
| GO:0010876 | lipid localization | 1.22E-06 | 2.99E-05 |
| GO:0051247 | positive regulation of protein metabolic process | 1.41E-06 | 3.44E-05 |
| GO:0008283 | cell proliferation | 1.59E-06 | 3.86E-05 |
| GO:2000026 | regulation of multicellular organismal development | 1.62E-06 | 3.90E-05 |
| GO:0007167 | enzyme linked receptor protein signaling pathway | 1.68E-06 | 4.01E-05 |
| GO:0009653 | anatomical structure morphogenesis | 1.68E-06 | 4.01E-05 |
| GO:0051253 | negative regulation of RNA metabolic process | 1.73E-06 | 4.10E-05 |
| GO:0046578 | regulation of Ras protein signal transduction | 1.78E-06 | 4.19E-05 |
| GO:0030036 | actin cytoskeleton organization | 2.07E-06 | 4.83E-05 |
| GO:0032386 | regulation of intracellular transport | 2.31E-06 | 5.36E-05 |
| GO:0010647 | positive regulation of cell communication | 2.32E-06 | 5.36E-05 |
| GO:0040007 | growth | 2.35E-06 | 5.41E-05 |
| GO:0007265 | Ras protein signal transduction | 2.66E-06 | 6.08E-05 |
| GO:0045892 | negative regulation of transcription, DNA-dependent | 2.74E-06 | 6.22E-05 |
| GO:0006163 | purine nucleotide metabolic process | 2.79E-06 | 6.30E-05 |
| GO:0006897 | endocytosis | 2.87E-06 | 6.44E-05 |
| GO:0001701 | in utero embryonic development | 2.90E-06 | 6.49E-05 |
| GO:0051172 | negative regulation of nitrogen compound metabolic process | 3.40E-06 | 7.56E-05 |
| GO:0023056 | positive regulation of signaling | 3.79E-06 | 8.38E-05 |
| GO:0051090 | regulation of sequence-specific DNA binding transcription factor activity | 4.05E-06 | 8.89E-05 |
| GO:0033124 | regulation of GTP catabolic process | 4.09E-06 | 8.94E-05 |
| GO:0000226 | microtubule cytoskeleton organization | 4.11E-06 | 8.94E-05 |
| GO:0019216 | regulation of lipid metabolic process | 4.20E-06 | 9.07E-05 |
| GO:0043623 | cellular protein complex assembly | 4.29E-06 | 9.21E-05 |
| GO:0044262 | cellular carbohydrate metabolic process | 4.37E-06 | 9.35E-05 |
| GO:0044242 | cellular lipid catabolic process | 4.68E-06 | 9.95E-05 |
| GO:0045934 | negative regulation of nucleobase-containing compound metabolic process | 5.07E-06 | 0.000107267 |
| GO:0045944 | positive regulation of transcription from RNA polymerase II promoter | 5.21E-06 | 0.0001096 |
| GO:0006066 | alcohol metabolic process | 5.26E-06 | 0.00011009 |
| GO:0043547 | positive regulation of GTPase activity | 5.57E-06 | 0.000115913 |
| GO:0043087 | regulation of GTPase activity | 5.99E-06 | 0.000124038 |
| GO:0010942 | positive regulation of cell death | 6.58E-06 | 0.000135663 |
| GO:0019320 | hexose catabolic process | 6.63E-06 | 0.000135886 |
| GO:0070201 | regulation of establishment of protein localization | 7.47E-06 | 0.000152408 |
| GO:0023057 | negative regulation of signaling | 8.58E-06 | 0.000173829 |
| GO:0030030 | cell projection organization | 8.61E-06 | 0.000173829 |
| GO:0016311 | dephosphorylation | 8.72E-06 | 0.000175237 |
| GO:0045860 | positive regulation of protein kinase activity | 8.83E-06 | 0.000176507 |
| GO:0046365 | monosaccharide catabolic process | 9.12E-06 | 0.000181391 |
| GO:0006006 | glucose metabolic process | 9.50E-06 | 0.000188009 |
| GO:0032880 | regulation of protein localization | 1.03E-05 | 0.000201956 |
| GO:0043068 | positive regulation of programmed cell death | 1.10E-05 | 0.000215803 |
| GO:0033674 | positive regulation of kinase activity | 1.11E-05 | 0.000216102 |
| GO:0071375 | cellular response to peptide hormone stimulus | 1.15E-05 | 0.000222849 |
| GO:0030811 | regulation of nucleotide catabolic process | 1.24E-05 | 0.000239031 |
| GO:0009968 | negative regulation of signal transduction | 1.36E-05 | 0.000261157 |
| GO:0010648 | negative regulation of cell communication | 1.37E-05 | 0.000262423 |
| GO:0043065 | positive regulation of apoptotic process | 1.51E-05 | 0.000288414 |
| GO:0007169 | transmembrane receptor protein tyrosine kinase signaling pathway | 1.69E-05 | 0.000319486 |
| GO:0060341 | regulation of cellular localization | 1.76E-05 | 0.000331387 |
| GO:0048584 | positive regulation of response to stimulus | 1.77E-05 | 0.000331387 |
| GO:0016042 | lipid catabolic process | 1.84E-05 | 0.000343346 |
| GO:0035295 | tube development | 2.05E-05 | 0.000380832 |
| GO:0051258 | protein polymerization | 2.20E-05 | 0.00040659 |
| GO:0060284 | regulation of cell development | 2.22E-05 | 0.000408777 |
| GO:0007399 | nervous system development | 2.51E-05 | 0.000460918 |
| GO:0006928 | cellular component movement | 2.59E-05 | 0.000472437 |
| GO:0006913 | nucleocytoplasmic transport | 2.67E-05 | 0.000483492 |
| GO:0006007 | glucose catabolic process | 2.67E-05 | 0.000483492 |
| GO:0042592 | homeostatic process | 2.70E-05 | 0.000485689 |
| GO:0072358 | cardiovascular system development | 2.71E-05 | 0.000485918 |
| GO:0006412 | translation | 3.02E-05 | 0.000539213 |
| GO:0051223 | regulation of protein transport | 3.19E-05 | 0.000565542 |
| GO:0051347 | positive regulation of transferase activity | 3.20E-05 | 0.000565542 |
| GO:0006869 | lipid transport | 3.35E-05 | 0.000589973 |
| GO:0072329 | monocarboxylic acid catabolic process | 3.50E-05 | 0.000613345 |
| GO:0051169 | nuclear transport | 3.95E-05 | 0.000689291 |
| GO:0071702 | organic substance transport | 4.01E-05 | 0.000697756 |
| GO:0000122 | negative regulation of transcription from RNA polymerase II promoter | 4.08E-05 | 0.000705988 |
| GO:0016477 | cell migration | 4.13E-05 | 0.00071117 |
| GO:0070647 | protein modification by small protein conjugation or removal | 4.64E-05 | 0.000795515 |
| GO:0048468 | cell development | 4.70E-05 | 0.000803158 |
| GO:0048193 | Golgi vesicle transport | 4.72E-05 | 0.000803186 |
| GO:0032147 | activation of protein kinase activity | 4.87E-05 | 0.000824722 |
| GO:0010608 | posttranscriptional regulation of gene expression | 4.89E-05 | 0.000824722 |
| GO:0071310 | cellular response to organic substance | 4.91E-05 | 0.000824722 |
| GO:0034622 | cellular macromolecular complex assembly | 4.94E-05 | 0.000827187 |
| GO:0019915 | lipid storage | 5.33E-05 | 0.000887579 |
| GO:0060548 | negative regulation of cell death | 5.35E-05 | 0.000887579 |
| GO:0051259 | protein oligomerization | 5.77E-05 | 0.000953941 |
| GO:0032868 | response to insulin stimulus | 6.05E-05 | 0.000995083 |
| GO:0034330 | cell junction organization | 6.08E-05 | 0.000996592 |
| GO:0051188 | cofactor biosynthetic process | 6.33E-05 | 0.001032409 |
| GO:0034504 | protein localization to nucleus | 6.56E-05 | 0.001066319 |
| GO:0071900 | regulation of protein serine/threonine kinase activity | 6.81E-05 | 0.00110193 |
| GO:0016053 | organic acid biosynthetic process | 7.01E-05 | 0.001130826 |
| GO:0051348 | negative regulation of transferase activity | 7.53E-05 | 0.001208751 |
| GO:0051049 | regulation of transport | 7.67E-05 | 0.001226809 |
| GO:0007015 | actin filament organization | 7.71E-05 | 0.001228286 |
| GO:0033157 | regulation of intracellular protein transport | 8.08E-05 | 0.001282331 |
| GO:0007005 | mitochondrion organization | 8.17E-05 | 0.00129118 |
| GO:0048870 | cell motility | 8.51E-05 | 0.001339607 |
| GO:0032446 | protein modification by small protein conjugation | 8.90E-05 | 0.001395955 |
| GO:0044283 | small molecule biosynthetic process | 9.32E-05 | 0.001455815 |
| GO:0017038 | protein import | 9.40E-05 | 0.001462255 |
| GO:0046486 | glycerolipid metabolic process | 9.51E-05 | 0.001473258 |
| GO:0006457 | protein folding | 9.55E-05 | 0.001474452 |
| GO:0016052 | carbohydrate catabolic process | 9.66E-05 | 0.001485086 |
| GO:0006644 | phospholipid metabolic process | 9.73E-05 | 0.001491011 |
| GO:0006470 | protein dephosphorylation | 0.000104721 | 0.001594232 |
| GO:0034621 | cellular macromolecular complex subunit organization | 0.000104873 | 0.001594232 |
| GO:0009062 | fatty acid catabolic process | 0.000106129 | 0.001607204 |
| GO:0016567 | protein ubiquitination | 0.000111643 | 0.001680713 |
| GO:0006091 | generation of precursor metabolites and energy | 0.000111823 | 0.001680713 |
| GO:0032318 | regulation of Ras GTPase activity | 0.000112453 | 0.00168384 |
| GO:0008285 | negative regulation of cell proliferation | 0.00011327 | 0.001689752 |
| GO:0019941 | modification-dependent protein catabolic process | 0.000115568 | 0.001717622 |
| GO:0030334 | regulation of cell migration | 0.000117502 | 0.001739894 |
| GO:0006096 | glycolysis | 0.000119441 | 0.001762079 |
| GO:0022603 | regulation of anatomical structure morphogenesis | 0.000120191 | 0.001766629 |
| GO:0043069 | negative regulation of programmed cell death | 0.000126274 | 0.001849245 |
| GO:0034101 | erythrocyte homeostasis | 0.000129863 | 0.001894866 |
| GO:0006511 | ubiquitin-dependent protein catabolic process | 0.000131128 | 0.001906362 |
| GO:0033673 | negative regulation of kinase activity | 0.000141263 | 0.002046273 |
| GO:0061180 | mammary gland epithelium development | 0.000145744 | 0.002103557 |
| GO:0071495 | cellular response to endogenous stimulus | 0.000147513 | 0.002121424 |
| GO:0030258 | lipid modification | 0.000152754 | 0.002188933 |
| GO:0032870 | cellular response to hormone stimulus | 0.000158583 | 0.002264346 |
| GO:0051270 | regulation of cellular component movement | 0.000162275 | 0.002293904 |
| GO:0043066 | negative regulation of apoptotic process | 0.000162514 | 0.002293904 |
| GO:0051098 | regulation of binding | 0.000162608 | 0.002293904 |
| GO:0035023 | regulation of Rho protein signal transduction | 0.000162949 | 0.002293904 |
| GO:0006575 | cellular modified amino acid metabolic process | 0.000165969 | 0.002328225 |
| GO:0030218 | erythrocyte differentiation | 0.000169545 | 0.002370075 |
| GO:0007017 | microtubule-based process | 0.000170956 | 0.00238147 |
| GO:0019395 | fatty acid oxidation | 0.000179562 | 0.00249267 |
| GO:0051248 | negative regulation of protein metabolic process | 0.000189123 | 0.002616305 |
| GO:0032869 | cellular response to insulin stimulus | 0.000193796 | 0.002671715 |
| GO:0061024 | membrane organization | 0.000197302 | 0.002710705 |
| GO:0006635 | fatty acid beta-oxidation | 0.000202377 | 0.002770899 |
| GO:0051493 | regulation of cytoskeleton organization | 0.000207073 | 0.002825517 |
| GO:0030097 | hemopoiesis | 0.00020894 | 0.002835695 |
| GO:0033043 | regulation of organelle organization | 0.000209237 | 0.002835695 |
| GO:0043009 | chordate embryonic development | 0.000226736 | 0.003062465 |
| GO:0016125 | sterol metabolic process | 0.000230111 | 0.003090386 |
| GO:0050767 | regulation of neurogenesis | 0.000230349 | 0.003090386 |
| GO:0022008 | neurogenesis | 0.000237612 | 0.003177168 |
| GO:0009719 | response to endogenous stimulus | 0.000240453 | 0.003204437 |
| GO:0043632 | modification-dependent macromolecule catabolic process | 0.000252401 | 0.00335113 |
| GO:0016337 | cell-cell adhesion | 0.000253137 | 0.00335113 |
| GO:0042692 | muscle cell differentiation | 0.000256685 | 0.003386893 |
| GO:0032989 | cellular component morphogenesis | 0.000259469 | 0.00340626 |
| GO:0007507 | heart development | 0.000259857 | 0.00340626 |
| GO:0006633 | fatty acid biosynthetic process | 0.000262865 | 0.003434432 |
| GO:0040011 | locomotion | 0.000267596 | 0.003484852 |
| GO:0009725 | response to hormone stimulus | 0.00027099 | 0.003517596 |
| GO:0031344 | regulation of cell projection organization | 0.000272644 | 0.003527608 |
| GO:0045664 | regulation of neuron differentiation | 0.00027694 | 0.003571635 |
| GO:0051170 | nuclear import | 0.000286919 | 0.003688426 |
| GO:0016055 | Wnt receptor signaling pathway | 0.000289177 | 0.003702051 |
| GO:0006518 | peptide metabolic process | 0.00028983 | 0.003702051 |
| GO:0022604 | regulation of cell morphogenesis | 0.000298402 | 0.003799396 |
| GO:0002520 | immune system development | 0.00029949 | 0.003800904 |
| GO:0030879 | mammary gland development | 0.000300422 | 0.003800904 |
| GO:0032844 | regulation of homeostatic process | 0.000302727 | 0.003817987 |
| GO:0010975 | regulation of neuron projection development | 0.000304047 | 0.003822573 |
| GO:0043433 | negative regulation of sequence-specific DNA binding transcription factor activity | 0.000308293 | 0.003863806 |
| GO:0006606 | protein import into nucleus | 0.000321314 | 0.004014417 |
| GO:0031175 | neuron projection development | 0.000325271 | 0.004051198 |
| GO:0000902 | cell morphogenesis | 0.000327085 | 0.004061142 |
| GO:2000145 | regulation of cell motility | 0.000340578 | 0.00421558 |
| GO:0009790 | embryo development | 0.000344087 | 0.00424586 |
| GO:0006917 | induction of apoptosis | 0.000363084 | 0.004466495 |
| GO:0030032 | lamellipodium assembly | 0.000374165 | 0.004587181 |
| GO:0051129 | negative regulation of cellular component organization | 0.00037519 | 0.004587181 |
| GO:0034440 | lipid oxidation | 0.00039864 | 0.004859035 |
| GO:0016044 | cellular membrane organization | 0.000405612 | 0.004922067 |
| GO:0008203 | cholesterol metabolic process | 0.000407266 | 0.004922067 |
| GO:0030010 | establishment of cell polarity | 0.000407505 | 0.004922067 |
| GO:0009792 | embryo development ending in birth or egg hatching | 0.000411698 | 0.004957739 |
| GO:0007163 | establishment or maintenance of cell polarity | 0.000438237 | 0.005259678 |
| GO:0051960 | regulation of nervous system development | 0.000439403 | 0.005259678 |
| GO:0048534 | hemopoietic or lymphoid organ development | 0.000444156 | 0.005300709 |
| GO:0060429 | epithelium development | 0.00044823 | 0.005333409 |
| GO:0051494 | negative regulation of cytoskeleton organization | 0.000453556 | 0.005380763 |
| GO:0045444 | fat cell differentiation | 0.00047386 | 0.005605012 |
| GO:0044257 | cellular protein catabolic process | 0.000479254 | 0.005652082 |
| GO:0043254 | regulation of protein complex assembly | 0.000489305 | 0.005753645 |
| GO:0016051 | carbohydrate biosynthetic process | 0.000494592 | 0.005798762 |
| GO:0009628 | response to abiotic stimulus | 0.000497698 | 0.005818114 |
| GO:0051603 | proteolysis involved in cellular protein catabolic process | 0.000499445 | 0.005821517 |
| GO:0043434 | response to peptide hormone stimulus | 0.000520439 | 0.006048587 |
| GO:0048585 | negative regulation of response to stimulus | 0.00054045 | 0.006262959 |
| GO:0007266 | Rho protein signal transduction | 0.000545116 | 0.006298771 |
| GO:0032321 | positive regulation of Rho GTPase activity | 0.000560677 | 0.006459904 |
| GO:0046822 | regulation of nucleocytoplasmic transport | 0.0005656 | 0.006497903 |
| GO:0051272 | positive regulation of cellular component movement | 0.00056926 | 0.006521207 |
| GO:0012502 | induction of programmed cell death | 0.000582271 | 0.006651201 |
| GO:0061061 | muscle structure development | 0.000586127 | 0.006676168 |
| GO:0010035 | response to inorganic substance | 0.000587799 | 0.006676196 |
| GO:0043161 | proteasomal ubiquitin-dependent protein catabolic process | 0.000599192 | 0.006786315 |
| GO:0045597 | positive regulation of cell differentiation | 0.000613955 | 0.006933881 |
| GO:0001944 | vasculature development | 0.000623822 | 0.007025463 |
| GO:0051345 | positive regulation of hydrolase activity | 0.000633563 | 0.007115122 |
| GO:0051091 | positive regulation of sequence-specific DNA binding transcription factor activity | 0.000635592 | 0.007117915 |
| GO:0007623 | circadian rhythm | 0.000640348 | 0.007151151 |
| GO:0048732 | gland development | 0.000645636 | 0.007190122 |
| GO:0032271 | regulation of protein polymerization | 0.000690597 | 0.007669462 |
| GO:0051050 | positive regulation of transport | 0.000696984 | 0.007718954 |
| GO:0051224 | negative regulation of protein transport | 0.000712345 | 0.007867285 |
| GO:0006520 | cellular amino acid metabolic process | 0.000725706 | 0.007992762 |
| GO:0032886 | regulation of microtubule-based process | 0.000733937 | 0.008061204 |
| GO:0070507 | regulation of microtubule cytoskeleton organization | 0.000751691 | 0.008220504 |
| GO:0006749 | glutathione metabolic process | 0.000752552 | 0.008220504 |
| GO:0008654 | phospholipid biosynthetic process | 0.000772134 | 0.008411425 |
| GO:0030163 | protein catabolic process | 0.000811127 | 0.008812187 |
| GO:0030182 | neuron differentiation | 0.000845777 | 0.009163727 |
| GO:0045017 | glycerolipid biosynthetic process | 0.000851429 | 0.009200032 |
| GO:0016197 | endosomal transport | 0.000856387 | 0.009228662 |
| GO:0046777 | protein autophosphorylation | 0.000860736 | 0.009250595 |
| GO:0010498 | proteasomal protein catabolic process | 0.000869864 | 0.009323639 |
| GO:0051260 | protein homooligomerization | 0.000907937 | 0.009705705 |
| GO:0070925 | organelle assembly | 0.000912628 | 0.009729829 |
| GO:0000165 | MAPK cascade | 0.000918906 | 0.009770713 |
| GO:0032320 | positive regulation of Ras GTPase activity | 0.000942839 | 0.009998591 |
| GO:0001568 | blood vessel development | 0.000950853 | 0.010056902 |
| GO:0044275 | cellular carbohydrate catabolic process | 0.000973002 | 0.010264013 |
| GO:0009611 | response to wounding | 0.001001077 | 0.010521649 |
| GO:0043393 | regulation of protein binding | 0.001002688 | 0.010521649 |
| GO:0006914 | autophagy | 0.00103459 | 0.010827987 |
| GO:2000147 | positive regulation of cell motility | 0.001066354 | 0.011131292 |
| GO:0051092 | positive regulation of NF-kappaB transcription factor activity | 0.001086569 | 0.011312773 |
| GO:0034329 | cell junction assembly | 0.001111245 | 0.011539632 |
| GO:0048646 | anatomical structure formation involved in morphogenesis | 0.001131488 | 0.011719398 |
| GO:0051130 | positive regulation of cellular component organization | 0.001179241 | 0.012156579 |
| GO:0050994 | regulation of lipid catabolic process | 0.001179778 | 0.012156579 |
| GO:0016054 | organic acid catabolic process | 0.001188995 | 0.012220061 |
| GO:0006458 | 'de novo' protein folding | 0.001198796 | 0.012280207 |
| GO:0030099 | myeloid cell differentiation | 0.001200991 | 0.012280207 |
| GO:0061077 | chaperone-mediated protein folding | 0.001226064 | 0.012504597 |
| GO:0048699 | generation of neurons | 0.00125657 | 0.012783118 |
| GO:0002274 | myeloid leukocyte activation | 0.001306077 | 0.013253032 |
| GO:0010740 | positive regulation of intracellular protein kinase cascade | 0.001342365 | 0.013586769 |
| GO:0045786 | negative regulation of cell cycle | 0.00135101 | 0.013611991 |
| GO:0007249 | I-kappaB kinase/NF-kappaB cascade | 0.001353168 | 0.013611991 |
| GO:0006469 | negative regulation of protein kinase activity | 0.001355071 | 0.013611991 |
| GO:0035264 | multicellular organism growth | 0.001398008 | 0.014008112 |
| GO:0032269 | negative regulation of cellular protein metabolic process | 0.001417492 | 0.01416783 |
| GO:0050727 | regulation of inflammatory response | 0.001455691 | 0.014513349 |
| GO:0097285 | cell-type specific apoptotic process | 0.001465483 | 0.014574631 |
| GO:0030335 | positive regulation of cell migration | 0.00147236 | 0.014606685 |
| GO:0002275 | myeloid cell activation involved in immune response | 0.001545641 | 0.015295727 |
| GO:0031110 | regulation of microtubule polymerization or depolymerization | 0.00156577 | 0.015456661 |
| GO:0032319 | regulation of Rho GTPase activity | 0.001594362 | 0.015676014 |
| GO:0051240 | positive regulation of multicellular organismal process | 0.001595832 | 0.015676014 |
| GO:0042991 | transcription factor import into nucleus | 0.001630122 | 0.015973599 |
| GO:0007160 | cell-matrix adhesion | 0.001635993 | 0.015991936 |
| GO:0002376 | immune system process | 0.00167048 | 0.016289213 |
| GO:0055114 | oxidation-reduction process | 0.001754529 | 0.017025749 |
| GO:0072594 | establishment of protein localization to organelle | 0.00183959 | 0.01780794 |
| GO:0019725 | cellular homeostasis | 0.001873366 | 0.018091103 |
| GO:0009108 | coenzyme biosynthetic process | 0.001902077 | 0.018302702 |
| GO:0044087 | regulation of cellular component biogenesis | 0.001904919 | 0.018302702 |
| GO:0090181 | regulation of cholesterol metabolic process | 0.00190969 | 0.018302702 |
| GO:0006417 | regulation of translation | 0.001913589 | 0.018302702 |
| GO:0009057 | macromolecule catabolic process | 0.001921298 | 0.018332572 |
| GO:0045216 | cell-cell junction organization | 0.001942918 | 0.018494732 |
| GO:0043470 | regulation of carbohydrate catabolic process | 0.001995941 | 0.018954329 |
| GO:0008202 | steroid metabolic process | 0.002026533 | 0.019170407 |
| GO:0006916 | anti-apoptosis | 0.002028285 | 0.019170407 |
| GO:0006650 | glycerophospholipid metabolic process | 0.002065265 | 0.019473895 |
| GO:0051402 | neuron apoptotic process | 0.00209578 | 0.019715125 |
| GO:0032273 | positive regulation of protein polymerization | 0.002104024 | 0.019726555 |
| GO:0033598 | mammary gland epithelial cell proliferation | 0.002106863 | 0.019726555 |
| GO:0009755 | hormone-mediated signaling pathway | 0.002162807 | 0.020203041 |
| GO:0035239 | tube morphogenesis | 0.002223794 | 0.020724311 |
| GO:0000271 | polysaccharide biosynthetic process | 0.002278546 | 0.021185174 |
| GO:0002263 | cell activation involved in immune response | 0.002313852 | 0.021463527 |
| GO:0008154 | actin polymerization or depolymerization | 0.00231996 | 0.021470366 |
| GO:0035335 | peptidyl-tyrosine dephosphorylation | 0.002386519 | 0.021986611 |
| GO:0032388 | positive regulation of intracellular transport | 0.002386741 | 0.021986611 |
| GO:0060443 | mammary gland morphogenesis | 0.00239333 | 0.02199663 |
| GO:0006140 | regulation of nucleotide metabolic process | 0.002423568 | 0.022172925 |
| GO:0032387 | negative regulation of intracellular transport | 0.002423604 | 0.022172925 |
| GO:0070482 | response to oxygen levels | 0.002445276 | 0.022320126 |
| GO:0043523 | regulation of neuron apoptotic process | 0.002571364 | 0.023417567 |
| GO:0040012 | regulation of locomotion | 0.002619466 | 0.023801425 |
| GO:1901137 | carbohydrate derivative biosynthetic process | 0.002649465 | 0.023965071 |
| GO:0031589 | cell-substrate adhesion | 0.002668162 | 0.02407971 |
| GO:0044265 | cellular macromolecule catabolic process | 0.002732724 | 0.02460683 |
| GO:0006611 | protein export from nucleus | 0.002755339 | 0.02475471 |
| GO:0007030 | Golgi organization | 0.002784425 | 0.024959931 |
| GO:0010883 | regulation of lipid storage | 0.002798627 | 0.025031117 |
| GO:0043405 | regulation of MAP kinase activity | 0.002814371 | 0.025115753 |
| GO:0031334 | positive regulation of protein complex assembly | 0.002835277 | 0.025245966 |
| GO:0051640 | organelle localization | 0.00284344 | 0.025262388 |
| GO:0042990 | regulation of transcription factor import into nucleus | 0.0028751 | 0.025487024 |
| GO:0051656 | establishment of organelle localization | 0.002905837 | 0.025702514 |
| path:hsa04910 | Insulin signaling pathway | 0.000551561 | 0.025785455 |
| GO:0046847 | filopodium assembly | 0.002962443 | 0.026145355 |
| GO:0060444 | branching involved in mammary gland duct morphogenesis | 0.002984873 | 0.026285288 |
| GO:0051094 | positive regulation of developmental process | 0.003026867 | 0.026596516 |
| GO:0043408 | regulation of MAPK cascade | 0.00304604 | 0.026706292 |
| GO:0006839 | mitochondrial transport | 0.003108272 | 0.027192282 |
| GO:0060603 | mammary gland duct morphogenesis | 0.003175502 | 0.02765044 |
| GO:0030323 | respiratory tube development | 0.00317631 | 0.02765044 |
| GO:0070997 | neuron death | 0.003185259 | 0.02765044 |
| GO:0001775 | cell activation | 0.003188307 | 0.02765044 |
| GO:0031098 | stress-activated protein kinase signaling cascade | 0.003248764 | 0.028113766 |
| GO:0045926 | negative regulation of growth | 0.003336975 | 0.028804293 |
| GO:0031109 | microtubule polymerization or depolymerization | 0.003342969 | 0.028804293 |
| GO:0018193 | peptidyl-amino acid modification | 0.003423155 | 0.029431773 |
| GO:0007155 | cell adhesion | 0.003453556 | 0.029629437 |
| GO:0071902 | positive regulation of protein serine/threonine kinase activity | 0.003616358 | 0.030936663 |
| GO:0071496 | cellular response to external stimulus | 0.0036214 | 0.030936663 |
| GO:0015980 | energy derivation by oxidation of organic compounds | 0.00363939 | 0.031024052 |
| GO:0051051 | negative regulation of transport | 0.003652667 | 0.031070982 |
| GO:0007618 | mating | 0.003684304 | 0.031212515 |
| GO:0010906 | regulation of glucose metabolic process | 0.003684919 | 0.031212515 |
| GO:0006084 | acetyl-CoA metabolic process | 0.003836995 | 0.032295384 |
| GO:0034599 | cellular response to oxidative stress | 0.003855073 | 0.032379373 |
| GO:0015988 | energy coupled proton transport, against electrochemical gradient | 0.003978096 | 0.033342618 |
| GO:0010563 | negative regulation of phosphorus metabolic process | 0.003991566 | 0.033385529 |
| GO:0016049 | cell growth | 0.004051098 | 0.033812717 |
| GO:0014015 | positive regulation of gliogenesis | 0.004124298 | 0.034280549 |
| GO:0043467 | regulation of generation of precursor metabolites and energy | 0.004147649 | 0.03440311 |
| GO:0048514 | blood vessel morphogenesis | 0.004170701 | 0.034522699 |
| GO:0006790 | sulfur compound metabolic process | 0.00418629 | 0.034580135 |
| GO:0018105 | peptidyl-serine phosphorylation | 0.004252013 | 0.035050612 |
| GO:0040017 | positive regulation of locomotion | 0.004272737 | 0.035148978 |
| GO:0042306 | regulation of protein import into nucleus | 0.00429485 | 0.03525834 |
| GO:0008286 | insulin receptor signaling pathway | 0.004348532 | 0.035625887 |
| GO:0007033 | vacuole organization | 0.004373826 | 0.035676243 |
| GO:0051085 | chaperone mediated protein folding requiring cofactor | 0.00438145 | 0.035676243 |
| GO:0030832 | regulation of actin filament length | 0.004395851 | 0.035720759 |
| GO:0030324 | lung development | 0.004413338 | 0.035790111 |
| GO:0030278 | regulation of ossification | 0.004434674 | 0.035890339 |
| GO:0022610 | biological adhesion | 0.00444786 | 0.035924332 |
| GO:0007006 | mitochondrial membrane organization | 0.004582615 | 0.036938096 |
| GO:0010565 | regulation of cellular ketone metabolic process | 0.004656429 | 0.03745755 |
| GO:0030048 | actin filament-based movement | 0.004735533 | 0.038017388 |
| GO:0001558 | regulation of cell growth | 0.004746909 | 0.038032349 |
| GO:0044282 | small molecule catabolic process | 0.004875904 | 0.038909908 |
| GO:0006639 | acylglycerol metabolic process | 0.005121455 | 0.040788003 |
| GO:0016050 | vesicle organization | 0.005203102 | 0.041355867 |
| GO:0060537 | muscle tissue development | 0.005271916 | 0.04181968 |
| GO:0030041 | actin filament polymerization | 0.005291364 | 0.041890838 |
| GO:0008064 | regulation of actin polymerization or depolymerization | 0.005361212 | 0.04235993 |
| GO:0051146 | striated muscle cell differentiation | 0.005466285 | 0.043099083 |
| GO:0036293 | response to decreased oxygen levels | 0.005476322 | 0.043099083 |
| GO:0022407 | regulation of cell-cell adhesion | 0.005499145 | 0.043193678 |
| GO:1900542 | regulation of purine nucleotide metabolic process | 0.005546376 | 0.04347924 |
| GO:2001236 | regulation of extrinsic apoptotic signaling pathway | 0.005614604 | 0.043792829 |
| GO:0048666 | neuron development | 0.00563128 | 0.043792829 |
| GO:0022402 | cell cycle process | 0.005633382 | 0.043792829 |
| GO:0006638 | neutral lipid metabolic process | 0.005647749 | 0.043792829 |
| GO:0031345 | negative regulation of cell projection organization | 0.005651606 | 0.043792829 |
| GO:0040008 | regulation of growth | 0.005695858 | 0.044046503 |
| GO:0007032 | endosome organization | 0.005720765 | 0.044107482 |
| GO:0042326 | negative regulation of phosphorylation | 0.005725809 | 0.044107482 |
| GO:0032885 | regulation of polysaccharide biosynthetic process | 0.005789301 | 0.044510821 |
| GO:0035966 | response to topologically incorrect protein | 0.00584454 | 0.044849272 |
| GO:0002224 | toll-like receptor signaling pathway | 0.005869489 | 0.044954438 |
| GO:0007052 | mitotic spindle organization | 0.005952173 | 0.045400364 |
| GO:0090316 | positive regulation of intracellular protein transport | 0.005971829 | 0.045400364 |
| GO:0060541 | respiratory system development | 0.005991971 | 0.045457115 |
| GO:0031331 | positive regulation of cellular catabolic process | 0.006022038 | 0.045598692 |
| GO:0044264 | cellular polysaccharide metabolic process | 0.006048736 | 0.045714268 |
| GO:0006612 | protein targeting to membrane | 0.006181956 | 0.046495514 |
| GO:0000209 | protein polyubiquitination | 0.006182825 | 0.046495514 |
| GO:0034614 | cellular response to reactive oxygen species | 0.006186997 | 0.046495514 |
| GO:0034637 | cellular carbohydrate biosynthetic process | 0.006234184 | 0.046710604 |
| GO:0030111 | regulation of Wnt receptor signaling pathway | 0.006238985 | 0.046710604 |
| GO:0006893 | Golgi to plasma membrane transport | 0.006334084 | 0.047333955 |
| GO:0006641 | triglyceride metabolic process | 0.006403883 | 0.047766275 |
| GO:0007041 | lysosomal transport | 0.006477562 | 0.048225872 |
| GO:0006979 | response to oxidative stress | 0.006555476 | 0.048715232 |
| GO:0007009 | plasma membrane organization | 0.006653432 | 0.049351431 |
| GO:0006109 | regulation of carbohydrate metabolic process | 0.006694032 | 0.049560631 |
| GO:0051099 | positive regulation of binding | 0.006738017 | 0.049794073 |

**Table S2: Gene Ontology terms enriched with PPFP peaks that contain only a PPARG motif, or only a PAX8 motif**

| **Geneset.ID** | **Description** | **PPARG_only *q*-value** | **PAX8_only *q*-value** | **significant in** |
| --- | --- | --- | --- | --- |
| GO:0008202 | steroid metabolic process | 3.31E-05 | 0.542082 | pparg_only |
| GO:0042692 | muscle cell differentiation | 9.76E-05 | 0.565956 | pparg_only |
| GO:0008203 | cholesterol metabolic process | 0.00021642 | 0.0608696 | pparg_only |
| GO:0051146 | striated muscle cell differentiation | 0.00035741 | 0.6678711 | pparg_only |
| GO:0061061 | muscle structure development | 0.00048947 | 0.6989164 | pparg_only |
| GO:0044242 | cellular lipid catabolic process | 0.00079542 | 0.3881836 | pparg_only |
| GO:0040007 | growth | 0.00157593 | 0.1871177 | pparg_only |
| GO:0000038 | very long-chain fatty acid metabolic process | 0.00247001 | 0.8970874 | pparg_only |
| GO:0060537 | muscle tissue development | 0.00285495 | 0.3890696 | pparg_only |
| GO:0046777 | protein autophosphorylation | 0.00372413 | 0.7061986 | pparg_only |
| GO:0016055 | Wnt receptor signaling pathway | 0.00401528 | 0.6344414 | pparg_only |
| GO:0014706 | striated muscle tissue development | 0.0047319 | 0.4518658 | pparg_only |
| GO:0002009 | morphogenesis of an epithelium | 0.00497002 | 0.0505264 | pparg_only |
| GO:0006913 | nucleocytoplasmic transport | 0.00497002 | 0.067707 | pparg_only |
| GO:0016042 | lipid catabolic process | 0.00613473 | 0.4476966 | pparg_only |
| GO:0055001 | muscle cell development | 0.00698634 | 0.3573717 | pparg_only |
| GO:0051169 | nuclear transport | 0.00751728 | 0.0870296 | pparg_only |
| GO:0001501 | skeletal system development | 0.00789359 | 0.0806103 | pparg_only |
| GO:0051098 | regulation of binding | 0.00800482 | 0.0649634 | pparg_only |
| GO:0006006 | glucose metabolic process | 0.00868627 | 0.1961814 | pparg_only |
| GO:0030048 | actin filament-based movement | 0.00937256 | 0.2752301 | pparg_only |
| GO:0006417 | regulation of translation | 0.00955105 | 0.3030887 | pparg_only |
| GO:0010565 | regulation of cellular ketone metabolic process | 0.00976783 | 0.0988056 | pparg_only |
| GO:0060048 | cardiac muscle contraction | 0.01011683 | 0.5811476 | pparg_only |
| GO:0051301 | cell division | 0.01111466 | 0.8920545 | pparg_only |
| GO:0010883 | regulation of lipid storage | 0.01222354 | 0.5735777 | pparg_only |
| GO:0000904 | cell morphogenesis involved in differentiation | 0.0133609 | 0.2331529 | pparg_only |
| GO:0048870 | cell motility | 0.01480655 | 0.08287 | pparg_only |
| GO:0002028 | regulation of sodium ion transport | 0.01536284 | 0.6909566 | pparg_only |
| GO:0051130 | positive regulation of cellular component organization | 0.01546001 | 0.3816011 | pparg_only |
| GO:0002521 | leukocyte differentiation | 0.01648448 | 0.7510752 | pparg_only |
| GO:0030111 | regulation of Wnt receptor signaling pathway | 0.01652971 | 0.5521766 | pparg_only |
| GO:0051531 | NFAT protein import into nucleus | 0.01744251 | 0.4476966 | pparg_only |
| GO:0048008 | platelet-derived growth factor receptor signaling pathway | 0.01771845 | 0.0547403 | pparg_only |
| GO:0044087 | regulation of cellular component biogenesis | 0.01956167 | 0.1041852 | pparg_only |
| GO:0071702 | organic substance transport | 0.01984443 | 0.1848338 | pparg_only |
| GO:0055002 | striated muscle cell development | 0.01984443 | 0.4094058 | pparg_only |
| GO:0035264 | multicellular organism growth | 0.02017168 | 0.5925668 | pparg_only |
| GO:0043550 | regulation of lipid kinase activity | 0.02203623 | 0.8264784 | pparg_only |
| GO:0010608 | posttranscriptional regulation of gene expression | 0.02260304 | 0.2185605 | pparg_only |
| GO:0007041 | lysosomal transport | 0.02286045 | 0.1749339 | pparg_only |
| GO:0010675 | regulation of cellular carbohydrate metabolic process | 0.02309736 | 0.864147 | pparg_only |
| GO:0010453 | regulation of cell fate commitment | 0.0257427 | 0.8450373 | pparg_only |
| GO:0006941 | striated muscle contraction | 0.02607999 | 0.893685 | pparg_only |
| GO:0032369 | negative regulation of lipid transport | 0.02871242 | 0.4454749 | pparg_only |
| GO:0007034 | vacuolar transport | 0.02887679 | 0.2958327 | pparg_only |
| GO:0051155 | positive regulation of striated muscle cell differentiation | 0.02935288 | 0.9066576 | pparg_only |
| GO:0006625 | protein targeting to peroxisome | 0.02964099 | 0.8994604 | pparg_only |
| GO:0040012 | regulation of locomotion | 0.03009814 | 0.1213177 | pparg_only |
| GO:0010676 | positive regulation of cellular carbohydrate metabolic process | 0.03102129 | 0.9852286 | pparg_only |
| GO:0001952 | regulation of cell-matrix adhesion | 0.03167917 | 0.0507065 | pparg_only |
| GO:0007589 | body fluid secretion | 0.03179067 | 0.371974 | pparg_only |
| GO:0040017 | positive regulation of locomotion | 0.03229316 | 0.1189013 | pparg_only |
| GO:0006109 | regulation of carbohydrate metabolic process | 0.03292272 | 0.8715827 | pparg_only |
| GO:0048589 | developmental growth | 0.03399511 | 0.5386921 | pparg_only |
| GO:0090068 | positive regulation of cell cycle process | 0.03461512 | 0.8535055 | pparg_only |
| GO:0030512 | negative regulation of transforming growth factor beta receptor signaling pathway | 0.03465802 | 0.6330843 | pparg_only |
| GO:0019217 | regulation of fatty acid metabolic process | 0.03493459 | 0.3283814 | pparg_only |
| GO:0018108 | peptidyl-tyrosine phosphorylation | 0.03765655 | 0.2685921 | pparg_only |
| GO:0031958 | corticosteroid receptor signaling pathway | 0.03856129 | 0.776205 | pparg_only |
| GO:0045913 | positive regulation of carbohydrate metabolic process | 0.03907648 | 0.9647873 | pparg_only |
| GO:0061008 | hepaticobiliary system development | 0.04003926 | 0.0976316 | pparg_only |
| GO:0055007 | cardiac muscle cell differentiation | 0.04138721 | 0.9940225 | pparg_only |
| GO:0045540 | regulation of cholesterol biosynthetic process | 0.04171181 | 0.2276207 | pparg_only |
| GO:0001889 | liver development | 0.04220237 | 0.0610115 | pparg_only |
| GO:0048812 | neuron projection morphogenesis | 0.04415828 | 0.4340047 | pparg_only |
| GO:0045596 | negative regulation of cell differentiation | 0.04658516 | 0.1110088 | pparg_only |
| GO:0001775 | cell activation | 0.04686698 | 0.192712 | pparg_only |
| GO:0034453 | microtubule anchoring | 0.0473635 | 0.4196972 | pparg_only |
| GO:0000165 | MAPK cascade | 0.0475635 | 0.1149733 | pparg_only |
| GO:0060070 | canonical Wnt receptor signaling pathway | 0.04955572 | 0.9097459 | pparg_only |
| GO:0007044 | cell-substrate junction assembly | 0.13051603 | 1.24E-05 | pax8_only |
| GO:0048041 | focal adhesion assembly | 0.12855719 | 3.33E-05 | pax8_only |
| GO:0034329 | cell junction assembly | 0.10649147 | 5.52E-05 | pax8_only |
| GO:0070482 | response to oxygen levels | 0.44924059 | 5.79E-05 | pax8_only |
| GO:0006916 | anti-apoptosis | 0.55857857 | 8.19E-05 | pax8_only |
| GO:0001666 | response to hypoxia | 0.44924059 | 0.0001483 | pax8_only |
| GO:0036293 | response to decreased oxygen levels | 0.37509276 | 0.0001757 | pax8_only |
| GO:0031589 | cell-substrate adhesion | 0.18916845 | 0.0009271 | pax8_only |
| GO:0051258 | protein polymerization | 0.06098779 | 0.0017952 | pax8_only |
| GO:0034621 | cellular macromolecular complex subunit organization | 0.18210323 | 0.0034468 | pax8_only |
| GO:0033043 | regulation of organelle organization | 0.17875095 | 0.0035041 | pax8_only |
| GO:0032271 | regulation of protein polymerization | 0.23849885 | 0.0039941 | pax8_only |
| GO:0034622 | cellular macromolecular complex assembly | 0.11481453 | 0.0046948 | pax8_only |
| GO:0032869 | cellular response to insulin stimulus | 0.20139117 | 0.0058399 | pax8_only |
| GO:0035148 | tube formation | 0.22177605 | 0.0075329 | pax8_only |
| GO:0030833 | regulation of actin filament polymerization | 0.30701965 | 0.0082396 | pax8_only |
| GO:0030838 | positive regulation of actin filament polymerization | 0.186398 | 0.0083234 | pax8_only |
| GO:0032273 | positive regulation of protein polymerization | 0.11235215 | 0.0083794 | pax8_only |
| GO:0043001 | Golgi to plasma membrane protein transport | 0.07719531 | 0.0102028 | pax8_only |
| GO:0030041 | actin filament polymerization | 0.16077706 | 0.0102028 | pax8_only |
| GO:0051493 | regulation of cytoskeleton organization | 0.25252996 | 0.0109886 | pax8_only |
| GO:0009100 | glycoprotein metabolic process | 0.40609763 | 0.016382 | pax8_only |
| GO:0001838 | embryonic epithelial tube formation | 0.34202305 | 0.0183983 | pax8_only |
| GO:0030832 | regulation of actin filament length | 0.35176694 | 0.0194113 | pax8_only |
| GO:0043112 | receptor metabolic process | 0.13576071 | 0.0200712 | pax8_only |
| GO:0008154 | actin polymerization or depolymerization | 0.12161555 | 0.0201135 | pax8_only |
| GO:0045860 | positive regulation of protein kinase activity | 0.71664127 | 0.0206966 | pax8_only |
| GO:0032321 | positive regulation of Rho GTPase activity | 0.07964898 | 0.024476 | pax8_only |
| GO:0009395 | phospholipid catabolic process | 0.94281407 | 0.0264931 | pax8_only |
| GO:0032320 | positive regulation of Ras GTPase activity | 0.12963397 | 0.0275555 | pax8_only |
| GO:0008064 | regulation of actin polymerization or depolymerization | 0.30327826 | 0.0292807 | pax8_only |
| GO:0030857 | negative regulation of epithelial cell differentiation | 0.96962391 | 0.0303402 | pax8_only |
| GO:0048598 | embryonic morphogenesis | 0.20281592 | 0.0308619 | pax8_only |
| GO:0051495 | positive regulation of cytoskeleton organization | 0.46382516 | 0.0331682 | pax8_only |
| GO:0007032 | endosome organization | 0.90983353 | 0.0332776 | pax8_only |
| GO:0019725 | cellular homeostasis | 0.25252996 | 0.0368145 | pax8_only |
| GO:0048872 | homeostasis of number of cells | 0.0892293 | 0.0369479 | pax8_only |
| GO:0043124 | negative regulation of I-kappaB kinase/NF-kappaB cascade | 0.6818015 | 0.0375818 | pax8_only |
| GO:0033674 | positive regulation of kinase activity | 0.61832171 | 0.0379731 | pax8_only |
| GO:0050729 | positive regulation of inflammatory response | 0.95060026 | 0.0391854 | pax8_only |
| GO:0016331 | morphogenesis of embryonic epithelium | 0.18916845 | 0.0399889 | pax8_only |
| GO:0051893 | regulation of focal adhesion assembly | 0.11377398 | 0.0458086 | pax8_only |
| GO:0070201 | regulation of establishment of protein localization | 0.1651806 | 0.0471184 | pax8_only |
| GO:0072657 | protein localization to membrane | 0.30520416 | 0.0471184 | pax8_only |
| GO:0051129 | negative regulation of cellular component organization | 0.68983595 | 0.0492265 | pax8_only |
| GO:0010638 | positive regulation of organelle organization | 0.12949583 | 0.0497023 | pax8_only |

**Table S3: Gene Ontology terms enriched in genes that contain PPFP peaks <10 kb from a TSS, or 10-100 kb from a TSS, or both.** GO terms are grouped based on key words within the gene set names.

| **Word in GO term** | **GO terms only enriched in peaks <10kb from TSS** | **GO terms only enriched in peaks 10-100kb from TSS** | **GO terms enriched in both** |
| --- | --- | --- | --- |
| cell cycle, mitotic | GO:0007049 cell cycle  GO:0051726 regulation of cell cycle  GO:0045786 negative regulation of cell cycle  GO:0010564 regulation of cell cycle process  GO:0090068 positive regulation of cell cycle process  GO:0007050 cell cycle arrest  GO:0031571 mitotic cell cycle G1/S transition DNA damage checkpoint  GO:0007052 mitotic spindle organization |  |  |
| protein folding | GO:0006457 protein folding  GO:0006458 'de novo' protein folding  GO:0061077 chaperone-mediated protein folding  GO:0051085 chaperone mediated protein folding requiring cofactor |  |  |
| Microtubule, cytoskeleton | GO:0000226 microtubule cytoskeleton organization  GO:0032886 regulation of microtubule-based process  GO:0070507 regulation of microtubule cytoskeleton organization  GO:0031109 microtubule polymerization or depolymerization  GO:0007017 microtubule-based process  GO:0031110 regulation of microtubule polymerization or depolymerization  GO:0031111 negative regulation of microtubule polymerization or depolymerization  GO:0007019 microtubule depolymerization  GO:0031114 regulation of microtubule depolymerization  GO:0051495 positive regulation of cytoskeleton organization  GO:0051494 negative regulation of cytoskeleton organization |  | GO:0007010 cytoskeleton organization  GO:0051493 regulation of cytoskeleton organization  GO:0030036 actin cytoskeleton organization |
| Locomotion, migration, motility |  | GO:0040017 positive regulation of locomotion  GO:0040012 regulation of locomotion  GO:0040011 locomotion  GO:0030335 positive regulation of cell migration  GO:0030334 regulation of cell migration  GO:0016477 cell migration  GO:2000147 positive regulation of cell motility  GO:2000145 regulation of cell motility  GO:0048870 cell motility |  |
| Wnt |  | GO:0060070 canonical Wnt receptor signaling pathway  GO:0060828 regulation of canonical Wnt receptor signaling pathway  GO:0016055 Wnt receptor signaling pathway  GO:0030111 regulation of Wnt receptor signaling pathway  GO:0030178 negative regulation of Wnt receptor signaling pathway |  |
| Morphogenesis, development | GO:0001890 placenta development  GO:0001892 embryonic placenta development  GO:0055001 muscle cell development  GO:0055002 striated muscle cell development  GO:0060711 labyrinthine layer development  GO:0060603 mammary gland duct morphogenesis | GO:0009653 anatomical structure morphogenesis  GO:0022603 regulation of anatomical structure morphogenesis  GO:0002009 morphogenesis of an epithelium  GO:0000902 cell morphogenesis  GO:0022604 regulation of cell morphogenesis  GO:0032989 cellular component morphogenesis  GO:0035239 tube morphogenesis  GO:0048729 tissue morphogenesis  GO:0048646 anatomical structure formation involved in morphogenesis  GO:0048514 blood vessel morphogenesis  GO:0001763 morphogenesis of a branching structure  GO:0000904 cell morphogenesis involved in differentiation  GO:0060562 epithelial tube morphogenesis  GO:0061138 morphogenesis of a branching epithelium  GO:0048754 branching morphogenesis of a tube  GO:0048598 embryonic morphogenesis  GO:0010769 regulation of cell morphogenesis involved in differentiation  GO:0022612 gland morphogenesis  GO:0048705 skeletal system morphogenesis  GO:2000027 regulation of organ morphogenesis  GO:0032990 cell part morphogenesis  GO:0060429 epithelium development  GO:0009790 embryo development  GO:0035295 tube development  GO:2000026 regulation of multicellular organismal development  GO:0060284 regulation of cell development  GO:0002520 immune system development  GO:0072358 cardiovascular system development  GO:0048534 hemopoietic or lymphoid organ development  GO:0007399 nervous system development  GO:0001944 vasculature development  GO:0048468 cell development  GO:0001568 blood vessel development  GO:0031175 neuron projection development  GO:0048732 gland development  GO:0001501 skeletal system development  GO:0007507 heart development  GO:0010975 regulation of neuron projection development  GO:0051960 regulation of nervous system development  GO:0051094 positive regulation of developmental process  GO:0030324 lung development  GO:0030323 respiratory tube development  GO:0060541 respiratory system development  GO:0007398 ectoderm development  GO:0048589 developmental growth  GO:0048608 reproductive structure development  GO:0048666 neuron development  GO:0010976 positive regulation of neuron projection development  GO:0003281 ventricular septum development | GO:0060444 branching involved in mammary gland duct morphogenesis  GO:0060443 mammary gland morphogenesis  GO:0001701 in utero embryonic development  GO:0061180 mammary gland epithelium development  GO:0050793 regulation of developmental process  GO:0030879 mammary gland development  GO:0009888 tissue development  GO:0009792 embryo development ending in birth or egg hatching  GO:0043009 chordate embryonic development |
| Metabolic, catabolic | GO:0051186 cofactor metabolic process  GO:0010565 regulation of cellular ketone metabolic process  GO:0051248 negative regulation of protein metabolic process  GO:0019217 regulation of fatty acid metabolic process  GO:0006637 acyl-CoA metabolic process  GO:0032269 negative regulation of cellular protein metabolic process  GO:0000038 very long-chain fatty acid metabolic process  GO:0010563 negative regulation of phosphorus metabolic process  GO:0006520 cellular amino acid metabolic process  GO:0010675 regulation of cellular carbohydrate metabolic process  GO:0090181 regulation of cholesterol metabolic process  GO:0043112 receptor metabolic process  GO:0010906 regulation of glucose metabolic process  GO:0009100 glycoprotein metabolic process  GO:0006109 regulation of carbohydrate metabolic process  GO:0006518 peptide metabolic process  GO:0019400 alditol metabolic process  GO:0010676 positive regulation of cellular carbohydrate metabolic process  GO:0045913 positive regulation of carbohydrate metabolic process  GO:0006749 glutathione metabolic process  GO:0016052 carbohydrate catabolic process  GO:0043161 proteasomal ubiquitin-dependent protein catabolic process  GO:0010498 proteasomal protein catabolic process  GO:0009896 positive regulation of catabolic process  GO:0031331 positive regulation of cellular catabolic process  GO:0019941 modification-dependent protein catabolic process  GO:0016054 organic acid catabolic process  GO:0006511 ubiquitin-dependent protein catabolic process  GO:0043632 modification-dependent macromolecule catabolic process  GO:0044275 cellular carbohydrate catabolic process  GO:0032801 receptor catabolic process  GO:0009057 macromolecule catabolic process  GO:0030163 protein catabolic process  GO:0044282 small molecule catabolic process  GO:0051603 proteolysis involved in cellular protein catabolic process  GO:0044257 cellular protein catabolic process  GO:0043470 regulation of carbohydrate catabolic process | GO:0009895 negative regulation of catabolic process  GO:0050995 negative regulation of lipid catabolic process  GO:0072593 reactive oxygen species metabolic process  GO:0045834 positive regulation of lipid metabolic process  GO:0006575 cellular modified amino acid metabolic process  GO:0042743 hydrogen peroxide metabolic process  GO:0019362 pyridine nucleotide metabolic process  GO:0006733 oxidoreduction coenzyme metabolic process | GO:0044281 small molecule metabolic process  GO:0006793 phosphorus metabolic process  GO:0006629 lipid metabolic process  GO:0042180 cellular ketone metabolic process  GO:0044255 cellular lipid metabolic process  GO:0019752 carboxylic acid metabolic process  GO:0005975 carbohydrate metabolic process  GO:0006082 organic acid metabolic process  GO:1901135 carbohydrate derivative metabolic process  GO:0071704 organic substance metabolic process  GO:0032787 monocarboxylic acid metabolic process  GO:0019318 hexose metabolic process  GO:0006631 fatty acid metabolic process  GO:0005996 monosaccharide metabolic process  GO:0010604 positive regulation of macromolecule metabolic process  GO:0006732 coenzyme metabolic process  GO:0042278 purine nucleoside metabolic process  GO:0009116 nucleoside metabolic process  GO:0032268 regulation of cellular protein metabolic process  GO:0046128 purine ribonucleoside metabolic process  GO:0009119 ribonucleoside metabolic process  GO:0019637 organophosphate metabolic process  GO:0009893 positive regulation of metabolic process  GO:0031325 positive regulation of cellular metabolic process  GO:0009892 negative regulation of metabolic process  GO:0010605 negative regulation of macromolecule metabolic process  GO:0031324 negative regulation of cellular metabolic process  GO:0044262 cellular carbohydrate metabolic process  GO:1901068 guanosine-containing compound metabolic process  GO:0006006 glucose metabolic process  GO:0019220 regulation of phosphate metabolic process  GO:0019216 regulation of lipid metabolic process  GO:0046039 GTP metabolic process  GO:0009141 nucleoside triphosphate metabolic process  GO:0006066 alcohol metabolic process  GO:0009144 purine nucleoside triphosphate metabolic process  GO:0055086 nucleobase-containing small molecule metabolic process  GO:0046483 heterocycle metabolic process  GO:0009199 ribonucleoside triphosphate metabolic process  GO:0009205 purine ribonucleoside triphosphate metabolic process  GO:0009259 ribonucleotide metabolic process  GO:0051246 regulation of protein metabolic process  GO:0006753 nucleoside phosphate metabolic process  GO:0032270 positive regulation of cellular protein metabolic process  GO:0006644 phospholipid metabolic process  GO:0009150 purine ribonucleotide metabolic process  GO:0046486 glycerolipid metabolic process  GO:0051254 positive regulation of RNA metabolic process  GO:0009117 nucleotide metabolic process  GO:0010562 positive regulation of phosphorus metabolic process  GO:0016125 sterol metabolic process  GO:0051253 negative regulation of RNA metabolic process  GO:0072521 purine-containing compound metabolic process  GO:0008203 cholesterol metabolic process  GO:0006140 regulation of nucleotide metabolic process  GO:0006638 neutral lipid metabolic process  GO:0051172 negative regulation of nitrogen compound metabolic process  GO:0051247 positive regulation of protein metabolic process  GO:0006639 acylglycerol metabolic process  GO:0045934 negative regulation of nucleobase-containing compound metabolic process  GO:0045935 positive regulation of nucleobase-containing compound metabolic process  GO:1900542 regulation of purine nucleotide metabolic process  GO:0051173 positive regulation of nitrogen compound metabolic process  GO:0006641 triglyceride metabolic process  GO:0006163 purine nucleotide metabolic process  GO:0006650 glycerophospholipid metabolic process  GO:0008202 steroid metabolic process  GO:0009056 catabolic process  GO:0044248 cellular catabolic process  GO:0009894 regulation of catabolic process  GO:0031329 regulation of cellular catabolic process  GO:0033124 regulation of GTP catabolic process  GO:0030811 regulation of nucleotide catabolic process  GO:1901136 carbohydrate derivative catabolic process  GO:0046434 organophosphate catabolic process  GO:0044242 cellular lipid catabolic process  GO:0006184 GTP catabolic process  GO:0006152 purine nucleoside catabolic process  GO:0009164 nucleoside catabolic process  GO:0016042 lipid catabolic process  GO:0042454 ribonucleoside catabolic process  GO:1901069 guanosine-containing compound catabolic process  GO:1901292 nucleoside phosphate catabolic process  GO:0034655 nucleobase-containing compound catabolic process  GO:0009166 nucleotide catabolic process  GO:0044270 cellular nitrogen compound catabolic process  GO:0006195 purine nucleotide catabolic process  GO:0009143 nucleoside triphosphate catabolic process  GO:0009146 purine nucleoside triphosphate catabolic process  GO:0072523 purine-containing compound catabolic process  GO:0009203 ribonucleoside triphosphate catabolic process  GO:0009261 ribonucleotide catabolic process  GO:0009207 purine ribonucleoside triphosphate catabolic process  GO:0046700 heterocycle catabolic process  GO:0009154 purine ribonucleotide catabolic process  GO:0009062 fatty acid catabolic process  GO:0072329 monocarboxylic acid catabolic process  GO:0019320 hexose catabolic process  GO:0046365 monosaccharide catabolic process  GO:0050994 regulation of lipid catabolic process  GO:0006007 glucose catabolic process |
| Others | GO:0065003 macromolecular complex assembly  GO:0034622 cellular macromolecular complex assembly  GO:0043623 cellular protein complex assembly  GO:0031334 positive regulation of protein complex assembly  GO:0034621 cellular macromolecular complex subunit organization  GO:0032273 positive regulation of protein polymerization  GO:0070482 response to oxygen levels  GO:0043254 regulation of protein complex assembly  GO:0036293 response to decreased oxygen levels  GO:0048193 Golgi vesicle transport  GO:0032271 regulation of protein polymerization  GO:0055114 oxidation-reduction process  GO:0001666 response to hypoxia  GO:0006091 generation of precursor metabolites and energy  GO:0051348 negative regulation of transferase activity  GO:0001933 negative regulation of protein phosphorylation  GO:0006893 Golgi to plasma membrane transport  GO:0033673 negative regulation of kinase activity  GO:1901137 carbohydrate derivative biosynthetic process  GO:0010638 positive regulation of organelle organization  GO:0033043 regulation of organelle organization  GO:0018105 peptidyl-serine phosphorylation  GO:0019319 hexose biosynthetic process  GO:0070925 organelle assembly  GO:0034504 protein localization to nucleus  GO:0009628 response to abiotic stimulus  GO:0044087 regulation of cellular component biogenesis  GO:0046364 monosaccharide biosynthetic process  GO:0042326 negative regulation of phosphorylation  GO:0006469 negative regulation of protein kinase activity  GO:0043001 Golgi to plasma membrane protein transport  GO:0071901 negative regulation of protein serine/threonine kinase activity  GO:0051169 nuclear transport  GO:0006913 nucleocytoplasmic transport  GO:0046320 regulation of fatty acid oxidation  GO:0006892 post-Golgi vesicle-mediated transport  GO:0006914 autophagy  GO:0016051 carbohydrate biosynthetic process  GO:0017038 protein import  GO:0034599 cellular response to oxidative stress  GO:0051259 protein oligomerization  GO:0006606 protein import into nucleus  GO:0051260 protein homooligomerization  GO:0018193 peptidyl-amino acid modification  GO:0046822 regulation of nucleocytoplasmic transport  GO:0006979 response to oxidative stress  GO:0042692 muscle cell differentiation  GO:0033674 positive regulation of kinase activity  GO:0051170 nuclear import  GO:0006094 gluconeogenesis  GO:0046835 carbohydrate phosphorylation  GO:0018209 peptidyl-serine modification  GO:0022900 electron transport chain  GO:0007173 epidermal growth factor receptor signaling pathway  GO:0010741 negative regulation of intracellular protein kinase cascade  GO:0051347 positive regulation of transferase activity  GO:0045860 positive regulation of protein kinase activity  GO:0007051 spindle organization  GO:2001238 positive regulation of extrinsic apoptotic signaling pathway  GO:0031400 negative regulation of protein modification process  GO:0030833 regulation of actin filament polymerization  GO:0097190 apoptotic signaling pathway  GO:0030041 actin filament polymerization  GO:0032387 negative regulation of intracellular transport  GO:0070085 glycosylation  GO:0071453 cellular response to oxygen levels  GO:0043467 regulation of generation of precursor metabolites and energy  GO:0031998 regulation of fatty acid beta-oxidation  GO:0051146 striated muscle cell differentiation  GO:0001783 B cell apoptotic process  GO:0051188 cofactor biosynthetic process  GO:0030514 negative regulation of BMP signaling pathway  GO:0097191 extrinsic apoptotic signaling pathway  GO:0042991 transcription factor import into nucleus  GO:0006096 glycolysis  GO:0002902 regulation of B cell apoptotic process  GO:0042990 regulation of transcription factor import into nucleus  GO:0009101 glycoprotein biosynthetic process  GO:0044092 negative regulation of molecular function  GO:2001235 positive regulation of apoptotic signaling pathway  GO:0042306 regulation of protein import into nucleus  GO:0046823 negative regulation of nucleocytoplasmic transport  GO:0048711 positive regulation of astrocyte differentiation  GO:0007033 vacuole organization  GO:0046890 regulation of lipid biosynthetic process  GO:0042773 ATP synthesis coupled electron transport  GO:0055088 lipid homeostasis  GO:0022407 regulation of cell-cell adhesion  GO:0036294 cellular response to decreased oxygen levels  GO:0015980 energy derivation by oxidation of organic compounds  GO:0016567 protein ubiquitination  GO:0032446 protein modification by small protein conjugation  GO:0070228 regulation of lymphocyte apoptotic process  GO:0042594 response to starvation  GO:0033003 regulation of mast cell activation  GO:0006974 response to DNA damage stimulus  GO:0042632 cholesterol homeostasis  GO:0030832 regulation of actin filament length | GO:0051239 regulation of multicellular organismal process  GO:0006928 cellular component movement  GO:0032879 regulation of localization  GO:0023056 positive regulation of signaling  GO:0010647 positive regulation of cell communication  GO:0048584 positive regulation of response to stimulus  GO:0030030 cell projection organization  GO:0051270 regulation of cellular component movement  GO:0007155 cell adhesion  GO:0032869 cellular response to insulin stimulus  GO:0022610 biological adhesion  GO:0030097 hemopoiesis  GO:0045944 positive regulation of transcription from RNA polymerase II promoter  GO:0022008 neurogenesis  GO:0002376 immune system process  GO:0032321 positive regulation of Rho GTPase activity  GO:0051090 regulation of sequence-specific DNA binding transcription factor activity  GO:0030855 epithelial cell differentiation  GO:0048699 generation of neurons  GO:0045597 positive regulation of cell differentiation  GO:0010959 regulation of metal ion transport  GO:0016337 cell-cell adhesion  GO:0051272 positive regulation of cellular component movement  GO:0006869 lipid transport  GO:0097285 cell-type specific apoptotic process  GO:0030099 myeloid cell differentiation  GO:0032870 cellular response to hormone stimulus  GO:0031663 lipopolysaccharide-mediated signaling pathway  GO:0048585 negative regulation of response to stimulus  GO:0030278 regulation of ossification  GO:0051050 positive regulation of transport  GO:0030182 neuron differentiation  GO:0001775 cell activation  GO:0008286 insulin receptor signaling pathway  GO:0048041 focal adhesion assembly  GO:0045321 leukocyte activation  GO:0002507 tolerance induction  GO:0008643 carbohydrate transport  GO:0008360 regulation of cell shape  GO:0031344 regulation of cell projection organization  GO:0002521 leukocyte differentiation  GO:0007266 Rho protein signal transduction  GO:0090150 establishment of protein localization to membrane  GO:0002275 myeloid cell activation involved in immune response  GO:0050767 regulation of neurogenesis  GO:0048878 chemical homeostasis  GO:0045596 negative regulation of cell differentiation  GO:0043523 regulation of neuron apoptotic process  GO:0030031 cell projection assembly  GO:0051049 regulation of transport  GO:0006470 protein dephosphorylation  GO:0010740 positive regulation of intracellular protein kinase cascade  GO:0090002 establishment of protein localization to plasma membrane  GO:0071216 cellular response to biotic stimulus  GO:0045664 regulation of neuron differentiation  GO:0060341 regulation of cellular localization  GO:0006873 cellular ion homeostasis  GO:0051402 neuron apoptotic process  GO:0019725 cellular homeostasis  GO:2001234 negative regulation of apoptotic signaling pathway  GO:0070997 neuron death  GO:0051130 positive regulation of cellular component organization  GO:0034101 erythrocyte homeostasis  GO:0030218 erythrocyte differentiation  GO:0007179 transforming growth factor beta receptor signaling pathway  GO:0002263 cell activation involved in immune response  GO:0071219 cellular response to molecule of bacterial origin  GO:0008154 actin polymerization or depolymerization  GO:0032388 positive regulation of intracellular transport  GO:0002224 toll-like receptor signaling pathway  GO:0031346 positive regulation of cell projection organization  GO:0043122 regulation of I-kappaB kinase/NF-kappaB cascade  GO:0002279 mast cell activation involved in immune response  GO:0001525 angiogenesis  GO:0016044 cellular membrane organization  GO:0002682 regulation of immune system process  GO:0030098 lymphocyte differentiation  GO:2001237 negative regulation of extrinsic apoptotic signaling pathway  GO:0045444 fat cell differentiation  GO:0050801 ion homeostasis  GO:0032494 response to peptidoglycan  GO:0002237 response to molecule of bacterial origin  GO:0007041 lysosomal transport  GO:0055082 cellular chemical homeostasis  GO:2000021 regulation of ion homeostasis  GO:0034142 toll-like receptor 4 signaling pathway  GO:0018108 peptidyl-tyrosine phosphorylation  GO:0030048 actin filament-based movement  GO:0046847 filopodium assembly  GO:0007034 vacuolar transport  GO:0051240 positive regulation of multicellular organismal process  GO:0002448 mast cell mediated immunity  GO:0007219 Notch signaling pathway  GO:0009060 aerobic respiration  GO:0010830 regulation of myotube differentiation  GO:0031529 ruffle organization  GO:0043299 leukocyte degranulation  GO:0009605 response to external stimulus  GO:0006917 induction of apoptosis  GO:0032970 regulation of actin filament-based process  GO:0046635 positive regulation of alpha-beta T cell activation  GO:0090316 positive regulation of intracellular protein transport  GO:0046474 glycerophospholipid biosynthetic process  GO:0034219 carbohydrate transmembrane transport  GO:0051017 actin filament bundle assembly  GO:0006875 cellular metal ion homeostasis  GO:0072659 protein localization to plasma membrane  GO:0006916 anti-apoptosis  GO:0043433 negative regulation of sequence-specific DNA binding transcription factor activity  GO:0010453 regulation of cell fate commitment  GO:0035088 establishment or maintenance of apical/basal cell polarity  GO:0090066 regulation of anatomical structure size  GO:0071222 cellular response to lipopolysaccharide  GO:0017015 regulation of transforming growth factor beta receptor signaling pathway  GO:0051279 regulation of release of sequestered calcium ion into cytosol  GO:0031668 cellular response to extracellular stimulus  GO:0030217 T cell differentiation  GO:0044319 wound healing, spreading of cells  GO:0045055 regulated secretory pathway  GO:0060251 regulation of glial cell proliferation  GO:0012502 induction of programmed cell death  GO:0033077 T cell differentiation in thymus  GO:0007585 respiratory gaseous exchange  GO:0001816 cytokine production  GO:0032496 response to lipopolysaccharide  GO:0046942 carboxylic acid transport  GO:0043270 positive regulation of ion transport  GO:0007163 establishment or maintenance of cell polarity  GO:0051480 cytosolic calcium ion homeostasis  GO:0002028 regulation of sodium ion transport  GO:0015909 long-chain fatty acid transport  GO:0015908 fatty acid transport | GO:0033036 macromolecule localization  GO:0016310 phosphorylation  GO:0045184 establishment of protein localization  GO:0034613 cellular protein localization  GO:0070727 cellular macromolecule localization  GO:0046907 intracellular transport  GO:0008104 protein localization  GO:0015031 protein transport  GO:0035556 intracellular signal transduction  GO:0006996 organelle organization  GO:0051641 cellular localization  GO:0009966 regulation of signal transduction  GO:0016265 death  GO:0008219 cell death  GO:0006886 intracellular protein transport  GO:0012501 programmed cell death  GO:0006915 apoptotic process  GO:0065009 regulation of molecular function  GO:0050790 regulation of catalytic activity  GO:0006468 protein phosphorylation  GO:0051649 establishment of localization in cell  GO:0044093 positive regulation of molecular function  GO:0016192 vesicle-mediated transport  GO:0023051 regulation of signaling  GO:0022607 cellular component assembly  GO:0048583 regulation of response to stimulus  GO:0043085 positive regulation of catalytic activity  GO:0042325 regulation of phosphorylation  GO:0043933 macromolecular complex subunit organization  GO:0043087 regulation of GTPase activity  GO:0033554 cellular response to stress  GO:0071822 protein complex subunit organization  GO:0044085 cellular component biogenesis  GO:0008610 lipid biosynthetic process  GO:0006461 protein complex assembly  GO:0070271 protein complex biogenesis  GO:0071844 cellular component assembly at cellular level  GO:0007243 intracellular protein kinase cascade  GO:0031399 regulation of protein modification process  GO:0001932 regulation of protein phosphorylation  GO:0043547 positive regulation of GTPase activity  GO:0051056 regulation of small GTPase mediated signal transduction  GO:0006605 protein targeting  GO:0051338 regulation of transferase activity  GO:0010941 regulation of cell death  GO:0043549 regulation of kinase activity  GO:0033365 protein localization to organelle  GO:0051258 protein polymerization  GO:0042981 regulation of apoptotic process  GO:0043067 regulation of programmed cell death  GO:0046578 regulation of Ras protein signal transduction  GO:0030029 actin filament-based process  GO:0070887 cellular response to chemical stimulus  GO:0007160 cell-matrix adhesion  GO:0010942 positive regulation of cell death  GO:0007264 small GTPase mediated signal transduction  GO:0031401 positive regulation of protein modification process  GO:0007167 enzyme linked receptor protein signaling pathway  GO:0006635 fatty acid beta-oxidation  GO:0043065 positive regulation of apoptotic process  GO:0034330 cell junction organization  GO:0009890 negative regulation of biosynthetic process  GO:0043068 positive regulation of programmed cell death  GO:0006366 transcription from RNA polymerase II promoter  GO:0031589 cell-substrate adhesion  GO:0031327 negative regulation of cellular biosynthetic process  GO:0045859 regulation of protein kinase activity  GO:0044283 small molecule biosynthetic process  GO:0045893 positive regulation of transcription, DNA-dependent  GO:0010627 regulation of intracellular protein kinase cascade  GO:0032318 regulation of Ras GTPase activity  GO:0019395 fatty acid oxidation  GO:0016053 organic acid biosynthetic process  GO:0010557 positive regulation of macromolecule biosynthetic process  GO:0030838 positive regulation of actin filament polymerization  GO:0042327 positive regulation of phosphorylation  GO:0042127 regulation of cell proliferation  GO:0034440 lipid oxidation  GO:0032386 regulation of intracellular transport  GO:0001934 positive regulation of protein phosphorylation  GO:0030258 lipid modification  GO:0048008 platelet-derived growth factor receptor signaling pathway  GO:0006357 regulation of transcription from RNA polymerase II promoter  GO:0008283 cell proliferation  GO:0010558 negative regulation of macromolecule biosynthetic process  GO:2000113 negative regulation of cellular macromolecule biosynthetic process  GO:0051128 regulation of cellular component organization  GO:0010628 positive regulation of gene expression  GO:0007169 transmembrane receptor protein tyrosine kinase signaling pathway  GO:0006412 translation  GO:0051345 positive regulation of hydrolase activity  GO:0033157 regulation of intracellular protein transport  GO:0032880 regulation of protein localization  GO:0034329 cell junction assembly  GO:0006897 endocytosis  GO:0009719 response to endogenous stimulus  GO:0008654 phospholipid biosynthetic process  GO:0035023 regulation of Rho protein signal transduction  GO:0010608 posttranscriptional regulation of gene expression  GO:0045017 glycerolipid biosynthetic process  GO:0010629 negative regulation of gene expression  GO:0010876 lipid localization  GO:0006633 fatty acid biosynthetic process  GO:0009725 response to hormone stimulus  GO:0019915 lipid storage  GO:0007005 mitochondrion organization  GO:0045892 negative regulation of transcription, DNA-dependent  GO:0030032 lamellipodium assembly  GO:0009968 negative regulation of signal transduction  GO:0009891 positive regulation of biosynthetic process  GO:0008285 negative regulation of cell proliferation  GO:0071375 cellular response to peptide hormone stimulus  GO:0070201 regulation of establishment of protein localization  GO:0010646 regulation of cell communication  GO:0023057 negative regulation of signaling  GO:0010648 negative regulation of cell communication  GO:0032844 regulation of homeostatic process  GO:0071310 cellular response to organic substance  GO:0045216 cell-cell junction organization  GO:2001233 regulation of apoptotic signaling pathway  GO:0031328 positive regulation of cellular biosynthetic process  GO:0071495 cellular response to endogenous stimulus  GO:0043069 negative regulation of programmed cell death  GO:0043066 negative regulation of apoptotic process  GO:0060548 negative regulation of cell death  GO:0032868 response to insulin stimulus  GO:0016311 dephosphorylation  GO:0007015 actin filament organization  GO:0043434 response to peptide hormone stimulus  GO:0032320 positive regulation of Ras GTPase activity  GO:0061024 membrane organization  GO:0000122 negative regulation of transcription from RNA polymerase II promoter  GO:0042592 homeostatic process  GO:0045595 regulation of cell differentiation  GO:0007009 plasma membrane organization  GO:0071702 organic substance transport  GO:0032319 regulation of Rho GTPase activity  GO:0010033 response to organic substance  GO:0014009 glial cell proliferation  GO:0006417 regulation of translation  GO:0002274 myeloid leukocyte activation  GO:0009611 response to wounding  GO:2001236 regulation of extrinsic apoptotic signaling pathway  GO:0040007 growth  GO:0010035 response to inorganic substance  GO:0007044 cell-substrate junction assembly  GO:0007265 Ras protein signal transduction  GO:0009967 positive regulation of signal transduction  GO:0051223 regulation of protein transport  GO:0007249 I-kappaB kinase/NF-kappaB cascade  GO:0035264 multicellular organism growth |

**Table S4: Thyroid genes differentially expressed in PPFP^Thy^;Pten^Thy-/-^ mice versus Pten^Thy-/-^ mice on a normal chow diet, using *q*<0.05 and absolute fold change >2 as cut-offs for significance**

| **Gene Symbol** | **log(Fold Change)** | ***p*-value** | ***q*-value** | **PPFP^Thy^;Pten^Thy-/-^ vs Pten^Thy-/-^** |
| --- | --- | --- | --- | --- |
| Irx2 | 6.445304504 | 0 | 0 | up |
| Ccdc3 | 5.781136307 | 0 | 0 | up |
| Rasl11b | 6.10157188 | 3.89E-286 | 1.88E-282 | up |
| 1700011M02Rik | 9.629683288 | 1.36E-208 | 4.93E-205 | up |
| Dmrtc1a | 7.034606178 | 1.73E-206 | 5.03E-203 | up |
| Slc2a12 | 5.634289604 | 1.68E-198 | 4.07E-195 | up |
| Gldc | 8.666818685 | 6.62E-194 | 1.37E-190 | up |
| Gm3002 | 5.621466444 | 7.71E-163 | 1.40E-159 | up |
| Ncam1 | 3.753925761 | 8.81E-150 | 1.42E-146 | up |
| Upk3b | 8.354705275 | 1.31E-146 | 1.73E-143 | up |
| Coro2b | 3.753971486 | 2.55E-144 | 3.08E-141 | up |
| Ltbp2 | 3.453174716 | 4.65E-133 | 5.19E-130 | up |
| 1810020O05Rik | 6.770616622 | 4.43E-130 | 4.59E-127 | up |
| Snap91 | 4.928937679 | 6.38E-130 | 6.17E-127 | up |
| Scn5a | 4.912162295 | 2.00E-125 | 1.71E-122 | up |
| Ak7 | 7.282891176 | 8.06E-122 | 6.15E-119 | up |
| Klhl29 | 4.082065579 | 3.36E-120 | 2.44E-117 | up |
| Gng2 | 3.048339954 | 3.94E-118 | 2.72E-115 | up |
| Parvb | 2.73524879 | 5.94E-116 | 3.59E-113 | up |
| Phactr1 | 3.898642746 | 2.46E-110 | 1.43E-107 | up |
| Tspan18 | 2.866404428 | 6.88E-110 | 3.84E-107 | up |
| Chst13 | 8.443116206 | 1.27E-107 | 6.84E-105 | up |
| Syt13 | 5.142317704 | 2.12E-107 | 1.10E-104 | up |
| Aqp3 | 4.008366627 | 2.74E-107 | 1.37E-104 | up |
| Snx31 | 4.068367671 | 2.81E-101 | 1.32E-98 | up |
| Corin | 6.991822171 | 5.43E-101 | 2.46E-98 | up |
| Mme | 5.17596947 | 5.76E-101 | 2.53E-98 | up |
| Rasgrf2 | 2.998307854 | 7.20E-101 | 3.07E-98 | up |
| Ephx2 | 2.804252335 | 5.65E-100 | 2.28E-97 | up |
| Acrv1 | 6.701848325 | 3.10E-98 | 1.22E-95 | up |
| Grik3 | 4.191521303 | 4.01E-97 | 1.49E-94 | up |
| Col26a1 | 4.291720882 | 8.09E-96 | 2.93E-93 | up |
| Ret | 4.487856557 | 4.25E-95 | 1.50E-92 | up |
| Kbtbd11 | 3.014112428 | 2.37E-94 | 8.18E-92 | up |
| Zfyve26 | 2.122442091 | 2.47E-93 | 8.16E-91 | up |
| Hsd3b6 | 8.920734355 | 4.53E-93 | 1.46E-90 | up |
| Cldn2 | 3.24821707 | 5.23E-93 | 1.65E-90 | up |
| Dctd | 4.158187013 | 3.39E-92 | 1.05E-89 | up |
| Smoc1 | 5.360312805 | 1.46E-91 | 4.16E-89 | up |
| Thsd4 | 4.424444703 | 1.24E-90 | 3.33E-88 | up |
| Igf2bp1 | 4.881332094 | 1.38E-90 | 3.63E-88 | up |
| Acsbg1 | 4.568936037 | 2.35E-90 | 5.99E-88 | up |
| Fa2h | 4.393381998 | 2.74E-90 | 6.84E-88 | up |
| Sox12 | 2.939700582 | 2.35E-89 | 5.77E-87 | up |
| Ryr3 | 7.432981564 | 6.36E-89 | 1.54E-86 | up |
| Chek1 | 4.139527441 | 1.06E-87 | 2.52E-85 | up |
| Adcy5 | 4.773868254 | 2.24E-87 | 5.25E-85 | up |
| Phyhipl | 6.312019944 | 2.21E-86 | 5.00E-84 | up |
| Aldob | 8.713481174 | 4.31E-86 | 9.61E-84 | up |
| Gm20554 | 4.335782531 | 1.42E-85 | 3.08E-83 | up |
| Shisa2 | 4.349088107 | 9.28E-85 | 1.95E-82 | up |
| Hpgd | 3.270591049 | 2.96E-84 | 6.13E-82 | up |
| Opcml | 4.688020281 | 3.55E-83 | 7.25E-81 | up |
| Akap12 | 1.912496626 | 6.12E-81 | 1.18E-78 | up |
| Slc51a | 7.125297181 | 2.97E-80 | 5.59E-78 | up |
| Rdh11 | 2.223962217 | 4.70E-79 | 8.74E-77 | up |
| Camk1g | 5.02835628 | 1.18E-78 | 2.17E-76 | up |
| Comp | 4.625113719 | 1.42E-77 | 2.54E-75 | up |
| Fam43a | 1.908074393 | 2.09E-77 | 3.70E-75 | up |
| Npr3 | 3.944820931 | 7.01E-77 | 1.20E-74 | up |
| Reln | 3.661493616 | 1.86E-76 | 3.14E-74 | up |
| Prima1 | 4.866152055 | 2.12E-76 | 3.53E-74 | up |
| Gstk1 | 3.258323709 | 4.53E-76 | 7.47E-74 | up |
| Vcan | 3.238963816 | 5.66E-76 | 9.22E-74 | up |
| Clec10a | 2.501283547 | 3.12E-74 | 5.03E-72 | up |
| Col11a1 | 5.393588733 | 3.78E-74 | 6.03E-72 | up |
| Bdh1 | 2.86092613 | 4.16E-73 | 6.49E-71 | up |
| Nlrp1a | 4.573805571 | 4.90E-73 | 7.57E-71 | up |
| Grb10 | 3.161989777 | 7.84E-71 | 1.16E-68 | up |
| Eaf2 | 2.462838614 | 4.25E-70 | 6.22E-68 | up |
| Murc | 6.597955295 | 5.02E-70 | 7.28E-68 | up |
| Myo3b | 4.442864281 | 1.28E-68 | 1.80E-66 | up |
| Galnt10 | 1.836259799 | 2.94E-67 | 4.03E-65 | up |
| Adra1b | 3.967357048 | 4.17E-67 | 5.55E-65 | up |
| Rgs19 | 1.730510513 | 1.08E-66 | 1.40E-64 | up |
| Tsku | 2.619847546 | 1.17E-66 | 1.51E-64 | up |
| Ppp4r4 | 3.766276662 | 5.89E-66 | 7.43E-64 | up |
| Gm9112 | 10.48725193 | 6.33E-66 | 7.91E-64 | up |
| 5830411N06Rik | 8.919597085 | 7.51E-66 | 9.23E-64 | up |
| Fam132a | 2.235598453 | 8.82E-66 | 1.07E-63 | up |
| Gadd45g | 3.613932751 | 2.83E-65 | 3.39E-63 | up |
| Ripply1 | 4.991151376 | 3.30E-65 | 3.92E-63 | up |
| Ctsf | 1.931175136 | 5.72E-65 | 6.70E-63 | up |
| Rcan2 | 3.062955177 | 9.69E-65 | 1.12E-62 | up |
| Dmrtc1b | 9.628516722 | 2.70E-64 | 3.08E-62 | up |
| Gsto1 | 2.798992397 | 5.71E-64 | 6.37E-62 | up |
| Alox5 | 3.484843796 | 1.95E-63 | 2.16E-61 | up |
| 1700003M02Rik | 2.763379657 | 2.78E-63 | 3.06E-61 | up |
| S100a5 | 4.831429994 | 3.40E-63 | 3.71E-61 | up |
| 2410131K14Rik | 2.304016219 | 4.17E-63 | 4.51E-61 | up |
| Pitpnm2 | 2.307442321 | 5.71E-63 | 6.14E-61 | up |
| Grik4 | 3.287759802 | 6.89E-63 | 7.35E-61 | up |
| 2810459M11Rik | 5.178457765 | 1.82E-62 | 1.91E-60 | up |
| Vwa5b1 | 8.907639465 | 5.93E-62 | 6.19E-60 | up |
| Slc23a1 | 1.991672084 | 1.08E-61 | 1.11E-59 | up |
| Slc8a1 | 3.009536727 | 2.80E-61 | 2.84E-59 | up |
| Col5a1 | 2.597357836 | 1.33E-60 | 1.33E-58 | up |
| Frem2 | 4.00657258 | 1.40E-60 | 1.39E-58 | up |
| Pde1a | 2.654220774 | 6.76E-60 | 6.53E-58 | up |
| Scgb1c1 | 5.640290582 | 6.88E-60 | 6.61E-58 | up |
| Tmeff1 | 1.819759861 | 1.54E-59 | 1.47E-57 | up |
| Mogat1 | 3.458884179 | 1.65E-59 | 1.56E-57 | up |
| Nfil3 | 3.857456737 | 4.21E-59 | 3.96E-57 | up |
| F7 | 4.83560633 | 2.04E-58 | 1.86E-56 | up |
| Polr1a | 1.858373275 | 2.16E-58 | 1.96E-56 | up |
| Dao | 3.605985541 | 2.88E-58 | 2.59E-56 | up |
| Hmgcl | 1.471940287 | 7.48E-58 | 6.65E-56 | up |
| Rcl1 | 1.799090361 | 1.96E-57 | 1.72E-55 | up |
| Eomes | 7.418330292 | 7.03E-57 | 6.15E-55 | up |
| Ephb2 | 2.25343273 | 5.53E-56 | 4.75E-54 | up |
| Fn1 | 2.307741543 | 1.35E-55 | 1.15E-53 | up |
| Erc2 | 3.167859786 | 1.65E-55 | 1.39E-53 | up |
| Slc22a23 | 2.612113355 | 8.21E-55 | 6.77E-53 | up |
| Adap1 | 2.085070568 | 9.80E-55 | 7.99E-53 | up |
| Mgat5b | 4.900576553 | 1.20E-54 | 9.72E-53 | up |
| Palmd | 2.087752611 | 1.93E-54 | 1.56E-52 | up |
| LOC100861615 | 5.703836012 | 2.32E-54 | 1.85E-52 | up |
| Lrat | 3.290431301 | 2.76E-54 | 2.19E-52 | up |
| Sipa1l3 | 2.665152521 | 3.06E-54 | 2.41E-52 | up |
| Adamtsl1 | 2.890688451 | 3.48E-54 | 2.73E-52 | up |
| F10 | 5.294017001 | 4.84E-54 | 3.73E-52 | up |
| Plxna2 | 1.775130786 | 7.84E-54 | 6.02E-52 | up |
| Gclc | 1.568344469 | 1.50E-53 | 1.14E-51 | up |
| Prkd1 | 2.432564647 | 1.77E-53 | 1.33E-51 | up |
| Cdc42ep2 | 1.821356013 | 1.83E-53 | 1.37E-51 | up |
| Slc22a19 | 7.799147676 | 2.01E-53 | 1.49E-51 | up |
| Chrna2 | 5.884031136 | 2.38E-53 | 1.76E-51 | up |
| Car12 | 4.498218522 | 1.52E-52 | 1.10E-50 | up |
| Pparg | 3.859880406 | 2.16E-52 | 1.56E-50 | up |
| Cyp3a57 | 8.413706182 | 3.19E-52 | 2.28E-50 | up |
| Tnfrsf4 | 3.905298945 | 4.75E-52 | 3.34E-50 | up |
| Nfe2l3 | 3.822831445 | 9.86E-52 | 6.91E-50 | up |
| Ormdl3 | 1.338931561 | 1.12E-51 | 7.84E-50 | up |
| Sprn | 4.321341001 | 1.34E-51 | 9.32E-50 | up |
| St3gal5 | 1.576700182 | 2.10E-51 | 1.45E-49 | up |
| Tbx2 | 1.903310812 | 2.20E-51 | 1.51E-49 | up |
| Timp3 | 3.033764971 | 3.17E-51 | 2.17E-49 | up |
| Atp1a4 | 6.175537244 | 4.01E-51 | 2.73E-49 | up |
| Wasf1 | 2.695015452 | 6.54E-51 | 4.39E-49 | up |
| Dnm1 | 1.91968389 | 8.38E-51 | 5.60E-49 | up |
| 1500009L16Rik | 3.896048373 | 1.49E-50 | 9.86E-49 | up |
| Rbm11 | 5.000366074 | 5.16E-50 | 3.34E-48 | up |
| Gpam | 2.671830205 | 7.16E-50 | 4.60E-48 | up |
| Entpd6 | 1.662655874 | 8.84E-50 | 5.60E-48 | up |
| Sqle | 1.892297996 | 9.02E-50 | 5.69E-48 | up |
| Gpd1 | 2.231204612 | 9.13E-50 | 5.73E-48 | up |
| Kcnh1 | 4.935844123 | 9.90E-50 | 6.19E-48 | up |
| Pex11a | 2.342299958 | 6.34E-49 | 3.85E-47 | up |
| Prex1 | 1.857739987 | 7.18E-49 | 4.34E-47 | up |
| Rdh12 | 3.100799192 | 1.08E-48 | 6.52E-47 | up |
| Fitm2 | 2.205229893 | 3.77E-48 | 2.23E-46 | up |
| Tpbg | 1.602730512 | 4.00E-48 | 2.35E-46 | up |
| Pear1 | 2.041063708 | 4.50E-48 | 2.62E-46 | up |
| Tbc1d16 | 1.778373369 | 5.27E-48 | 3.05E-46 | up |
| Mttp | 2.599896004 | 6.41E-48 | 3.66E-46 | up |
| Slit1 | 4.579270922 | 1.19E-47 | 6.76E-46 | up |
| 2610019F03Rik | 1.785065902 | 1.29E-47 | 7.31E-46 | up |
| Vil1 | 2.259502487 | 1.52E-47 | 8.57E-46 | up |
| Gnao1 | 3.954978735 | 1.53E-47 | 8.57E-46 | up |
| Tiam2 | 3.016011836 | 1.63E-47 | 9.08E-46 | up |
| Ap3b2 | 3.226716154 | 1.69E-47 | 9.42E-46 | up |
| Shmt1 | 2.306473111 | 3.15E-47 | 1.73E-45 | up |
| Sorcs2 | 3.421049938 | 4.47E-47 | 2.45E-45 | up |
| Kif7 | 2.732220874 | 1.17E-46 | 6.31E-45 | up |
| Alpl | 2.32187056 | 1.17E-46 | 6.33E-45 | up |
| Hs3st3b1 | 2.725246066 | 9.99E-46 | 5.33E-44 | up |
| Adm2 | 3.771925738 | 1.15E-45 | 6.09E-44 | up |
| Cebpa | 1.419081172 | 1.45E-45 | 7.62E-44 | up |
| Dbt | 1.49337625 | 2.00E-45 | 1.04E-43 | up |
| Maob | 2.832575554 | 5.47E-45 | 2.82E-43 | up |
| Trib2 | 1.515948152 | 6.58E-45 | 3.39E-43 | up |
| Baiap2l2 | 5.207840019 | 1.20E-44 | 6.13E-43 | up |
| Net1 | 1.527710244 | 1.51E-44 | 7.67E-43 | up |
| Ces3a | 7.433624971 | 3.58E-44 | 1.80E-42 | up |
| Slc16a1 | 2.743776338 | 4.52E-44 | 2.27E-42 | up |
| Mlkl | 2.3707441 | 1.11E-43 | 5.54E-42 | up |
| Man1c1 | 1.645964392 | 1.38E-43 | 6.86E-42 | up |
| Tdrd9 | 3.157400505 | 2.55E-43 | 1.26E-41 | up |
| Bpifa6 | 8.586039699 | 2.58E-43 | 1.28E-41 | up |
| Gm19990 | 3.885297957 | 3.35E-43 | 1.65E-41 | up |
| Trim9 | 2.189959109 | 4.98E-43 | 2.44E-41 | up |
| Lrpprc | 1.295125186 | 6.22E-43 | 3.04E-41 | up |
| Mtg1 | 1.536794567 | 7.00E-43 | 3.41E-41 | up |
| Ucp3 | 3.163222341 | 8.55E-43 | 4.15E-41 | up |
| 1700018G05Rik | 8.648108909 | 1.42E-42 | 6.80E-41 | up |
| Nuak2 | 1.8047665 | 1.52E-42 | 7.30E-41 | up |
| Itga7 | 2.275941037 | 1.75E-42 | 8.37E-41 | up |
| Tnni3 | 4.19218138 | 2.73E-42 | 1.30E-40 | up |
| Mroh2a | 5.983460407 | 7.24E-42 | 3.40E-40 | up |
| Fgf4 | 7.676153887 | 1.15E-41 | 5.38E-40 | up |
| Gm5796 | 6.4936766 | 1.23E-41 | 5.72E-40 | up |
| Pabpc4l | 3.256951106 | 1.53E-41 | 7.06E-40 | up |
| Hpgds | 2.354221319 | 2.03E-41 | 9.28E-40 | up |
| Tlr5 | 2.342073259 | 1.22E-40 | 5.44E-39 | up |
| Emid1 | 3.078472878 | 1.73E-40 | 7.65E-39 | up |
| Cenpt | 1.289551594 | 1.82E-40 | 7.98E-39 | up |
| 4933402E13Rik | 4.592548395 | 1.84E-40 | 8.03E-39 | up |
| Zxda | 1.649338795 | 1.87E-40 | 8.17E-39 | up |
| Hsd17b14 | 3.205690419 | 2.08E-40 | 9.04E-39 | up |
| Fam122b | 1.991188439 | 2.10E-40 | 9.11E-39 | up |
| Cspg5 | 3.248727663 | 2.61E-40 | 1.12E-38 | up |
| Rasd1 | 2.244139427 | 2.21E-39 | 9.38E-38 | up |
| Bnc1 | 5.430103369 | 2.60E-39 | 1.10E-37 | up |
| Ltc4s | 2.407469918 | 2.97E-39 | 1.25E-37 | up |
| Gm3558 | 4.021585697 | 3.29E-39 | 1.38E-37 | up |
| Sox7 | 3.083725808 | 1.00E-38 | 4.14E-37 | up |
| Abcb6 | 1.391221128 | 1.12E-38 | 4.60E-37 | up |
| Rimklb | 4.000455486 | 1.21E-38 | 4.96E-37 | up |
| Plbd1 | 3.481685144 | 1.47E-38 | 6.00E-37 | up |
| Cyp2c70 | 4.853054045 | 1.57E-38 | 6.37E-37 | up |
| Itsn1 | 1.165557425 | 2.90E-38 | 1.17E-36 | up |
| Gm10406 | 3.581169478 | 3.33E-38 | 1.34E-36 | up |
| Echs1 | 1.356560427 | 5.27E-38 | 2.10E-36 | up |
| Myh10 | 1.910160828 | 5.72E-38 | 2.27E-36 | up |
| Mdn1 | 1.676854212 | 5.72E-38 | 2.27E-36 | up |
| Etfdh | 1.254774592 | 6.38E-38 | 2.52E-36 | up |
| D10Wsu102e | 1.249589073 | 1.01E-37 | 3.96E-36 | up |
| 1810041L15Rik | 3.390233975 | 1.13E-37 | 4.43E-36 | up |
| Ep400 | 1.481507487 | 1.20E-37 | 4.69E-36 | up |
| Ivl | 7.137267933 | 1.39E-37 | 5.40E-36 | up |
| Apex1 | 1.593237945 | 1.79E-37 | 6.89E-36 | up |
| Gm2897 | 6.998861299 | 3.05E-37 | 1.16E-35 | up |
| Lix1l | 1.688743851 | 3.08E-37 | 1.17E-35 | up |
| Elmod1 | 4.706993707 | 3.13E-37 | 1.18E-35 | up |
| Trim16 | 1.428527368 | 3.62E-37 | 1.36E-35 | up |
| Cdh6 | 3.849807748 | 3.82E-37 | 1.44E-35 | up |
| Plk2 | 1.464319284 | 6.78E-37 | 2.53E-35 | up |
| Guca1b | 6.863350544 | 6.96E-37 | 2.59E-35 | up |
| Acat1 | 1.809932216 | 8.71E-37 | 3.21E-35 | up |
| Msx2 | 7.652734496 | 1.00E-36 | 3.67E-35 | up |
| Rrs1 | 1.11619764 | 3.10E-36 | 1.12E-34 | up |
| Kif6 | 3.657149717 | 5.85E-36 | 2.10E-34 | up |
| Lrrn2 | 2.772355794 | 6.40E-36 | 2.29E-34 | up |
| Lmnb2 | 1.163558645 | 1.93E-35 | 6.74E-34 | up |
| Ccnd1 | 1.830257743 | 2.09E-35 | 7.26E-34 | up |
| Ikzf3 | 5.123096954 | 2.13E-35 | 7.38E-34 | up |
| Cped1 | 2.272344908 | 2.80E-35 | 9.68E-34 | up |
| Cacna1h | 2.151310559 | 3.23E-35 | 1.11E-33 | up |
| Mesdc1 | 1.396791798 | 5.45E-35 | 1.85E-33 | up |
| Kpna3 | 1.313762684 | 6.96E-35 | 2.35E-33 | up |
| Dpysl5 | 3.956915746 | 8.98E-35 | 3.01E-33 | up |
| Acat2 | 1.898117337 | 9.32E-35 | 3.12E-33 | up |
| Adamts15 | 2.77742623 | 9.49E-35 | 3.17E-33 | up |
| Galnt18 | 1.853687119 | 1.13E-34 | 3.75E-33 | up |
| Nipsnap1 | 1.540442448 | 1.21E-34 | 4.00E-33 | up |
| Kctd17 | 1.208258238 | 1.63E-34 | 5.27E-33 | up |
| Slc6a18 | 3.949644197 | 2.38E-34 | 7.64E-33 | up |
| Dlgap2 | 4.196422874 | 2.49E-34 | 7.98E-33 | up |
| Atp10b | 3.208676877 | 2.64E-34 | 8.44E-33 | up |
| Cadm4 | 1.123117184 | 2.84E-34 | 9.07E-33 | up |
| 9130227L01Rik | 7.576716293 | 3.74E-34 | 1.19E-32 | up |
| Kif17 | 2.966673762 | 4.21E-34 | 1.34E-32 | up |
| Htra1 | 1.733151403 | 4.50E-34 | 1.43E-32 | up |
| Amph | 2.162490442 | 5.63E-34 | 1.78E-32 | up |
| Steap1 | 4.72792891 | 5.76E-34 | 1.81E-32 | up |
| Inhbb | 2.024088282 | 5.75E-34 | 1.81E-32 | up |
| Smyd5 | 1.390417189 | 6.15E-34 | 1.93E-32 | up |
| Filip1 | 2.096331634 | 6.55E-34 | 2.05E-32 | up |
| Galnt12 | 1.452496952 | 7.33E-34 | 2.27E-32 | up |
| 9030624J02Rik | 1.093213185 | 7.31E-34 | 2.27E-32 | up |
| Sema6d | 1.73578255 | 1.30E-33 | 4.00E-32 | up |
| Glis1 | 4.343376886 | 1.80E-33 | 5.54E-32 | up |
| Dennd2a | 1.56897748 | 2.26E-33 | 6.94E-32 | up |
| Chsy1 | 2.074878008 | 2.41E-33 | 7.37E-32 | up |
| Igfbp2 | 5.896324067 | 2.43E-33 | 7.41E-32 | up |
| Kcne3 | 2.933570637 | 2.86E-33 | 8.72E-32 | up |
| 2010204K13Rik | 3.544755617 | 2.88E-33 | 8.75E-32 | up |
| Cluh | 1.163961366 | 3.56E-33 | 1.08E-31 | up |
| Irs2 | 2.540719667 | 5.30E-33 | 1.60E-31 | up |
| Nuggc | 4.552126812 | 8.04E-33 | 2.42E-31 | up |
| Dock9 | 1.277379891 | 1.06E-32 | 3.17E-31 | up |
| Acad9 | 2.221412297 | 1.25E-32 | 3.71E-31 | up |
| Atad3a | 1.251577941 | 1.27E-32 | 3.75E-31 | up |
| Gpc2 | 1.899588316 | 1.54E-32 | 4.55E-31 | up |
| Txnrd3 | 1.572007202 | 1.66E-32 | 4.90E-31 | up |
| Gfm1 | 1.144734026 | 1.88E-32 | 5.49E-31 | up |
| Peg3 | 3.006745043 | 2.00E-32 | 5.82E-31 | up |
| Hsd17b7 | 1.693854816 | 2.27E-32 | 6.61E-31 | up |
| Asb2 | 3.789572856 | 3.33E-32 | 9.62E-31 | up |
| Tnfsf18 | 4.318786276 | 3.75E-32 | 1.08E-30 | up |
| Ddx51 | 1.264028321 | 5.09E-32 | 1.45E-30 | up |
| Gjb5 | 2.571261839 | 1.08E-31 | 3.05E-30 | up |
| 4930502E18Rik | 7.88394132 | 1.41E-31 | 3.95E-30 | up |
| Slc22a1 | 6.129948442 | 2.45E-31 | 6.81E-30 | up |
| Zhx1 | 1.23940851 | 2.71E-31 | 7.53E-30 | up |
| B3gnt7 | 3.47293887 | 3.54E-31 | 9.79E-30 | up |
| Fanca | 2.247386742 | 3.77E-31 | 1.04E-29 | up |
| Ccne1 | 2.228927338 | 3.95E-31 | 1.09E-29 | up |
| Impa2 | 2.187806247 | 4.80E-31 | 1.32E-29 | up |
| Rasgef1b | 1.932310536 | 1.03E-30 | 2.79E-29 | up |
| Fndc3c1 | 6.860695313 | 1.18E-30 | 3.19E-29 | up |
| Elovl2 | 1.977741472 | 1.18E-30 | 3.19E-29 | up |
| D930015E06Rik | 1.209247267 | 1.23E-30 | 3.32E-29 | up |
| Stk32a | 3.297015384 | 1.86E-30 | 4.97E-29 | up |
| Rnd2 | 2.213739983 | 1.92E-30 | 5.10E-29 | up |
| Abcc4 | 1.559213509 | 2.19E-30 | 5.81E-29 | up |
| Mfsd4 | 1.82403823 | 2.88E-30 | 7.59E-29 | up |
| Uroc1 | 4.829274446 | 2.98E-30 | 7.85E-29 | up |
| P2ry2 | 1.976277291 | 4.33E-30 | 1.14E-28 | up |
| Dcun1d4 | 1.255254579 | 4.85E-30 | 1.27E-28 | up |
| St3gal2 | 1.559867265 | 7.44E-30 | 1.94E-28 | up |
| Tbl2 | 1.130557954 | 9.00E-30 | 2.34E-28 | up |
| Plac8 | 2.244696451 | 1.06E-29 | 2.74E-28 | up |
| Adarb2 | 7.510005714 | 1.46E-29 | 3.74E-28 | up |
| Slc35g1 | 2.184654094 | 1.46E-29 | 3.74E-28 | up |
| Clec2l | 2.199968068 | 1.47E-29 | 3.77E-28 | up |
| Otof | 4.349611237 | 1.81E-29 | 4.64E-28 | up |
| Rprm | 3.55308049 | 1.88E-29 | 4.79E-28 | up |
| Aqp1 | 2.215236246 | 1.94E-29 | 4.93E-28 | up |
| 9230105E05Rik | 5.163606557 | 2.00E-29 | 5.07E-28 | up |
| Bche | 3.22712087 | 2.39E-29 | 6.03E-28 | up |
| Wisp2 | 2.009729176 | 2.58E-29 | 6.51E-28 | up |
| Efcab6 | 3.634722336 | 2.96E-29 | 7.43E-28 | up |
| Gba | 1.158802685 | 3.25E-29 | 8.15E-28 | up |
| Mpped1 | 7.688557554 | 3.29E-29 | 8.21E-28 | up |
| Mbtd1 | 1.031195551 | 4.84E-29 | 1.19E-27 | up |
| Cacna1b | 3.911782611 | 5.33E-29 | 1.31E-27 | up |
| Adrb2 | 1.724065167 | 6.25E-29 | 1.52E-27 | up |
| Wdr46 | 1.11571103 | 7.84E-29 | 1.90E-27 | up |
| Rrp12 | 1.629333862 | 8.81E-29 | 2.13E-27 | up |
| Chrdl1 | 2.044442966 | 1.27E-28 | 3.05E-27 | up |
| Nptx1 | 2.112275552 | 1.28E-28 | 3.08E-27 | up |
| 1190003K10Rik | 7.762073604 | 1.30E-28 | 3.11E-27 | up |
| Grm4 | 7.324816235 | 1.31E-28 | 3.14E-27 | up |
| Cldn16 | 6.168360314 | 1.39E-28 | 3.30E-27 | up |
| Scara5 | 1.499823067 | 1.51E-28 | 3.58E-27 | up |
| Nadkd1 | 1.235558455 | 1.59E-28 | 3.77E-27 | up |
| Avpr1a | 3.492573271 | 1.76E-28 | 4.13E-27 | up |
| Nr5a1 | 2.39516319 | 1.98E-28 | 4.66E-27 | up |
| 1-Mar | 4.120269596 | 2.16E-28 | 5.06E-27 | up |
| Hsd17b4 | 1.024393904 | 3.28E-28 | 7.65E-27 | up |
| C430049B03Rik | 2.286980715 | 3.49E-28 | 8.13E-27 | up |
| Dnase1l2 | 2.031113982 | 5.79E-28 | 1.34E-26 | up |
| Lipg | 4.31065585 | 7.80E-28 | 1.79E-26 | up |
| Acot12 | 4.065698649 | 8.54E-28 | 1.95E-26 | up |
| Sdk1 | 2.823160648 | 9.18E-28 | 2.09E-26 | up |
| Etnk2 | 1.45898553 | 9.67E-28 | 2.19E-26 | up |
| Aldh3b2 | 3.368441996 | 1.24E-27 | 2.81E-26 | up |
| Gpr20 | 3.320366692 | 1.44E-27 | 3.25E-26 | up |
| Csrnp3 | 3.914790959 | 1.78E-27 | 3.99E-26 | up |
| Cdkn2b | 1.432385594 | 2.33E-27 | 5.22E-26 | up |
| Aldh4a1 | 1.17496295 | 2.47E-27 | 5.50E-26 | up |
| Bckdhb | 1.280069854 | 3.19E-27 | 7.07E-26 | up |
| Slc13a5 | 2.884135424 | 3.49E-27 | 7.73E-26 | up |
| Trub1 | 1.440499353 | 3.70E-27 | 8.19E-26 | up |
| Slc41a2 | 1.248063517 | 3.81E-27 | 8.41E-26 | up |
| Calml3 | 1.843944513 | 3.89E-27 | 8.57E-26 | up |
| Nrk | 7.236680139 | 3.96E-27 | 8.71E-26 | up |
| 3110009F21Rik | 7.440244053 | 4.76E-27 | 1.04E-25 | up |
| Sdr16c6 | 6.532644839 | 4.80E-27 | 1.05E-25 | up |
| Mn1 | 2.039219286 | 5.85E-27 | 1.28E-25 | up |
| Satb2 | 2.643528466 | 6.59E-27 | 1.43E-25 | up |
| Noc3l | 1.353243675 | 6.76E-27 | 1.47E-25 | up |
| Zfp711 | 3.760599671 | 1.00E-26 | 2.17E-25 | up |
| Hsd11b2 | 2.312691218 | 1.60E-26 | 3.41E-25 | up |
| 9930013L23Rik | 4.724609665 | 1.65E-26 | 3.51E-25 | up |
| Frmpd4 | 3.132100585 | 1.74E-26 | 3.69E-25 | up |
| Dpf3 | 2.210477771 | 1.79E-26 | 3.81E-25 | up |
| Lamb1 | 1.326656112 | 2.57E-26 | 5.42E-25 | up |
| Psapl1 | 3.774797686 | 2.92E-26 | 6.15E-25 | up |
| Cst8 | 4.435258886 | 2.96E-26 | 6.22E-25 | up |
| Auts2 | 1.746752868 | 3.49E-26 | 7.30E-25 | up |
| Prr5 | 1.600407736 | 3.58E-26 | 7.49E-25 | up |
| 2410018M08Rik | 2.149089976 | 3.80E-26 | 7.91E-25 | up |
| Sh3rf3 | 2.321151544 | 5.37E-26 | 1.10E-24 | up |
| Timm8a1 | 1.1735951 | 5.50E-26 | 1.13E-24 | up |
| Folh1 | 5.394591592 | 6.19E-26 | 1.27E-24 | up |
| C1qtnf3 | 4.192943396 | 7.11E-26 | 1.45E-24 | up |
| Pld6 | 3.671365945 | 7.80E-26 | 1.59E-24 | up |
| BC048679 | 6.784097531 | 8.55E-26 | 1.74E-24 | up |
| Cngb1 | 1.918151675 | 1.04E-25 | 2.11E-24 | up |
| Hcrtr1 | 4.568600467 | 1.07E-25 | 2.16E-24 | up |
| Nkd2 | 1.669164148 | 1.08E-25 | 2.18E-24 | up |
| Ces1g | 4.078686088 | 1.10E-25 | 2.21E-24 | up |
| Dennd5b | 1.506281895 | 1.39E-25 | 2.79E-24 | up |
| Mex3a | 1.648992654 | 1.47E-25 | 2.96E-24 | up |
| Cst12 | 4.264439238 | 2.13E-25 | 4.25E-24 | up |
| 1700024P16Rik | 1.634865849 | 2.14E-25 | 4.26E-24 | up |
| Fcrl1 | 3.950218236 | 2.22E-25 | 4.41E-24 | up |
| Plagl1 | 1.398615079 | 2.68E-25 | 5.29E-24 | up |
| Gpa33 | 4.392463729 | 3.03E-25 | 5.96E-24 | up |
| Cxcl12 | 1.875498911 | 3.07E-25 | 6.03E-24 | up |
| Pde3b | 1.677009474 | 3.29E-25 | 6.46E-24 | up |
| Ly6k | 3.016899933 | 3.30E-25 | 6.48E-24 | up |
| Cox10 | 1.186754734 | 3.57E-25 | 6.99E-24 | up |
| Podn | 1.714979267 | 4.40E-25 | 8.59E-24 | up |
| Ube2q2 | 1.083806485 | 4.43E-25 | 8.63E-24 | up |
| Flrt1 | 2.248242277 | 4.78E-25 | 9.28E-24 | up |
| Acat3 | 3.716949582 | 4.84E-25 | 9.38E-24 | up |
| Sftpb | 4.337394362 | 5.31E-25 | 1.02E-23 | up |
| Acads | 1.128317619 | 5.92E-25 | 1.14E-23 | up |
| Shh | 8.622669346 | 5.93E-25 | 1.14E-23 | up |
| Isyna1 | 1.03565611 | 6.60E-25 | 1.26E-23 | up |
| Scarb1 | 1.120439025 | 6.78E-25 | 1.30E-23 | up |
| 9230112J17Rik | 4.201408994 | 7.24E-25 | 1.38E-23 | up |
| Cd320 | 1.060280414 | 7.30E-25 | 1.39E-23 | up |
| Zxdb | 1.505764555 | 7.35E-25 | 1.40E-23 | up |
| Arnt2 | 3.229712988 | 7.53E-25 | 1.43E-23 | up |
| Cd79b | 1.812220373 | 8.34E-25 | 1.58E-23 | up |
| Ednrb | 1.61609505 | 1.25E-24 | 2.34E-23 | up |
| Otub2 | 1.319436832 | 1.26E-24 | 2.35E-23 | up |
| Spryd4 | 1.11394015 | 1.34E-24 | 2.51E-23 | up |
| Klf16 | 1.078107276 | 1.56E-24 | 2.90E-23 | up |
| Ankhd1 | 1.259556722 | 1.72E-24 | 3.19E-23 | up |
| Edil3 | 4.215206008 | 1.75E-24 | 3.24E-23 | up |
| 1110038B12Rik | 1.350922338 | 2.45E-24 | 4.51E-23 | up |
| Dgat2 | 1.766051738 | 2.55E-24 | 4.70E-23 | up |
| Katnb1 | 1.048291931 | 2.72E-24 | 4.98E-23 | up |
| Trmt61a | 1.188375104 | 3.20E-24 | 5.85E-23 | up |
| AI464131 | 1.061405532 | 4.28E-24 | 7.77E-23 | up |
| Slc22a21 | 2.221894462 | 4.41E-24 | 7.99E-23 | up |
| Fkbp5 | 1.443316056 | 4.56E-24 | 8.25E-23 | up |
| Necab1 | 3.247925344 | 4.96E-24 | 8.92E-23 | up |
| Pabpc1 | 1.315606068 | 5.06E-24 | 9.08E-23 | up |
| Unc5b | 1.592136449 | 5.72E-24 | 1.02E-22 | up |
| Mfap3l | 1.129299418 | 7.08E-24 | 1.27E-22 | up |
| Upk1a | 3.604430635 | 7.98E-24 | 1.42E-22 | up |
| Snhg6 | 1.7676844 | 8.12E-24 | 1.45E-22 | up |
| Adam12 | 1.740363935 | 8.88E-24 | 1.58E-22 | up |
| Vkorc1l1 | 1.27662066 | 1.17E-23 | 2.08E-22 | up |
| Dpy19l3 | 1.647344411 | 1.21E-23 | 2.14E-22 | up |
| Acsl5 | 1.192376595 | 1.26E-23 | 2.23E-22 | up |
| Mthfr | 1.047419146 | 1.45E-23 | 2.56E-22 | up |
| 1700019D03Rik | 2.688333294 | 1.73E-23 | 3.03E-22 | up |
| St8sia6 | 3.244834179 | 1.83E-23 | 3.19E-22 | up |
| Angpt2 | 2.584770809 | 3.23E-23 | 5.58E-22 | up |
| Fmod | 2.829607243 | 3.75E-23 | 6.45E-22 | up |
| Clcn6 | 1.478800565 | 3.81E-23 | 6.56E-22 | up |
| Vnn1 | 3.218362612 | 3.84E-23 | 6.60E-22 | up |
| Lrrk2 | 1.709511645 | 4.16E-23 | 7.13E-22 | up |
| Nhp2 | 1.121775992 | 4.43E-23 | 7.59E-22 | up |
| Slc2a6 | 2.317067678 | 5.08E-23 | 8.67E-22 | up |
| Cyp27b1 | 4.081788694 | 5.53E-23 | 9.42E-22 | up |
| Slc4a7 | 1.182903409 | 6.23E-23 | 1.06E-21 | up |
| Tfap2c | 1.760844922 | 6.91E-23 | 1.16E-21 | up |
| Oxtr | 3.810072491 | 6.93E-23 | 1.17E-21 | up |
| Stxbp1 | 1.404381611 | 7.31E-23 | 1.23E-21 | up |
| Adamtsl2 | 2.165966677 | 1.07E-22 | 1.79E-21 | up |
| Pgm5 | 2.642800421 | 1.08E-22 | 1.80E-21 | up |
| Otogl | 3.543399708 | 1.30E-22 | 2.16E-21 | up |
| Ppara | 1.441739002 | 1.32E-22 | 2.20E-21 | up |
| 2810032G03Rik | 3.32004816 | 1.50E-22 | 2.48E-21 | up |
| Fam163a | 3.39915574 | 1.54E-22 | 2.55E-21 | up |
| D8Ertd82e | 1.616883984 | 1.70E-22 | 2.80E-21 | up |
| Usp46 | 1.323982003 | 1.77E-22 | 2.91E-21 | up |
| Ap1s2 | 1.368809177 | 1.89E-22 | 3.11E-21 | up |
| Slc6a19 | 1.934102985 | 2.18E-22 | 3.56E-21 | up |
| Ppp2r2b | 4.04369901 | 2.44E-22 | 3.96E-21 | up |
| Camk2n1 | 1.646965512 | 2.51E-22 | 4.07E-21 | up |
| Wfdc8 | 7.12587629 | 2.75E-22 | 4.45E-21 | up |
| Ccdc86 | 1.086666975 | 2.84E-22 | 4.59E-21 | up |
| Obscn | 3.089306625 | 2.86E-22 | 4.60E-21 | up |
| B230312C02Rik | 6.889023424 | 3.15E-22 | 5.06E-21 | up |
| 4930555G01Rik | 5.074528034 | 3.45E-22 | 5.53E-21 | up |
| Rps6ka3 | 1.861918986 | 3.71E-22 | 5.93E-21 | up |
| Hist1h4i | 1.957240829 | 4.07E-22 | 6.50E-21 | up |
| Elac2 | 1.101181315 | 4.31E-22 | 6.86E-21 | up |
| Ncoa2 | 1.095289386 | 4.44E-22 | 7.05E-21 | up |
| Sbf1 | 1.14134302 | 4.94E-22 | 7.83E-21 | up |
| Dok6 | 6.082401311 | 5.00E-22 | 7.91E-21 | up |
| Mvd | 1.063110294 | 5.00E-22 | 7.91E-21 | up |
| 4931408D14Rik | 2.643335675 | 5.44E-22 | 8.58E-21 | up |
| Gucy2f | 3.770410573 | 5.62E-22 | 8.85E-21 | up |
| Atr | 1.099232669 | 5.74E-22 | 9.02E-21 | up |
| Tnfrsf18 | 1.970388705 | 6.79E-22 | 1.06E-20 | up |
| Cxcr4 | 1.908200307 | 7.73E-22 | 1.21E-20 | up |
| Slco4a1 | 3.544835142 | 8.14E-22 | 1.26E-20 | up |
| Pcdh7 | 1.489255995 | 8.14E-22 | 1.26E-20 | up |
| Ptprn2 | 3.77988405 | 8.74E-22 | 1.36E-20 | up |
| Rbm20 | 1.243498127 | 8.84E-22 | 1.37E-20 | up |
| Dnahc11 | 1.846222306 | 9.84E-22 | 1.52E-20 | up |
| Cry1 | 1.379507449 | 1.03E-21 | 1.60E-20 | up |
| Fam169a | 3.251024408 | 1.13E-21 | 1.74E-20 | up |
| Wdr16 | 1.444320721 | 1.20E-21 | 1.85E-20 | up |
| Ly6d | 4.057440098 | 1.26E-21 | 1.93E-20 | up |
| Dll4 | 1.512836239 | 1.35E-21 | 2.06E-20 | up |
| Tmem48 | 1.486852145 | 1.44E-21 | 2.19E-20 | up |
| Scn4a | 2.635933423 | 1.49E-21 | 2.27E-20 | up |
| Serpine1 | 2.103520753 | 1.71E-21 | 2.59E-20 | up |
| Epb4.9 | 1.051931451 | 1.71E-21 | 2.59E-20 | up |
| Sowaha | 1.547514897 | 1.96E-21 | 2.98E-20 | up |
| Park2 | 1.42078177 | 2.12E-21 | 3.21E-20 | up |
| Tmem139 | 1.561518313 | 2.13E-21 | 3.23E-20 | up |
| Cd3eap | 1.05329052 | 2.13E-21 | 3.23E-20 | up |
| Cdk18 | 1.205379444 | 2.15E-21 | 3.24E-20 | up |
| Med12l | 1.752873923 | 2.33E-21 | 3.50E-20 | up |
| Shq1 | 1.305377381 | 2.40E-21 | 3.60E-20 | up |
| Cmip | 1.058994068 | 2.75E-21 | 4.09E-20 | up |
| Homer1 | 1.083016299 | 2.75E-21 | 4.10E-20 | up |
| Acoxl | 3.126254952 | 2.92E-21 | 4.33E-20 | up |
| BC016423 | 1.254178381 | 2.92E-21 | 4.33E-20 | up |
| Eif4ebp1 | 1.072049319 | 3.01E-21 | 4.46E-20 | up |
| Klhl21 | 1.113771783 | 3.08E-21 | 4.56E-20 | up |
| Scn9a | 5.922959943 | 3.50E-21 | 5.15E-20 | up |
| Abcc3 | 2.215399393 | 3.71E-21 | 5.47E-20 | up |
| Rab40b | 1.822937463 | 3.76E-21 | 5.53E-20 | up |
| Ak4 | 2.491164171 | 4.04E-21 | 5.94E-20 | up |
| Col8a1 | 1.500803362 | 4.18E-21 | 6.13E-20 | up |
| Arhgap23 | 1.027893724 | 4.63E-21 | 6.79E-20 | up |
| Angptl3 | 3.981555821 | 6.16E-21 | 8.97E-20 | up |
| Tpsab1 | 4.276858957 | 6.39E-21 | 9.27E-20 | up |
| Cd163l1 | 3.234930641 | 6.65E-21 | 9.62E-20 | up |
| Cxx1c | 1.552536371 | 7.21E-21 | 1.04E-19 | up |
| Mycbpap | 1.252958333 | 7.32E-21 | 1.06E-19 | up |
| Fam174b | 2.009401222 | 9.46E-21 | 1.36E-19 | up |
| Adamts17 | 3.287908412 | 9.51E-21 | 1.36E-19 | up |
| Eda | 1.817164477 | 1.03E-20 | 1.47E-19 | up |
| Obp2b | 4.890043685 | 1.04E-20 | 1.49E-19 | up |
| Acot3 | 5.267256761 | 1.22E-20 | 1.74E-19 | up |
| Mical2 | 1.303976175 | 1.59E-20 | 2.26E-19 | up |
| Sdk2 | 4.634502711 | 1.63E-20 | 2.31E-19 | up |
| Slc22a14 | 6.851959292 | 1.64E-20 | 2.32E-19 | up |
| Lrrtm1 | 7.576492349 | 1.75E-20 | 2.47E-19 | up |
| Spint4 | 6.849783057 | 1.79E-20 | 2.52E-19 | up |
| Bcl2l13 | 1.01611233 | 1.85E-20 | 2.60E-19 | up |
| Spock1 | 3.658415649 | 2.59E-20 | 3.63E-19 | up |
| 6430573F11Rik | 2.418263044 | 2.91E-20 | 4.07E-19 | up |
| Btc | 1.77444011 | 3.62E-20 | 5.03E-19 | up |
| Mfsd7b | 1.063416173 | 3.72E-20 | 5.15E-19 | up |
| Trim62 | 1.041087398 | 3.78E-20 | 5.23E-19 | up |
| Nars2 | 1.168064185 | 4.06E-20 | 5.60E-19 | up |
| Tenm2 | 4.641568689 | 4.67E-20 | 6.40E-19 | up |
| Tubb2b | 1.839389293 | 4.72E-20 | 6.46E-19 | up |
| Fam196b | 4.48855975 | 5.69E-20 | 7.76E-19 | up |
| Fbxo36 | 1.447617943 | 6.87E-20 | 9.33E-19 | up |
| Col4a3 | 1.245373033 | 7.78E-20 | 1.05E-18 | up |
| Dach2 | 2.748037543 | 8.62E-20 | 1.16E-18 | up |
| Wif1 | 4.890411426 | 9.91E-20 | 1.33E-18 | up |
| Ces1a | 3.596933452 | 1.05E-19 | 1.41E-18 | up |
| Gpihbp1 | 1.845446043 | 1.19E-19 | 1.60E-18 | up |
| Cgnl1 | 1.329764189 | 1.34E-19 | 1.80E-18 | up |
| Foxc1 | 1.858979184 | 1.64E-19 | 2.18E-18 | up |
| Slc1a4 | 1.609384223 | 2.09E-19 | 2.77E-18 | up |
| Lbh | 1.035857405 | 2.15E-19 | 2.85E-18 | up |
| Slc22a12 | 5.006215826 | 2.19E-19 | 2.89E-18 | up |
| Ffar4 | 3.928649393 | 2.28E-19 | 3.02E-18 | up |
| Fmo5 | 1.228947892 | 2.37E-19 | 3.13E-18 | up |
| Pou3f1 | 3.829721147 | 2.76E-19 | 3.63E-18 | up |
| Mdm1 | 1.279622047 | 2.81E-19 | 3.69E-18 | up |
| Esrp2 | 1.08720805 | 3.03E-19 | 3.97E-18 | up |
| Sult5a1 | 5.012604586 | 3.10E-19 | 4.06E-18 | up |
| Prkce | 1.131178605 | 3.64E-19 | 4.74E-18 | up |
| Fgl2 | 2.973054658 | 3.74E-19 | 4.85E-18 | up |
| D630029K05Rik | 4.06539408 | 3.98E-19 | 5.16E-18 | up |
| Nmt2 | 1.44416023 | 4.52E-19 | 5.81E-18 | up |
| Gnai1 | 1.252591199 | 4.67E-19 | 5.98E-18 | up |
| Efr3b | 1.266724451 | 4.76E-19 | 6.08E-18 | up |
| Klhdc8a | 1.213381567 | 4.78E-19 | 6.10E-18 | up |
| Mkx | 3.907756807 | 4.99E-19 | 6.37E-18 | up |
| Prr15 | 1.173484758 | 5.24E-19 | 6.67E-18 | up |
| Gjb3 | 2.051758563 | 5.52E-19 | 7.00E-18 | up |
| Pinx1 | 1.09825666 | 5.64E-19 | 7.14E-18 | up |
| Kitl | 2.251291036 | 5.69E-19 | 7.19E-18 | up |
| Olfm2 | 3.91439924 | 6.02E-19 | 7.60E-18 | up |
| Aspg | 1.79886233 | 8.70E-19 | 1.09E-17 | up |
| Prdm14 | 7.541351474 | 1.00E-18 | 1.25E-17 | up |
| A330021E22Rik | 2.102066238 | 1.15E-18 | 1.44E-17 | up |
| Lpin1 | 1.037362749 | 1.27E-18 | 1.58E-17 | up |
| Aadat | 4.027892286 | 1.30E-18 | 1.61E-17 | up |
| Tmem8c | 4.169950811 | 1.45E-18 | 1.79E-17 | up |
| Usp31 | 1.095491545 | 1.59E-18 | 1.96E-17 | up |
| Slc15a5 | 3.780218231 | 2.13E-18 | 2.61E-17 | up |
| Wnt5a | 1.132039402 | 2.14E-18 | 2.61E-17 | up |
| Ednra | 1.666144169 | 2.17E-18 | 2.64E-17 | up |
| Lpgat1 | 1.600470851 | 2.36E-18 | 2.87E-17 | up |
| Sema3a | 2.994797337 | 2.40E-18 | 2.90E-17 | up |
| Magi2 | 2.126086928 | 2.72E-18 | 3.28E-17 | up |
| Trpv6 | 3.318931031 | 2.75E-18 | 3.32E-17 | up |
| Col8a2 | 2.138325226 | 2.97E-18 | 3.57E-17 | up |
| Pik3ap1 | 2.094412364 | 3.03E-18 | 3.63E-17 | up |
| Tbc1d30 | 2.408401612 | 3.07E-18 | 3.68E-17 | up |
| Gm5126 | 3.319137738 | 3.24E-18 | 3.88E-17 | up |
| Tmem97 | 1.18690882 | 4.15E-18 | 4.93E-17 | up |
| Col10a1 | 3.64914184 | 4.26E-18 | 5.05E-17 | up |
| Aim1l | 3.207620727 | 4.33E-18 | 5.14E-17 | up |
| Dhcr7 | 1.299788506 | 5.65E-18 | 6.66E-17 | up |
| Sfxn2 | 1.956943795 | 5.89E-18 | 6.92E-17 | up |
| Mmachc | 1.012412169 | 5.90E-18 | 6.93E-17 | up |
| Phyh | 1.085951865 | 6.18E-18 | 7.23E-17 | up |
| Rnf150 | 1.146794323 | 6.86E-18 | 8.00E-17 | up |
| Prex2 | 1.204168817 | 8.04E-18 | 9.32E-17 | up |
| Ank3 | 1.662984389 | 9.72E-18 | 1.13E-16 | up |
| Pdk1 | 1.238566229 | 9.82E-18 | 1.14E-16 | up |
| Muc1 | 2.874730357 | 1.01E-17 | 1.17E-16 | up |
| Nov | 1.500610092 | 1.17E-17 | 1.35E-16 | up |
| Dio3 | 2.341699549 | 1.30E-17 | 1.49E-16 | up |
| Fam60a | 1.585426639 | 1.31E-17 | 1.51E-16 | up |
| Enc1 | 1.167062904 | 1.37E-17 | 1.57E-16 | up |
| Cntn2 | 4.114122255 | 1.83E-17 | 2.08E-16 | up |
| Hesx1 | 4.679974814 | 1.84E-17 | 2.09E-16 | up |
| Pcdh17 | 1.892721718 | 1.84E-17 | 2.09E-16 | up |
| AK129341 | 1.191360629 | 1.85E-17 | 2.10E-16 | up |
| Timm10 | 1.245258558 | 1.93E-17 | 2.19E-16 | up |
| Vav2 | 1.248042726 | 2.05E-17 | 2.31E-16 | up |
| Cdkn2a | 2.243912731 | 2.33E-17 | 2.62E-16 | up |
| Tmem132e | 5.257747263 | 2.66E-17 | 2.97E-16 | up |
| Zscan18 | 1.556230635 | 2.70E-17 | 3.01E-16 | up |
| Cym | 7.287334683 | 2.77E-17 | 3.08E-16 | up |
| Zw10 | 1.035004441 | 2.87E-17 | 3.19E-16 | up |
| Camkk1 | 1.226174611 | 2.91E-17 | 3.23E-16 | up |
| Utp20 | 1.029042025 | 3.73E-17 | 4.12E-16 | up |
| Tekt1 | 2.485851785 | 4.18E-17 | 4.60E-16 | up |
| Dst | 1.104379907 | 4.57E-17 | 4.98E-16 | up |
| Cytip | 1.149687376 | 4.60E-17 | 5.01E-16 | up |
| Adamts4 | 3.320881016 | 5.26E-17 | 5.71E-16 | up |
| Maml3 | 1.236392276 | 5.42E-17 | 5.87E-16 | up |
| Thbs3 | 1.515168242 | 6.95E-17 | 7.47E-16 | up |
| Paqr5 | 2.118333138 | 8.04E-17 | 8.58E-16 | up |
| 1700031F05Rik | 6.667329014 | 9.22E-17 | 9.81E-16 | up |
| Celf4 | 1.779597128 | 9.72E-17 | 1.03E-15 | up |
| Myo1b | 1.208799611 | 1.04E-16 | 1.10E-15 | up |
| Amt | 1.064034701 | 1.08E-16 | 1.14E-15 | up |
| Rspo2 | 4.399115107 | 1.21E-16 | 1.27E-15 | up |
| Olfml2b | 1.458661292 | 1.27E-16 | 1.33E-15 | up |
| Morc4 | 1.212156484 | 1.61E-16 | 1.68E-15 | up |
| Urb1 | 1.003737389 | 1.84E-16 | 1.91E-15 | up |
| Aplnr | 1.303091795 | 1.85E-16 | 1.92E-15 | up |
| Sulf2 | 1.022452609 | 1.99E-16 | 2.06E-15 | up |
| Ttc7b | 1.004680208 | 2.12E-16 | 2.19E-15 | up |
| Cdca7 | 1.537303149 | 2.28E-16 | 2.35E-15 | up |
| Naa25 | 1.207151643 | 2.44E-16 | 2.51E-15 | up |
| Bckdha | 1.051889854 | 2.52E-16 | 2.58E-15 | up |
| Iba57 | 1.056407206 | 2.59E-16 | 2.66E-15 | up |
| 5430425J12Rik | 2.559534332 | 2.60E-16 | 2.67E-15 | up |
| Gng4 | 5.199156063 | 2.85E-16 | 2.91E-15 | up |
| Piga | 1.30763355 | 2.85E-16 | 2.91E-15 | up |
| Apold1 | 1.578088944 | 2.87E-16 | 2.93E-15 | up |
| Slc2a3 | 1.684624477 | 2.99E-16 | 3.04E-15 | up |
| Ar | 2.617280748 | 3.03E-16 | 3.08E-15 | up |
| Tspan1 | 1.36992494 | 3.51E-16 | 3.56E-15 | up |
| Tnfrsf11b | 2.322709801 | 3.72E-16 | 3.76E-15 | up |
| Immp2l | 1.833289277 | 3.97E-16 | 4.01E-15 | up |
| Fst | 2.649841053 | 4.49E-16 | 4.51E-15 | up |
| Bcat1 | 1.065022436 | 5.00E-16 | 5.00E-15 | up |
| Prkch | 1.093321244 | 5.77E-16 | 5.74E-15 | up |
| Cd93 | 1.576578423 | 6.11E-16 | 6.06E-15 | up |
| Sema4g | 2.753448483 | 7.08E-16 | 7.01E-15 | up |
| Dchs1 | 1.419566757 | 7.34E-16 | 7.26E-15 | up |
| Rnf152 | 1.66671879 | 7.60E-16 | 7.50E-15 | up |
| Rnf144a | 1.231162811 | 8.11E-16 | 7.99E-15 | up |
| Plin5 | 1.718328686 | 8.70E-16 | 8.56E-15 | up |
| 9230104L09Rik | 6.387411065 | 1.07E-15 | 1.04E-14 | up |
| Frat2 | 1.409900915 | 1.22E-15 | 1.19E-14 | up |
| Sox5 | 1.053441903 | 1.22E-15 | 1.19E-14 | up |
| Maff | 1.394567813 | 1.35E-15 | 1.30E-14 | up |
| Sned1 | 1.291736619 | 1.36E-15 | 1.31E-14 | up |
| Sema3d | 2.355936925 | 1.39E-15 | 1.34E-14 | up |
| Ptx3 | 2.937247283 | 1.78E-15 | 1.70E-14 | up |
| Bsn | 1.750496158 | 1.78E-15 | 1.70E-14 | up |
| Nes | 1.203987689 | 1.78E-15 | 1.70E-14 | up |
| Thbs2 | 1.664435423 | 2.21E-15 | 2.10E-14 | up |
| Zfp941 | 1.749238453 | 2.29E-15 | 2.17E-14 | up |
| Atp8a2 | 3.066511565 | 2.34E-15 | 2.21E-14 | up |
| Adtrp | 1.114003774 | 2.38E-15 | 2.25E-14 | up |
| Angptl4 | 1.525047628 | 2.57E-15 | 2.42E-14 | up |
| Ano3 | 2.554685184 | 3.01E-15 | 2.82E-14 | up |
| Gata6 | 2.052910351 | 3.03E-15 | 2.83E-14 | up |
| Dpf1 | 2.905993538 | 3.16E-15 | 2.95E-14 | up |
| Fbxo27 | 2.334550069 | 3.58E-15 | 3.32E-14 | up |
| Stab2 | 3.754604983 | 3.59E-15 | 3.33E-14 | up |
| Lrrc16b | 2.116937263 | 3.67E-15 | 3.40E-14 | up |
| Myom3 | 3.24208639 | 3.86E-15 | 3.57E-14 | up |
| Uba6 | 1.289879899 | 4.10E-15 | 3.79E-14 | up |
| Kcns3 | 1.525024695 | 5.11E-15 | 4.68E-14 | up |
| Igfbp3 | 1.716872412 | 5.47E-15 | 4.99E-14 | up |
| Morn1 | 1.178219656 | 5.61E-15 | 5.11E-14 | up |
| Sh3tc1 | 1.178852801 | 5.62E-15 | 5.12E-14 | up |
| Cxcr7 | 1.324667335 | 5.80E-15 | 5.27E-14 | up |
| Ttc22 | 2.395993496 | 6.13E-15 | 5.57E-14 | up |
| Hhat | 1.399149562 | 6.18E-15 | 5.61E-14 | up |
| Zfp558 | 1.492011789 | 7.12E-15 | 6.43E-14 | up |
| Phospho1 | 1.946848006 | 7.48E-15 | 6.74E-14 | up |
| D030025P21Rik | 2.953358341 | 7.62E-15 | 6.85E-14 | up |
| Sh2d5 | 2.501349553 | 7.84E-15 | 7.04E-14 | up |
| Npy | 3.728494627 | 7.94E-15 | 7.12E-14 | up |
| Pyroxd2 | 1.371103352 | 8.43E-15 | 7.55E-14 | up |
| Sdcbp2 | 2.314499796 | 9.23E-15 | 8.22E-14 | up |
| Zfp444 | 1.047100557 | 9.63E-15 | 8.54E-14 | up |
| Dnajb3 | 2.506683367 | 1.06E-14 | 9.36E-14 | up |
| Pcx | 1.028119804 | 1.15E-14 | 1.01E-13 | up |
| Cntn3 | 3.826142165 | 1.23E-14 | 1.07E-13 | up |
| Ano1 | 1.111019093 | 1.27E-14 | 1.11E-13 | up |
| Hbb-bh1 | 7.752126911 | 1.35E-14 | 1.17E-13 | up |
| Hhipl1 | 2.386531697 | 1.36E-14 | 1.18E-13 | up |
| Slc16a12 | 2.065687058 | 1.53E-14 | 1.33E-13 | up |
| Suv39h2 | 1.470074725 | 1.67E-14 | 1.45E-13 | up |
| Gja1 | 1.172558478 | 1.82E-14 | 1.58E-13 | up |
| Bnc2 | 3.133671154 | 1.92E-14 | 1.66E-13 | up |
| A730090H04Rik | 4.578860596 | 2.05E-14 | 1.76E-13 | up |
| Vash2 | 1.421556283 | 2.23E-14 | 1.91E-13 | up |
| Slc25a10 | 1.011532538 | 2.41E-14 | 2.06E-13 | up |
| Aldh1l2 | 2.287950917 | 2.45E-14 | 2.10E-13 | up |
| Itpka | 2.017497593 | 2.69E-14 | 2.29E-13 | up |
| Fscn1 | 1.162398988 | 2.95E-14 | 2.51E-13 | up |
| Fras1 | 1.193715777 | 3.14E-14 | 2.67E-13 | up |
| Ppp1r14c | 2.11894523 | 3.30E-14 | 2.80E-13 | up |
| Nudt12 | 1.218323651 | 3.37E-14 | 2.85E-13 | up |
| Xkr6 | 3.228325467 | 3.39E-14 | 2.86E-13 | up |
| Ttc39c | 1.334141024 | 3.52E-14 | 2.97E-13 | up |
| D830030K20Rik | 6.581185893 | 3.58E-14 | 3.02E-13 | up |
| Mdga1 | 1.308656333 | 3.66E-14 | 3.08E-13 | up |
| Nhsl2 | 1.488818897 | 3.94E-14 | 3.32E-13 | up |
| Nkg7 | 4.296742431 | 4.36E-14 | 3.65E-13 | up |
| Rasal1 | 2.164621545 | 4.84E-14 | 4.04E-13 | up |
| Sdpr | 1.094861061 | 5.10E-14 | 4.25E-13 | up |
| Ppargc1b | 1.971702398 | 5.44E-14 | 4.52E-13 | up |
| Grem2 | 2.687778431 | 5.73E-14 | 4.75E-13 | up |
| Fam195a | 1.392420693 | 5.80E-14 | 4.81E-13 | up |
| Hdx | 3.601755811 | 5.85E-14 | 4.84E-13 | up |
| Krt25 | 3.885627023 | 6.42E-14 | 5.30E-13 | up |
| Auh | 1.018933154 | 7.01E-14 | 5.76E-13 | up |
| Guca1a | 1.846045549 | 7.87E-14 | 6.46E-13 | up |
| Lypd1 | 3.26976978 | 8.77E-14 | 7.17E-13 | up |
| Wfdc13 | 5.882463101 | 9.27E-14 | 7.55E-13 | up |
| Nav3 | 2.511237316 | 1.28E-13 | 1.03E-12 | up |
| Nlgn3 | 2.80692357 | 1.30E-13 | 1.05E-12 | up |
| Vsnl1 | 2.389083103 | 1.40E-13 | 1.13E-12 | up |
| Aph1c | 1.142927516 | 1.40E-13 | 1.13E-12 | up |
| Epb4.1l4a | 1.134732337 | 1.48E-13 | 1.19E-12 | up |
| Fcgr2b | 1.085416451 | 1.50E-13 | 1.21E-12 | up |
| Adhfe1 | 1.533072424 | 1.58E-13 | 1.27E-12 | up |
| Cyp2r1 | 1.965964659 | 1.59E-13 | 1.27E-12 | up |
| Notch4 | 1.036451041 | 1.62E-13 | 1.30E-12 | up |
| Dapk2 | 2.046895851 | 1.70E-13 | 1.36E-12 | up |
| Grhl3 | 2.143622403 | 1.77E-13 | 1.41E-12 | up |
| Gcdh | 1.118747829 | 1.99E-13 | 1.59E-12 | up |
| Slc7a5 | 1.927000888 | 2.55E-13 | 2.02E-12 | up |
| Foxo6 | 1.275014459 | 2.58E-13 | 2.04E-12 | up |
| Slc18a1 | 1.415235677 | 2.67E-13 | 2.11E-12 | up |
| Slc26a2 | 1.503780266 | 2.71E-13 | 2.14E-12 | up |
| Evi5l | 1.150914071 | 2.79E-13 | 2.20E-12 | up |
| Adcy1 | 2.832799887 | 2.80E-13 | 2.21E-12 | up |
| Zfpm1 | 1.040397008 | 3.10E-13 | 2.42E-12 | up |
| Rundc3b | 2.179811786 | 3.15E-13 | 2.46E-12 | up |
| Lox | 1.839642916 | 3.33E-13 | 2.60E-12 | up |
| Igfbp7 | 1.104226129 | 3.59E-13 | 2.79E-12 | up |
| Mettl7b | 4.260601962 | 4.22E-13 | 3.26E-12 | up |
| Ephb1 | 1.564708557 | 4.37E-13 | 3.37E-12 | up |
| Gpc6 | 1.48366797 | 4.78E-13 | 3.67E-12 | up |
| Gm11837 | 1.957953069 | 5.26E-13 | 4.03E-12 | up |
| Mbnl3 | 1.140661248 | 5.26E-13 | 4.03E-12 | up |
| Arntl2 | 1.568760198 | 5.85E-13 | 4.47E-12 | up |
| Nudt4 | 1.164156571 | 6.02E-13 | 4.59E-12 | up |
| Cth | 2.748776113 | 6.34E-13 | 4.83E-12 | up |
| Zpbp2 | 2.879734229 | 6.48E-13 | 4.93E-12 | up |
| Ranbp17 | 1.484465777 | 7.14E-13 | 5.41E-12 | up |
| Pcdh12 | 1.270745991 | 7.30E-13 | 5.51E-12 | up |
| B3gnt4 | 2.303899288 | 7.52E-13 | 5.67E-12 | up |
| Folr1 | 1.583972261 | 8.59E-13 | 6.45E-12 | up |
| Paox | 1.285532888 | 8.93E-13 | 6.70E-12 | up |
| Lamc3 | 1.81628445 | 9.53E-13 | 7.13E-12 | up |
| Muc6 | 2.878602505 | 9.93E-13 | 7.40E-12 | up |
| Slc26a9 | 2.638368403 | 1.18E-12 | 8.74E-12 | up |
| Cntn1 | 3.465146383 | 1.20E-12 | 8.88E-12 | up |
| Spin2 | 1.408269328 | 1.29E-12 | 9.55E-12 | up |
| Sh3pxd2a | 1.10782679 | 1.30E-12 | 9.62E-12 | up |
| Anxa8 | 1.647029552 | 1.34E-12 | 9.91E-12 | up |
| Mab21l2 | 2.668003248 | 1.45E-12 | 1.07E-11 | up |
| Trpc3 | 1.384655887 | 1.51E-12 | 1.11E-11 | up |
| 4921507P07Rik | 1.730762925 | 1.68E-12 | 1.23E-11 | up |
| Mgmt | 1.433448404 | 1.86E-12 | 1.36E-11 | up |
| Dhtkd1 | 2.13106003 | 1.95E-12 | 1.42E-11 | up |
| Ptp4a3 | 1.344433769 | 1.96E-12 | 1.43E-11 | up |
| Neto2 | 2.387751919 | 2.01E-12 | 1.46E-11 | up |
| E2f8 | 1.649663576 | 2.11E-12 | 1.53E-11 | up |
| Flt4 | 1.09184644 | 2.17E-12 | 1.57E-11 | up |
| Cd36 | 1.930403374 | 2.26E-12 | 1.64E-11 | up |
| Pde4c | 2.071374989 | 2.34E-12 | 1.69E-11 | up |
| Acot4 | 1.967447207 | 2.34E-12 | 1.69E-11 | up |
| Stc1 | 1.473877686 | 2.51E-12 | 1.80E-11 | up |
| Ube2ql1 | 2.699908704 | 2.55E-12 | 1.83E-11 | up |
| Itga9 | 1.10946985 | 2.66E-12 | 1.91E-11 | up |
| Has1 | 2.874307011 | 2.81E-12 | 2.01E-11 | up |
| Asb5 | 1.301941298 | 3.01E-12 | 2.14E-11 | up |
| Atp2a3 | 1.825695859 | 3.02E-12 | 2.15E-11 | up |
| Bdnf | 2.150646151 | 3.13E-12 | 2.23E-11 | up |
| Asb9 | 1.583149394 | 3.13E-12 | 2.23E-11 | up |
| 4933406C10Rik | 2.169959206 | 3.15E-12 | 2.24E-11 | up |
| Padi2 | 1.625192737 | 3.25E-12 | 2.30E-11 | up |
| 1190003J15Rik | 1.662317856 | 3.41E-12 | 2.41E-11 | up |
| Spp1 | 2.154908592 | 3.48E-12 | 2.46E-11 | up |
| Raver2 | 1.094443396 | 3.50E-12 | 2.47E-11 | up |
| Qtrt1 | 1.122073124 | 3.55E-12 | 2.51E-11 | up |
| Ostn | 5.855687634 | 3.70E-12 | 2.60E-11 | up |
| Hyi | 1.164506192 | 3.85E-12 | 2.71E-11 | up |
| Loxl3 | 1.150067484 | 3.93E-12 | 2.76E-11 | up |
| Pcsk5 | 1.012191677 | 4.05E-12 | 2.85E-11 | up |
| Acaa1b | 2.32291254 | 4.17E-12 | 2.93E-11 | up |
| Kcnj8 | 1.344952172 | 4.46E-12 | 3.13E-11 | up |
| Cdh13 | 2.018710645 | 4.52E-12 | 3.17E-11 | up |
| Lrp8 | 1.857599673 | 4.65E-12 | 3.25E-11 | up |
| Dancr | 1.229258996 | 4.67E-12 | 3.26E-11 | up |
| Sgtb | 1.299684482 | 5.71E-12 | 3.96E-11 | up |
| Prss53 | 2.01279047 | 6.01E-12 | 4.16E-11 | up |
| Snx29 | 1.629789858 | 6.20E-12 | 4.27E-11 | up |
| 4930572O13Rik | 3.021963728 | 6.33E-12 | 4.36E-11 | up |
| Tert | 1.040410737 | 6.51E-12 | 4.47E-11 | up |
| Syt9 | 2.436511089 | 6.59E-12 | 4.52E-11 | up |
| Nrxn2 | 2.329710074 | 7.47E-12 | 5.11E-11 | up |
| Dnase1 | 3.192156806 | 7.57E-12 | 5.17E-11 | up |
| Cyp24a1 | 5.092537463 | 8.02E-12 | 5.47E-11 | up |
| Tbc1d9 | 1.515853019 | 8.05E-12 | 5.49E-11 | up |
| Pdss1 | 1.290378408 | 8.12E-12 | 5.53E-11 | up |
| Rgs9 | 1.477717506 | 8.52E-12 | 5.79E-11 | up |
| Cadps2 | 1.104683283 | 8.52E-12 | 5.79E-11 | up |
| Sox6 | 2.22640122 | 9.07E-12 | 6.14E-11 | up |
| Gm10451 | 2.750918128 | 1.00E-11 | 6.78E-11 | up |
| Sprr1a | 4.179449855 | 1.07E-11 | 7.22E-11 | up |
| Shroom4 | 1.749167259 | 1.23E-11 | 8.24E-11 | up |
| Doc2b | 2.918981885 | 1.29E-11 | 8.61E-11 | up |
| Ces2c | 5.007226276 | 1.29E-11 | 8.62E-11 | up |
| Col5a2 | 1.179926184 | 1.30E-11 | 8.69E-11 | up |
| Edar | 1.474877788 | 1.35E-11 | 9.01E-11 | up |
| Nrarp | 1.056076111 | 1.61E-11 | 1.06E-10 | up |
| Gm11992 | 2.344616794 | 1.68E-11 | 1.11E-10 | up |
| Nckap5 | 1.301156683 | 1.71E-11 | 1.13E-10 | up |
| Enpp6 | 2.087044881 | 1.76E-11 | 1.16E-10 | up |
| Cfh | 1.252020692 | 1.78E-11 | 1.17E-10 | up |
| Pitx1 | 2.591455953 | 1.94E-11 | 1.27E-10 | up |
| Dcxr | 1.036577485 | 2.18E-11 | 1.42E-10 | up |
| Ovol1 | 1.187232552 | 2.46E-11 | 1.60E-10 | up |
| Tnfsf11 | 3.706352902 | 2.57E-11 | 1.66E-10 | up |
| Steap2 | 2.14179237 | 2.58E-11 | 1.67E-10 | up |
| M1ap | 2.558469599 | 3.08E-11 | 1.98E-10 | up |
| Abcg1 | 1.120893834 | 3.14E-11 | 2.02E-10 | up |
| D630023F18Rik | 2.391185482 | 3.26E-11 | 2.09E-10 | up |
| Npas3 | 2.958240822 | 3.48E-11 | 2.23E-10 | up |
| Tepp | 1.210068521 | 4.28E-11 | 2.72E-10 | up |
| Uts2 | 3.833544639 | 4.32E-11 | 2.74E-10 | up |
| Pappa2 | 3.047538987 | 4.33E-11 | 2.75E-10 | up |
| Ros1 | 2.672076021 | 4.60E-11 | 2.91E-10 | up |
| Rnf180 | 1.675926244 | 4.66E-11 | 2.94E-10 | up |
| Abcb9 | 1.185368296 | 4.75E-11 | 2.99E-10 | up |
| Sepsecs | 1.370330547 | 5.34E-11 | 3.34E-10 | up |
| BC055324 | 1.394560889 | 5.78E-11 | 3.61E-10 | up |
| Stc2 | 1.108444392 | 5.95E-11 | 3.71E-10 | up |
| Scube1 | 1.367409503 | 6.16E-11 | 3.83E-10 | up |
| Add2 | 2.510182371 | 6.60E-11 | 4.10E-10 | up |
| Cklf | 1.066961964 | 6.71E-11 | 4.16E-10 | up |
| Elovl6 | 1.217734073 | 7.16E-11 | 4.42E-10 | up |
| Kif27 | 1.280953361 | 7.98E-11 | 4.91E-10 | up |
| Bcl6b | 1.480741521 | 8.04E-11 | 4.94E-10 | up |
| Xrcc3 | 1.019658185 | 9.50E-11 | 5.81E-10 | up |
| Gm15401 | 2.609551262 | 1.01E-10 | 6.17E-10 | up |
| Lrrc3 | 1.113934092 | 1.08E-10 | 6.58E-10 | up |
| Pvrl1 | 1.04682873 | 1.12E-10 | 6.79E-10 | up |
| Fat3 | 3.5595535 | 1.23E-10 | 7.42E-10 | up |
| Fbn2 | 2.800598285 | 1.38E-10 | 8.27E-10 | up |
| Nos2 | 1.878221928 | 1.44E-10 | 8.61E-10 | up |
| Epha3 | 2.536774023 | 1.53E-10 | 9.13E-10 | up |
| Plb1 | 1.925076684 | 1.54E-10 | 9.24E-10 | up |
| Mycl1 | 2.234912389 | 1.55E-10 | 9.25E-10 | up |
| Ppp1r12b | 1.258484985 | 1.65E-10 | 9.80E-10 | up |
| Clca5 | 2.914164223 | 1.68E-10 | 1.00E-09 | up |
| Papss2 | 1.002896898 | 1.77E-10 | 1.05E-09 | up |
| Kcnq1 | 1.378453238 | 1.82E-10 | 1.08E-09 | up |
| Cml2 | 2.23813741 | 1.86E-10 | 1.10E-09 | up |
| Gm17495 | 2.371639151 | 1.89E-10 | 1.12E-09 | up |
| Meis2 | 1.02762251 | 1.95E-10 | 1.15E-09 | up |
| Gm1976 | 1.229500231 | 1.97E-10 | 1.16E-09 | up |
| Cdh5 | 1.04722761 | 2.12E-10 | 1.24E-09 | up |
| Syt17 | 2.15656155 | 2.31E-10 | 1.35E-09 | up |
| Trpc6 | 1.646282935 | 2.41E-10 | 1.41E-09 | up |
| Rgs4 | 1.030012885 | 2.57E-10 | 1.50E-09 | up |
| Nqo1 | 1.339574049 | 2.64E-10 | 1.54E-09 | up |
| 9130206I24Rik | 1.666017242 | 2.73E-10 | 1.59E-09 | up |
| Il13ra2 | 3.650496686 | 2.76E-10 | 1.60E-09 | up |
| Gpr113 | 4.869375278 | 2.78E-10 | 1.62E-09 | up |
| Wnt9a | 1.807502205 | 2.93E-10 | 1.70E-09 | up |
| Nxph4 | 2.942481598 | 3.25E-10 | 1.87E-09 | up |
| Ablim2 | 1.339279747 | 3.49E-10 | 2.00E-09 | up |
| Ip6k3 | 2.035734197 | 3.66E-10 | 2.10E-09 | up |
| Crabp2 | 3.007598554 | 3.72E-10 | 2.13E-09 | up |
| Ung | 1.242207652 | 3.77E-10 | 2.16E-09 | up |
| C4b | 1.303480332 | 4.56E-10 | 2.59E-09 | up |
| Eln | 1.342368258 | 4.63E-10 | 2.63E-09 | up |
| Tmsb15a | 4.117790028 | 4.95E-10 | 2.80E-09 | up |
| Col22a1 | 3.195280515 | 5.41E-10 | 3.05E-09 | up |
| Ttbk1 | 2.729631832 | 5.49E-10 | 3.09E-09 | up |
| Kbtbd8 | 1.121052829 | 5.52E-10 | 3.11E-09 | up |
| Epb4.2 | 2.713596938 | 6.01E-10 | 3.37E-09 | up |
| 1500015L24Rik | 1.822903246 | 6.03E-10 | 3.38E-09 | up |
| Ahsg | 2.784183861 | 6.08E-10 | 3.40E-09 | up |
| Cldn5 | 1.664999168 | 6.16E-10 | 3.44E-09 | up |
| Slc25a21 | 1.614478957 | 6.57E-10 | 3.66E-09 | up |
| Gadl1 | 4.016929069 | 6.60E-10 | 3.68E-09 | up |
| Map3k15 | 1.560012216 | 6.64E-10 | 3.70E-09 | up |
| Gpr135 | 1.934866015 | 7.03E-10 | 3.91E-09 | up |
| Shank2 | 1.261350308 | 7.16E-10 | 3.98E-09 | up |
| Gatsl2 | 1.063473155 | 7.55E-10 | 4.18E-09 | up |
| Unc5d | 6.208203208 | 7.98E-10 | 4.39E-09 | up |
| Icam5 | 1.687541659 | 8.29E-10 | 4.55E-09 | up |
| Megf10 | 2.52979814 | 8.64E-10 | 4.73E-09 | up |
| Cpne7 | 2.058660435 | 8.90E-10 | 4.87E-09 | up |
| Zdhhc23 | 1.699832316 | 9.50E-10 | 5.18E-09 | up |
| Disp2 | 1.594670411 | 9.50E-10 | 5.18E-09 | up |
| Apln | 1.782148021 | 9.81E-10 | 5.34E-09 | up |
| Hist1h4h | 1.518629481 | 1.03E-09 | 5.59E-09 | up |
| Hsd17b2 | 1.751965711 | 1.09E-09 | 5.90E-09 | up |
| 9130024F11Rik | 2.804606637 | 1.09E-09 | 5.91E-09 | up |
| Xlr | 1.546744898 | 1.22E-09 | 6.60E-09 | up |
| Rsph4a | 2.068213253 | 1.34E-09 | 7.18E-09 | up |
| Sulf1 | 1.178226451 | 1.46E-09 | 7.79E-09 | up |
| Xpnpep3 | 1.157155944 | 1.47E-09 | 7.85E-09 | up |
| Lama4 | 1.054575466 | 1.57E-09 | 8.36E-09 | up |
| Gnb4 | 1.117663419 | 1.61E-09 | 8.53E-09 | up |
| Cadm3 | 1.43383457 | 1.62E-09 | 8.59E-09 | up |
| Lypd6 | 1.159851297 | 1.62E-09 | 8.59E-09 | up |
| Trip13 | 1.21600781 | 1.63E-09 | 8.63E-09 | up |
| Lyst | 1.185995666 | 1.64E-09 | 8.67E-09 | up |
| Lrrc15 | 2.818987795 | 1.70E-09 | 8.98E-09 | up |
| Apoc1 | 1.228634373 | 1.74E-09 | 9.15E-09 | up |
| Sfrp4 | 1.737090537 | 1.84E-09 | 9.69E-09 | up |
| Ramp3 | 1.196549534 | 1.93E-09 | 1.01E-08 | up |
| H2-M9 | 1.739513795 | 2.22E-09 | 1.16E-08 | up |
| Syne3 | 1.225252558 | 2.39E-09 | 1.24E-08 | up |
| Ptprt | 4.264478781 | 2.45E-09 | 1.27E-08 | up |
| Cpsf4l | 2.49177177 | 2.55E-09 | 1.32E-08 | up |
| Idh1 | 1.113519212 | 2.70E-09 | 1.39E-08 | up |
| Cbwd1 | 1.023289658 | 2.96E-09 | 1.52E-08 | up |
| Npc2 | 1.002489132 | 3.06E-09 | 1.57E-08 | up |
| Ryr2 | 2.46517801 | 3.31E-09 | 1.69E-08 | up |
| Gal3st2 | 2.919319701 | 3.57E-09 | 1.81E-08 | up |
| Postn | 1.15374314 | 3.63E-09 | 1.84E-08 | up |
| 9130230L23Rik | 1.804546144 | 3.73E-09 | 1.89E-08 | up |
| Pgm2l1 | 1.014349049 | 3.91E-09 | 1.98E-08 | up |
| Alk | 2.115395587 | 4.21E-09 | 2.12E-08 | up |
| Rasl11a | 1.61610461 | 4.36E-09 | 2.19E-08 | up |
| Hdac4 | 1.294989187 | 4.63E-09 | 2.32E-08 | up |
| Rgs8 | 3.203544695 | 4.70E-09 | 2.35E-08 | up |
| Cdk5r1 | 1.1342837 | 5.04E-09 | 2.51E-08 | up |
| Krt9 | 6.319628237 | 5.29E-09 | 2.62E-08 | up |
| Zfp804b | 2.240713775 | 5.55E-09 | 2.75E-08 | up |
| Kcnj4 | 3.995877926 | 5.60E-09 | 2.77E-08 | up |
| Ogdhl | 1.023321243 | 6.03E-09 | 2.97E-08 | up |
| Gldn | 2.724458903 | 6.16E-09 | 3.04E-08 | up |
| Tmem252 | 2.159106678 | 6.50E-09 | 3.19E-08 | up |
| Gpr123 | 3.047098047 | 7.02E-09 | 3.43E-08 | up |
| Clvs1 | 1.915144865 | 7.17E-09 | 3.50E-08 | up |
| Angptl7 | 2.475568693 | 7.26E-09 | 3.54E-08 | up |
| B4galnt2 | 2.395443566 | 7.30E-09 | 3.56E-08 | up |
| Trp53i11 | 1.38112742 | 7.58E-09 | 3.69E-08 | up |
| Spag4 | 1.809188139 | 7.86E-09 | 3.81E-08 | up |
| Arxes2 | 1.189441214 | 8.31E-09 | 4.02E-08 | up |
| Il24 | 3.905507737 | 8.41E-09 | 4.06E-08 | up |
| Grpr | 2.722059115 | 8.57E-09 | 4.14E-08 | up |
| Slc7a3 | 2.403446767 | 8.78E-09 | 4.23E-08 | up |
| 3010026O09Rik | 1.019880253 | 8.86E-09 | 4.27E-08 | up |
| Vstm2b | 2.029560947 | 8.89E-09 | 4.28E-08 | up |
| Rftn2 | 1.620445792 | 9.18E-09 | 4.42E-08 | up |
| Gli1 | 1.893695366 | 9.30E-09 | 4.47E-08 | up |
| Sema5b | 2.44055016 | 9.35E-09 | 4.49E-08 | up |
| Zfp69 | 1.170354032 | 9.36E-09 | 4.49E-08 | up |
| Slc46a2 | 4.753553522 | 9.41E-09 | 4.51E-08 | up |
| Lnx1 | 1.24392561 | 1.00E-08 | 4.80E-08 | up |
| Kif26b | 2.081699644 | 1.03E-08 | 4.90E-08 | up |
| Nbeal1 | 1.247057581 | 1.06E-08 | 5.05E-08 | up |
| Bora | 1.113557541 | 1.09E-08 | 5.15E-08 | up |
| Rassf8 | 1.523274234 | 1.12E-08 | 5.30E-08 | up |
| Gjb4 | 1.229654051 | 1.12E-08 | 5.31E-08 | up |
| Nr2f1 | 1.194790467 | 1.27E-08 | 5.95E-08 | up |
| Slfn10-ps | 2.776088221 | 1.30E-08 | 6.09E-08 | up |
| Luzp2 | 2.607019591 | 1.33E-08 | 6.26E-08 | up |
| Hist1h1d | 1.612215336 | 1.42E-08 | 6.63E-08 | up |
| Ces1d | 1.069652646 | 1.53E-08 | 7.14E-08 | up |
| Lcn2 | 2.863700583 | 2.04E-08 | 9.40E-08 | up |
| Erg | 1.236497256 | 2.05E-08 | 9.47E-08 | up |
| Hs6st3 | 2.859617599 | 2.20E-08 | 1.01E-07 | up |
| Gpc1 | 1.420312687 | 2.36E-08 | 1.08E-07 | up |
| Stard4 | 1.432118457 | 2.59E-08 | 1.18E-07 | up |
| Iqcg | 1.15633063 | 2.59E-08 | 1.18E-07 | up |
| Svopl | 1.2217713 | 2.82E-08 | 1.28E-07 | up |
| 1700055N04Rik | 2.352158601 | 3.03E-08 | 1.37E-07 | up |
| Rnft2 | 1.209094438 | 3.29E-08 | 1.48E-07 | up |
| Pdpr | 1.394833964 | 3.76E-08 | 1.68E-07 | up |
| Bmp2 | 1.497214901 | 3.84E-08 | 1.72E-07 | up |
| Gfra1 | 1.53249858 | 4.10E-08 | 1.83E-07 | up |
| Vash1 | 1.264075212 | 4.71E-08 | 2.09E-07 | up |
| Aldh1a3 | 1.732893178 | 5.34E-08 | 2.35E-07 | up |
| Pthlh | 1.661240347 | 5.67E-08 | 2.48E-07 | up |
| Tacr1 | 1.378614735 | 5.69E-08 | 2.49E-07 | up |
| Tnfrsf22 | 1.482195644 | 5.83E-08 | 2.54E-07 | up |
| Lat | 2.456797766 | 6.11E-08 | 2.66E-07 | up |
| Wnt2 | 2.0179544 | 6.17E-08 | 2.68E-07 | up |
| Eppin | 5.05270026 | 6.40E-08 | 2.78E-07 | up |
| Chac1 | 1.557102566 | 6.69E-08 | 2.90E-07 | up |
| Gm2115 | 3.566011713 | 7.48E-08 | 3.21E-07 | up |
| Phf16 | 1.436462639 | 8.23E-08 | 3.52E-07 | up |
| Vstm4 | 1.096883773 | 8.31E-08 | 3.55E-07 | up |
| Tacr3 | 2.84789526 | 8.47E-08 | 3.62E-07 | up |
| Ephb6 | 1.677614271 | 9.21E-08 | 3.92E-07 | up |
| Arpp21 | 3.497958458 | 1.00E-07 | 4.25E-07 | up |
| Rims2 | 1.191987476 | 1.04E-07 | 4.40E-07 | up |
| Penk | 1.388478776 | 1.08E-07 | 4.54E-07 | up |
| Agtr2 | 2.499146777 | 1.12E-07 | 4.69E-07 | up |
| P2rx3 | 1.779852424 | 1.19E-07 | 5.00E-07 | up |
| Rgs5 | 1.044650755 | 1.23E-07 | 5.13E-07 | up |
| Adamts2 | 1.039302248 | 1.24E-07 | 5.20E-07 | up |
| Sema6b | 1.073982698 | 1.32E-07 | 5.51E-07 | up |
| Sarm1 | 1.384184662 | 1.62E-07 | 6.66E-07 | up |
| Prdm11 | 1.331894224 | 1.64E-07 | 6.76E-07 | up |
| Sfrp1 | 1.208642121 | 1.72E-07 | 7.07E-07 | up |
| 9230110C19Rik | 1.154222689 | 1.78E-07 | 7.29E-07 | up |
| Ly6g6c | 2.722092043 | 1.97E-07 | 8.03E-07 | up |
| Tshz2 | 1.108032641 | 2.02E-07 | 8.21E-07 | up |
| Camk1d | 1.124782513 | 2.17E-07 | 8.82E-07 | up |
| Itga2 | 2.185454619 | 2.18E-07 | 8.84E-07 | up |
| Asb11 | 2.482188201 | 2.37E-07 | 9.60E-07 | up |
| Gdpd2 | 2.867698827 | 2.43E-07 | 9.84E-07 | up |
| Gabrp | 1.815565512 | 2.53E-07 | 1.02E-06 | up |
| Fgf18 | 2.098722092 | 2.55E-07 | 1.03E-06 | up |
| Fam13c | 1.288622558 | 2.55E-07 | 1.03E-06 | up |
| Dbx2 | 1.936956047 | 2.62E-07 | 1.05E-06 | up |
| Cd109 | 1.458536229 | 2.70E-07 | 1.09E-06 | up |
| Acot5 | 2.795571677 | 2.82E-07 | 1.13E-06 | up |
| Lrguk | 1.431362651 | 2.84E-07 | 1.14E-06 | up |
| Kif18a | 1.022076336 | 3.08E-07 | 1.23E-06 | up |
| Gda | 1.044130967 | 3.13E-07 | 1.25E-06 | up |
| Ticrr | 1.654425043 | 3.19E-07 | 1.27E-06 | up |
| Bambi | 1.323872194 | 3.21E-07 | 1.28E-06 | up |
| Efcc1 | 1.515758091 | 3.30E-07 | 1.31E-06 | up |
| Igsf1 | 1.968453953 | 3.37E-07 | 1.33E-06 | up |
| Gfod1 | 1.01592922 | 3.56E-07 | 1.41E-06 | up |
| Fibin | 1.311950722 | 3.57E-07 | 1.41E-06 | up |
| Sntb1 | 1.289334031 | 3.74E-07 | 1.47E-06 | up |
| Vsig8 | 1.328733106 | 3.86E-07 | 1.52E-06 | up |
| Foxc2 | 1.585993885 | 3.89E-07 | 1.53E-06 | up |
| Nrip1 | 1.456614969 | 3.94E-07 | 1.55E-06 | up |
| Upp1 | 1.19224852 | 4.04E-07 | 1.58E-06 | up |
| Zbtb32 | 1.144951303 | 4.21E-07 | 1.64E-06 | up |
| Sez6l2 | 1.077910336 | 4.23E-07 | 1.65E-06 | up |
| Lrriq1 | 2.149049761 | 4.25E-07 | 1.66E-06 | up |
| Gsg2 | 1.236599972 | 4.26E-07 | 1.66E-06 | up |
| Tcp11l1 | 1.034790238 | 4.30E-07 | 1.68E-06 | up |
| Pcsk4 | 1.217893637 | 4.56E-07 | 1.77E-06 | up |
| Gprc5a | 1.008560868 | 4.72E-07 | 1.83E-06 | up |
| Ammecr1 | 1.289643544 | 5.12E-07 | 1.97E-06 | up |
| Hs2st1 | 1.026598038 | 5.21E-07 | 2.01E-06 | up |
| Fezf1 | 3.879617262 | 5.51E-07 | 2.12E-06 | up |
| Nox4 | 1.090455981 | 5.67E-07 | 2.17E-06 | up |
| Fam19a3 | 1.308348974 | 5.87E-07 | 2.25E-06 | up |
| Gpr98 | 1.400589382 | 6.00E-07 | 2.29E-06 | up |
| Bmp3 | 2.139458494 | 6.16E-07 | 2.35E-06 | up |
| Atp6v1b1 | 2.631819788 | 6.28E-07 | 2.39E-06 | up |
| Lmbrd2 | 1.45930568 | 6.70E-07 | 2.54E-06 | up |
| Plcl2 | 1.075916365 | 6.97E-07 | 2.64E-06 | up |
| Cdh11 | 1.154506293 | 7.04E-07 | 2.67E-06 | up |
| Gcg | 7.152891993 | 7.58E-07 | 2.86E-06 | up |
| Ccdc176 | 1.048786766 | 7.62E-07 | 2.88E-06 | up |
| Dnahc17 | 2.38460652 | 7.74E-07 | 2.92E-06 | up |
| Srgap1 | 1.051661398 | 8.72E-07 | 3.27E-06 | up |
| E130012A19Rik | 1.084790008 | 8.80E-07 | 3.30E-06 | up |
| Ccdc67 | 1.400122744 | 9.09E-07 | 3.40E-06 | up |
| Adra1d | 1.668891065 | 9.28E-07 | 3.47E-06 | up |
| E330009J07Rik | 1.413459173 | 9.56E-07 | 3.56E-06 | up |
| E2f7 | 1.226339987 | 9.95E-07 | 3.70E-06 | up |
| Cntnap5a | 2.034657347 | 1.06E-06 | 3.91E-06 | up |
| Olfml2a | 1.262212006 | 1.13E-06 | 4.16E-06 | up |
| 2810408I11Rik | 1.304119568 | 1.17E-06 | 4.28E-06 | up |
| Kcnj2 | 1.145609347 | 1.18E-06 | 4.34E-06 | up |
| Rdh1 | 2.062889425 | 1.19E-06 | 4.38E-06 | up |
| Fam83a | 1.924873197 | 1.23E-06 | 4.50E-06 | up |
| Wipf3 | 1.505050604 | 1.24E-06 | 4.54E-06 | up |
| Gm15413 | 1.146827834 | 1.34E-06 | 4.90E-06 | up |
| Tmem82 | 1.010077186 | 1.48E-06 | 5.37E-06 | up |
| Slc14a2 | 1.438756254 | 1.52E-06 | 5.50E-06 | up |
| Catsperd | 1.351628362 | 1.53E-06 | 5.52E-06 | up |
| Gm14207 | 1.472956102 | 1.61E-06 | 5.80E-06 | up |
| 2810417H13Rik | 1.278421246 | 1.61E-06 | 5.81E-06 | up |
| Slc29a2 | 1.049729394 | 1.73E-06 | 6.22E-06 | up |
| Gpx3 | 1.178751186 | 1.75E-06 | 6.27E-06 | up |
| Slc25a34 | 1.70976542 | 1.76E-06 | 6.30E-06 | up |
| Plac1 | 4.560874021 | 1.82E-06 | 6.50E-06 | up |
| Slc35f2 | 1.005519352 | 1.86E-06 | 6.65E-06 | up |
| Gareml | 2.530461634 | 1.89E-06 | 6.74E-06 | up |
| Cdh2 | 1.255845314 | 1.94E-06 | 6.90E-06 | up |
| Ildr2 | 1.133985397 | 1.94E-06 | 6.92E-06 | up |
| Vsig2 | 1.634620575 | 1.97E-06 | 7.01E-06 | up |
| Mgp | 1.067850107 | 2.06E-06 | 7.30E-06 | up |
| Dhrs9 | 2.185758214 | 2.09E-06 | 7.42E-06 | up |
| Asgr2 | 1.225377104 | 2.13E-06 | 7.54E-06 | up |
| Micalcl | 1.008580501 | 2.14E-06 | 7.56E-06 | up |
| Sprr2g | 3.898602641 | 2.20E-06 | 7.78E-06 | up |
| Hist1h1e | 1.222222295 | 2.25E-06 | 7.93E-06 | up |
| Kcnt1 | 3.036465308 | 2.30E-06 | 8.12E-06 | up |
| Extl1 | 2.023621286 | 2.39E-06 | 8.41E-06 | up |
| Alox15 | 2.189146019 | 2.56E-06 | 8.98E-06 | up |
| Thbs4 | 2.164093898 | 2.62E-06 | 9.16E-06 | up |
| Ppp1r3e | 1.22888972 | 2.70E-06 | 9.41E-06 | up |
| Itih1 | 3.15638138 | 2.76E-06 | 9.63E-06 | up |
| Lcor | 1.556077804 | 2.85E-06 | 9.89E-06 | up |
| Amdhd1 | 2.151555514 | 2.88E-06 | 1.00E-05 | up |
| Rhov | 1.483600481 | 2.90E-06 | 1.01E-05 | up |
| Pde10a | 1.117813376 | 2.92E-06 | 1.01E-05 | up |
| Trpc1 | 1.006111118 | 3.12E-06 | 1.08E-05 | up |
| Adamts8 | 1.084405258 | 3.23E-06 | 1.11E-05 | up |
| Areg | 2.230776358 | 3.24E-06 | 1.12E-05 | up |
| Ly75 | 1.083465306 | 3.39E-06 | 1.17E-05 | up |
| Nxnl1 | 1.334782509 | 3.46E-06 | 1.19E-05 | up |
| Dna2 | 1.040206982 | 4.02E-06 | 1.37E-05 | up |
| Glb1l2 | 1.225386761 | 4.09E-06 | 1.39E-05 | up |
| Cacng1 | 1.476667415 | 4.55E-06 | 1.53E-05 | up |
| Ddi2 | 1.051783541 | 4.65E-06 | 1.56E-05 | up |
| Col25a1 | 1.996790059 | 4.77E-06 | 1.60E-05 | up |
| Tnfrsf26 | 1.294175682 | 5.02E-06 | 1.68E-05 | up |
| Dclk2 | 1.294948858 | 5.14E-06 | 1.72E-05 | up |
| Cntn4 | 2.998105361 | 5.16E-06 | 1.72E-05 | up |
| Adamts6 | 1.586852102 | 5.25E-06 | 1.75E-05 | up |
| Cacng7 | 1.299990005 | 5.43E-06 | 1.80E-05 | up |
| 2900079G21Rik | 2.486195662 | 5.44E-06 | 1.81E-05 | up |
| Matn3 | 3.630725145 | 5.83E-06 | 1.93E-05 | up |
| Myh7b | 1.884863609 | 6.12E-06 | 2.02E-05 | up |
| 9130019P16Rik | 1.533250225 | 6.74E-06 | 2.21E-05 | up |
| Nt5dc2 | 1.064242753 | 6.81E-06 | 2.23E-05 | up |
| Awat2 | 1.648454872 | 6.85E-06 | 2.24E-05 | up |
| Npm2 | 1.816831585 | 7.19E-06 | 2.35E-05 | up |
| Cyp46a1 | 1.282704626 | 7.20E-06 | 2.35E-05 | up |
| Ptgs2 | 1.610541591 | 7.43E-06 | 2.42E-05 | up |
| Fam124a | 1.014782645 | 7.53E-06 | 2.45E-05 | up |
| Meiob | 1.433066455 | 7.59E-06 | 2.46E-05 | up |
| Gm266 | 1.871056878 | 7.88E-06 | 2.55E-05 | up |
| F2rl3 | 1.889165562 | 8.00E-06 | 2.59E-05 | up |
| Esco2 | 1.020777058 | 8.74E-06 | 2.82E-05 | up |
| Adra2a | 1.385138298 | 9.85E-06 | 3.15E-05 | up |
| Ceacam19 | 1.366697888 | 9.96E-06 | 3.19E-05 | up |
| AI593442 | 2.324805465 | 1.04E-05 | 3.31E-05 | up |
| Iqsec3 | 1.203532093 | 1.04E-05 | 3.34E-05 | up |
| Endou | 1.649441049 | 1.05E-05 | 3.36E-05 | up |
| Frat1 | 1.154542555 | 1.14E-05 | 3.61E-05 | up |
| L3mbtl4 | 1.1299964 | 1.14E-05 | 3.62E-05 | up |
| 9230114K14Rik | 1.137748526 | 1.17E-05 | 3.70E-05 | up |
| Chst1 | 1.108983956 | 1.17E-05 | 3.71E-05 | up |
| Ngf | 1.201789378 | 1.20E-05 | 3.78E-05 | up |
| Lama1 | 2.048251528 | 1.23E-05 | 3.89E-05 | up |
| Mnx1 | 2.310247802 | 1.28E-05 | 4.02E-05 | up |
| Itgb3 | 1.044048027 | 1.30E-05 | 4.10E-05 | up |
| Tmem211 | 1.756515881 | 1.33E-05 | 4.17E-05 | up |
| Dmp1 | 1.814857133 | 1.41E-05 | 4.41E-05 | up |
| Pcdhb10 | 1.641530323 | 1.41E-05 | 4.41E-05 | up |
| P2rx1 | 1.697573744 | 1.43E-05 | 4.48E-05 | up |
| Gimap5 | 1.137825397 | 1.49E-05 | 4.63E-05 | up |
| Cela1 | 1.383757369 | 1.67E-05 | 5.16E-05 | up |
| Casc5 | 1.099964046 | 1.74E-05 | 5.36E-05 | up |
| Gpr88 | 2.757681677 | 1.86E-05 | 5.71E-05 | up |
| Chrnb2 | 1.204745709 | 2.03E-05 | 6.21E-05 | up |
| 4930429F24Rik | 1.28151989 | 2.33E-05 | 7.08E-05 | up |
| Adora2a | 1.024183245 | 2.49E-05 | 7.53E-05 | up |
| Pkd1l2 | 1.603257358 | 2.56E-05 | 7.72E-05 | up |
| Nr5a2 | 1.414769157 | 2.59E-05 | 7.80E-05 | up |
| Lrrc8b | 1.018927986 | 2.78E-05 | 8.34E-05 | up |
| Rad51b | 1.134530735 | 2.90E-05 | 8.67E-05 | up |
| Samd12 | 1.556983632 | 3.53E-05 | 0.000104 | up |
| Emilin3 | 2.581353371 | 3.63E-05 | 0.0001068 | up |
| C130083M11Rik | 1.248508572 | 3.72E-05 | 0.0001093 | up |
| Lrr1 | 1.328799094 | 3.73E-05 | 0.0001094 | up |
| Ggnbp1 | 1.112030917 | 3.93E-05 | 0.0001149 | up |
| Adprhl1 | 2.339524278 | 4.08E-05 | 0.0001188 | up |
| Prg4 | 1.128290764 | 4.11E-05 | 0.0001197 | up |
| Eno4 | 1.090178708 | 4.69E-05 | 0.0001354 | up |
| AI854703 | 2.294402175 | 5.00E-05 | 0.0001435 | up |
| Mark1 | 1.037300413 | 5.25E-05 | 0.0001501 | up |
| 4930470H14Rik | 1.119578519 | 5.29E-05 | 0.0001511 | up |
| Wdfy2 | 1.020935961 | 5.41E-05 | 0.0001544 | up |
| LOC100504608 | 1.269983005 | 5.49E-05 | 0.0001566 | up |
| Nmu | 1.650731017 | 5.53E-05 | 0.0001577 | up |
| Fam217a | 1.918079576 | 5.81E-05 | 0.000165 | up |
| Rhbg | 1.079605629 | 6.12E-05 | 0.000173 | up |
| Nhs | 1.113115128 | 6.24E-05 | 0.0001763 | up |
| Epdr1 | 1.178505945 | 6.68E-05 | 0.0001874 | up |
| D830031N03Rik | 1.247849464 | 6.80E-05 | 0.0001903 | up |
| Zfyve28 | 1.088537529 | 6.96E-05 | 0.0001945 | up |
| Gm10658 | 1.09857529 | 7.07E-05 | 0.0001971 | up |
| Ubash3a | 2.435318162 | 7.53E-05 | 0.0002093 | up |
| Adamts13 | 2.486239816 | 7.72E-05 | 0.0002142 | up |
| Dnahc9 | 2.457983837 | 7.74E-05 | 0.0002146 | up |
| Gm1332 | 3.061482523 | 7.80E-05 | 0.000216 | up |
| Trim67 | 1.149214323 | 8.33E-05 | 0.0002298 | up |
| Hk2 | 1.048682668 | 8.59E-05 | 0.0002365 | up |
| Slc2a13 | 1.159309855 | 9.03E-05 | 0.0002479 | up |
| Rps6ka6 | 1.184514582 | 9.40E-05 | 0.0002575 | up |
| Kif1a | 1.144266499 | 9.59E-05 | 0.0002623 | up |
| Hipk2 | 1.291608799 | 9.94E-05 | 0.0002711 | up |
| B3galt1 | 1.606770261 | 0.0001005 | 0.0002738 | up |
| Rnf125 | 1.164437455 | 0.0001018 | 0.0002773 | up |
| Lepr | 1.065623899 | 0.000102 | 0.0002778 | up |
| Kcne4 | 1.141178166 | 0.0001022 | 0.0002783 | up |
| Dusp8 | 1.000761369 | 0.0001099 | 0.0002975 | up |
| Grtp1 | 1.159395993 | 0.0001202 | 0.0003228 | up |
| Klhl4 | 1.033940188 | 0.0001258 | 0.0003365 | up |
| Wscd2 | 1.456380585 | 0.0001307 | 0.0003485 | up |
| Pcdhb9 | 1.368028928 | 0.0001321 | 0.000352 | up |
| BC006965 | 1.045814741 | 0.0001406 | 0.0003724 | up |
| Frmd5 | 2.254764891 | 0.0001425 | 0.0003771 | up |
| Hs6st2 | 1.248321151 | 0.0001479 | 0.0003901 | up |
| Chsy3 | 1.458028562 | 0.0001506 | 0.0003964 | up |
| Rapgef5 | 1.124220185 | 0.0001584 | 0.0004158 | up |
| Tmem44 | 1.423089754 | 0.0001599 | 0.0004192 | up |
| Stil | 1.064927543 | 0.0001735 | 0.0004519 | up |
| Uprt | 1.153038217 | 0.0001767 | 0.0004597 | up |
| Lgr5 | 1.877565525 | 0.0001832 | 0.000476 | up |
| Pcdhb5 | 1.532653847 | 0.0001925 | 0.000498 | up |
| Galr2 | 1.255326862 | 0.0001987 | 0.0005128 | up |
| Gdf10 | 1.030322588 | 0.0002036 | 0.0005243 | up |
| Bdkrb2 | 1.159460822 | 0.000218 | 0.0005592 | up |
| Cysltr2 | 1.532630225 | 0.000221 | 0.0005662 | up |
| Cyp26a1 | 1.032251476 | 0.0002334 | 0.0005953 | up |
| Hsbp1l1 | 1.109227309 | 0.0002377 | 0.000605 | up |
| Kcnma1 | 1.677249021 | 0.0002429 | 0.0006174 | up |
| Syt5 | 1.330402042 | 0.000244 | 0.00062 | up |
| Ovgp1 | 1.115802903 | 0.0002734 | 0.0006891 | up |
| Fam163b | 1.342824225 | 0.0002766 | 0.0006965 | up |
| Foxp2 | 1.043329192 | 0.0002775 | 0.0006983 | up |
| Prkg2 | 1.225593141 | 0.0002806 | 0.0007049 | up |
| Tdg | 1.087370874 | 0.0002996 | 0.0007491 | up |
| Dab1 | 1.39430037 | 0.0003007 | 0.0007516 | up |
| Slc6a1 | 4.55380902 | 0.0003197 | 0.0007938 | up |
| Ttpa | 1.495961563 | 0.0003267 | 0.0008094 | up |
| Ptch2 | 1.473229199 | 0.0003549 | 0.0008736 | up |
| Pard6g | 1.02519255 | 0.0003872 | 0.0009477 | up |
| Gcgr | 1.244617857 | 0.0004235 | 0.0010309 | up |
| Tmem178b | 1.265537039 | 0.0004383 | 0.0010637 | up |
| Cntnap2 | 1.379401888 | 0.0004566 | 0.0011047 | up |
| 4931403E22Rik | 1.129280139 | 0.0004711 | 0.0011375 | up |
| Prokr1 | 1.136183349 | 0.0005227 | 0.0012521 | up |
| Gm10584 | 1.028881971 | 0.0005772 | 0.0013731 | up |
| Serpinb1a | 1.040981051 | 0.0005815 | 0.0013822 | up |
| Arsj | 1.244648432 | 0.0006197 | 0.0014661 | up |
| Cntnap3 | 2.203769109 | 0.0006328 | 0.0014951 | up |
| Dlx3 | 1.233563634 | 0.000721 | 0.0016846 | up |
| Tmem151a | 1.148284162 | 0.000767 | 0.0017829 | up |
| Nr4a3 | 1.197560865 | 0.0008453 | 0.0019534 | up |
| Ctxn3 | 2.392080455 | 0.0008594 | 0.0019824 | up |
| Gm4841 | 1.066482787 | 0.0009467 | 0.0021672 | up |
| Creb3l3 | 1.992147075 | 0.0010158 | 0.0023112 | up |
| B3gnt3 | 1.008302606 | 0.0010565 | 0.002399 | up |
| Rarres1 | 1.323027425 | 0.0010659 | 0.0024167 | up |
| 4930578C19Rik | 1.08812051 | 0.0012533 | 0.0028028 | up |
| Bai2 | 1.098102206 | 0.001304 | 0.0029067 | up |
| Sult1d1 | 1.199778436 | 0.0013879 | 0.0030736 | up |
| Bst1 | 1.614791357 | 0.0014422 | 0.0031815 | up |
| C2cd4b | 1.396764932 | 0.0014814 | 0.0032558 | up |
| Pcdhb11 | 1.064445176 | 0.0016655 | 0.0036235 | up |
| D330050G23Rik | 1.192757427 | 0.0016681 | 0.003628 | up |
| Cd3d | 1.86635066 | 0.0016905 | 0.0036702 | up |
| Esr1 | 1.187423273 | 0.0017012 | 0.0036905 | up |
| Cilp2 | 1.183820644 | 0.0018737 | 0.0040317 | up |
| Rdh9 | 1.055488455 | 0.0019342 | 0.0041478 | up |
| Cd3g | 1.507582972 | 0.0022562 | 0.0047671 | up |
| Mmp13 | 1.450463274 | 0.0022778 | 0.0048078 | up |
| Zfp366 | 1.002258777 | 0.0024694 | 0.0051761 | up |
| Slc15a1 | 1.087161413 | 0.0024816 | 0.0051994 | up |
| 4833422C13Rik | 1.061263118 | 0.0024831 | 0.0052019 | up |
| Ifi27l2b | 1.671900903 | 0.002651 | 0.0055233 | up |
| Cyp1a1 | 1.725538293 | 0.0027443 | 0.0057046 | up |
| Piwil4 | 1.312056118 | 0.0027637 | 0.0057423 | up |
| Wt1 | 1.245165483 | 0.0027906 | 0.0057925 | up |
| Ereg | 1.403833741 | 0.0031375 | 0.0064452 | up |
| Cyp26b1 | 1.19725136 | 0.0032768 | 0.0067066 | up |
| Sntg1 | 1.207047577 | 0.0036013 | 0.0073099 | up |
| B430212C06Rik | 1.177197944 | 0.0037184 | 0.0075328 | up |
| Dpp6 | 1.696610386 | 0.0038411 | 0.0077565 | up |
| Dusp4 | 1.118954193 | 0.0040688 | 0.008164 | up |
| Igsf21 | 1.654603929 | 0.0041036 | 0.0082237 | up |
| Gp1bb | 1.003919004 | 0.00426 | 0.0085124 | up |
| Shisa7 | 1.526906068 | 0.0043296 | 0.0086324 | up |
| Vnn3 | 1.711385913 | 0.0044321 | 0.0088138 | up |
| St8sia1 | 1.307690153 | 0.0045868 | 0.009094 | up |
| Kcnc4 | 1.880304733 | 0.0047192 | 0.0093323 | up |
| Otoa | 1.389043186 | 0.0051035 | 0.0100185 | up |
| Scel | 1.29572792 | 0.0052996 | 0.0103697 | up |
| Foxd2 | 1.01598865 | 0.0054124 | 0.0105662 | up |
| Inhba | 1.199192946 | 0.0058212 | 0.0112883 | up |
| Acan | 2.749899177 | 0.0062721 | 0.0120869 | up |
| Spsb4 | 1.023068577 | 0.00628 | 0.0120973 | up |
| Nrxn3 | 1.576249093 | 0.0064257 | 0.0123468 | up |
| Nsg2 | 1.912322704 | 0.0070533 | 0.0134123 | up |
| 3830403N18Rik | 1.476342337 | 0.0071835 | 0.013633 | up |
| 2610018G03Rik | 1.035148093 | 0.0076943 | 0.0145284 | up |
| Sult1b1 | 1.000492872 | 0.0082325 | 0.015446 | up |
| Ankrd1 | 2.194976701 | 0.0082431 | 0.015462 | up |
| Wnk3 | 1.078473496 | 0.0086624 | 0.0161711 | up |
| Slc30a2 | 1.654153018 | 0.0087894 | 0.0163829 | up |
| Gdf5 | 1.241455506 | 0.0096759 | 0.0178745 | up |
| Cml3 | 1.074027876 | 0.0105852 | 0.0193791 | up |
| Col24a1 | 1.353390492 | 0.0108449 | 0.0198044 | up |
| Mei1 | 1.154760442 | 0.0108617 | 0.0198301 | up |
| Fpr1 | 1.645925299 | 0.0121807 | 0.0219866 | up |
| Smyd1 | 1.233759852 | 0.0133909 | 0.0238973 | up |
| 2010003K11Rik | 1.308942453 | 0.0153653 | 0.0270417 | up |
| Serpinb9b | 1.259131125 | 0.0158229 | 0.0277459 | up |
| Serpinb11 | 1.140770953 | 0.0168452 | 0.0293541 | up |
| Tnnt1 | 1.698467472 | 0.0173883 | 0.0302099 | up |
| G530011O06Rik | 1.405911115 | 0.0183607 | 0.0317774 | up |
| Sprr2b | 2.69144761 | 0.0208319 | 0.0355498 | up |
| Gm11128 | 1.233830506 | 0.0229149 | 0.0387443 | up |
| Htra4 | 1.053027158 | 0.0238295 | 0.0400913 | up |
| Afp | 1.360516581 | 0.0280881 | 0.0463883 | up |
| Cnp | -2.796712443 | 7.64E-147 | 1.11E-143 | down |
| Mall | -2.61557717 | 1.75E-127 | 1.59E-124 | down |
| Rtn1 | -5.129461194 | 4.13E-122 | 3.33E-119 | down |
| Tesc | -5.658795255 | 1.56E-117 | 1.03E-114 | down |
| Anxa3 | -2.617258702 | 8.30E-117 | 5.24E-114 | down |
| Itgb4 | -2.714464411 | 1.05E-102 | 5.09E-100 | down |
| Ptges | -3.904308317 | 4.19E-100 | 1.74E-97 | down |
| Rlbp1 | -4.011338926 | 4.85E-98 | 1.85E-95 | down |
| Plxnb1 | -2.787400091 | 7.85E-94 | 2.65E-91 | down |
| Sel1l3 | -6.466832197 | 3.81E-92 | 1.15E-89 | down |
| Mfi2 | -4.198872655 | 3.96E-92 | 1.17E-89 | down |
| Ngef | -3.847512842 | 6.68E-92 | 1.94E-89 | down |
| Tnfrsf9 | -4.416672398 | 1.49E-91 | 4.17E-89 | down |
| Paqr6 | -4.671229207 | 2.43E-91 | 6.66E-89 | down |
| Aldh3a2 | -1.562922847 | 2.31E-90 | 5.98E-88 | down |
| Dclk1 | -4.066078164 | 2.40E-87 | 5.53E-85 | down |
| Inf2 | -2.066472359 | 9.42E-86 | 2.07E-83 | down |
| Eno2 | -3.636102183 | 3.04E-85 | 6.49E-83 | down |
| Ooep | -4.042122297 | 9.17E-83 | 1.85E-80 | down |
| Itga4 | -3.11134794 | 1.01E-82 | 2.01E-80 | down |
| Atp6v0e2 | -2.080815039 | 1.30E-81 | 2.55E-79 | down |
| Meis3 | -3.502831759 | 1.93E-80 | 3.69E-78 | down |
| Emb | -2.358935794 | 1.44E-78 | 2.61E-76 | down |
| Cd9 | -2.909559012 | 2.58E-77 | 4.50E-75 | down |
| Nyap1 | -4.700092008 | 3.75E-77 | 6.47E-75 | down |
| Gca | -1.85663233 | 5.02E-74 | 7.91E-72 | down |
| Sytl1 | -3.827850768 | 5.86E-73 | 8.95E-71 | down |
| Cxcl11 | -6.754164935 | 3.55E-71 | 5.36E-69 | down |
| Naaa | -1.986993752 | 4.71E-71 | 7.04E-69 | down |
| Cldn9 | -3.751362596 | 8.04E-70 | 1.16E-67 | down |
| Evpl | -2.360401378 | 1.88E-69 | 2.67E-67 | down |
| Adora1 | -2.182072845 | 1.47E-67 | 2.05E-65 | down |
| Ikbke | -2.645639837 | 1.65E-67 | 2.27E-65 | down |
| Gipc2 | -3.983910653 | 3.63E-67 | 4.92E-65 | down |
| Plcxd1 | -2.446788683 | 4.09E-67 | 5.50E-65 | down |
| Adam22 | -2.2693697 | 5.74E-67 | 7.57E-65 | down |
| Clic3 | -2.557719008 | 6.66E-67 | 8.71E-65 | down |
| Ttc39a | -2.765658803 | 5.23E-66 | 6.65E-64 | down |
| Plcd4 | -2.148947403 | 7.29E-66 | 9.04E-64 | down |
| Mxra7 | -2.588716782 | 2.37E-65 | 2.86E-63 | down |
| Vwa7 | -5.315901176 | 4.45E-65 | 5.25E-63 | down |
| Thnsl2 | -2.886006827 | 6.64E-65 | 7.71E-63 | down |
| Fam46b | -4.239134613 | 2.94E-64 | 3.34E-62 | down |
| Nfkbia | -1.994679812 | 4.38E-64 | 4.93E-62 | down |
| Mamdc2 | -5.050998453 | 1.27E-62 | 1.34E-60 | down |
| Fam53b | -1.647946953 | 7.30E-62 | 7.56E-60 | down |
| Bcap29 | -2.279603539 | 2.00E-61 | 2.04E-59 | down |
| Mmgt2 | -1.716310709 | 7.34E-61 | 7.39E-59 | down |
| Paqr4 | -2.230582344 | 1.95E-60 | 1.92E-58 | down |
| Lgals1 | -2.77930346 | 4.65E-60 | 4.55E-58 | down |
| Cacnb3 | -2.484660252 | 6.56E-60 | 6.39E-58 | down |
| Smim14 | -1.410226203 | 5.21E-59 | 4.88E-57 | down |
| Crb1 | -4.740010881 | 5.98E-59 | 5.56E-57 | down |
| Acot11 | -3.308051698 | 1.42E-58 | 1.32E-56 | down |
| Cldn4 | -3.093807154 | 2.03E-58 | 1.86E-56 | down |
| Ephx1 | -1.608728065 | 4.50E-58 | 4.03E-56 | down |
| Ptpn22 | -2.161644874 | 1.71E-57 | 1.51E-55 | down |
| Tnfsf12 | -2.413707128 | 8.89E-57 | 7.73E-55 | down |
| Myh14 | -1.954603087 | 4.98E-56 | 4.30E-54 | down |
| Fcer2a | -5.095128124 | 9.94E-56 | 8.48E-54 | down |
| 9530077C05Rik | -3.791644516 | 1.78E-55 | 1.49E-53 | down |
| Fbxo2 | -3.12930404 | 3.19E-55 | 2.66E-53 | down |
| Gstt1 | -2.995068331 | 6.85E-55 | 5.68E-53 | down |
| Btbd6 | -1.842372264 | 9.09E-55 | 7.45E-53 | down |
| Sobp | -3.330595697 | 1.94E-54 | 1.56E-52 | down |
| Fzd6 | -1.58025212 | 3.95E-54 | 3.08E-52 | down |
| Reep2 | -3.328092072 | 4.11E-54 | 3.19E-52 | down |
| Lrp4 | -2.741696085 | 1.22E-53 | 9.31E-52 | down |
| Acox2 | -5.413849096 | 1.72E-53 | 1.30E-51 | down |
| Sema3b | -2.44391562 | 3.46E-53 | 2.55E-51 | down |
| Atp2b4 | -2.044867313 | 7.92E-53 | 5.81E-51 | down |
| S100b | -3.674887936 | 8.02E-53 | 5.85E-51 | down |
| Dlg4 | -2.7169236 | 2.80E-52 | 2.01E-50 | down |
| P2rx6 | -5.050030914 | 3.60E-52 | 2.56E-50 | down |
| Acnat1 | -3.585299924 | 4.44E-52 | 3.14E-50 | down |
| Mia | -3.545232347 | 4.52E-51 | 3.07E-49 | down |
| 1500015O10Rik | -4.395035318 | 4.99E-51 | 3.37E-49 | down |
| Pianp | -1.999859874 | 8.86E-51 | 5.90E-49 | down |
| Pvrl4 | -2.390109271 | 1.72E-50 | 1.13E-48 | down |
| Slc7a7 | -2.062645669 | 1.80E-50 | 1.18E-48 | down |
| Gsn | -3.283120919 | 2.75E-50 | 1.79E-48 | down |
| Cnn2 | -1.543193758 | 2.96E-50 | 1.92E-48 | down |
| D630003M21Rik | -2.783415275 | 5.36E-50 | 3.45E-48 | down |
| Kctd4 | -4.205252353 | 8.37E-50 | 5.34E-48 | down |
| Igfals | -6.915310857 | 8.40E-50 | 5.34E-48 | down |
| Entpd3 | -4.839858539 | 1.00E-49 | 6.25E-48 | down |
| Pam | -2.269453109 | 1.66E-49 | 1.03E-47 | down |
| Lamc2 | -2.279959765 | 2.72E-49 | 1.68E-47 | down |
| Sh3d21 | -2.569023255 | 3.36E-49 | 2.07E-47 | down |
| Tacstd2 | -2.721144671 | 5.39E-49 | 3.30E-47 | down |
| Capsl | -3.239500475 | 5.56E-49 | 3.39E-47 | down |
| Cd40 | -3.924055099 | 2.30E-48 | 1.38E-46 | down |
| Il34 | -1.897549056 | 2.66E-48 | 1.59E-46 | down |
| Tmem79 | -2.324060185 | 2.93E-48 | 1.74E-46 | down |
| Pnmal2 | -2.498659974 | 3.31E-48 | 1.96E-46 | down |
| Slc34a2 | -5.130539855 | 4.38E-48 | 2.56E-46 | down |
| Agbl2 | -3.954825064 | 4.88E-48 | 2.83E-46 | down |
| Rhpn2 | -2.074262242 | 5.98E-48 | 3.44E-46 | down |
| Athl1 | -2.223846228 | 6.21E-48 | 3.56E-46 | down |
| Mansc4 | -4.285722822 | 1.50E-47 | 8.48E-46 | down |
| Slco2a1 | -2.146813633 | 1.77E-47 | 9.80E-46 | down |
| Col6a2 | -2.373023372 | 2.25E-47 | 1.24E-45 | down |
| Pnck | -3.36673381 | 5.09E-47 | 2.78E-45 | down |
| Ap3m2 | -1.752443177 | 1.11E-46 | 6.03E-45 | down |
| Sirpa | -1.78089787 | 1.82E-46 | 9.78E-45 | down |
| Sgpp1 | -1.297096671 | 2.94E-46 | 1.57E-44 | down |
| Pdlim2 | -1.659717285 | 1.03E-45 | 5.48E-44 | down |
| Scg5 | -3.474857459 | 1.31E-45 | 6.93E-44 | down |
| D730039F16Rik | -3.207285867 | 1.51E-45 | 7.93E-44 | down |
| Nfatc1 | -1.553622816 | 3.78E-45 | 1.97E-43 | down |
| Slc35e4 | -2.000120072 | 5.18E-45 | 2.69E-43 | down |
| Lzts2 | -1.222445456 | 1.08E-44 | 5.53E-43 | down |
| Serinc2 | -4.050272814 | 1.81E-44 | 9.17E-43 | down |
| 9030612E09Rik | -4.988936426 | 2.67E-44 | 1.35E-42 | down |
| Grik5 | -2.561814363 | 2.20E-43 | 1.09E-41 | down |
| Slc26a4 | -6.685687561 | 1.03E-42 | 5.00E-41 | down |
| Pmp22 | -1.723156651 | 1.33E-42 | 6.41E-41 | down |
| Tor1b | -1.20595864 | 3.28E-42 | 1.55E-40 | down |
| Mt1 | -2.884054058 | 3.27E-42 | 1.55E-40 | down |
| Eya2 | -1.660625383 | 6.15E-42 | 2.90E-40 | down |
| Smyd2 | -1.513465957 | 7.38E-42 | 3.46E-40 | down |
| Lama3 | -2.388672935 | 1.37E-41 | 6.37E-40 | down |
| Aqp11 | -2.585103433 | 1.39E-41 | 6.43E-40 | down |
| Hebp2 | -2.545523709 | 1.67E-41 | 7.65E-40 | down |
| Rhbdl3 | -3.1759497 | 2.39E-41 | 1.09E-39 | down |
| Arl4c | -2.539988975 | 3.88E-41 | 1.77E-39 | down |
| Npffr2 | -4.842215001 | 4.34E-41 | 1.97E-39 | down |
| Nacc2 | -1.530874112 | 4.48E-41 | 2.02E-39 | down |
| St3gal4 | -1.670558204 | 5.47E-41 | 2.47E-39 | down |
| Arhgef19 | -1.511760025 | 6.95E-41 | 3.12E-39 | down |
| 2310007B03Rik | -3.726920701 | 8.70E-41 | 3.89E-39 | down |
| Ccl11 | -3.270532418 | 1.05E-40 | 4.67E-39 | down |
| Slc9a3r1 | -1.225179198 | 1.24E-40 | 5.51E-39 | down |
| Tle4 | -2.09949699 | 1.67E-40 | 7.39E-39 | down |
| Slc6a8 | -1.223987099 | 1.83E-40 | 8.01E-39 | down |
| Fam19a5 | -3.044955442 | 2.27E-40 | 9.79E-39 | down |
| Glb1l | -1.476771863 | 4.71E-40 | 2.02E-38 | down |
| Zfp462 | -1.880038671 | 7.40E-40 | 3.17E-38 | down |
| Rtkn | -1.408851253 | 9.54E-40 | 4.07E-38 | down |
| Rassf4 | -1.758821412 | 2.56E-39 | 1.09E-37 | down |
| Tagln2 | -1.722472218 | 3.33E-39 | 1.40E-37 | down |
| Icam1 | -1.822248967 | 5.96E-39 | 2.49E-37 | down |
| 2010300C02Rik | -3.824105847 | 5.97E-39 | 2.49E-37 | down |
| Slc43a2 | -1.788999096 | 6.40E-39 | 2.66E-37 | down |
| Fam221a | -2.44543335 | 7.64E-39 | 3.17E-37 | down |
| Alcam | -1.40520414 | 1.02E-38 | 4.22E-37 | down |
| Krt19 | -2.727724042 | 1.34E-38 | 5.46E-37 | down |
| Thrsp | -2.670473816 | 1.78E-38 | 7.21E-37 | down |
| Trnp1 | -4.162287436 | 4.08E-38 | 1.64E-36 | down |
| Cd44 | -2.088420248 | 4.65E-38 | 1.87E-36 | down |
| Rab33b | -1.16009085 | 5.51E-38 | 2.19E-36 | down |
| Vangl1 | -1.147298354 | 6.88E-38 | 2.71E-36 | down |
| 5730559C18Rik | -4.301418797 | 8.73E-38 | 3.43E-36 | down |
| Sdc4 | -1.514346628 | 1.68E-37 | 6.52E-36 | down |
| Lrig3 | -1.676674432 | 1.77E-37 | 6.85E-36 | down |
| B4galnt1 | -1.235463758 | 1.78E-37 | 6.88E-36 | down |
| Lrfn3 | -2.878350071 | 2.03E-37 | 7.78E-36 | down |
| Sstr3 | -4.825429478 | 2.09E-37 | 7.98E-36 | down |
| Crlf3 | -1.326828291 | 2.11E-37 | 8.05E-36 | down |
| Nceh1 | -1.603876501 | 2.96E-37 | 1.13E-35 | down |
| Fggy | -2.145080213 | 4.25E-37 | 1.59E-35 | down |
| Lrfn4 | -1.380109135 | 6.04E-37 | 2.26E-35 | down |
| Ebp | -1.44319687 | 7.31E-37 | 2.71E-35 | down |
| Tcn2 | -2.109199273 | 7.73E-37 | 2.86E-35 | down |
| Slc22a18 | -3.041094737 | 8.04E-37 | 2.97E-35 | down |
| Gatm | -1.73131202 | 1.44E-36 | 5.26E-35 | down |
| Rnase1 | -5.533508955 | 1.72E-36 | 6.30E-35 | down |
| Cercam | -1.521315288 | 2.30E-36 | 8.37E-35 | down |
| 9130017N09Rik | -4.98029289 | 2.34E-36 | 8.50E-35 | down |
| Timp2 | -2.125988465 | 3.12E-36 | 1.13E-34 | down |
| Gem | -1.806328205 | 5.29E-36 | 1.91E-34 | down |
| Dennd2d | -1.420828006 | 5.42E-36 | 1.95E-34 | down |
| Kif3c | -1.504817268 | 7.07E-36 | 2.53E-34 | down |
| S100a14 | -4.532293011 | 7.65E-36 | 2.73E-34 | down |
| Unc5c | -3.427140139 | 1.20E-35 | 4.24E-34 | down |
| Enho | -2.604791533 | 1.31E-35 | 4.64E-34 | down |
| Cd81 | -1.676847061 | 1.37E-35 | 4.84E-34 | down |
| Pbxip1 | -1.494153832 | 1.54E-35 | 5.42E-34 | down |
| Fut9 | -4.843964989 | 1.58E-35 | 5.56E-34 | down |
| Cxcl16 | -1.886059406 | 1.67E-35 | 5.85E-34 | down |
| Fhad1 | -5.154328557 | 1.87E-35 | 6.53E-34 | down |
| Sec11c | -1.456532394 | 2.47E-35 | 8.54E-34 | down |
| Nme7 | -1.357946158 | 2.84E-35 | 9.80E-34 | down |
| Nfkbie | -2.349703002 | 3.64E-35 | 1.25E-33 | down |
| Il18 | -3.817554573 | 3.77E-35 | 1.29E-33 | down |
| Ppp1r3c | -2.565781433 | 4.75E-35 | 1.62E-33 | down |
| Prkag2 | -1.278634557 | 5.02E-35 | 1.71E-33 | down |
| Slc44a4 | -2.646945618 | 5.64E-35 | 1.91E-33 | down |
| Vps37d | -3.311279555 | 6.17E-35 | 2.09E-33 | down |
| Kcnc2 | -2.95768689 | 7.08E-35 | 2.38E-33 | down |
| Il13ra1 | -1.302520342 | 1.01E-34 | 3.36E-33 | down |
| Srrm4 | -6.143710371 | 1.07E-34 | 3.56E-33 | down |
| Phyhd1 | -2.291032241 | 1.18E-34 | 3.92E-33 | down |
| Fam65c | -3.359505559 | 1.25E-34 | 4.11E-33 | down |
| Cx3cl1 | -1.63780532 | 1.28E-34 | 4.22E-33 | down |
| Usp35 | -2.155264073 | 1.36E-34 | 4.47E-33 | down |
| C77080 | -1.525730497 | 1.41E-34 | 4.61E-33 | down |
| Sh3bp1 | -2.204856449 | 1.45E-34 | 4.73E-33 | down |
| AU021092 | -2.729518529 | 1.48E-34 | 4.83E-33 | down |
| Edn1 | -2.03719114 | 1.49E-34 | 4.84E-33 | down |
| Traf1 | -3.054491114 | 1.53E-34 | 4.95E-33 | down |
| Marveld1 | -1.295024841 | 1.56E-34 | 5.04E-33 | down |
| Col9a3 | -2.761069178 | 2.10E-34 | 6.77E-33 | down |
| Tmem51 | -1.478497662 | 2.11E-34 | 6.80E-33 | down |
| Cant1 | -1.035597931 | 6.88E-34 | 2.15E-32 | down |
| Plch2 | -2.548040717 | 7.33E-34 | 2.27E-32 | down |
| Mt2 | -2.629427812 | 7.29E-34 | 2.27E-32 | down |
| Tap2 | -1.519210175 | 8.81E-34 | 2.72E-32 | down |
| Tlr2 | -1.652276012 | 1.02E-33 | 3.16E-32 | down |
| Gal3st3 | -4.291235037 | 4.11E-33 | 1.24E-31 | down |
| Snph | -1.784153678 | 8.29E-33 | 2.49E-31 | down |
| Lta | -1.706842493 | 9.10E-33 | 2.73E-31 | down |
| Gpx8 | -1.835832367 | 1.02E-32 | 3.06E-31 | down |
| Ms4a7 | -1.941786766 | 1.09E-32 | 3.26E-31 | down |
| Scnn1a | -1.258172865 | 1.13E-32 | 3.38E-31 | down |
| Cdc42ep5 | -2.304432048 | 1.20E-32 | 3.57E-31 | down |
| Npr2 | -1.722686577 | 1.47E-32 | 4.33E-31 | down |
| Rasa4 | -1.641333139 | 1.70E-32 | 4.98E-31 | down |
| Id2 | -1.967730845 | 1.73E-32 | 5.06E-31 | down |
| Bik | -2.300130387 | 1.88E-32 | 5.49E-31 | down |
| Il1rap | -1.494761112 | 2.45E-32 | 7.12E-31 | down |
| Tnfsf13 | -2.970535456 | 2.58E-32 | 7.47E-31 | down |
| Car8 | -3.325373458 | 3.47E-32 | 1.00E-30 | down |
| Stxbp6 | -1.610100631 | 4.21E-32 | 1.21E-30 | down |
| Smpd3 | -2.206519811 | 4.33E-32 | 1.24E-30 | down |
| Shisa4 | -1.292797176 | 4.56E-32 | 1.30E-30 | down |
| Plekha2 | -1.243271284 | 4.94E-32 | 1.41E-30 | down |
| Scn3a | -3.328029767 | 7.12E-32 | 2.03E-30 | down |
| Phlda3 | -1.370122788 | 7.32E-32 | 2.08E-30 | down |
| Kit | -1.763582032 | 7.36E-32 | 2.09E-30 | down |
| Cyba | -1.912512943 | 7.81E-32 | 2.21E-30 | down |
| Tmem63a | -1.083924394 | 1.05E-31 | 2.96E-30 | down |
| Prune2 | -2.705911973 | 1.31E-31 | 3.68E-30 | down |
| Amigo1 | -1.453640791 | 1.54E-31 | 4.30E-30 | down |
| Unc5cl | -1.899573517 | 1.99E-31 | 5.55E-30 | down |
| Gbp8 | -5.348331312 | 2.06E-31 | 5.76E-30 | down |
| Dusp18 | -1.820896737 | 2.98E-31 | 8.27E-30 | down |
| Rragd | -2.085143515 | 5.24E-31 | 1.44E-29 | down |
| Bnipl | -3.17805133 | 5.69E-31 | 1.56E-29 | down |
| Ephb3 | -1.841032944 | 6.40E-31 | 1.75E-29 | down |
| Rabgap1l | -1.140163796 | 6.53E-31 | 1.78E-29 | down |
| Sv2a | -3.359068496 | 7.04E-31 | 1.92E-29 | down |
| Fam92a | -1.25142095 | 9.49E-31 | 2.58E-29 | down |
| Arhgef6 | -1.902155039 | 9.82E-31 | 2.66E-29 | down |
| Samd9l | -1.332943985 | 1.35E-30 | 3.64E-29 | down |
| Lamb3 | -2.71250542 | 1.55E-30 | 4.16E-29 | down |
| A230050P20Rik | -2.073634518 | 1.58E-30 | 4.22E-29 | down |
| Aplp1 | -1.965907345 | 1.80E-30 | 4.82E-29 | down |
| Prr15l | -1.624608685 | 1.89E-30 | 5.02E-29 | down |
| Csrp1 | -1.645461191 | 2.43E-30 | 6.42E-29 | down |
| Gadd45b | -1.953579217 | 2.43E-30 | 6.42E-29 | down |
| Naprt1 | -1.795097884 | 4.52E-30 | 1.19E-28 | down |
| Hdac7 | -1.164869847 | 6.28E-30 | 1.64E-28 | down |
| Ephx4 | -4.362768689 | 7.58E-30 | 1.97E-28 | down |
| Ociad2 | -1.503620845 | 9.39E-30 | 2.44E-28 | down |
| Esrrb | -2.762139753 | 1.10E-29 | 2.85E-28 | down |
| Dlgap4 | -1.082942741 | 1.10E-29 | 2.85E-28 | down |
| Muc20 | -2.93021828 | 1.14E-29 | 2.93E-28 | down |
| Abtb2 | -1.672511691 | 1.88E-29 | 4.79E-28 | down |
| Fam181b | -2.827454014 | 1.93E-29 | 4.91E-28 | down |
| Flot2 | -1.017728282 | 2.36E-29 | 5.96E-28 | down |
| Ocln | -2.085260404 | 3.20E-29 | 8.04E-28 | down |
| Irs3 | -2.156620226 | 3.27E-29 | 8.17E-28 | down |
| Gpcpd1 | -1.285432528 | 3.74E-29 | 9.33E-28 | down |
| Nipal2 | -1.16035131 | 3.75E-29 | 9.34E-28 | down |
| Sertm1 | -7.197341657 | 3.77E-29 | 9.36E-28 | down |
| Ankrd33b | -1.748664832 | 3.89E-29 | 9.66E-28 | down |
| Inha | -3.461266415 | 4.05E-29 | 1.00E-27 | down |
| Glt25d2 | -1.233380437 | 4.50E-29 | 1.11E-27 | down |
| Tcea3 | -3.880675873 | 4.72E-29 | 1.16E-27 | down |
| S100a16 | -1.398732739 | 4.79E-29 | 1.18E-27 | down |
| Cplx2 | -2.113533958 | 6.06E-29 | 1.48E-27 | down |
| BC064078 | -3.222611396 | 6.33E-29 | 1.54E-27 | down |
| Ccrl1 | -2.898861319 | 7.03E-29 | 1.71E-27 | down |
| Mapk4 | -2.177424111 | 7.63E-29 | 1.85E-27 | down |
| Ggact | -1.325594373 | 9.31E-29 | 2.24E-27 | down |
| Atxn1 | -1.138317802 | 1.12E-28 | 2.69E-27 | down |
| Hsd3b7 | -1.630275388 | 1.61E-28 | 3.81E-27 | down |
| Tubb6 | -1.457393376 | 1.64E-28 | 3.89E-27 | down |
| B3galt5 | -2.531004339 | 1.66E-28 | 3.92E-27 | down |
| Gli2 | -2.501195496 | 1.72E-28 | 4.07E-27 | down |
| Napsa | -2.048940802 | 2.06E-28 | 4.84E-27 | down |
| Idua | -1.314786347 | 2.57E-28 | 6.02E-27 | down |
| Crip1 | -1.940227318 | 5.10E-28 | 1.19E-26 | down |
| Csdc2 | -2.724912091 | 5.14E-28 | 1.19E-26 | down |
| Cuedc1 | -1.14129076 | 5.51E-28 | 1.28E-26 | down |
| Clcnka | -2.46373997 | 5.77E-28 | 1.34E-26 | down |
| Sord | -1.664434535 | 5.85E-28 | 1.35E-26 | down |
| Prnp | -1.495089179 | 6.56E-28 | 1.51E-26 | down |
| Capg | -1.245207384 | 7.18E-28 | 1.65E-26 | down |
| Tmc4 | -1.289496848 | 7.24E-28 | 1.66E-26 | down |
| D430019H16Rik | -2.439417014 | 7.86E-28 | 1.79E-26 | down |
| Sorbs3 | -1.117431449 | 7.97E-28 | 1.82E-26 | down |
| Aatk | -1.776919142 | 1.02E-27 | 2.31E-26 | down |
| Gltp | -1.121277288 | 1.20E-27 | 2.71E-26 | down |
| Efhd2 | -1.295378497 | 1.36E-27 | 3.07E-26 | down |
| Pml | -1.021206477 | 1.54E-27 | 3.47E-26 | down |
| Fbxo6 | -1.136386766 | 1.62E-27 | 3.64E-26 | down |
| Serpinb9 | -2.337970672 | 2.19E-27 | 4.90E-26 | down |
| Osgin2 | -1.585411556 | 2.37E-27 | 5.29E-26 | down |
| Heph | -1.650416451 | 2.38E-27 | 5.30E-26 | down |
| Pycard | -1.647373607 | 2.90E-27 | 6.44E-26 | down |
| Myl12b | -1.099663474 | 3.94E-27 | 8.67E-26 | down |
| Chad | -2.36176077 | 4.71E-27 | 1.03E-25 | down |
| Rgn | -3.15619743 | 5.03E-27 | 1.10E-25 | down |
| Arpc1b | -1.323144215 | 5.95E-27 | 1.30E-25 | down |
| Tcta | -1.014004148 | 6.00E-27 | 1.30E-25 | down |
| Klhl32 | -2.509446437 | 9.70E-27 | 2.10E-25 | down |
| Cpa4 | -4.505124507 | 9.73E-27 | 2.10E-25 | down |
| Chrnb1 | -1.578018238 | 1.26E-26 | 2.71E-25 | down |
| Enpp5 | -1.099082022 | 1.27E-26 | 2.74E-25 | down |
| Gyg | -1.004756219 | 1.30E-26 | 2.80E-25 | down |
| Il15 | -2.06834709 | 1.32E-26 | 2.83E-25 | down |
| Tmem184a | -1.105617767 | 1.36E-26 | 2.90E-25 | down |
| Ctsb | -1.578840163 | 1.43E-26 | 3.06E-25 | down |
| Nrbp2 | -1.319427523 | 1.47E-26 | 3.14E-25 | down |
| Pla2g4f | -4.149068651 | 1.62E-26 | 3.46E-25 | down |
| Agr2 | -2.100873696 | 2.25E-26 | 4.77E-25 | down |
| Smim5 | -2.513905696 | 2.30E-26 | 4.86E-25 | down |
| Rab27a | -2.054834792 | 2.44E-26 | 5.15E-25 | down |
| Irf5 | -1.97124946 | 2.57E-26 | 5.42E-25 | down |
| Emilin2 | -1.884621883 | 3.17E-26 | 6.64E-25 | down |
| Atp1b1 | -1.617767082 | 3.62E-26 | 7.56E-25 | down |
| 2610028H24Rik | -4.821342666 | 3.93E-26 | 8.17E-25 | down |
| Cx3cr1 | -1.58669589 | 4.06E-26 | 8.43E-25 | down |
| Il17re | -1.371188539 | 4.21E-26 | 8.73E-25 | down |
| Slc28a3 | -3.179765721 | 4.59E-26 | 9.49E-25 | down |
| Sfxn3 | -1.065869962 | 4.81E-26 | 9.92E-25 | down |
| Serpinb6b | -2.670614903 | 5.00E-26 | 1.03E-24 | down |
| Smoc2 | -2.733308881 | 8.21E-26 | 1.67E-24 | down |
| Rrad | -2.485589157 | 9.67E-26 | 1.96E-24 | down |
| 4933431E20Rik | -1.68911167 | 1.67E-25 | 3.34E-24 | down |
| Rab19 | -1.932019132 | 1.88E-25 | 3.77E-24 | down |
| Rgcc | -2.781824405 | 2.14E-25 | 4.26E-24 | down |
| Fxyd4 | -2.22380544 | 2.43E-25 | 4.82E-24 | down |
| Mif4gd | -1.018728278 | 2.44E-25 | 4.84E-24 | down |
| Sh3yl1 | -1.372293139 | 2.77E-25 | 5.46E-24 | down |
| Sla | -2.200654089 | 2.80E-25 | 5.51E-24 | down |
| Porcn | -1.310541123 | 3.71E-25 | 7.25E-24 | down |
| Trpv4 | -1.587234881 | 3.93E-25 | 7.68E-24 | down |
| Rbm43 | -1.23475038 | 4.61E-25 | 8.97E-24 | down |
| 1110051M20Rik | -1.527254525 | 4.92E-25 | 9.52E-24 | down |
| Abca8b | -1.683511665 | 5.67E-25 | 1.09E-23 | down |
| Zfp605 | -1.088883862 | 6.01E-25 | 1.15E-23 | down |
| Fzd2 | -1.279101363 | 6.80E-25 | 1.30E-23 | down |
| Tmprss2 | -1.476788687 | 7.64E-25 | 1.45E-23 | down |
| Nfkb2 | -1.012779991 | 9.44E-25 | 1.78E-23 | down |
| Zcchc12 | -4.294219272 | 9.63E-25 | 1.81E-23 | down |
| Pls3 | -1.09578623 | 1.09E-24 | 2.05E-23 | down |
| Plekhb1 | -1.275298346 | 1.62E-24 | 3.01E-23 | down |
| Ank2 | -1.91952953 | 2.02E-24 | 3.74E-23 | down |
| Lrtm2 | -4.238042887 | 2.04E-24 | 3.78E-23 | down |
| D7Ertd443e | -3.497464956 | 2.36E-24 | 4.35E-23 | down |
| Sema4a | -2.012097211 | 2.39E-24 | 4.42E-23 | down |
| H2afy2 | -2.178489205 | 2.63E-24 | 4.83E-23 | down |
| Kif26a | -1.476267122 | 2.67E-24 | 4.89E-23 | down |
| Efcab4a | -1.284655345 | 3.02E-24 | 5.52E-23 | down |
| Rasd2 | -3.805793042 | 3.46E-24 | 6.32E-23 | down |
| Dram1 | -1.733314791 | 3.57E-24 | 6.50E-23 | down |
| Kctd6 | -1.465320874 | 3.95E-24 | 7.19E-23 | down |
| Rem2 | -3.353222254 | 4.40E-24 | 7.98E-23 | down |
| Kcnk1 | -1.327075123 | 4.59E-24 | 8.28E-23 | down |
| Marveld3 | -1.213605879 | 4.63E-24 | 8.35E-23 | down |
| Ttc9 | -2.516243085 | 4.88E-24 | 8.78E-23 | down |
| Abhd14b | -1.202690564 | 7.89E-24 | 1.41E-22 | down |
| Rras | -1.284746456 | 9.67E-24 | 1.72E-22 | down |
| Axin2 | -2.349649137 | 1.08E-23 | 1.92E-22 | down |
| Gm10635 | -3.515218302 | 1.28E-23 | 2.26E-22 | down |
| Klhdc8b | -1.536298372 | 1.30E-23 | 2.30E-22 | down |
| H2-K1 | -1.596717164 | 1.40E-23 | 2.46E-22 | down |
| Cyp4v3 | -1.579594935 | 1.50E-23 | 2.64E-22 | down |
| Tmem176a | -1.189553264 | 1.52E-23 | 2.67E-22 | down |
| Dnase1l1 | -1.126471053 | 1.55E-23 | 2.71E-22 | down |
| Lmo3 | -2.961127089 | 1.62E-23 | 2.84E-22 | down |
| Pik3cd | -1.640878404 | 1.79E-23 | 3.12E-22 | down |
| Gm15706 | -2.617398453 | 2.20E-23 | 3.83E-22 | down |
| Mgat4a | -1.072310543 | 2.33E-23 | 4.04E-22 | down |
| Efna4 | -1.381358949 | 2.34E-23 | 4.05E-22 | down |
| Ten1 | -1.1980206 | 2.41E-23 | 4.17E-22 | down |
| Mboat2 | -1.436388939 | 2.76E-23 | 4.77E-22 | down |
| Camk1 | -1.046411468 | 2.77E-23 | 4.79E-22 | down |
| Trim47 | -1.419713712 | 3.73E-23 | 6.43E-22 | down |
| C1qtnf1 | -1.626569692 | 4.70E-23 | 8.04E-22 | down |
| Them5 | -3.474275157 | 5.53E-23 | 9.42E-22 | down |
| 2610034B18Rik | -1.185882708 | 5.60E-23 | 9.53E-22 | down |
| Atp13a5 | -4.549123508 | 5.64E-23 | 9.57E-22 | down |
| Cpeb1 | -1.388263369 | 5.75E-23 | 9.76E-22 | down |
| Rsph9 | -2.124648186 | 6.44E-23 | 1.09E-21 | down |
| Asgr1 | -2.396055303 | 6.66E-23 | 1.13E-21 | down |
| Krt80 | -3.175171602 | 6.67E-23 | 1.13E-21 | down |
| B9d1 | -1.330682801 | 6.87E-23 | 1.16E-21 | down |
| Evc2 | -1.942426465 | 6.95E-23 | 1.17E-21 | down |
| Aass | -3.262232533 | 7.64E-23 | 1.28E-21 | down |
| Ctss | -1.53692534 | 7.95E-23 | 1.33E-21 | down |
| Mgam | -3.902872765 | 1.06E-22 | 1.78E-21 | down |
| Mtmr11 | -1.209085227 | 1.23E-22 | 2.05E-21 | down |
| Cradd | -1.41824213 | 1.30E-22 | 2.16E-21 | down |
| Slc31a2 | -1.079351212 | 1.34E-22 | 2.22E-21 | down |
| St6gal1 | -1.815122701 | 1.34E-22 | 2.22E-21 | down |
| Ptgds | -3.314597038 | 1.49E-22 | 2.47E-21 | down |
| Ptpru | -1.935443535 | 1.57E-22 | 2.58E-21 | down |
| Eva1a | -1.225245655 | 1.72E-22 | 2.84E-21 | down |
| Arhgef40 | -1.114062842 | 2.25E-22 | 3.67E-21 | down |
| Clu | -2.395636622 | 2.28E-22 | 3.71E-21 | down |
| Bhlhe41 | -1.282465552 | 2.39E-22 | 3.88E-21 | down |
| Car13 | -1.30419062 | 2.45E-22 | 3.97E-21 | down |
| Dpysl2 | -1.428049911 | 2.67E-22 | 4.33E-21 | down |
| Prdm5 | -2.265005531 | 2.86E-22 | 4.60E-21 | down |
| Myl9 | -1.557918332 | 2.99E-22 | 4.81E-21 | down |
| Antxr2 | -1.265157677 | 3.46E-22 | 5.55E-21 | down |
| Hspb8 | -2.711624144 | 3.57E-22 | 5.71E-21 | down |
| H2-DMb1 | -1.650236447 | 3.57E-22 | 5.72E-21 | down |
| Avpi1 | -1.299093879 | 4.36E-22 | 6.93E-21 | down |
| Foxf2 | -2.005337794 | 4.44E-22 | 7.05E-21 | down |
| Dnahc10 | -2.534633648 | 5.05E-22 | 7.98E-21 | down |
| Entpd2 | -1.914221773 | 5.12E-22 | 8.08E-21 | down |
| Egfl6 | -2.664504326 | 5.72E-22 | 9.00E-21 | down |
| D630045J12Rik | -1.413631377 | 5.75E-22 | 9.03E-21 | down |
| Nudt14 | -1.305730652 | 6.06E-22 | 9.51E-21 | down |
| Tmem255a | -2.14804841 | 6.13E-22 | 9.61E-21 | down |
| Maf | -1.244627631 | 7.58E-22 | 1.18E-20 | down |
| Rps6ka1 | -1.003822929 | 9.83E-22 | 1.52E-20 | down |
| Fhl2 | -1.591093366 | 1.01E-21 | 1.56E-20 | down |
| Abhd6 | -1.599614265 | 1.07E-21 | 1.65E-20 | down |
| Lphn1 | -1.38016998 | 1.15E-21 | 1.77E-20 | down |
| Nt5e | -1.869813286 | 1.25E-21 | 1.93E-20 | down |
| Eps8l1 | -1.371084025 | 1.34E-21 | 2.06E-20 | down |
| Scg3 | -4.729596226 | 1.37E-21 | 2.09E-20 | down |
| Creb3 | -1.00827764 | 1.98E-21 | 3.00E-20 | down |
| Slc29a1 | -1.30643103 | 2.18E-21 | 3.28E-20 | down |
| Kctd15 | -2.404958203 | 2.19E-21 | 3.30E-20 | down |
| Hhatl | -3.987521525 | 2.40E-21 | 3.60E-20 | down |
| Ankrd29 | -2.08129209 | 2.61E-21 | 3.90E-20 | down |
| Tspo | -1.000891077 | 2.64E-21 | 3.93E-20 | down |
| Bco2 | -2.973943092 | 2.76E-21 | 4.10E-20 | down |
| Lpl | -2.297795345 | 3.25E-21 | 4.80E-20 | down |
| Lefty2 | -3.11022524 | 3.47E-21 | 5.12E-20 | down |
| Lamb2 | -1.099389034 | 4.63E-21 | 6.79E-20 | down |
| Slc25a29 | -1.164023334 | 4.70E-21 | 6.88E-20 | down |
| Acpp | -3.014711049 | 5.05E-21 | 7.39E-20 | down |
| Tmem194b | -1.756869822 | 5.36E-21 | 7.82E-20 | down |
| Ppapdc1b | -1.198612301 | 5.67E-21 | 8.27E-20 | down |
| Ctsh | -1.740661911 | 5.73E-21 | 8.36E-20 | down |
| Has3 | -2.096783556 | 6.49E-21 | 9.40E-20 | down |
| Ntf5 | -1.774096418 | 6.50E-21 | 9.41E-20 | down |
| Cldn8 | -2.028315482 | 8.05E-21 | 1.16E-19 | down |
| Igsf3 | -1.169633305 | 9.70E-21 | 1.39E-19 | down |
| Nek3 | -1.544571825 | 1.02E-20 | 1.46E-19 | down |
| Spib | -3.13502935 | 1.16E-20 | 1.65E-19 | down |
| Cited4 | -1.288570516 | 1.26E-20 | 1.79E-19 | down |
| Fgd2 | -2.014635352 | 1.37E-20 | 1.95E-19 | down |
| Slc44a3 | -1.278486854 | 1.39E-20 | 1.98E-19 | down |
| Pdxp | -1.989110201 | 1.41E-20 | 2.00E-19 | down |
| Adssl1 | -1.741434456 | 1.43E-20 | 2.02E-19 | down |
| Rab4a | -1.070880464 | 1.74E-20 | 2.46E-19 | down |
| Pde4a | -1.656360961 | 1.79E-20 | 2.52E-19 | down |
| Ece1 | -1.096884471 | 2.32E-20 | 3.25E-19 | down |
| Aifm3 | -1.610952473 | 2.91E-20 | 4.07E-19 | down |
| Arap2 | -1.433472589 | 3.01E-20 | 4.20E-19 | down |
| Il1rn | -3.468755343 | 3.04E-20 | 4.23E-19 | down |
| Cttnbp2 | -2.313654282 | 3.48E-20 | 4.83E-19 | down |
| Lrrc8e | -1.104880824 | 4.25E-20 | 5.86E-19 | down |
| Arhgap6 | -1.043616639 | 4.54E-20 | 6.24E-19 | down |
| Antxr1 | -1.445092948 | 4.87E-20 | 6.66E-19 | down |
| Ankrd9 | -2.218388335 | 5.40E-20 | 7.37E-19 | down |
| Dhrs7 | -1.225376221 | 6.20E-20 | 8.45E-19 | down |
| C2cd4d | -3.481857385 | 6.47E-20 | 8.80E-19 | down |
| Mmp14 | -1.594950062 | 6.66E-20 | 9.06E-19 | down |
| Il18r1 | -2.269144437 | 6.89E-20 | 9.35E-19 | down |
| Cited2 | -1.024414682 | 6.93E-20 | 9.40E-19 | down |
| Nudt16 | -1.594287161 | 9.74E-20 | 1.31E-18 | down |
| Ccdc19 | -2.166200069 | 1.09E-19 | 1.46E-18 | down |
| Ccdc106 | -2.750604081 | 1.37E-19 | 1.83E-18 | down |
| Lgals3 | -1.922565025 | 1.58E-19 | 2.11E-18 | down |
| S100a1 | -1.449995338 | 1.59E-19 | 2.12E-18 | down |
| Hyal3 | -1.83945586 | 1.60E-19 | 2.14E-18 | down |
| Homer2 | -1.90586748 | 1.84E-19 | 2.45E-18 | down |
| Cd24a | -1.420505754 | 2.09E-19 | 2.77E-18 | down |
| S100a11 | -1.504005973 | 2.18E-19 | 2.89E-18 | down |
| C1qb | -1.201494133 | 2.41E-19 | 3.18E-18 | down |
| Camk2a | -3.129838725 | 2.64E-19 | 3.48E-18 | down |
| Jun | -1.403906697 | 2.69E-19 | 3.53E-18 | down |
| Bcam | -1.23932688 | 2.96E-19 | 3.89E-18 | down |
| Galnt5 | -2.068279039 | 3.16E-19 | 4.12E-18 | down |
| Tmem237 | -1.00540979 | 3.22E-19 | 4.21E-18 | down |
| Gpr153 | -1.551019054 | 3.27E-19 | 4.26E-18 | down |
| Zfp707 | -1.253107573 | 3.42E-19 | 4.46E-18 | down |
| Spata6 | -1.016700101 | 3.54E-19 | 4.61E-18 | down |
| Plekhd1 | -1.197717479 | 4.02E-19 | 5.21E-18 | down |
| Slc7a4 | -1.369041254 | 4.06E-19 | 5.25E-18 | down |
| Sec14l5 | -2.270683269 | 4.11E-19 | 5.31E-18 | down |
| Col6a1 | -1.517466172 | 4.19E-19 | 5.41E-18 | down |
| Ifih1 | -1.443364495 | 4.29E-19 | 5.53E-18 | down |
| D930048N14Rik | -1.267917722 | 4.41E-19 | 5.67E-18 | down |
| Praf2 | -1.140631349 | 4.56E-19 | 5.85E-18 | down |
| Enkur | -2.136858662 | 4.67E-19 | 5.97E-18 | down |
| Scn3b | -2.075085268 | 5.18E-19 | 6.59E-18 | down |
| Iyd | -1.35296364 | 5.28E-19 | 6.71E-18 | down |
| Ptpn2 | -1.107527924 | 5.36E-19 | 6.81E-18 | down |
| Epcam | -1.082990117 | 5.58E-19 | 7.07E-18 | down |
| Mast3 | -1.00120256 | 6.10E-19 | 7.69E-18 | down |
| C920025E04Rik | -1.578680968 | 9.93E-19 | 1.24E-17 | down |
| Slc25a18 | -3.202460283 | 1.22E-18 | 1.52E-17 | down |
| Aldh5a1 | -1.375963163 | 1.23E-18 | 1.53E-17 | down |
| Zfp651 | -1.04904296 | 1.28E-18 | 1.58E-17 | down |
| Mpped2 | -1.466261609 | 1.58E-18 | 1.95E-17 | down |
| Rab17 | -1.93465969 | 1.71E-18 | 2.10E-17 | down |
| Mmp15 | -1.050443394 | 1.78E-18 | 2.19E-17 | down |
| Ptk2b | -1.038109109 | 1.84E-18 | 2.27E-17 | down |
| Tff2 | -7.261569821 | 1.89E-18 | 2.32E-17 | down |
| Slc1a3 | -1.405041723 | 1.98E-18 | 2.43E-17 | down |
| Cdsn | -2.801058007 | 2.14E-18 | 2.62E-17 | down |
| S100a13 | -1.16739955 | 2.16E-18 | 2.64E-17 | down |
| Pllp | -2.041555809 | 2.17E-18 | 2.64E-17 | down |
| Ifi35 | -1.102393747 | 2.20E-18 | 2.68E-17 | down |
| Crip2 | -1.142605237 | 2.27E-18 | 2.76E-17 | down |
| Lrrc26 | -3.466231403 | 2.44E-18 | 2.95E-17 | down |
| Dpep1 | -1.783558247 | 2.53E-18 | 3.06E-17 | down |
| B4galt2 | -1.402436284 | 2.55E-18 | 3.08E-17 | down |
| Slc43a3 | -1.202085239 | 2.56E-18 | 3.09E-17 | down |
| Krt18 | -1.807252176 | 2.61E-18 | 3.14E-17 | down |
| Bfsp2 | -3.857076097 | 2.77E-18 | 3.34E-17 | down |
| C1qc | -1.15463617 | 2.79E-18 | 3.35E-17 | down |
| Matn4 | -1.85621136 | 3.11E-18 | 3.73E-17 | down |
| Tmem107 | -1.300988537 | 3.81E-18 | 4.56E-17 | down |
| Adprm | -1.038016711 | 3.92E-18 | 4.67E-17 | down |
| Cxcl10 | -3.397010743 | 3.93E-18 | 4.68E-17 | down |
| Mxra8 | -1.198097459 | 3.97E-18 | 4.72E-17 | down |
| E030019B13Rik | -1.403468899 | 4.13E-18 | 4.91E-17 | down |
| Foxred2 | -1.650824596 | 5.16E-18 | 6.09E-17 | down |
| Gpr179 | -2.635847846 | 5.73E-18 | 6.74E-17 | down |
| Hpcal4 | -4.45939942 | 5.96E-18 | 6.99E-17 | down |
| Gm128 | -3.421462889 | 5.97E-18 | 7.00E-17 | down |
| Tekt4 | -1.758929523 | 6.29E-18 | 7.35E-17 | down |
| Phlda2 | -3.384017765 | 6.76E-18 | 7.90E-17 | down |
| Dagla | -1.167588927 | 6.86E-18 | 8.00E-17 | down |
| Csf1r | -1.025120813 | 7.02E-18 | 8.17E-17 | down |
| Fhod3 | -1.992077482 | 7.06E-18 | 8.22E-17 | down |
| Gm5141 | -2.092675379 | 7.31E-18 | 8.50E-17 | down |
| Wnt4 | -1.30473689 | 7.86E-18 | 9.11E-17 | down |
| Rdm1 | -1.345674948 | 1.05E-17 | 1.21E-16 | down |
| Atf5 | -1.028933635 | 1.15E-17 | 1.33E-16 | down |
| Whrn | -1.449131461 | 1.15E-17 | 1.33E-16 | down |
| Pak3 | -1.36417813 | 1.42E-17 | 1.63E-16 | down |
| Sgpl1 | -1.08618663 | 1.46E-17 | 1.67E-16 | down |
| Ccdc136 | -1.986625634 | 1.51E-17 | 1.73E-16 | down |
| Htr1d | -6.125722806 | 1.58E-17 | 1.80E-16 | down |
| Pqlc3 | -1.317413915 | 1.58E-17 | 1.81E-16 | down |
| Tyrobp | -1.30112088 | 1.72E-17 | 1.97E-16 | down |
| Tmem86a | -1.034148305 | 1.81E-17 | 2.06E-16 | down |
| Dyrk3 | -1.492575767 | 1.96E-17 | 2.22E-16 | down |
| Myb | -3.862275064 | 2.00E-17 | 2.26E-16 | down |
| Vtcn1 | -3.853158955 | 2.21E-17 | 2.49E-16 | down |
| Etv5 | -1.33712029 | 2.23E-17 | 2.51E-16 | down |
| Slc37a2 | -1.980191017 | 2.36E-17 | 2.66E-16 | down |
| Slc4a3 | -1.083150853 | 2.43E-17 | 2.72E-16 | down |
| Rhbdf2 | -1.128970886 | 2.44E-17 | 2.74E-16 | down |
| Blnk | -1.191218504 | 2.52E-17 | 2.82E-16 | down |
| Snhg11 | -3.44581519 | 2.97E-17 | 3.29E-16 | down |
| Nenf | -1.122020302 | 3.13E-17 | 3.47E-16 | down |
| Def6 | -1.762903158 | 3.26E-17 | 3.60E-16 | down |
| Slc25a24 | -1.304974909 | 3.76E-17 | 4.15E-16 | down |
| Tcp11 | -3.001885978 | 3.79E-17 | 4.18E-16 | down |
| Itga3 | -1.123672143 | 4.19E-17 | 4.61E-16 | down |
| Trank1 | -1.092747212 | 4.36E-17 | 4.77E-16 | down |
| 6720489N17Rik | -2.12396719 | 4.63E-17 | 5.04E-16 | down |
| Havcr2 | -1.882262496 | 4.82E-17 | 5.25E-16 | down |
| Hnmt | -1.461854257 | 5.01E-17 | 5.45E-16 | down |
| Barx2 | -4.510895937 | 5.36E-17 | 5.81E-16 | down |
| Clcnkb | -2.513697375 | 5.87E-17 | 6.34E-16 | down |
| Gng7 | -1.27213952 | 5.91E-17 | 6.37E-16 | down |
| Ezr | -1.081817329 | 6.15E-17 | 6.63E-16 | down |
| Cd74 | -2.029168925 | 6.19E-17 | 6.67E-16 | down |
| Hepacam | -4.361663718 | 7.33E-17 | 7.86E-16 | down |
| Plekho2 | -1.005362796 | 7.51E-17 | 8.04E-16 | down |
| Gm5480 | -2.372535894 | 7.99E-17 | 8.54E-16 | down |
| Socs2 | -2.070739544 | 8.05E-17 | 8.59E-16 | down |
| Tmem43 | -1.002189275 | 9.39E-17 | 9.99E-16 | down |
| Wisp1 | -1.768574233 | 9.46E-17 | 1.01E-15 | down |
| Prokr2 | -4.307979158 | 1.01E-16 | 1.07E-15 | down |
| Scx | -1.652908488 | 1.04E-16 | 1.10E-15 | down |
| Cers4 | -1.393946787 | 1.06E-16 | 1.12E-15 | down |
| Pcsk1n | -6.04090637 | 1.08E-16 | 1.14E-15 | down |
| Upb1 | -2.278403586 | 1.09E-16 | 1.15E-15 | down |
| Cd83 | -1.874277385 | 1.24E-16 | 1.30E-15 | down |
| Hspa1b | -1.098243548 | 1.30E-16 | 1.37E-15 | down |
| C530008M17Rik | -1.401402899 | 1.40E-16 | 1.47E-15 | down |
| Neat1 | -1.992069346 | 1.41E-16 | 1.48E-15 | down |
| Lgi3 | -2.189870089 | 1.54E-16 | 1.60E-15 | down |
| Pde1c | -1.891573722 | 1.56E-16 | 1.63E-15 | down |
| Lrrc8d | -1.009587703 | 1.60E-16 | 1.67E-15 | down |
| Fndc5 | -4.754508838 | 2.00E-16 | 2.07E-15 | down |
| Dnase2a | -1.165493331 | 2.07E-16 | 2.15E-15 | down |
| Ptms | -1.103867535 | 2.12E-16 | 2.19E-15 | down |
| Gm3604 | -2.029160152 | 2.22E-16 | 2.29E-15 | down |
| Ppp2r2c | -3.74585552 | 2.24E-16 | 2.31E-15 | down |
| Snx32 | -1.718393485 | 2.45E-16 | 2.51E-15 | down |
| Klc3 | -1.312988704 | 2.45E-16 | 2.52E-15 | down |
| Ly6e | -1.360333951 | 2.65E-16 | 2.71E-15 | down |
| Ahnak | -1.089010859 | 2.96E-16 | 3.01E-15 | down |
| Tssk6 | -2.02256122 | 3.09E-16 | 3.14E-15 | down |
| Icosl | -1.171448777 | 3.14E-16 | 3.19E-15 | down |
| Zfp820 | -2.041331086 | 3.18E-16 | 3.23E-15 | down |
| AF251705 | -1.953581453 | 3.31E-16 | 3.36E-15 | down |
| Nrip3 | -2.752504443 | 3.56E-16 | 3.60E-15 | down |
| Upk2 | -3.796613026 | 3.68E-16 | 3.72E-15 | down |
| Dusp14 | -1.157490473 | 3.87E-16 | 3.90E-15 | down |
| Gpm6b | -1.59166372 | 3.97E-16 | 4.00E-15 | down |
| Gpr37l1 | -2.921169992 | 4.06E-16 | 4.09E-15 | down |
| Pla2g5 | -1.338425885 | 4.28E-16 | 4.29E-15 | down |
| Abcc8 | -4.651365983 | 5.46E-16 | 5.45E-15 | down |
| Dkk3 | -1.610635044 | 5.60E-16 | 5.58E-15 | down |
| Nfatc2 | -1.628679923 | 5.85E-16 | 5.81E-15 | down |
| Cdh16 | -2.286003835 | 6.68E-16 | 6.62E-15 | down |
| Slamf9 | -1.520074316 | 7.04E-16 | 6.97E-15 | down |
| Sh3bp2 | -1.242725154 | 7.88E-16 | 7.77E-15 | down |
| Slain1 | -1.385717272 | 8.28E-16 | 8.15E-15 | down |
| C1qa | -1.19909058 | 9.11E-16 | 8.94E-15 | down |
| Pde9a | -1.917003705 | 9.47E-16 | 9.28E-15 | down |
| Dmrta1 | -1.835380218 | 1.00E-15 | 9.79E-15 | down |
| Tnfrsf1b | -1.30833534 | 1.18E-15 | 1.15E-14 | down |
| Fam20c | -1.365689466 | 1.18E-15 | 1.15E-14 | down |
| Slc5a9 | -1.318729788 | 1.21E-15 | 1.18E-14 | down |
| Lipk | -4.134549708 | 1.35E-15 | 1.31E-14 | down |
| Ly6g6e | -1.537661115 | 1.47E-15 | 1.42E-14 | down |
| Greb1l | -1.150165449 | 1.48E-15 | 1.43E-14 | down |
| Trem2 | -1.278457247 | 1.60E-15 | 1.54E-14 | down |
| Scamp5 | -1.174727476 | 1.62E-15 | 1.56E-14 | down |
| Nmi | -1.00832661 | 1.77E-15 | 1.69E-14 | down |
| Myo15 | -3.313462143 | 1.77E-15 | 1.69E-14 | down |
| Zcchc18 | -3.448542587 | 1.83E-15 | 1.74E-14 | down |
| Cpt1c | -1.284177512 | 1.86E-15 | 1.77E-14 | down |
| Gpr34 | -1.618993508 | 1.98E-15 | 1.89E-14 | down |
| Ccdc160 | -1.307886841 | 2.13E-15 | 2.02E-14 | down |
| Cd209a | -2.476868046 | 2.25E-15 | 2.13E-14 | down |
| Celf3 | -4.169437081 | 2.31E-15 | 2.19E-14 | down |
| St14 | -1.165690681 | 2.44E-15 | 2.30E-14 | down |
| Kcnk7 | -2.572253642 | 2.57E-15 | 2.42E-14 | down |
| Esyt3 | -2.540985141 | 2.92E-15 | 2.74E-14 | down |
| Tmem220 | -2.03111797 | 2.97E-15 | 2.78E-14 | down |
| Mypop | -1.570572205 | 3.03E-15 | 2.84E-14 | down |
| Gfra4 | -1.797031764 | 3.14E-15 | 2.93E-14 | down |
| Amotl1 | -1.381266421 | 4.04E-15 | 3.74E-14 | down |
| Ccdc64b | -1.124847411 | 4.36E-15 | 4.01E-14 | down |
| Agr3 | -1.876660728 | 4.61E-15 | 4.24E-14 | down |
| Gpr27 | -3.077704175 | 4.62E-15 | 4.24E-14 | down |
| Anxa9 | -1.414016091 | 4.79E-15 | 4.40E-14 | down |
| Spo11 | -3.661815582 | 4.81E-15 | 4.41E-14 | down |
| Pld4 | -1.271583502 | 4.90E-15 | 4.49E-14 | down |
| Fkbp2 | -1.065279064 | 5.14E-15 | 4.71E-14 | down |
| 4930581F22Rik | -2.196091603 | 5.34E-15 | 4.88E-14 | down |
| Col9a1 | -1.35156969 | 5.52E-15 | 5.04E-14 | down |
| Lair1 | -1.587436386 | 5.71E-15 | 5.19E-14 | down |
| Phf15 | -1.002363265 | 6.02E-15 | 5.47E-14 | down |
| Sfxn5 | -1.124457177 | 6.38E-15 | 5.79E-14 | down |
| Tmem119 | -1.342876164 | 6.39E-15 | 5.79E-14 | down |
| Slc25a42 | -1.206798077 | 6.71E-15 | 6.07E-14 | down |
| Ift27 | -1.279291745 | 7.27E-15 | 6.56E-14 | down |
| Lrrc27 | -1.291143397 | 7.83E-15 | 7.03E-14 | down |
| Selplg | -1.686170839 | 8.00E-15 | 7.17E-14 | down |
| 1190005I06Rik | -1.735714134 | 8.19E-15 | 7.34E-14 | down |
| Rab7l1 | -1.017357838 | 8.48E-15 | 7.58E-14 | down |
| H2-Aa | -1.996110587 | 8.72E-15 | 7.79E-14 | down |
| Cxcl17 | -2.729217654 | 8.74E-15 | 7.80E-14 | down |
| Cav2 | -1.045156476 | 8.82E-15 | 7.87E-14 | down |
| Cdc42ep1 | -1.145983572 | 9.43E-15 | 8.38E-14 | down |
| Ovol2 | -2.157313627 | 9.49E-15 | 8.43E-14 | down |
| Krt84 | -3.461610024 | 9.66E-15 | 8.57E-14 | down |
| Oat | -1.219319983 | 1.01E-14 | 8.94E-14 | down |
| H2-T23 | -1.76942707 | 1.01E-14 | 8.95E-14 | down |
| Nipa1 | -1.24790814 | 1.04E-14 | 9.19E-14 | down |
| Hs3st4 | -5.418338632 | 1.06E-14 | 9.34E-14 | down |
| Ptgfrn | -1.027917891 | 1.06E-14 | 9.37E-14 | down |
| Sult1a1 | -1.450117266 | 1.08E-14 | 9.50E-14 | down |
| Fhdc1 | -1.006506952 | 1.09E-14 | 9.58E-14 | down |
| Perp | -1.209191461 | 1.13E-14 | 9.95E-14 | down |
| Chac2 | -1.2653833 | 1.14E-14 | 1.00E-13 | down |
| Cdh1 | -1.09449594 | 1.15E-14 | 1.01E-13 | down |
| Tmem14a | -1.320404309 | 1.17E-14 | 1.03E-13 | down |
| Cybrd1 | -1.65773182 | 1.59E-14 | 1.38E-13 | down |
| Dpep2 | -1.336351867 | 1.70E-14 | 1.47E-13 | down |
| Gm12295 | -4.180595279 | 1.82E-14 | 1.58E-13 | down |
| Anxa2 | -1.383597364 | 1.83E-14 | 1.58E-13 | down |
| Jund | -1.071395695 | 2.02E-14 | 1.74E-13 | down |
| Lrrc10b | -2.938343651 | 2.13E-14 | 1.83E-13 | down |
| Lgr6 | -3.627382554 | 2.22E-14 | 1.91E-13 | down |
| Cst6 | -2.594894845 | 2.69E-14 | 2.30E-13 | down |
| C630043F03Rik | -1.225465402 | 3.06E-14 | 2.60E-13 | down |
| Fos | -3.500558963 | 3.26E-14 | 2.77E-13 | down |
| 4933407K13Rik | -1.259056739 | 3.27E-14 | 2.78E-13 | down |
| Gpt | -1.368006344 | 3.34E-14 | 2.83E-13 | down |
| Trim7 | -1.143662244 | 3.42E-14 | 2.89E-13 | down |
| Map1a | -1.924839461 | 3.54E-14 | 2.98E-13 | down |
| Klhl23 | -1.338630976 | 3.94E-14 | 3.32E-13 | down |
| Wnt5b | -1.611328348 | 4.13E-14 | 3.47E-13 | down |
| Ccdc60 | -2.782077826 | 4.40E-14 | 3.68E-13 | down |
| Fam107a | -2.28032173 | 4.58E-14 | 3.82E-13 | down |
| Efcab1 | -3.870132949 | 4.59E-14 | 3.83E-13 | down |
| Tmem71 | -1.494242135 | 4.85E-14 | 4.04E-13 | down |
| Nnat | -2.451482553 | 5.42E-14 | 4.51E-13 | down |
| Slc35f3 | -1.127534071 | 5.69E-14 | 4.73E-13 | down |
| H2-DMb2 | -1.550371517 | 5.70E-14 | 4.73E-13 | down |
| Phlda1 | -1.082603987 | 5.74E-14 | 4.76E-13 | down |
| Tmbim1 | -1.010407755 | 5.83E-14 | 4.83E-13 | down |
| Cchcr1 | -1.047481131 | 5.98E-14 | 4.94E-13 | down |
| D10Bwg1379e | -3.884006692 | 6.21E-14 | 5.14E-13 | down |
| B3galtl | -1.124403982 | 6.86E-14 | 5.64E-13 | down |
| Slit2 | -1.059310673 | 7.77E-14 | 6.38E-13 | down |
| Cyp4b1 | -2.251954501 | 7.77E-14 | 6.38E-13 | down |
| Dzip1 | -1.101835015 | 8.56E-14 | 7.01E-13 | down |
| Ikzf1 | -1.939573121 | 8.61E-14 | 7.04E-13 | down |
| Gas6 | -1.262919645 | 9.19E-14 | 7.49E-13 | down |
| Enpp3 | -1.802528968 | 9.19E-14 | 7.49E-13 | down |
| 4932438H23Rik | -1.150599258 | 1.04E-13 | 8.42E-13 | down |
| Evi2a | -1.506502936 | 1.13E-13 | 9.15E-13 | down |
| Rnf217 | -1.31251268 | 1.15E-13 | 9.31E-13 | down |
| Sh3rf2 | -4.220519614 | 1.23E-13 | 9.91E-13 | down |
| Zdhhc15 | -1.163719953 | 1.43E-13 | 1.15E-12 | down |
| Relb | -1.078515843 | 1.45E-13 | 1.17E-12 | down |
| Fam211b | -1.105866657 | 1.46E-13 | 1.18E-12 | down |
| Krt8 | -1.469397808 | 1.47E-13 | 1.18E-12 | down |
| Dcx | -2.72206562 | 2.00E-13 | 1.59E-12 | down |
| Tmem30b | -1.155656754 | 2.07E-13 | 1.65E-12 | down |
| Zfp808 | -1.733424741 | 2.26E-13 | 1.79E-12 | down |
| Resp18 | -5.649885454 | 2.30E-13 | 1.83E-12 | down |
| Ifnlr1 | -1.088678741 | 2.57E-13 | 2.03E-12 | down |
| Strip2 | -1.991916206 | 2.67E-13 | 2.11E-12 | down |
| H2-Ab1 | -1.858124651 | 2.89E-13 | 2.27E-12 | down |
| Lpcat2 | -1.466463024 | 3.00E-13 | 2.35E-12 | down |
| Ric3 | -2.164722113 | 3.26E-13 | 2.54E-12 | down |
| Scube2 | -2.652292132 | 3.28E-13 | 2.56E-12 | down |
| AI414108 | -1.687154045 | 3.66E-13 | 2.84E-12 | down |
| Cdcp1 | -1.472208014 | 3.69E-13 | 2.87E-12 | down |
| B3galt2 | -3.452433831 | 3.93E-13 | 3.04E-12 | down |
| Ulk4 | -1.715598861 | 4.04E-13 | 3.13E-12 | down |
| Fkbp1b | -1.815674526 | 4.15E-13 | 3.21E-12 | down |
| Slc41a3 | -1.697993145 | 4.28E-13 | 3.31E-12 | down |
| Gnal | -1.123784478 | 4.47E-13 | 3.44E-12 | down |
| Retnla | -2.75782731 | 4.55E-13 | 3.50E-12 | down |
| Krtcap3 | -1.116393414 | 4.57E-13 | 3.52E-12 | down |
| Nudt7 | -1.059494786 | 4.79E-13 | 3.67E-12 | down |
| L3mbtl1 | -3.020548228 | 5.31E-13 | 4.06E-12 | down |
| Lypd3 | -2.245728591 | 6.12E-13 | 4.67E-12 | down |
| Gm6623 | -2.324642493 | 6.34E-13 | 4.83E-12 | down |
| Ccdc177 | -2.51033523 | 6.83E-13 | 5.19E-12 | down |
| Lad1 | -1.171151016 | 7.24E-13 | 5.47E-12 | down |
| Cftr | -3.204146939 | 7.62E-13 | 5.75E-12 | down |
| Rph3al | -1.372810423 | 7.71E-13 | 5.81E-12 | down |
| Zdhhc14 | -1.07907245 | 8.01E-13 | 6.04E-12 | down |
| Rimkla | -2.935923141 | 8.02E-13 | 6.04E-12 | down |
| Slc7a10 | -2.029796865 | 8.39E-13 | 6.31E-12 | down |
| Arhgef33 | -1.355540057 | 8.73E-13 | 6.56E-12 | down |
| Gm7694 | -1.064299506 | 9.01E-13 | 6.76E-12 | down |
| Plekhn1 | -1.388210011 | 9.10E-13 | 6.82E-12 | down |
| Grik2 | -2.542078478 | 9.30E-13 | 6.96E-12 | down |
| Cxcl1 | -2.785074367 | 9.60E-13 | 7.17E-12 | down |
| Creb3l4 | -1.388522053 | 1.03E-12 | 7.65E-12 | down |
| Syp | -1.522978258 | 1.12E-12 | 8.29E-12 | down |
| Bbox1 | -3.607946373 | 1.25E-12 | 9.20E-12 | down |
| 2900005J15Rik | -1.793784202 | 1.38E-12 | 1.01E-11 | down |
| Sertad4 | -1.426921506 | 1.76E-12 | 1.28E-11 | down |
| Spsb1 | -1.313793604 | 1.77E-12 | 1.29E-11 | down |
| Cyp2d22 | -1.14340872 | 2.00E-12 | 1.46E-11 | down |
| Ppfia4 | -1.276954456 | 2.05E-12 | 1.49E-11 | down |
| Kiss1r | -1.16243607 | 2.09E-12 | 1.52E-11 | down |
| H2-DMa | -1.431179039 | 2.32E-12 | 1.68E-11 | down |
| Fblim1 | -1.157677051 | 2.46E-12 | 1.78E-11 | down |
| Plin1 | -2.576326811 | 2.47E-12 | 1.78E-11 | down |
| Ly86 | -1.555793427 | 2.47E-12 | 1.78E-11 | down |
| Il10ra | -1.287423026 | 2.50E-12 | 1.80E-11 | down |
| Mboat1 | -2.097942717 | 2.51E-12 | 1.80E-11 | down |
| Ccdc88b | -1.501129885 | 2.56E-12 | 1.84E-11 | down |
| Gm17821 | -1.691768867 | 2.60E-12 | 1.87E-11 | down |
| Arrb2 | -1.032800466 | 2.63E-12 | 1.88E-11 | down |
| 6330416G13Rik | -1.099525474 | 2.73E-12 | 1.95E-11 | down |
| Snap25 | -4.854389423 | 2.82E-12 | 2.02E-11 | down |
| 2810410L24Rik | -1.291801312 | 2.95E-12 | 2.10E-11 | down |
| Ltk | -1.302880156 | 3.01E-12 | 2.14E-11 | down |
| Acp5 | -1.723949685 | 3.08E-12 | 2.19E-11 | down |
| Epsti1 | -1.797436183 | 3.28E-12 | 2.32E-11 | down |
| Sox9 | -1.052368246 | 3.28E-12 | 2.32E-11 | down |
| Tmem202 | -1.988936528 | 3.56E-12 | 2.51E-11 | down |
| Fcer1g | -1.226341796 | 3.69E-12 | 2.60E-11 | down |
| Ccdc141 | -1.073886437 | 4.51E-12 | 3.16E-11 | down |
| Ptpro | -1.414404722 | 4.57E-12 | 3.19E-11 | down |
| Slc35d2 | -1.246548571 | 4.62E-12 | 3.23E-11 | down |
| Foxq1 | -1.368034703 | 4.69E-12 | 3.27E-11 | down |
| Nat1 | -2.348745282 | 4.71E-12 | 3.29E-11 | down |
| 2610035D17Rik | -1.252345574 | 5.16E-12 | 3.59E-11 | down |
| Aif1l | -1.276221789 | 5.18E-12 | 3.60E-11 | down |
| Slc38a3 | -1.584739089 | 5.72E-12 | 3.96E-11 | down |
| Slc16a6 | -1.217047226 | 5.87E-12 | 4.06E-11 | down |
| Tbata | -3.919093172 | 6.05E-12 | 4.18E-11 | down |
| Usp2 | -1.546087735 | 6.10E-12 | 4.21E-11 | down |
| Cdk8 | -1.003853189 | 6.26E-12 | 4.31E-11 | down |
| I830012O16Rik | -1.605719633 | 6.42E-12 | 4.42E-11 | down |
| AA414768 | -1.662177864 | 6.66E-12 | 4.57E-11 | down |
| Apoe | -1.28730891 | 7.57E-12 | 5.18E-11 | down |
| Igsf6 | -1.433656869 | 7.67E-12 | 5.24E-11 | down |
| Ms4a4d | -1.90256619 | 8.21E-12 | 5.59E-11 | down |
| Fam178b | -1.37448858 | 8.57E-12 | 5.82E-11 | down |
| Ltf | -5.311391707 | 8.71E-12 | 5.90E-11 | down |
| Rln1 | -2.333599232 | 9.01E-12 | 6.11E-11 | down |
| Mcoln2 | -2.357324736 | 9.07E-12 | 6.14E-11 | down |
| Chgb | -4.953568587 | 9.16E-12 | 6.20E-11 | down |
| Grem1 | -2.153111742 | 9.42E-12 | 6.37E-11 | down |
| Nat14 | -1.370220047 | 9.59E-12 | 6.49E-11 | down |
| Prrt2 | -1.683601054 | 9.84E-12 | 6.64E-11 | down |
| Fam47e | -2.470800067 | 1.01E-11 | 6.80E-11 | down |
| Klk1 | -4.342331761 | 1.05E-11 | 7.09E-11 | down |
| Inpp5j | -1.177011827 | 1.10E-11 | 7.40E-11 | down |
| Rgs10 | -1.223625346 | 1.11E-11 | 7.46E-11 | down |
| P2ry13 | -1.568555731 | 1.15E-11 | 7.70E-11 | down |
| Hsf4 | -1.342595104 | 1.18E-11 | 7.89E-11 | down |
| Gm8909 | -2.168677292 | 1.20E-11 | 8.05E-11 | down |
| Fmn1 | -1.005618337 | 1.24E-11 | 8.32E-11 | down |
| Dnm3 | -1.180150574 | 1.31E-11 | 8.76E-11 | down |
| Csf1 | -1.277092792 | 1.37E-11 | 9.14E-11 | down |
| AI848285 | -2.631231627 | 1.38E-11 | 9.17E-11 | down |
| Mapk13 | -1.127278982 | 1.39E-11 | 9.24E-11 | down |
| H2-K2 | -1.629267221 | 1.41E-11 | 9.41E-11 | down |
| Aif1 | -1.486923673 | 1.45E-11 | 9.65E-11 | down |
| Bcl3 | -1.500352362 | 1.49E-11 | 9.90E-11 | down |
| H2-D1 | -1.156043912 | 1.62E-11 | 1.07E-10 | down |
| Gsta3 | -1.994999047 | 1.66E-11 | 1.10E-10 | down |
| Tmem191c | -1.574679336 | 1.66E-11 | 1.10E-10 | down |
| Nupr1 | -1.310907995 | 1.82E-11 | 1.20E-10 | down |
| Klf2 | -1.225562657 | 1.93E-11 | 1.27E-10 | down |
| Zfp940 | -1.602103516 | 2.05E-11 | 1.34E-10 | down |
| Ifit3 | -1.56283257 | 2.08E-11 | 1.36E-10 | down |
| Krt15 | -5.064124017 | 2.09E-11 | 1.37E-10 | down |
| AI413582 | -1.166073781 | 2.32E-11 | 1.51E-10 | down |
| Kcnh2 | -2.802172832 | 2.48E-11 | 1.61E-10 | down |
| Colq | -5.071169305 | 2.55E-11 | 1.65E-10 | down |
| P4htm | -1.435311718 | 2.99E-11 | 1.92E-10 | down |
| 1700037C18Rik | -1.279259732 | 3.14E-11 | 2.02E-10 | down |
| E130311K13Rik | -1.102431348 | 3.37E-11 | 2.16E-10 | down |
| Ly6g6d | -1.479663777 | 3.53E-11 | 2.26E-10 | down |
| Dock11 | -1.474025671 | 3.65E-11 | 2.33E-10 | down |
| Ctf1 | -1.023687222 | 3.72E-11 | 2.38E-10 | down |
| Il7r | -2.105795639 | 3.96E-11 | 2.52E-10 | down |
| Tnfrsf11a | -1.635730967 | 4.17E-11 | 2.65E-10 | down |
| Sec14l2 | -1.231416751 | 4.54E-11 | 2.87E-10 | down |
| Rnf183 | -1.031731022 | 4.65E-11 | 2.94E-10 | down |
| Nckap1l | -1.108779495 | 4.76E-11 | 3.00E-10 | down |
| Plekhf1 | -1.116858294 | 4.86E-11 | 3.06E-10 | down |
| 4930414L22Rik | -1.208907376 | 4.88E-11 | 3.07E-10 | down |
| Map6d1 | -1.447549713 | 4.93E-11 | 3.10E-10 | down |
| Slc2a4rg-ps | -1.125428233 | 4.95E-11 | 3.11E-10 | down |
| Nek5 | -2.108619695 | 5.13E-11 | 3.22E-10 | down |
| Ccl22 | -4.038351147 | 5.56E-11 | 3.48E-10 | down |
| Klf6 | -1.022953441 | 5.76E-11 | 3.60E-10 | down |
| Cd14 | -1.254792588 | 5.90E-11 | 3.68E-10 | down |
| Prelp | -1.257171803 | 6.23E-11 | 3.88E-10 | down |
| Lrrc23 | -2.818518412 | 6.53E-11 | 4.06E-10 | down |
| Car3 | -3.190800068 | 6.56E-11 | 4.08E-10 | down |
| Lbp | -1.460306098 | 6.73E-11 | 4.17E-10 | down |
| Clec12a | -1.813069788 | 6.74E-11 | 4.18E-10 | down |
| Fmnl1 | -1.176941783 | 6.78E-11 | 4.19E-10 | down |
| Lmod2 | -1.571454793 | 6.90E-11 | 4.27E-10 | down |
| Coro1a | -1.284822221 | 7.17E-11 | 4.42E-10 | down |
| Cybb | -1.054396696 | 7.89E-11 | 4.85E-10 | down |
| Sfxn4 | -1.48956793 | 9.56E-11 | 5.85E-10 | down |
| Fcgr1 | -1.225673179 | 9.62E-11 | 5.88E-10 | down |
| Nfkbid | -1.613223774 | 9.78E-11 | 5.97E-10 | down |
| Actr3b | -1.0780936 | 1.03E-10 | 6.29E-10 | down |
| Scg2 | -4.209555844 | 1.08E-10 | 6.57E-10 | down |
| Met | -1.223545142 | 1.12E-10 | 6.79E-10 | down |
| Card9 | -1.733428288 | 1.12E-10 | 6.81E-10 | down |
| Amz1 | -1.427320897 | 1.24E-10 | 7.48E-10 | down |
| Fam105a | -1.168611151 | 1.24E-10 | 7.51E-10 | down |
| Exph5 | -1.183168242 | 1.25E-10 | 7.58E-10 | down |
| Wnt7b | -1.779771944 | 1.28E-10 | 7.74E-10 | down |
| 5830428M24Rik | -1.452100558 | 1.29E-10 | 7.79E-10 | down |
| Zfp36 | -1.214884632 | 1.37E-10 | 8.27E-10 | down |
| Hhex | -1.063573602 | 1.40E-10 | 8.42E-10 | down |
| Obsl1 | -1.446368677 | 1.47E-10 | 8.83E-10 | down |
| Ramp1 | -2.288206767 | 1.58E-10 | 9.43E-10 | down |
| Slc16a11 | -1.075416054 | 1.59E-10 | 9.49E-10 | down |
| Scn2b | -1.373498205 | 1.73E-10 | 1.03E-09 | down |
| Tmem52 | -1.4108555 | 1.76E-10 | 1.05E-09 | down |
| Ptprn | -2.579344646 | 1.94E-10 | 1.14E-09 | down |
| Opn3 | -1.102436799 | 1.98E-10 | 1.17E-09 | down |
| Lrp11 | -1.071150041 | 2.03E-10 | 1.19E-09 | down |
| Plekha6 | -1.283893468 | 2.04E-10 | 1.20E-09 | down |
| Mpeg1 | -1.076828265 | 2.14E-10 | 1.26E-09 | down |
| Chmp4c | -1.295003072 | 2.32E-10 | 1.36E-09 | down |
| Mterfd3 | -1.366240918 | 2.43E-10 | 1.42E-09 | down |
| Tmem54 | -2.54946767 | 2.59E-10 | 1.51E-09 | down |
| Igf2 | -2.585269675 | 2.62E-10 | 1.53E-09 | down |
| Vamp1 | -1.00790816 | 2.65E-10 | 1.54E-09 | down |
| Fosb | -2.826188254 | 2.65E-10 | 1.54E-09 | down |
| F2rl1 | -1.167004977 | 2.67E-10 | 1.55E-09 | down |
| Cpne2 | -1.270848035 | 2.94E-10 | 1.70E-09 | down |
| Cbs | -2.301234487 | 3.01E-10 | 1.74E-09 | down |
| Tmie | -1.478759486 | 3.09E-10 | 1.79E-09 | down |
| Kcnk3 | -2.119813251 | 3.24E-10 | 1.87E-09 | down |
| Npff | -1.919861114 | 3.37E-10 | 1.94E-09 | down |
| Pcbp3 | -1.226687195 | 3.42E-10 | 1.97E-09 | down |
| Fgfbp3 | -1.331486337 | 3.47E-10 | 1.99E-09 | down |
| Gmfg | -1.254810405 | 3.54E-10 | 2.03E-09 | down |
| Abca8a | -1.265479758 | 3.57E-10 | 2.05E-09 | down |
| Adam8 | -1.816358855 | 3.83E-10 | 2.19E-09 | down |
| Gpr65 | -1.396475656 | 4.15E-10 | 2.36E-09 | down |
| Ccdc13 | -2.432542495 | 4.33E-10 | 2.46E-09 | down |
| Kng2 | -1.442225339 | 4.82E-10 | 2.73E-09 | down |
| Lrrc49 | -1.029287675 | 4.91E-10 | 2.78E-09 | down |
| Ubd | -3.697128117 | 4.93E-10 | 2.79E-09 | down |
| Gal3st4 | -1.284076661 | 5.10E-10 | 2.88E-09 | down |
| Vill | -2.191094793 | 5.36E-10 | 3.02E-09 | down |
| Mycn | -1.684396727 | 5.39E-10 | 3.04E-09 | down |
| H2-Ea-ps | -1.770691209 | 5.43E-10 | 3.06E-09 | down |
| H2-T3 | -4.459935406 | 5.83E-10 | 3.27E-09 | down |
| Flrt3 | -1.019122337 | 5.87E-10 | 3.29E-09 | down |
| Sfpi1 | -1.214409644 | 6.17E-10 | 3.44E-09 | down |
| Pard3b | -1.149842598 | 6.22E-10 | 3.47E-09 | down |
| Kcnn4 | -1.6830769 | 6.26E-10 | 3.50E-09 | down |
| 5330417C22Rik | -2.155692658 | 6.40E-10 | 3.57E-09 | down |
| Ascl1 | -4.494657884 | 6.42E-10 | 3.58E-09 | down |
| AI317395 | -2.8406921 | 7.00E-10 | 3.89E-09 | down |
| Sst | -4.917990668 | 7.22E-10 | 4.00E-09 | down |
| Fam129a | -1.298139365 | 7.40E-10 | 4.10E-09 | down |
| Bmp4 | -1.448820504 | 7.61E-10 | 4.21E-09 | down |
| Brsk1 | -1.134115183 | 7.73E-10 | 4.27E-09 | down |
| Tnfaip8l2 | -1.33007997 | 7.75E-10 | 4.28E-09 | down |
| Hepacam2 | -2.862967955 | 8.13E-10 | 4.47E-09 | down |
| Noxo1 | -1.039597362 | 8.28E-10 | 4.55E-09 | down |
| Itgb8 | -2.054072016 | 8.49E-10 | 4.66E-09 | down |
| Alg6 | -1.054465679 | 8.55E-10 | 4.69E-09 | down |
| Syne4 | -1.429363906 | 8.56E-10 | 4.70E-09 | down |
| Trex1 | -1.012672403 | 8.78E-10 | 4.81E-09 | down |
| Ncmap | -1.23067748 | 8.98E-10 | 4.91E-09 | down |
| Amy1 | -2.089686212 | 9.33E-10 | 5.09E-09 | down |
| Hmga2-ps1 | -1.400976155 | 9.33E-10 | 5.09E-09 | down |
| Chadl | -1.853717719 | 9.71E-10 | 5.28E-09 | down |
| Apobec1 | -1.04502809 | 9.93E-10 | 5.40E-09 | down |
| Lilra5 | -1.391883569 | 1.08E-09 | 5.85E-09 | down |
| Kcnj13 | -2.15946969 | 1.11E-09 | 6.03E-09 | down |
| Gpr114 | -1.66182342 | 1.12E-09 | 6.05E-09 | down |
| Fermt3 | -1.161664821 | 1.15E-09 | 6.24E-09 | down |
| Tsc22d3 | -1.02285283 | 1.16E-09 | 6.26E-09 | down |
| Serpinb8 | -1.35530528 | 1.28E-09 | 6.90E-09 | down |
| Cidec | -2.857571753 | 1.29E-09 | 6.95E-09 | down |
| Olfm1 | -1.045247982 | 1.32E-09 | 7.07E-09 | down |
| Gm684 | -1.942319591 | 1.32E-09 | 7.07E-09 | down |
| Cox7a1 | -1.738981971 | 1.35E-09 | 7.22E-09 | down |
| E530011L22Rik | -1.593207908 | 1.36E-09 | 7.28E-09 | down |
| 1700001C02Rik | -2.282724639 | 1.48E-09 | 7.86E-09 | down |
| Srcrb4d | -1.214194021 | 1.50E-09 | 7.97E-09 | down |
| Slc36a2 | -2.904621588 | 1.58E-09 | 8.38E-09 | down |
| Dner | -4.163265345 | 1.68E-09 | 8.90E-09 | down |
| Itgax | -1.648235184 | 1.71E-09 | 9.01E-09 | down |
| Dnajc22 | -1.674822797 | 1.72E-09 | 9.08E-09 | down |
| Pcsk1 | -4.443926154 | 1.81E-09 | 9.54E-09 | down |
| Cd52 | -1.66023395 | 1.84E-09 | 9.69E-09 | down |
| Il18bp | -1.344463356 | 1.91E-09 | 1.00E-08 | down |
| Car15 | -1.7190444 | 1.97E-09 | 1.03E-08 | down |
| Ccr5 | -1.445734808 | 2.06E-09 | 1.08E-08 | down |
| Rnf32 | -1.209237332 | 2.10E-09 | 1.10E-08 | down |
| BC020402 | -1.100760328 | 2.10E-09 | 1.10E-08 | down |
| Pla2g2d | -2.212087594 | 2.16E-09 | 1.13E-08 | down |
| C3ar1 | -1.014224052 | 2.18E-09 | 1.14E-08 | down |
| AI467606 | -1.307081187 | 2.21E-09 | 1.15E-08 | down |
| Clstn3 | -3.497122126 | 2.30E-09 | 1.20E-08 | down |
| Sec14l4 | -2.215747983 | 2.45E-09 | 1.27E-08 | down |
| H2-Ob | -1.869449332 | 2.56E-09 | 1.33E-08 | down |
| Trpm2 | -1.702964541 | 2.68E-09 | 1.38E-08 | down |
| Tceal3 | -2.419748639 | 2.72E-09 | 1.40E-08 | down |
| Cd59b | -1.202765373 | 2.87E-09 | 1.48E-08 | down |
| Pir | -1.004977565 | 2.92E-09 | 1.50E-08 | down |
| Atp6v1g2 | -1.223553444 | 3.18E-09 | 1.63E-08 | down |
| Creg2 | -2.101977168 | 3.18E-09 | 1.63E-08 | down |
| Car9 | -2.053273931 | 3.22E-09 | 1.64E-08 | down |
| Larp6 | -1.219489216 | 3.27E-09 | 1.67E-08 | down |
| Bmp7 | -1.141876979 | 3.41E-09 | 1.74E-08 | down |
| Traf5 | -1.165559065 | 3.49E-09 | 1.77E-08 | down |
| Dok7 | -3.393704635 | 3.50E-09 | 1.78E-08 | down |
| Slco1a5 | -2.649827517 | 3.62E-09 | 1.84E-08 | down |
| Rab36 | -1.278118829 | 3.80E-09 | 1.92E-08 | down |
| Emr1 | -1.013117105 | 3.96E-09 | 2.00E-08 | down |
| Pcsk2 | -5.36968487 | 3.98E-09 | 2.01E-08 | down |
| Gm5086 | -2.190199402 | 4.04E-09 | 2.04E-08 | down |
| Slc9a2 | -2.450991824 | 4.06E-09 | 2.05E-08 | down |
| Lilra6 | -2.1190106 | 4.17E-09 | 2.10E-08 | down |
| Adm | -1.183852514 | 4.33E-09 | 2.18E-08 | down |
| Kcnn2 | -1.197212461 | 4.41E-09 | 2.21E-08 | down |
| F2 | -2.332059917 | 4.41E-09 | 2.21E-08 | down |
| Slc6a14 | -1.470489323 | 4.42E-09 | 2.21E-08 | down |
| Chd5 | -2.035822 | 4.78E-09 | 2.39E-08 | down |
| Cd48 | -1.448635183 | 4.95E-09 | 2.46E-08 | down |
| Tppp3 | -1.056817297 | 5.04E-09 | 2.51E-08 | down |
| Calca | -6.000327335 | 5.22E-09 | 2.59E-08 | down |
| 1110046J04Rik | -1.444354828 | 5.25E-09 | 2.61E-08 | down |
| 2310040G24Rik | -1.263460768 | 5.27E-09 | 2.62E-08 | down |
| Rbp4 | -2.768849873 | 5.38E-09 | 2.67E-08 | down |
| Tnfrsf12a | -1.035172834 | 5.68E-09 | 2.81E-08 | down |
| Vwf | -1.343402445 | 5.72E-09 | 2.83E-08 | down |
| Fcgr4 | -2.056821425 | 5.96E-09 | 2.94E-08 | down |
| Apbb1ip | -1.09544885 | 6.21E-09 | 3.06E-08 | down |
| Gm12185 | -1.791907635 | 6.62E-09 | 3.25E-08 | down |
| Ckmt1 | -1.175581697 | 7.00E-09 | 3.43E-08 | down |
| Pglyrp1 | -2.861383752 | 7.09E-09 | 3.46E-08 | down |
| Gas1 | -1.111208968 | 7.55E-09 | 3.68E-08 | down |
| Selenbp1 | -1.002852283 | 8.42E-09 | 4.07E-08 | down |
| Cd37 | -1.261202759 | 8.63E-09 | 4.16E-08 | down |
| Lat2 | -1.040017571 | 9.37E-09 | 4.50E-08 | down |
| Igtp | -1.018892199 | 1.01E-08 | 4.84E-08 | down |
| Gpr19 | -1.318396954 | 1.02E-08 | 4.86E-08 | down |
| Tspan8 | -1.245104834 | 1.02E-08 | 4.86E-08 | down |
| Gm1673 | -2.59255883 | 1.02E-08 | 4.86E-08 | down |
| Hrasls | -1.81029686 | 1.03E-08 | 4.92E-08 | down |
| Pdk4 | -1.268515987 | 1.05E-08 | 4.98E-08 | down |
| Cyp2e1 | -2.87811772 | 1.06E-08 | 5.06E-08 | down |
| Zmynd15 | -1.271284377 | 1.07E-08 | 5.10E-08 | down |
| Kcp | -1.547805758 | 1.11E-08 | 5.26E-08 | down |
| Ltbp1 | -1.140209173 | 1.26E-08 | 5.95E-08 | down |
| Efna2 | -1.600199077 | 1.28E-08 | 6.02E-08 | down |
| Tmem45b | -3.111674272 | 1.36E-08 | 6.38E-08 | down |
| Mapk15 | -2.151414965 | 1.36E-08 | 6.39E-08 | down |
| Lyl1 | -1.169076104 | 1.37E-08 | 6.41E-08 | down |
| Rab6b | -1.587423436 | 1.46E-08 | 6.83E-08 | down |
| Crybb1 | -2.425127796 | 1.48E-08 | 6.90E-08 | down |
| Zfp947 | -1.033795945 | 1.53E-08 | 7.14E-08 | down |
| Dgkb | -3.247644833 | 1.53E-08 | 7.14E-08 | down |
| Stmn3 | -2.892414796 | 1.54E-08 | 7.19E-08 | down |
| Cyp27a1 | -1.412652665 | 1.56E-08 | 7.28E-08 | down |
| Krt7 | -1.338568432 | 1.61E-08 | 7.50E-08 | down |
| 4732471J01Rik | -1.759860004 | 1.61E-08 | 7.50E-08 | down |
| Slc22a3 | -2.939150428 | 1.63E-08 | 7.56E-08 | down |
| Siglece | -1.476776863 | 1.64E-08 | 7.62E-08 | down |
| Cpa2 | -1.930775138 | 1.70E-08 | 7.91E-08 | down |
| Lyz1 | -1.184199206 | 1.82E-08 | 8.42E-08 | down |
| Lst1 | -1.554597733 | 1.88E-08 | 8.72E-08 | down |
| Metrn | -1.411929686 | 1.97E-08 | 9.12E-08 | down |
| Rnf207 | -2.296049466 | 2.00E-08 | 9.23E-08 | down |
| Lck | -1.749159895 | 2.00E-08 | 9.24E-08 | down |
| Gm13251 | -1.072355139 | 2.12E-08 | 9.78E-08 | down |
| Zmynd10 | -3.269772945 | 2.13E-08 | 9.79E-08 | down |
| Opn4 | -1.919648898 | 2.32E-08 | 1.06E-07 | down |
| Loxl4 | -1.322956466 | 2.34E-08 | 1.07E-07 | down |
| H2-Bl | -2.190832958 | 2.46E-08 | 1.12E-07 | down |
| Dio1 | -1.308721305 | 2.51E-08 | 1.15E-07 | down |
| Neu2 | -2.000306602 | 2.59E-08 | 1.18E-07 | down |
| Fam183b | -2.906536836 | 2.66E-08 | 1.21E-07 | down |
| Psmb9 | -1.329033986 | 2.78E-08 | 1.26E-07 | down |
| Fmo1 | -1.308223843 | 2.79E-08 | 1.27E-07 | down |
| Eppk1 | -1.373895226 | 2.80E-08 | 1.27E-07 | down |
| Pglyrp2 | -3.209886247 | 2.88E-08 | 1.30E-07 | down |
| Gm20257 | -1.433579943 | 2.90E-08 | 1.31E-07 | down |
| Fam151b | -1.030425454 | 2.95E-08 | 1.34E-07 | down |
| Tmem51as1 | -1.258159885 | 3.05E-08 | 1.37E-07 | down |
| Slitrk6 | -3.348168041 | 3.06E-08 | 1.38E-07 | down |
| Wfikkn2 | -1.445261387 | 3.28E-08 | 1.47E-07 | down |
| Neurl3 | -1.056981719 | 3.35E-08 | 1.50E-07 | down |
| Trpm3 | -1.595871813 | 3.46E-08 | 1.55E-07 | down |
| Fcho1 | -1.411400104 | 3.59E-08 | 1.61E-07 | down |
| Tmprss13 | -2.302737903 | 3.86E-08 | 1.73E-07 | down |
| Hspb1 | -1.007655021 | 4.03E-08 | 1.80E-07 | down |
| Abcd2 | -1.641147812 | 4.16E-08 | 1.85E-07 | down |
| Cfd | -2.422385779 | 4.19E-08 | 1.87E-07 | down |
| Pcyt1b | -1.87966832 | 4.26E-08 | 1.90E-07 | down |
| Xpnpep2 | -2.058799863 | 4.60E-08 | 2.04E-07 | down |
| Myocd | -2.708844369 | 4.73E-08 | 2.09E-07 | down |
| Gamt | -1.24023302 | 4.80E-08 | 2.12E-07 | down |
| Gstm7 | -1.001018997 | 4.95E-08 | 2.19E-07 | down |
| Trf | -1.092769228 | 5.01E-08 | 2.21E-07 | down |
| Insrr | -2.631912464 | 5.02E-08 | 2.22E-07 | down |
| Fxyd3 | -2.405297967 | 5.04E-08 | 2.22E-07 | down |
| Kctd8 | -1.21444697 | 5.21E-08 | 2.29E-07 | down |
| 1700048O20Rik | -1.646707069 | 5.69E-08 | 2.49E-07 | down |
| Serpinb1b | -2.047059449 | 5.90E-08 | 2.57E-07 | down |
| Fndc7 | -1.747743045 | 5.91E-08 | 2.58E-07 | down |
| Lax1 | -2.538013875 | 6.00E-08 | 2.62E-07 | down |
| Fads6 | -1.402984327 | 6.08E-08 | 2.64E-07 | down |
| Rcsd1 | -1.21680306 | 6.26E-08 | 2.72E-07 | down |
| Syt7 | -1.373147789 | 6.30E-08 | 2.74E-07 | down |
| Cd72 | -1.43119673 | 6.39E-08 | 2.77E-07 | down |
| Zfp934 | -1.312940794 | 6.43E-08 | 2.79E-07 | down |
| Alas2 | -2.136355707 | 6.48E-08 | 2.81E-07 | down |
| Sgcd | -2.336820228 | 6.78E-08 | 2.93E-07 | down |
| H2-Eb1 | -1.440360738 | 7.05E-08 | 3.04E-07 | down |
| Ddah1 | -1.166144236 | 7.09E-08 | 3.05E-07 | down |
| Aldh1b1 | -2.571616406 | 7.48E-08 | 3.21E-07 | down |
| Psmb8 | -1.042006399 | 7.99E-08 | 3.42E-07 | down |
| Fam229b | -1.395874297 | 8.63E-08 | 3.68E-07 | down |
| Slc7a8 | -1.088677631 | 8.68E-08 | 3.70E-07 | down |
| BC052688 | -1.78397926 | 8.86E-08 | 3.77E-07 | down |
| Pfkfb1 | -1.657435697 | 9.04E-08 | 3.85E-07 | down |
| Tnfsf13b | -1.363419875 | 9.10E-08 | 3.87E-07 | down |
| Gm20605 | -1.205018202 | 9.24E-08 | 3.92E-07 | down |
| Lilrb3 | -1.100311811 | 9.48E-08 | 4.02E-07 | down |
| Niacr1 | -2.604492362 | 9.50E-08 | 4.03E-07 | down |
| Cldn23 | -2.135240665 | 1.01E-07 | 4.29E-07 | down |
| Igdcc4 | -2.148923003 | 1.02E-07 | 4.30E-07 | down |
| Synpo2 | -1.347274487 | 1.08E-07 | 4.55E-07 | down |
| Adipoq | -2.710413441 | 1.09E-07 | 4.59E-07 | down |
| Aqp6 | -1.281436855 | 1.12E-07 | 4.69E-07 | down |
| Gstm6 | -1.537387165 | 1.27E-07 | 5.31E-07 | down |
| Ccr2 | -1.089156236 | 1.31E-07 | 5.47E-07 | down |
| Lfng | -1.007606435 | 1.34E-07 | 5.57E-07 | down |
| AI182371 | -1.91618379 | 1.38E-07 | 5.75E-07 | down |
| Tppp | -1.065270911 | 1.45E-07 | 6.00E-07 | down |
| Runx3 | -1.304009987 | 1.52E-07 | 6.30E-07 | down |
| Faxc | -1.185338643 | 1.56E-07 | 6.45E-07 | down |
| Peli3 | -1.237783193 | 1.63E-07 | 6.72E-07 | down |
| P2ry12 | -1.21944574 | 1.64E-07 | 6.76E-07 | down |
| Clec7a | -1.463995269 | 1.67E-07 | 6.88E-07 | down |
| Milr1 | -1.311087482 | 1.96E-07 | 8.00E-07 | down |
| Ccnb1ip1 | -1.898989972 | 1.96E-07 | 8.01E-07 | down |
| Apod | -1.580584379 | 1.96E-07 | 8.02E-07 | down |
| Zfp879 | -1.639279292 | 2.02E-07 | 8.23E-07 | down |
| Csf3r | -1.183986917 | 2.05E-07 | 8.33E-07 | down |
| Evi2b | -1.270704365 | 2.19E-07 | 8.90E-07 | down |
| Mutyh | -1.085197379 | 2.21E-07 | 8.97E-07 | down |
| Bex2 | -1.416232448 | 2.32E-07 | 9.42E-07 | down |
| Cd209b | -2.40956862 | 2.34E-07 | 9.47E-07 | down |
| Clec5a | -1.309313241 | 2.49E-07 | 1.01E-06 | down |
| Ndn | -1.18965052 | 2.57E-07 | 1.03E-06 | down |
| Ltb4r1 | -1.724228879 | 2.58E-07 | 1.04E-06 | down |
| Srpk3 | -2.268332406 | 2.81E-07 | 1.13E-06 | down |
| Hopx | -1.087472398 | 2.91E-07 | 1.17E-06 | down |
| Mest | -1.11839181 | 2.92E-07 | 1.17E-06 | down |
| Fbxl16 | -1.264749629 | 3.03E-07 | 1.21E-06 | down |
| Nkd1 | -1.028883381 | 3.07E-07 | 1.22E-06 | down |
| Stmnd1 | -3.03191971 | 3.28E-07 | 1.30E-06 | down |
| Tmc3 | -1.849590076 | 3.32E-07 | 1.32E-06 | down |
| Lix1 | -1.236740987 | 3.39E-07 | 1.34E-06 | down |
| Cd33 | -1.113165286 | 3.49E-07 | 1.38E-06 | down |
| Vav1 | -1.087222701 | 3.55E-07 | 1.40E-06 | down |
| Sh2d1b1 | -1.872196524 | 3.65E-07 | 1.44E-06 | down |
| Lpxn | -1.247567852 | 3.82E-07 | 1.50E-06 | down |
| Tnfrsf25 | -1.522030165 | 3.95E-07 | 1.55E-06 | down |
| Gbp11 | -2.555446602 | 4.01E-07 | 1.57E-06 | down |
| Mybpc2 | -2.528423591 | 4.33E-07 | 1.69E-06 | down |
| Cox8b | -3.471745083 | 4.37E-07 | 1.70E-06 | down |
| Cnn1 | -1.853746665 | 4.40E-07 | 1.71E-06 | down |
| Tmod1 | -1.506726758 | 4.44E-07 | 1.73E-06 | down |
| Klrb1b | -2.035780723 | 4.45E-07 | 1.73E-06 | down |
| F5 | -1.218759491 | 4.52E-07 | 1.76E-06 | down |
| Adrb3 | -3.029728342 | 4.60E-07 | 1.78E-06 | down |
| S100a6 | -1.133635769 | 4.61E-07 | 1.79E-06 | down |
| Clec3b | -1.166686031 | 4.79E-07 | 1.85E-06 | down |
| Cxcl2 | -3.288224379 | 4.88E-07 | 1.89E-06 | down |
| Nexn | -1.778907759 | 5.41E-07 | 2.08E-06 | down |
| Ceacam10 | -2.948821462 | 5.88E-07 | 2.25E-06 | down |
| Cfp | -1.068497716 | 5.89E-07 | 2.25E-06 | down |
| Cd180 | -1.437710579 | 6.07E-07 | 2.31E-06 | down |
| Gfap | -1.133381121 | 6.35E-07 | 2.42E-06 | down |
| Pln | -3.624611108 | 6.52E-07 | 2.48E-06 | down |
| Flt3l | -1.210037619 | 7.05E-07 | 2.67E-06 | down |
| Tceal5 | -1.495815508 | 7.46E-07 | 2.82E-06 | down |
| Rgs1 | -2.100089889 | 7.61E-07 | 2.88E-06 | down |
| Epb4.1l3 | -1.027391087 | 8.46E-07 | 3.18E-06 | down |
| Ier3 | -1.069867732 | 8.54E-07 | 3.21E-06 | down |
| Fut10 | -1.267048155 | 8.59E-07 | 3.23E-06 | down |
| Arl11 | -1.209476478 | 8.91E-07 | 3.34E-06 | down |
| 1810034E14Rik | -1.65165954 | 9.21E-07 | 3.44E-06 | down |
| Stra6 | -2.071665807 | 9.32E-07 | 3.48E-06 | down |
| 9830107B12Rik | -1.832198973 | 1.00E-06 | 3.72E-06 | down |
| Dusp1 | -1.521862787 | 1.02E-06 | 3.78E-06 | down |
| Vit | -1.317990062 | 1.02E-06 | 3.79E-06 | down |
| Krt4 | -3.383521057 | 1.02E-06 | 3.80E-06 | down |
| 6030419C18Rik | -1.175660945 | 1.09E-06 | 4.02E-06 | down |
| Neurl1a | -2.252410168 | 1.11E-06 | 4.08E-06 | down |
| Mrap | -2.388801988 | 1.15E-06 | 4.22E-06 | down |
| Sox15 | -1.328049525 | 1.16E-06 | 4.25E-06 | down |
| Rwdd2a | -1.395717948 | 1.22E-06 | 4.47E-06 | down |
| Sostdc1 | -1.406755984 | 1.25E-06 | 4.56E-06 | down |
| 0610009B14Rik | -1.086811534 | 1.28E-06 | 4.68E-06 | down |
| Irf4 | -1.510082756 | 1.34E-06 | 4.87E-06 | down |
| Dcstamp | -1.310796308 | 1.37E-06 | 4.98E-06 | down |
| Kcnmb4 | -1.641710644 | 1.42E-06 | 5.16E-06 | down |
| Cmbl | -1.374255145 | 1.45E-06 | 5.25E-06 | down |
| Klra2 | -2.055383892 | 1.50E-06 | 5.44E-06 | down |
| Tnn | -2.299435031 | 1.52E-06 | 5.49E-06 | down |
| Btla | -1.724334396 | 1.54E-06 | 5.55E-06 | down |
| Cd300lf | -1.367637515 | 1.61E-06 | 5.80E-06 | down |
| Degs2 | -1.244604377 | 1.65E-06 | 5.96E-06 | down |
| Olfm4 | -1.801650618 | 1.67E-06 | 6.00E-06 | down |
| Cytl1 | -2.650263296 | 1.73E-06 | 6.20E-06 | down |
| Dok2 | -1.281667475 | 1.74E-06 | 6.25E-06 | down |
| Retn | -2.803530171 | 1.78E-06 | 6.36E-06 | down |
| Cd209d | -2.139832163 | 1.82E-06 | 6.50E-06 | down |
| E130309D14Rik | -1.401300448 | 1.89E-06 | 6.74E-06 | down |
| Tbx6 | -1.604240336 | 1.91E-06 | 6.81E-06 | down |
| Edaradd | -1.135010705 | 2.02E-06 | 7.18E-06 | down |
| Duoxa1 | -1.239161828 | 2.18E-06 | 7.70E-06 | down |
| Map3k8 | -1.058596816 | 2.21E-06 | 7.80E-06 | down |
| Radil | -1.112758732 | 2.30E-06 | 8.10E-06 | down |
| Tmod2 | -1.058634102 | 2.35E-06 | 8.28E-06 | down |
| Msln | -3.272774748 | 2.43E-06 | 8.54E-06 | down |
| Cyp2j13 | -1.411749024 | 2.44E-06 | 8.56E-06 | down |
| Pf4 | -1.416170526 | 2.50E-06 | 8.77E-06 | down |
| Junb | -1.155718997 | 2.52E-06 | 8.82E-06 | down |
| F630042J09Rik | -1.395470881 | 2.58E-06 | 9.03E-06 | down |
| Cacnb1 | -1.087787261 | 2.62E-06 | 9.15E-06 | down |
| Neu3 | -1.041801478 | 2.62E-06 | 9.16E-06 | down |
| Clca4 | -2.350651216 | 2.69E-06 | 9.40E-06 | down |
| Slc17a9 | -1.337662551 | 2.80E-06 | 9.74E-06 | down |
| Ak1 | -1.270132627 | 2.97E-06 | 1.03E-05 | down |
| Syt2 | -1.314089223 | 2.98E-06 | 1.03E-05 | down |
| Lefty1 | -1.293472912 | 3.02E-06 | 1.05E-05 | down |
| Oit1 | -1.790445356 | 3.03E-06 | 1.05E-05 | down |
| Cd86 | -1.075872973 | 3.13E-06 | 1.08E-05 | down |
| Pnpla3 | -2.193562169 | 3.20E-06 | 1.10E-05 | down |
| Pknox2 | -1.231181088 | 3.24E-06 | 1.12E-05 | down |
| Prox2 | -1.272537833 | 3.38E-06 | 1.16E-05 | down |
| Bin2 | -1.096572134 | 3.62E-06 | 1.24E-05 | down |
| Muc5b | -3.824808761 | 3.71E-06 | 1.27E-05 | down |
| Serpinb12 | -3.582918479 | 3.72E-06 | 1.27E-05 | down |
| Fbxo17 | -1.045047295 | 3.90E-06 | 1.33E-05 | down |
| 4930451C15Rik | -2.310669563 | 4.00E-06 | 1.36E-05 | down |
| 9530053A07Rik | -1.958215185 | 4.13E-06 | 1.40E-05 | down |
| AI463170 | -1.338841658 | 4.15E-06 | 1.41E-05 | down |
| Ifitm1 | -1.295250161 | 4.17E-06 | 1.41E-05 | down |
| Sox2 | -3.435081949 | 4.21E-06 | 1.43E-05 | down |
| Gpx2 | -1.126919424 | 4.22E-06 | 1.43E-05 | down |
| Slc5a5 | -1.993829337 | 4.27E-06 | 1.44E-05 | down |
| Stmn2 | -1.12756894 | 4.37E-06 | 1.48E-05 | down |
| Siglech | -1.726960358 | 4.37E-06 | 1.48E-05 | down |
| Ntn3 | -1.603843408 | 4.50E-06 | 1.51E-05 | down |
| Kazald1 | -1.194714171 | 4.59E-06 | 1.54E-05 | down |
| Musk | -3.351062355 | 4.68E-06 | 1.57E-05 | down |
| Lmtk3 | -1.038841365 | 4.70E-06 | 1.58E-05 | down |
| Nat8l | -1.980653338 | 4.84E-06 | 1.62E-05 | down |
| Map7d2 | -1.113248095 | 4.90E-06 | 1.64E-05 | down |
| Gm5860 | -1.520488034 | 5.28E-06 | 1.76E-05 | down |
| Cacna2d2 | -1.779027488 | 5.70E-06 | 1.89E-05 | down |
| Orm1 | -2.482455284 | 5.98E-06 | 1.97E-05 | down |
| Myrip | -1.477142136 | 6.14E-06 | 2.02E-05 | down |
| Atf3 | -1.445742666 | 6.26E-06 | 2.06E-05 | down |
| Srpx | -1.136793685 | 6.31E-06 | 2.08E-05 | down |
| Msh5 | -1.356274939 | 6.48E-06 | 2.13E-05 | down |
| 1110017D15Rik | -1.926090352 | 6.61E-06 | 2.17E-05 | down |
| Rapsn | -1.736371888 | 7.09E-06 | 2.32E-05 | down |
| 4930502A04Rik | -1.043839725 | 7.24E-06 | 2.36E-05 | down |
| Ptchd1 | -1.365408285 | 7.26E-06 | 2.37E-05 | down |
| Kcna6 | -2.341891318 | 7.34E-06 | 2.39E-05 | down |
| Gys2 | -3.124851487 | 7.37E-06 | 2.40E-05 | down |
| Pdlim4 | -1.022743775 | 7.37E-06 | 2.40E-05 | down |
| Upk1b | -2.888528888 | 7.44E-06 | 2.42E-05 | down |
| Col20a1 | -1.044504678 | 7.57E-06 | 2.46E-05 | down |
| Stx19 | -1.830543915 | 7.58E-06 | 2.46E-05 | down |
| 4930404I05Rik | -1.293469193 | 7.64E-06 | 2.48E-05 | down |
| Beta-s | -2.050950587 | 7.83E-06 | 2.54E-05 | down |
| Flt3 | -2.253083853 | 7.84E-06 | 2.54E-05 | down |
| Rmst | -1.065366933 | 8.55E-06 | 2.76E-05 | down |
| Myrf | -1.200118597 | 8.89E-06 | 2.86E-05 | down |
| Krt42 | -1.220741066 | 8.93E-06 | 2.87E-05 | down |
| Cd209f | -1.496696017 | 9.19E-06 | 2.96E-05 | down |
| Gpr176 | -1.199243437 | 9.57E-06 | 3.07E-05 | down |
| Gpr81 | -1.297218606 | 1.02E-05 | 3.28E-05 | down |
| Gm4980 | -1.024003207 | 1.08E-05 | 3.46E-05 | down |
| Dbndd1 | -1.034597203 | 1.09E-05 | 3.47E-05 | down |
| Kcnq4 | -1.522469536 | 1.16E-05 | 3.68E-05 | down |
| 1700001L19Rik | -1.949727803 | 1.17E-05 | 3.70E-05 | down |
| Ccdc17 | -1.008213024 | 1.20E-05 | 3.80E-05 | down |
| G630090E17Rik | -1.45624412 | 1.21E-05 | 3.82E-05 | down |
| Maats1 | -1.76409948 | 1.23E-05 | 3.87E-05 | down |
| 2310014L17Rik | -1.399558878 | 1.35E-05 | 4.23E-05 | down |
| Naip5 | -1.322126452 | 1.40E-05 | 4.37E-05 | down |
| Ssu2 | -1.61054764 | 1.44E-05 | 4.51E-05 | down |
| Zfp454 | -1.006012234 | 1.46E-05 | 4.57E-05 | down |
| Cd209g | -1.739642929 | 1.50E-05 | 4.67E-05 | down |
| Slamf7 | -2.270076548 | 1.51E-05 | 4.71E-05 | down |
| C5ar2 | -1.23296787 | 1.52E-05 | 4.73E-05 | down |
| Dtna | -1.448021827 | 1.55E-05 | 4.82E-05 | down |
| Ccdc33 | -2.638148797 | 1.59E-05 | 4.94E-05 | down |
| Cyp2f2 | -3.085520347 | 1.59E-05 | 4.95E-05 | down |
| Gpr35 | -1.464479377 | 1.60E-05 | 4.96E-05 | down |
| Efcab12 | -1.107967411 | 1.60E-05 | 4.97E-05 | down |
| Kynu | -3.125738556 | 1.63E-05 | 5.03E-05 | down |
| Tubb3 | -2.567257159 | 1.67E-05 | 5.15E-05 | down |
| Serpina3i | -1.640438398 | 1.75E-05 | 5.39E-05 | down |
| Sync | -1.812555049 | 1.76E-05 | 5.42E-05 | down |
| Foxa1 | -1.070729497 | 1.82E-05 | 5.60E-05 | down |
| B930003M22Rik | -1.111209653 | 1.82E-05 | 5.60E-05 | down |
| Col11a2 | -1.07610178 | 1.94E-05 | 5.95E-05 | down |
| Ang | -1.32895022 | 1.98E-05 | 6.07E-05 | down |
| Scd3 | -1.207315812 | 2.00E-05 | 6.10E-05 | down |
| Gpr132 | -2.088569466 | 2.04E-05 | 6.23E-05 | down |
| 2210039B01Rik | -1.253866346 | 2.10E-05 | 6.41E-05 | down |
| 2310007L24Rik | -2.244545585 | 2.17E-05 | 6.61E-05 | down |
| Col6a5 | -1.476245045 | 2.21E-05 | 6.73E-05 | down |
| BC096441 | -1.282524315 | 2.27E-05 | 6.90E-05 | down |
| Shank1 | -1.228804722 | 2.59E-05 | 7.81E-05 | down |
| Orm2 | -2.535752834 | 2.62E-05 | 7.90E-05 | down |
| Acsm1 | -2.810269854 | 2.63E-05 | 7.90E-05 | down |
| Pnoc | -2.662853745 | 2.67E-05 | 8.03E-05 | down |
| Lrrc25 | -1.003384025 | 2.71E-05 | 8.14E-05 | down |
| Lep | -2.566798141 | 2.85E-05 | 8.53E-05 | down |
| Nkx2-2 | -2.587749109 | 3.00E-05 | 8.94E-05 | down |
| B130024G19Rik | -1.035182312 | 3.02E-05 | 8.98E-05 | down |
| Lrmp | -1.119739748 | 3.05E-05 | 9.06E-05 | down |
| Tlr9 | -1.305999743 | 3.07E-05 | 9.12E-05 | down |
| Anpep | -1.119172197 | 3.23E-05 | 9.58E-05 | down |
| Gm3716 | -1.432274797 | 3.26E-05 | 9.66E-05 | down |
| Il21r | -1.237133885 | 3.29E-05 | 9.75E-05 | down |
| Rspo1 | -1.732019959 | 3.33E-05 | 9.86E-05 | down |
| Aldh3a1 | -2.048548212 | 3.39E-05 | 0.0001002 | down |
| C1qtnf2 | -1.287684337 | 3.45E-05 | 0.0001018 | down |
| Gm16938 | -1.28282719 | 3.46E-05 | 0.0001021 | down |
| Cd209c | -1.084921849 | 3.56E-05 | 0.000105 | down |
| Tnmd | -3.104881881 | 3.64E-05 | 0.000107 | down |
| Ank1 | -2.153936552 | 3.70E-05 | 0.0001086 | down |
| Itgal | -1.138312636 | 3.70E-05 | 0.0001087 | down |
| Oas2 | -1.06666485 | 3.72E-05 | 0.0001091 | down |
| Scimp | -1.228266952 | 3.78E-05 | 0.0001109 | down |
| Dok5 | -1.702048977 | 3.80E-05 | 0.0001113 | down |
| E130102H24Rik | -1.209646171 | 3.81E-05 | 0.0001116 | down |
| 2410004P03Rik | -2.748535092 | 3.82E-05 | 0.0001118 | down |
| Pcolce2 | -1.070050148 | 3.94E-05 | 0.000115 | down |
| Fgr | -1.101121208 | 3.95E-05 | 0.0001153 | down |
| Pigr | -1.971891335 | 4.11E-05 | 0.0001198 | down |
| F630028O10Rik | -1.194056973 | 4.22E-05 | 0.0001229 | down |
| Ccl24 | -2.047733689 | 4.28E-05 | 0.0001244 | down |
| H2-Q2 | -1.240727774 | 4.49E-05 | 0.00013 | down |
| C7 | -2.422607468 | 4.58E-05 | 0.0001325 | down |
| Cited1 | -1.391697804 | 4.60E-05 | 0.000133 | down |
| Clec4a3 | -1.079432959 | 4.65E-05 | 0.0001344 | down |
| Ccl5 | -1.589963518 | 4.67E-05 | 0.0001349 | down |
| Gcnt3 | -1.435897396 | 4.73E-05 | 0.0001365 | down |
| Glipr1 | -1.147798474 | 4.78E-05 | 0.0001377 | down |
| Gm15408 | -1.536172137 | 4.79E-05 | 0.0001381 | down |
| 3110070M22Rik | -1.047658774 | 4.81E-05 | 0.0001387 | down |
| Bcl11a | -1.864950273 | 4.91E-05 | 0.0001412 | down |
| Cda | -3.203093117 | 4.94E-05 | 0.000142 | down |
| Rasal3 | -1.275279187 | 4.99E-05 | 0.0001435 | down |
| Gata3 | -3.2585913 | 5.02E-05 | 0.0001441 | down |
| Mlxipl | -1.724803099 | 5.08E-05 | 0.0001456 | down |
| Kif19a | -1.576890403 | 5.14E-05 | 0.0001474 | down |
| Cd300lb | -1.088793786 | 5.15E-05 | 0.0001475 | down |
| Rbp1 | -1.124569525 | 5.27E-05 | 0.0001508 | down |
| Snx20 | -1.074158089 | 5.57E-05 | 0.0001586 | down |
| Olfr78 | -1.605923687 | 5.60E-05 | 0.0001595 | down |
| Ybx2 | -1.248765962 | 5.66E-05 | 0.0001609 | down |
| Zcchc5 | -2.054630558 | 5.81E-05 | 0.000165 | down |
| C1qtnf9 | -1.213834317 | 5.87E-05 | 0.0001666 | down |
| Stat4 | -2.040090571 | 5.99E-05 | 0.0001697 | down |
| Gm4532 | -1.247198033 | 6.17E-05 | 0.0001745 | down |
| Syn1 | -1.434694829 | 6.26E-05 | 0.0001768 | down |
| Snca | -1.934999891 | 6.36E-05 | 0.0001792 | down |
| 5430427O19Rik | -1.456051297 | 6.45E-05 | 0.0001815 | down |
| Inmt | -1.593375838 | 6.53E-05 | 0.0001836 | down |
| Zfp296 | -1.046520648 | 6.57E-05 | 0.0001848 | down |
| Frmpd1 | -1.629665535 | 6.60E-05 | 0.0001856 | down |
| Vwa2 | -1.109249676 | 6.83E-05 | 0.0001912 | down |
| Clec4a2 | -1.165045396 | 6.84E-05 | 0.0001915 | down |
| 5830454E08Rik | -1.236741363 | 7.42E-05 | 0.0002062 | down |
| Chi3l1 | -1.39250258 | 7.88E-05 | 0.0002184 | down |
| C1qtnf4 | -1.139224849 | 7.99E-05 | 0.000221 | down |
| Sytl4 | -1.075936405 | 8.00E-05 | 0.0002213 | down |
| Slc39a4 | -2.048621416 | 8.02E-05 | 0.0002218 | down |
| Batf3 | -1.333553751 | 8.22E-05 | 0.000227 | down |
| Angptl6 | -1.094055021 | 8.25E-05 | 0.0002278 | down |
| Grp | -2.451802729 | 8.43E-05 | 0.0002322 | down |
| Vip | -4.188722207 | 8.85E-05 | 0.0002432 | down |
| Kmo | -1.342546402 | 9.28E-05 | 0.0002545 | down |
| H2-Q5 | -1.254232709 | 9.79E-05 | 0.0002674 | down |
| E330020D12Rik | -1.008570154 | 9.84E-05 | 0.0002687 | down |
| Sec1 | -1.881327596 | 0.0001004 | 0.0002737 | down |
| AI662270 | -1.075874113 | 0.0001013 | 0.000276 | down |
| Dnaic2 | -2.749696178 | 0.0001028 | 0.0002796 | down |
| Bmx | -1.163832846 | 0.0001034 | 0.0002811 | down |
| Pyhin1 | -1.344959505 | 0.0001068 | 0.0002895 | down |
| Slamf8 | -1.346618032 | 0.0001078 | 0.0002922 | down |
| C1rl | -1.067259768 | 0.0001109 | 0.0002998 | down |
| Otop1 | -2.493654783 | 0.0001143 | 0.0003083 | down |
| Upk3bl | -1.073125517 | 0.0001144 | 0.0003085 | down |
| Lipf | -3.924449136 | 0.000117 | 0.0003148 | down |
| Upk3a | -3.289458316 | 0.0001182 | 0.0003177 | down |
| Il8 | -2.80706018 | 0.0001215 | 0.000326 | down |
| Gm5820 | -1.239631209 | 0.0001295 | 0.0003458 | down |
| Tmem212 | -3.515974682 | 0.0001297 | 0.0003462 | down |
| Wdr63 | -3.019588571 | 0.0001309 | 0.000349 | down |
| Sftpd | -1.645717373 | 0.000132 | 0.0003516 | down |
| Tspan32 | -1.264014011 | 0.0001332 | 0.0003545 | down |
| Six1 | -1.453055342 | 0.0001383 | 0.0003669 | down |
| Plcb2 | -1.112562973 | 0.0001406 | 0.0003723 | down |
| D330045A20Rik | -1.181534377 | 0.0001461 | 0.0003857 | down |
| Pram1 | -1.076418107 | 0.0001495 | 0.0003938 | down |
| Foxj1 | -2.240573025 | 0.0001509 | 0.0003969 | down |
| Gm5431 | -1.269222205 | 0.0001551 | 0.0004076 | down |
| Iqcd | -1.123506354 | 0.0001593 | 0.0004179 | down |
| Qpct | -1.077167122 | 0.000162 | 0.0004244 | down |
| Ccdc108 | -2.997374745 | 0.0001656 | 0.0004333 | down |
| Rbpjl | -1.591756841 | 0.0001657 | 0.0004334 | down |
| Efhc1 | -1.076695272 | 0.0001683 | 0.0004399 | down |
| Scgb3a2 | -3.097068531 | 0.0001693 | 0.0004421 | down |
| 1700019L03Rik | -1.323228371 | 0.0001809 | 0.0004701 | down |
| Abcg3 | -1.455482005 | 0.0001875 | 0.0004862 | down |
| Gm14378 | -1.092535982 | 0.0001884 | 0.0004882 | down |
| Apol6 | -1.512140511 | 0.0001948 | 0.0005037 | down |
| Tnip3 | -1.755839729 | 0.0001955 | 0.0005051 | down |
| Naip6 | -1.535275407 | 0.0001956 | 0.0005054 | down |
| 2310065F04Rik | -4.51364655 | 0.0001957 | 0.0005055 | down |
| Slc38a4 | -1.559052535 | 0.0002007 | 0.0005177 | down |
| Cbr2 | -1.41525937 | 0.0002114 | 0.0005435 | down |
| Mcoln3 | -3.353578768 | 0.0002136 | 0.0005487 | down |
| Trem1 | -1.204596243 | 0.0002137 | 0.0005488 | down |
| Tnf | -1.695580259 | 0.0002204 | 0.0005649 | down |
| Pira6 | -1.852248381 | 0.0002311 | 0.0005899 | down |
| Abp1 | -2.567860101 | 0.0002376 | 0.000605 | down |
| Clic6 | -2.361689548 | 0.0002391 | 0.0006083 | down |
| Ccdc24 | -1.11302161 | 0.0002392 | 0.0006084 | down |
| H2-Q1 | -1.330614815 | 0.0002496 | 0.0006333 | down |
| Tnfrsf19 | -2.057519506 | 0.0002505 | 0.0006353 | down |
| Gm11127 | -1.069049022 | 0.0002632 | 0.0006654 | down |
| Oas1g | -1.428275657 | 0.0002721 | 0.0006866 | down |
| Sgsm1 | -1.162194133 | 0.0002788 | 0.0007015 | down |
| Zfp783 | -1.493818551 | 0.0002822 | 0.0007087 | down |
| Chst10 | -1.662252934 | 0.0002864 | 0.0007186 | down |
| Ebi3 | -1.117956597 | 0.000287 | 0.0007198 | down |
| Col17a1 | -2.193080168 | 0.0003033 | 0.0007575 | down |
| Dnahc7b | -1.270879277 | 0.000307 | 0.0007658 | down |
| Vwa3a | -1.197906024 | 0.0003107 | 0.0007741 | down |
| Itln1 | -2.927585282 | 0.0003118 | 0.0007762 | down |
| Fam26f | -1.695397804 | 0.0003169 | 0.0007876 | down |
| Lag3 | -1.166937759 | 0.0003182 | 0.0007907 | down |
| Capn3 | -2.288805029 | 0.0003201 | 0.0007947 | down |
| Gm14492 | -1.158426734 | 0.0003243 | 0.0008042 | down |
| H2-Q10 | -1.313379115 | 0.000359 | 0.0008833 | down |
| Abca6 | -1.417127977 | 0.0003594 | 0.0008843 | down |
| Asb16 | -1.501386007 | 0.0003706 | 0.0009097 | down |
| Cxcl13 | -1.96794147 | 0.0004091 | 0.0009976 | down |
| Rgs18 | -1.383816255 | 0.0004239 | 0.0010317 | down |
| Atp8b4 | -1.490310937 | 0.000426 | 0.0010362 | down |
| Akr1c13 | -1.850364729 | 0.0004363 | 0.0010597 | down |
| Gm10767 | -1.010290974 | 0.0004457 | 0.0010799 | down |
| Gm13476 | -1.077038201 | 0.0004483 | 0.0010856 | down |
| Dnali1 | -3.101546149 | 0.0004557 | 0.0011028 | down |
| 2810029C07Rik | -1.102196266 | 0.0004669 | 0.0011282 | down |
| 2310042D19Rik | -1.280532344 | 0.0004714 | 0.0011378 | down |
| Itgb7 | -1.116934266 | 0.0004724 | 0.0011402 | down |
| Syn2 | -2.385115807 | 0.0005003 | 0.0012017 | down |
| Cpn1 | -1.417679081 | 0.0005055 | 0.0012132 | down |
| Il1b | -1.766996265 | 0.0005169 | 0.0012389 | down |
| Cdo1 | -1.044420575 | 0.0005251 | 0.0012573 | down |
| Cd207 | -2.132038627 | 0.0005344 | 0.0012778 | down |
| Srd5a1 | -1.67065426 | 0.0005546 | 0.001323 | down |
| Gm11545 | -1.583549772 | 0.0005561 | 0.0013262 | down |
| Fam198a | -1.214545036 | 0.0005751 | 0.0013686 | down |
| Stap1 | -1.455449943 | 0.0005788 | 0.0013763 | down |
| Lypd2 | -1.16080849 | 0.0005977 | 0.0014186 | down |
| Cd163 | -1.006411786 | 0.0006013 | 0.0014268 | down |
| 5031414D18Rik | -1.176994881 | 0.0006049 | 0.0014348 | down |
| Tdrkh | -1.201084772 | 0.0006094 | 0.0014443 | down |
| Cyp2a5 | -2.854281006 | 0.000612 | 0.0014495 | down |
| 3110007F17Rik | -1.015010233 | 0.0006288 | 0.0014863 | down |
| Pvalb | -3.581928783 | 0.0006345 | 0.0014987 | down |
| Hbb-b1 | -2.508155532 | 0.0006467 | 0.0015247 | down |
| Abcb4 | -2.006222272 | 0.0006481 | 0.0015273 | down |
| Atp13a4 | -1.327239481 | 0.0006575 | 0.0015485 | down |
| BC051019 | -2.77988826 | 0.0006697 | 0.0015752 | down |
| Pgr | -1.319066689 | 0.0006833 | 0.0016053 | down |
| Dnahc2 | -1.894811054 | 0.0006861 | 0.0016113 | down |
| Sash3 | -1.005598003 | 0.0006964 | 0.0016321 | down |
| 2010107G12Rik | -1.728283064 | 0.0007177 | 0.0016773 | down |
| Tslp | -1.127131584 | 0.0007217 | 0.0016859 | down |
| Fmo3 | -2.358028715 | 0.0007258 | 0.0016945 | down |
| Cacnb2 | -1.402793268 | 0.0007709 | 0.0017917 | down |
| Gm10433 | -1.050845774 | 0.0007874 | 0.0018274 | down |
| Bpifa1 | -3.324606979 | 0.0007935 | 0.0018401 | down |
| Gm13845 | -1.077608417 | 0.0007944 | 0.0018416 | down |
| Negr1 | -1.315092699 | 0.0008111 | 0.0018781 | down |
| Myl3 | -4.995361633 | 0.0008968 | 0.0020635 | down |
| Pkib | -1.06689161 | 0.0009538 | 0.002182 | down |
| Mfsd2a | -1.646203259 | 0.0009907 | 0.0022572 | down |
| Kcnj11 | -2.968700786 | 0.0010757 | 0.0024375 | down |
| Dynlrb2 | -3.185998108 | 0.0011137 | 0.0025153 | down |
| Lcat | -1.052104898 | 0.001114 | 0.0025157 | down |
| Skap1 | -1.89472105 | 0.0011578 | 0.0026072 | down |
| Nmnat2 | -1.396838475 | 0.0011686 | 0.002628 | down |
| Cdhr3 | -2.792038285 | 0.0012136 | 0.0027212 | down |
| Lrrn1 | -1.817539427 | 0.0012383 | 0.0027726 | down |
| Greb1 | -1.230010629 | 0.0012779 | 0.0028533 | down |
| Cntfr | -1.747658813 | 0.001318 | 0.0029333 | down |
| Sox21 | -1.938199103 | 0.0013231 | 0.0029426 | down |
| Dapl1 | -1.892089151 | 0.0013536 | 0.0030058 | down |
| Adamdec1 | -1.955243065 | 0.0013656 | 0.0030287 | down |
| Cyp3a13 | -1.039983048 | 0.0013935 | 0.003085 | down |
| Crygs | -1.186647698 | 0.0014141 | 0.0031273 | down |
| 3-Sep | -1.250382289 | 0.0014323 | 0.0031622 | down |
| Serpina3n | -1.035715591 | 0.0014426 | 0.0031819 | down |
| Slc2a4 | -1.500528881 | 0.0014437 | 0.0031839 | down |
| Adig | -1.573600884 | 0.0014467 | 0.0031885 | down |
| Unc13a | -1.071453588 | 0.0014495 | 0.0031938 | down |
| Dnajb13 | -1.28313649 | 0.0015016 | 0.0032952 | down |
| Slc23a3 | -1.088353252 | 0.0015367 | 0.0033656 | down |
| Kcnrg | -1.784441404 | 0.0015501 | 0.0033928 | down |
| Dnahc6 | -1.488692193 | 0.0015595 | 0.0034107 | down |
| Ppbp | -1.536444517 | 0.0015727 | 0.0034366 | down |
| Tubb1 | -1.541413164 | 0.0016873 | 0.0036659 | down |
| Atp1a3 | -1.028706922 | 0.0017076 | 0.0037035 | down |
| Dhh | -1.256138238 | 0.0018019 | 0.0038889 | down |
| Wdr52 | -1.995939284 | 0.0018779 | 0.0040402 | down |
| Rtn2 | -1.150980825 | 0.0019449 | 0.0041689 | down |
| AU023871 | -1.187012962 | 0.0019959 | 0.0042649 | down |
| 4930452B06Rik | -1.361838419 | 0.001997 | 0.0042666 | down |
| Adam28 | -1.701382323 | 0.0020477 | 0.0043633 | down |
| Ndrg2 | -1.309444293 | 0.00206 | 0.0043857 | down |
| Camk2b | -1.876163304 | 0.0020925 | 0.00445 | down |
| Actn3 | -2.794150898 | 0.002098 | 0.0044602 | down |
| Kl | -3.270800461 | 0.0022021 | 0.0046581 | down |
| 9430037G07Rik | -1.336831952 | 0.0023705 | 0.0049897 | down |
| Pon1 | -1.506947902 | 0.0023725 | 0.0049932 | down |
| Tpm2 | -1.802621107 | 0.0024314 | 0.0051054 | down |
| Dnahc5 | -2.627049249 | 0.0024481 | 0.0051351 | down |
| AU040972 | -3.327384151 | 0.0024743 | 0.0051849 | down |
| Dmbt1 | -3.104348112 | 0.0024953 | 0.0052235 | down |
| Nrgn | -1.274701869 | 0.002519 | 0.0052693 | down |
| Rpl3l | -2.219569822 | 0.0025424 | 0.0053137 | down |
| Grip2 | -1.299680411 | 0.0025671 | 0.0053599 | down |
| Serpinb5 | -1.642537509 | 0.0028708 | 0.0059513 | down |
| Btnl2 | -1.166995033 | 0.0029214 | 0.0060465 | down |
| Tcf15 | -1.073753524 | 0.002998 | 0.0061866 | down |
| Vpreb3 | -1.005882795 | 0.003035 | 0.0062549 | down |
| 9330159F19Rik | -1.765793801 | 0.0030733 | 0.0063267 | down |
| Mylk4 | -2.810561289 | 0.0030786 | 0.0063349 | down |
| Dhrs7c | -2.885738558 | 0.0031107 | 0.0063943 | down |
| Shc3 | -1.103213647 | 0.0031571 | 0.0064782 | down |
| Ptprr | -1.105487462 | 0.0031899 | 0.0065417 | down |
| Ptger3 | -1.107036497 | 0.0033146 | 0.0067751 | down |
| Fabp7 | -2.51441528 | 0.0035556 | 0.0072272 | down |
| Cxcr6 | -1.131057307 | 0.003655 | 0.0074106 | down |
| D730005E14Rik | -1.998505916 | 0.0038698 | 0.0078048 | down |
| Krt14 | -2.089600125 | 0.0039653 | 0.0079762 | down |
| Tgm1 | -1.062987592 | 0.0040014 | 0.0080456 | down |
| Myom1 | -1.059429073 | 0.0040034 | 0.0080485 | down |
| Ppapdc3 | -1.159822361 | 0.0040805 | 0.0081853 | down |
| 1700001O22Rik | -1.035890297 | 0.0041057 | 0.0082267 | down |
| Sbk2 | -2.70045358 | 0.004317 | 0.0086097 | down |
| Rxrg | -1.31097401 | 0.0044369 | 0.0088221 | down |
| Rbm24 | -2.319693654 | 0.0045754 | 0.0090738 | down |
| Mak | -1.011086395 | 0.0045949 | 0.0091089 | down |
| Sec14l3 | -2.900668709 | 0.0046826 | 0.0092687 | down |
| Cyp2b10 | -2.375713918 | 0.0046867 | 0.0092756 | down |
| Chga | -2.958384528 | 0.0048995 | 0.0096493 | down |
| Prdm8 | -1.06797285 | 0.0049735 | 0.0097831 | down |
| Ttn | -2.474953128 | 0.0051845 | 0.0101638 | down |
| Kcnab1 | -1.100637049 | 0.0052068 | 0.010206 | down |
| Trim55 | -2.42707737 | 0.0053569 | 0.0104678 | down |
| Cryba2 | -3.025241419 | 0.0053893 | 0.0105268 | down |
| Padi1 | -1.231389378 | 0.0055097 | 0.0107345 | down |
| 9330159M07Rik | -1.407523395 | 0.0055104 | 0.0107345 | down |
| Kcna1 | -1.583648454 | 0.0056782 | 0.0110316 | down |
| Grap2 | -1.191856152 | 0.0056847 | 0.0110428 | down |
| Tbx1 | -1.671647236 | 0.0058529 | 0.0113359 | down |
| Ccl3 | -1.352867966 | 0.0058736 | 0.0113687 | down |
| Lctl | -1.137099719 | 0.0060561 | 0.0116969 | down |
| Gpnmb | -1.106585864 | 0.006316 | 0.0121601 | down |
| Sytl3 | -1.253840601 | 0.0065087 | 0.0124847 | down |
| Myh4 | -3.153370624 | 0.0065317 | 0.0125239 | down |
| Ctse | -1.015837639 | 0.0065531 | 0.0125565 | down |
| Pacsin1 | -1.096881867 | 0.0066644 | 0.0127345 | down |
| Slamf6 | -1.265442188 | 0.0066782 | 0.0127576 | down |
| Gm6377 | -1.188804822 | 0.0069058 | 0.0131628 | down |
| Pgam2 | -1.860826789 | 0.0069525 | 0.0132432 | down |
| Fbp2 | -1.032909497 | 0.007027 | 0.0133692 | down |
| Isl1 | -1.095429428 | 0.0070388 | 0.0133865 | down |
| Tnni2 | -2.509831594 | 0.0070544 | 0.0134126 | down |
| Ifitm10 | -1.014049351 | 0.0070935 | 0.01348 | down |
| Padi4 | -1.420372868 | 0.0072211 | 0.013699 | down |
| Scn4b | -1.571560246 | 0.0072464 | 0.0137435 | down |
| Xcr1 | -1.174719911 | 0.0072694 | 0.0137834 | down |
| Ckmt2 | -2.959947889 | 0.0078196 | 0.0147476 | down |
| BC048546 | -2.535231746 | 0.0078493 | 0.0147999 | down |
| Pygo1 | -1.131460057 | 0.0079388 | 0.0149512 | down |
| Cidea | -1.472096445 | 0.0080257 | 0.0150991 | down |
| Fgf13 | -1.453819103 | 0.0083786 | 0.0157019 | down |
| Aox3 | -1.679410807 | 0.0087261 | 0.0162774 | down |
| Scgb1a1 | -1.141390172 | 0.0090204 | 0.0167853 | down |
| Kbtbd13 | -1.856348316 | 0.0092701 | 0.0171881 | down |
| Casq1 | -2.235169162 | 0.0093237 | 0.0172723 | down |
| Popdc2 | -1.388416795 | 0.0097183 | 0.017946 | down |
| Tmod4 | -1.12124437 | 0.0101004 | 0.0185547 | down |
| Klhl38 | -1.397188322 | 0.0101063 | 0.0185632 | down |
| Myoc | -1.524276067 | 0.0105842 | 0.0193791 | down |
| Mlf1 | -1.283219654 | 0.0107231 | 0.0196068 | down |
| Prkcq | -1.55359636 | 0.0108981 | 0.0198917 | down |
| Ampd1 | -2.626843155 | 0.0110713 | 0.0201696 | down |
| Acsm3 | -1.354049708 | 0.0111335 | 0.020278 | down |
| Slc22a4 | -1.209910374 | 0.011442 | 0.0207824 | down |
| Ldb3 | -2.077589276 | 0.0120697 | 0.0218024 | down |
| 2310002L09Rik | -2.565153837 | 0.0121139 | 0.0218714 | down |
| 1-Sep | -1.027371326 | 0.012594 | 0.0226284 | down |
| Clec4b1 | -1.101255982 | 0.0126821 | 0.0227696 | down |
| Cmya5 | -1.925452877 | 0.012777 | 0.0229214 | down |
| Mypn | -2.594208579 | 0.0129388 | 0.023176 | down |
| Esrrg | -2.37005578 | 0.0130764 | 0.0234023 | down |
| Myoz1 | -2.46165005 | 0.0134615 | 0.0240115 | down |
| Osm | -1.172362111 | 0.013653 | 0.0243023 | down |
| Cadps | -1.16654847 | 0.0137473 | 0.0244575 | down |
| Stac3 | -1.451730174 | 0.0139996 | 0.0248589 | down |
| Ky | -1.856710445 | 0.0142356 | 0.0252403 | down |
| Dusp13 | -1.612845144 | 0.0143589 | 0.0254431 | down |
| Mylk2 | -1.90221447 | 0.0149542 | 0.0263822 | down |
| Myadml2 | -1.803859301 | 0.0149865 | 0.0264294 | down |
| Cd2 | -1.267631812 | 0.0150195 | 0.026478 | down |
| P2ry10 | -1.253306602 | 0.0151725 | 0.0267282 | down |
| Spag16 | -1.976958943 | 0.0154475 | 0.0271698 | down |
| Fxyd2 | -1.084821426 | 0.0154633 | 0.0271943 | down |
| Ptprz1 | -1.575546384 | 0.015468 | 0.0271993 | down |
| Sgca | -2.382104202 | 0.0156356 | 0.0274608 | down |
| Spink8 | -2.394729551 | 0.0167136 | 0.0291493 | down |
| Fabp3 | -1.39404281 | 0.0171307 | 0.029798 | down |
| Olfr1396 | -1.005879514 | 0.0172469 | 0.029975 | down |
| Atp2a1 | -2.534283962 | 0.0176988 | 0.0306905 | down |
| Acss3 | -1.560703802 | 0.0183836 | 0.0318095 | down |
| Sirpb1a | -1.42412992 | 0.0189596 | 0.0327086 | down |
| P2rx5 | -1.071665516 | 0.0194452 | 0.0334509 | down |
| 2310069B03Rik | -1.572511089 | 0.0194671 | 0.0334846 | down |
| Mettl21e | -2.137667394 | 0.0198439 | 0.034068 | down |
| Hrc | -1.877237683 | 0.0201288 | 0.0345041 | down |
| Cyp4a12b | -2.282574621 | 0.0203049 | 0.0347731 | down |
| Mylpf | -2.092750793 | 0.023574 | 0.0397106 | down |
| Alpk2 | -1.395697243 | 0.023845 | 0.0401083 | down |
| Hba-a2 | -1.277412398 | 0.0258494 | 0.0431032 | down |
| Tcap | -2.06879501 | 0.0262727 | 0.0437385 | down |
| Ckm | -2.310069705 | 0.026675 | 0.0443371 | down |
| Jsrp1 | -1.847176789 | 0.0267758 | 0.044469 | down |
| Ccdc164 | -1.349180245 | 0.0277142 | 0.0458594 | down |
| Eno3 | -1.22866939 | 0.0279895 | 0.0462516 | down |
| Dmrt2 | -1.361155111 | 0.0286171 | 0.0471867 | down |
| Fitm1 | -1.9957989 | 0.0294145 | 0.0483367 | down |
| Slc4a1 | -1.249111124 | 0.0304941 | 0.0499242 | down |

**Table S5: Comparison of gene expression changes in human PPFP thyroid carcinomas versus mouse PPFP thyroid carcinomas.** Genes previously found to be differentially expressed in human PPFP thyroid carcinomas versus other thyroid neoplasms and normal thyroids [8] were assessed for differential expression in PPFP^Thy^;Pten^Thy-/-^ mice versus Pten^Thy-/-^ control mice fed normal chow (-pio), and in PPFP^Thy^;Pten^Thy-/-^ mice fed pioglitazone versus normal chow. We define mouse genes to be induced if log fold change >0, and repressed if log fold change <0.

| **Symbol** | **Human PPFP carcinomas** | **PPFP^Thy^;Pten^Thy-/-^ mice –pio vs. Pten^Thy-/-^ mice –pio** | **PPFP^Thy^;Pten^Thy-/-^ mice +pio vs. PPFP^Thy^;Pten^Thy-/-^ mice -pio** |
| --- | --- | --- | --- |
| C12orf49 | down | up | down |
| C3orf14 | down | down | down |
| ABCC3 | up | up | up |
| ABCC9 | up | up | down |
| ACAA1 | up | up | up |
| ACADM | up | up | up |
| ACADS | up | up | down |
| ACSF2 | up | down | up |
| ACY1 | up | up | down |
| ADAM9 | up | up | up |
| AGL | up | up | down |
| AGPAT3 | up | up | down |
| ALDH5A1 | down | down | up |
| AMPD2 | up | up | down |
| ANGPTL4 | up | up | up |
| ANKS1A | up | down | down |
| AP1S1 | up | up | up |
| APBB2 | up | up | down |
| APOL6 | up | down | up |
| AQP7 | up | down | up |
| ARG2 | down | down | down |
| ARID5B | up | down | up |
| ASPA | up | down | up |
| ASS1 | up | up | up |
| ATP10B | up | up | down |
| ATP6V0E2 | down | down | up |
| ATP8A1 | down | up | down |
| BBS9 | down | down | up |
| BCL2L1 | up | down | up |
| CACNA1G | up | down | up |
| CACNA2D2 | down | down | down |
| CADM1 | down | down | up |
| CAMKK2 | up | up | down |
| CAND2 | down | up | down |
| CBR4 | up | up | up |
| CBS | down | down | up |
| CEP112 | down | down | up |
| CERS6 | down | down | down |
| CHPT1 | down | down | up |
| CLCNKA | up | down | down |
| CNR1 | up | up | down |
| COL13A1 | up | down | down |
| COL4A1 | up | up | up |
| COL4A2 | up | up | down |
| CUEDC1 | down | down | down |
| CXCR4 | up | up | down |
| CYB5R2 | up | up | up |
| DBP | down | down | up |
| DECR2 | up | down | up |
| DENND3 | up | down | down |
| DENND4A | up | up | up |
| DHCR24 | up | down | down |
| DNASE1 | up | up | down |
| DSP | up | down | down |
| EDIL3 | up | up | up |
| EHD1 | up | up | down |
| EIF4EBP2 | up | up | up |
| ENO2 | down | down | up |
| ENO3 | up | down | up |
| ENTPD5 | up | up | up |
| EPHB2 | down | up | down |
| EPS8L1 | up | down | down |
| EPS8L2 | up | down | down |
| ESD | down | down | up |
| ESRP2 | up | up | down |
| ETFB | up | up | up |
| EYA2 | down | down | up |
| F13A1 | up | up | up |
| F5 | up | down | down |
| FAM174B | down | up | down |
| FAT2 | up | up | down |
| FBN1 | up | up | up |
| FBN2 | up | up | up |
| FBP1 | up | up | up |
| FDFT1 | up | down | down |
| FERMT1 | up | down | down |
| FHL2 | up | down | up |
| FZD5 | up | down | down |
| GADD45G | up | up | up |
| GALNT6 | up | down | up |
| GDF5 | up | up | up |
| GFAP | up | down | down |
| GLRX | up | down | down |
| GMDS | up | up | down |
| GNG11 | up | up | up |
| GPD1 | up | up | up |
| GPR153 | up | down | up |
| GPRASP1 | down | up | down |
| GRK5 | up | up | down |
| GSTM3 | up | down | down |
| HIC2 | up | down | down |
| HK2 | down | up | up |
| HPN | down | down | down |
| HSPB7 | up | down | up |
| IGFBP2 | up | up | up |
| INF2 | up | down | up |
| INPP4B | up | up | up |
| INTS8 | up | up | down |
| IPCEF1 | up | up | down |
| ITPR1 | up | up | down |
| KDM1A | up | down | up |
| KLF8 | up | down | up |
| KLHDC2 | up | down | down |
| LAMA4 | up | up | down |
| LARP6 | down | down | down |
| LBH | down | up | up |
| LPAR2 | up | up | down |
| MAN1C1 | down | up | up |
| MARCH1 | up | up | up |
| MBOAT2 | down | down | down |
| MET | down | down | down |
| MFAP3L | up | up | down |
| MFSD9 | up | up | up |
| MGST3 | up | up | up |
| MID1IP1 | down | up | down |
| MLPH | down | down | down |
| MME | up | up | up |
| MRPL35 | down | up | down |
| MXI1 | down | down | up |
| MYOZ1 | up | down | up |
| NAP1L3 | down | up | down |
| NFE2L3 | up | up | up |
| NID1 | up | up | up |
| NMB | up | up | up |
| NR2F6 | up | up | up |
| PALLD | up | down | down |
| PARP3 | up | down | up |
| PAWR | up | down | up |
| PBX3 | down | down | up |
| PCP4 | down | down | down |
| PC | up | up | down |
| PDE8B | down | down | down |
| PDGFRL | down | up | up |
| PDZK1 | up | up | down |
| PFKL | up | up | down |
| PGF | up | down | up |
| PHF2 | down | up | down |
| PHKB | up | up | down |
| PIGH | down | down | down |
| PKP4 | down | down | down |
| PLA2G15 | up | down | up |
| PLBD1 | up | up | up |
| PLEKHB1 | down | down | down |
| PLEKHG3 | down | down | up |
| PLIN1 | up | down | up |
| PLS1 | up | up | down |
| PLXNA1 | up | up | up |
| PLXNB2 | up | down | up |
| PML | up | down | up |
| PMP22 | up | down | down |
| POU6F1 | up | down | down |
| PPARG | up | up | down |
| PRKCA | down | down | up |
| PSD3 | down | down | up |
| PTPN21 | up | down | down |
| PTTG1 | down | down | up |
| PXDN | up | up | up |
| RAB20 | up | down | up |
| RAB4A | up | down | down |
| RAD52 | up | down | down |
| RAP1GAP2 | up | up | up |
| RASL11B | up | up | down |
| RASSF4 | up | down | up |
| RBFOX1 | up | down | down |
| RCAN1 | up | up | up |
| REPS2 | up | down | down |
| RHOB | up | down | down |
| RNF220 | down | down | down |
| RSU1 | up | down | up |
| RWDD3 | up | down | down |
| S100A5 | up | up | down |
| SALL1 | down | down | down |
| SCNN1A | up | down | down |
| SCNN1B | up | down | down |
| SECISBP2L | up | up | up |
| SEPT8 | up | down | down |
| SERPINE2 | down | up | up |
| SH3BP4 | up | down | down |
| SLC15A1 | up | up | down |
| SLC19A1 | up | up | up |
| SLC1A1 | down | down | down |
| SLC25A20 | up | up | up |
| SLC26A4 | down | down | up |
| SLC38A1 | down | down | down |
| SLC39A8 | up | down | up |
| SLC44A4 | up | down | down |
| SLC7A7 | up | down | up |
| SLC7A8 | up | down | up |
| SLC9A1 | up | down | up |
| SLK | up | up | up |
| SMS | down | down | down |
| SORD | up | down | up |
| SSX2IP | down | up | down |
| STBD1 | up | up | up |
| SV2A | down | down | up |
| TBC1D9 | up | up | down |
| TCF7L1 | up | down | down |
| TES | up | up | down |
| TFEB | down | down | down |
| TFPI2 | up | up | up |
| THBS1 | up | down | down |
| TIMP3 | down | up | down |
| TKT | up | up | down |
| TLR4 | up | down | up |
| TM7SF2 | up | down | down |
| TMBIM1 | up | down | up |
| TMBIM6 | up | down | up |
| TMEFF1 | up | up | down |
| TMEM135 | up | up | down |
| TMOD1 | down | down | down |
| TMX4 | down | down | up |
| TNFRSF21 | up | down | up |
| TNFSF12 | down | down | up |
| TOR1AIP1 | down | down | up |
| TRIL | up | up | down |
| TRPV6 | up | up | up |
| TSPO | up | down | up |
| TSPYL5 | up | up | down |
| TUBA4A | down | up | down |
| UCP2 | up | down | up |
| VAPB | up | down | up |
| VWF | down | down | up |
| WFS1 | up | down | down |
| XK | up | down | down |
| ZDHHC13 | up | up | down |
| ZMAT3 | up | up | up |

**Table S6: Thyroid genes differentially expressed in PPFP^Thy^;Pten^Thy-/-^ mice fed pioglitazone versus control diet, using *q*<0.05 and absolute fold change >2 as cut-offs for significance**

| **Gene Symbol** | **Log(Fold Change)** | ***p*-value** | ***q*-value** | **Pioglitazone vs control diet** |
| --- | --- | --- | --- | --- |
| Ctss | 4.2351077 | 7.32E-115 | 2.66E-111 | up |
| Sirpa | 3.0439286 | 1.39E-103 | 3.36E-100 | up |
| Trem2 | 3.4613026 | 2.40E-83 | 2.67E-80 | up |
| Tyrobp | 3.2277595 | 1.23E-77 | 1.05E-74 | up |
| Mpeg1 | 3.5587758 | 5.83E-71 | 4.16E-68 | up |
| Lyz2 | 4.132943 | 2.22E-68 | 1.40E-65 | up |
| Cd300a | 3.2857166 | 7.50E-66 | 4.35E-63 | up |
| Apoe | 3.6206493 | 4.87E-62 | 2.62E-59 | up |
| C1qb | 2.4527794 | 9.16E-62 | 4.58E-59 | up |
| Pltp | 2.1376886 | 1.38E-61 | 6.67E-59 | up |
| AF251705 | 4.3630319 | 3.79E-60 | 1.72E-57 | up |
| Lpl | 4.5964797 | 2.05E-59 | 8.75E-57 | up |
| Lyz1 | 4.0079686 | 4.33E-59 | 1.76E-56 | up |
| Ncf2 | 3.2224827 | 4.36E-59 | 1.76E-56 | up |
| Cybb | 2.993119 | 1.22E-58 | 4.79E-56 | up |
| Cd84 | 3.7020975 | 2.28E-57 | 8.28E-55 | up |
| Clec7a | 5.4310197 | 1.03E-56 | 3.54E-54 | up |
| Angptl4 | 3.6222952 | 2.49E-56 | 8.39E-54 | up |
| Myo1f | 3.1332877 | 9.72E-56 | 3.21E-53 | up |
| C1qc | 2.237766 | 2.54E-53 | 7.69E-51 | up |
| Itgax | 5.0040192 | 1.27E-52 | 3.69E-50 | up |
| Fcgr3 | 3.0025357 | 1.33E-52 | 3.79E-50 | up |
| Laptm5 | 2.8723564 | 2.03E-51 | 5.67E-49 | up |
| Fcer1g | 2.9595284 | 9.78E-51 | 2.58E-48 | up |
| Lgals3 | 3.5992974 | 4.99E-50 | 1.25E-47 | up |
| Ms4a7 | 2.6232773 | 4.93E-50 | 1.25E-47 | up |
| Tnfrsf1b | 2.7298334 | 1.44E-49 | 3.48E-47 | up |
| Wfdc13 | 5.6973905 | 3.39E-48 | 7.80E-46 | up |
| Cyth4 | 2.4147178 | 1.68E-47 | 3.80E-45 | up |
| C1qa | 2.3805413 | 2.01E-47 | 4.48E-45 | up |
| Nkain1 | 4.3954344 | 3.66E-47 | 7.94E-45 | up |
| Hvcn1 | 2.2564033 | 3.68E-47 | 7.94E-45 | up |
| Tcirg1 | 2.3102557 | 4.68E-47 | 9.84E-45 | up |
| Lat2 | 2.855946 | 9.54E-47 | 1.98E-44 | up |
| Nckap1l | 2.647275 | 9.65E-46 | 1.82E-43 | up |
| Cd68 | 3.1447939 | 9.75E-45 | 1.77E-42 | up |
| Basp1 | 3.8990267 | 1.52E-44 | 2.68E-42 | up |
| Efnb1 | 1.3402907 | 2.17E-44 | 3.80E-42 | up |
| Acp5 | 3.99987 | 1.66E-43 | 2.83E-41 | up |
| Cyp4v3 | 2.356037 | 6.72E-43 | 1.10E-40 | up |
| Cd53 | 2.8754074 | 1.92E-42 | 3.09E-40 | up |
| Cpe | 2.2775265 | 2.74E-42 | 4.23E-40 | up |
| Irf5 | 2.8010965 | 3.44E-42 | 5.25E-40 | up |
| Ctsk | 2.735388 | 5.36E-42 | 8.10E-40 | up |
| C3ar1 | 2.5736818 | 6.43E-42 | 9.61E-40 | up |
| Pik3r5 | 3.3169173 | 1.31E-41 | 1.92E-39 | up |
| Fam105a | 2.7164578 | 2.69E-41 | 3.83E-39 | up |
| Itgb2 | 3.513224 | 2.88E-41 | 4.05E-39 | up |
| Fabp4 | 4.2734631 | 2.92E-41 | 4.07E-39 | up |
| Pld4 | 2.4445469 | 3.33E-41 | 4.61E-39 | up |
| Fgr | 4.0257562 | 3.53E-41 | 4.83E-39 | up |
| Csf1r | 1.8073217 | 7.18E-41 | 9.50E-39 | up |
| Trf | 3.0709453 | 2.67E-40 | 3.45E-38 | up |
| Slc11a1 | 3.1080132 | 3.12E-40 | 4.01E-38 | up |
| Slc6a6 | 2.0650138 | 4.47E-40 | 5.63E-38 | up |
| Cxcl16 | 2.169289 | 6.70E-40 | 8.31E-38 | up |
| Aebp1 | 2.5461624 | 2.31E-39 | 2.77E-37 | up |
| Nfam1 | 2.6707247 | 2.61E-39 | 3.11E-37 | up |
| Steap3 | 2.4298065 | 3.12E-39 | 3.65E-37 | up |
| Unc93b1 | 1.9001048 | 3.22E-39 | 3.74E-37 | up |
| Gpnmb | 7.1029873 | 3.86E-39 | 4.45E-37 | up |
| 2200002D01Rik | 3.5074647 | 4.27E-39 | 4.88E-37 | up |
| Chi3l3 | 8.1802734 | 1.36E-38 | 1.52E-36 | up |
| Mmp12 | 6.16822 | 1.50E-38 | 1.66E-36 | up |
| Apobec1 | 2.4516977 | 1.76E-38 | 1.94E-36 | up |
| Sfpi1 | 2.8537847 | 3.33E-38 | 3.57E-36 | up |
| Cd22 | 4.0078353 | 5.78E-38 | 6.04E-36 | up |
| Ear2 | 9.6784192 | 2.92E-37 | 2.97E-35 | up |
| 4632428N05Rik | 2.0768258 | 6.58E-37 | 6.58E-35 | up |
| Ptprc | 2.7247988 | 7.40E-37 | 7.35E-35 | up |
| Pianp | 1.8593154 | 1.54E-36 | 1.50E-34 | up |
| Selplg | 3.0005064 | 1.65E-36 | 1.60E-34 | up |
| Mxra8 | 2.0375586 | 2.66E-36 | 2.53E-34 | up |
| Tifab | 2.3924844 | 3.04E-36 | 2.87E-34 | up |
| Tlr13 | 2.5479905 | 4.79E-36 | 4.49E-34 | up |
| Lilrb3 | 2.8856179 | 5.71E-36 | 5.31E-34 | up |
| Abcg1 | 2.510476 | 7.51E-36 | 6.94E-34 | up |
| Arl11 | 3.410801 | 7.76E-36 | 7.13E-34 | up |
| Cd300lb | 3.7828774 | 1.10E-35 | 9.98E-34 | up |
| Ubd | 7.7914272 | 1.38E-35 | 1.25E-33 | up |
| Pvrl3 | 2.5505539 | 1.60E-35 | 1.43E-33 | up |
| Atp1a3 | 4.5355951 | 2.70E-35 | 2.35E-33 | up |
| Arrb2 | 2.0371897 | 2.89E-35 | 2.50E-33 | up |
| Bmi1 | 1.2940655 | 3.40E-35 | 2.92E-33 | up |
| Id2 | 2.1540364 | 7.22E-35 | 6.17E-33 | up |
| Dpep2 | 2.3140863 | 9.99E-35 | 8.47E-33 | up |
| Tmem86a | 1.611 | 3.96E-34 | 3.26E-32 | up |
| Lilrb4 | 3.1734801 | 5.02E-34 | 4.04E-32 | up |
| Plek | 2.6860385 | 5.30E-34 | 4.22E-32 | up |
| Coro1a | 2.630371 | 5.55E-34 | 4.37E-32 | up |
| Tgfb3 | 1.5679096 | 6.11E-34 | 4.79E-32 | up |
| Hsd17b11 | 1.5605504 | 1.63E-33 | 1.26E-31 | up |
| Ctsz | 1.6412257 | 1.79E-33 | 1.37E-31 | up |
| Alox5ap | 2.5866791 | 2.29E-33 | 1.74E-31 | up |
| Ncbp1 | 1.6612286 | 2.90E-33 | 2.19E-31 | up |
| Uap1l1 | 2.2723654 | 4.05E-33 | 3.02E-31 | up |
| Amz1 | 2.90827 | 4.53E-33 | 3.34E-31 | up |
| Il7r | 4.1837685 | 8.54E-33 | 6.26E-31 | up |
| Slc37a2 | 3.0542882 | 9.81E-33 | 7.11E-31 | up |
| Ms4a6d | 2.6324639 | 1.18E-32 | 8.50E-31 | up |
| Evi2a | 2.6336655 | 1.54E-32 | 1.10E-30 | up |
| Cacna1g | 3.1622578 | 1.94E-32 | 1.38E-30 | up |
| Cd48 | 3.237694 | 2.21E-32 | 1.55E-30 | up |
| Tgfb2 | 2.6943553 | 2.41E-32 | 1.68E-30 | up |
| Fam49a | 2.1115324 | 2.51E-32 | 1.74E-30 | up |
| Dock2 | 2.6684657 | 5.50E-32 | 3.75E-30 | up |
| Havcr2 | 2.8728584 | 5.72E-32 | 3.87E-30 | up |
| Il10ra | 2.3906413 | 6.06E-32 | 4.09E-30 | up |
| Btbd11 | 4.4825279 | 6.12E-32 | 4.11E-30 | up |
| Efhd2 | 1.5353252 | 6.26E-32 | 4.19E-30 | up |
| Lipa | 1.4246629 | 1.04E-31 | 6.92E-30 | up |
| Meis3 | 2.2166048 | 1.77E-31 | 1.14E-29 | up |
| H2-DMb1 | 2.1737092 | 2.07E-31 | 1.33E-29 | up |
| Sel1l3 | 3.3971764 | 2.49E-31 | 1.60E-29 | up |
| Pdk4 | 2.9304903 | 3.90E-31 | 2.49E-29 | up |
| Fcgr2b | 1.9299056 | 4.29E-31 | 2.73E-29 | up |
| Csf2rb | 3.8746204 | 4.37E-31 | 2.77E-29 | up |
| 1300002K09Rik | 6.8814445 | 4.50E-31 | 2.84E-29 | up |
| Tnfaip8l2 | 2.7363898 | 5.02E-31 | 3.15E-29 | up |
| Dclk1 | 2.3863163 | 5.45E-31 | 3.41E-29 | up |
| Syk | 2.6002118 | 5.88E-31 | 3.66E-29 | up |
| Clec12a | 3.5643049 | 6.23E-31 | 3.86E-29 | up |
| Fermt3 | 2.401536 | 6.69E-31 | 4.13E-29 | up |
| Il1rn | 4.8306073 | 7.49E-31 | 4.59E-29 | up |
| Cd83 | 2.8438297 | 9.41E-31 | 5.66E-29 | up |
| Icam1 | 1.7848614 | 1.35E-30 | 8.11E-29 | up |
| Mmp11 | 1.9569833 | 1.36E-30 | 8.13E-29 | up |
| Lrp1 | 1.7675878 | 1.71E-30 | 1.01E-28 | up |
| Pla2g15 | 1.3528289 | 2.06E-30 | 1.21E-28 | up |
| Csf2rb2 | 3.8418239 | 2.21E-30 | 1.29E-28 | up |
| B430306N03Rik | 3.3399581 | 3.49E-30 | 2.00E-28 | up |
| Mdk | 1.9543712 | 3.91E-30 | 2.22E-28 | up |
| Pcdh7 | 1.9707867 | 4.38E-30 | 2.46E-28 | up |
| Tnfrsf9 | 2.427753 | 5.76E-30 | 3.21E-28 | up |
| Ly9 | 4.2964735 | 6.20E-30 | 3.43E-28 | up |
| Kctd12 | 1.4438043 | 7.52E-30 | 4.15E-28 | up |
| Cd300lf | 3.6535976 | 1.12E-29 | 6.18E-28 | up |
| Hk3 | 3.2020762 | 1.46E-29 | 8.01E-28 | up |
| Igsf6 | 2.5969202 | 1.83E-29 | 9.95E-28 | up |
| Fcgr1 | 2.3139117 | 2.24E-29 | 1.21E-27 | up |
| Arhgap30 | 2.5219445 | 2.76E-29 | 1.48E-27 | up |
| P2ry6 | 2.2306145 | 9.02E-29 | 4.69E-27 | up |
| Cisd3 | 1.6756802 | 9.78E-29 | 5.07E-27 | up |
| Sdc1 | 1.5067244 | 1.56E-28 | 8.05E-27 | up |
| Tmsb4x | 1.8754942 | 1.82E-28 | 9.26E-27 | up |
| Vav1 | 2.5640768 | 2.35E-28 | 1.19E-26 | up |
| Rap2b | 1.2307476 | 2.55E-28 | 1.28E-26 | up |
| Rgs10 | 2.1483495 | 2.98E-28 | 1.49E-26 | up |
| Plin1 | 4.5472001 | 3.21E-28 | 1.59E-26 | up |
| Cd52 | 3.3657637 | 3.22E-28 | 1.59E-26 | up |
| Cd40 | 3.0832746 | 3.71E-28 | 1.82E-26 | up |
| Fabp5 | 4.3339535 | 4.38E-28 | 2.13E-26 | up |
| Fhl2 | 1.9614289 | 4.40E-28 | 2.13E-26 | up |
| Cebpb | 2.259133 | 6.14E-28 | 2.96E-26 | up |
| Arhgdib | 1.9675133 | 8.60E-28 | 4.12E-26 | up |
| Commd3 | 1.2099626 | 1.04E-27 | 4.92E-26 | up |
| Slc34a2 | 3.8580145 | 1.07E-27 | 5.04E-26 | up |
| Emr1 | 2.0774885 | 1.12E-27 | 5.29E-26 | up |
| Apobr | 3.1769184 | 1.23E-27 | 5.77E-26 | up |
| Olfm1 | 2.1090503 | 1.37E-27 | 6.39E-26 | up |
| Eef2k | 2.1054944 | 1.41E-27 | 6.58E-26 | up |
| Cntn4 | 4.8351895 | 1.68E-27 | 7.71E-26 | up |
| Parvg | 2.5717075 | 1.80E-27 | 8.19E-26 | up |
| Itgb5 | 1.2401666 | 2.16E-27 | 9.82E-26 | up |
| Plxnc1 | 2.5828632 | 2.19E-27 | 9.94E-26 | up |
| Ccdc88b | 2.4938358 | 2.27E-27 | 1.02E-25 | up |
| Tnfsf12 | 1.7410172 | 2.44E-27 | 1.10E-25 | up |
| Serpinb6b | 2.9924886 | 2.71E-27 | 1.22E-25 | up |
| Epor | 2.885535 | 2.98E-27 | 1.34E-25 | up |
| Pqlc3 | 1.8175058 | 3.16E-27 | 1.40E-25 | up |
| Lyl1 | 2.4132103 | 4.45E-27 | 1.95E-25 | up |
| Zeb2 | 1.8660541 | 6.02E-27 | 2.63E-25 | up |
| Fam107b | 2.4016153 | 6.05E-27 | 2.64E-25 | up |
| Neurl3 | 2.3119583 | 6.88E-27 | 2.98E-25 | up |
| Ptpro | 2.3765779 | 7.87E-27 | 3.38E-25 | up |
| Lfng | 2.3386396 | 8.37E-27 | 3.58E-25 | up |
| Cotl1 | 1.7494819 | 8.76E-27 | 3.74E-25 | up |
| Ccr2 | 2.4812666 | 1.17E-26 | 4.93E-25 | up |
| Cpxm1 | 2.9050514 | 1.22E-26 | 5.13E-25 | up |
| Clec4n | 4.0914944 | 1.33E-26 | 5.59E-25 | up |
| Csf2ra | 1.6709553 | 1.54E-26 | 6.42E-25 | up |
| Lpcat2 | 2.3211403 | 1.65E-26 | 6.87E-25 | up |
| Mmp2 | 2.6928012 | 1.89E-26 | 7.84E-25 | up |
| Clec4d | 3.7052559 | 3.33E-26 | 1.37E-24 | up |
| H6pd | 1.2856395 | 5.04E-26 | 2.06E-24 | up |
| AI662270 | 3.1949836 | 5.18E-26 | 2.12E-24 | up |
| Tbxas1 | 2.9232827 | 5.45E-26 | 2.22E-24 | up |
| Slamf9 | 2.1407892 | 5.77E-26 | 2.34E-24 | up |
| Ttc36 | 2.8287502 | 6.12E-26 | 2.48E-24 | up |
| Adam8 | 3.4271167 | 7.69E-26 | 3.10E-24 | up |
| Csf3r | 2.6067791 | 9.72E-26 | 3.89E-24 | up |
| Btk | 2.6892629 | 1.14E-25 | 4.53E-24 | up |
| Mettl7b | 4.6671978 | 1.26E-25 | 4.99E-24 | up |
| Gatm | 1.5583964 | 1.43E-25 | 5.62E-24 | up |
| Npl | 2.2979626 | 1.58E-25 | 6.21E-24 | up |
| Lgmn | 1.3370785 | 1.60E-25 | 6.27E-24 | up |
| Hp | 2.5658286 | 1.79E-25 | 7.00E-24 | up |
| Il4ra | 1.2871371 | 2.84E-25 | 1.10E-23 | up |
| Ggt5 | 1.9440835 | 2.99E-25 | 1.15E-23 | up |
| Ms4a6c | 1.9540787 | 4.10E-25 | 1.56E-23 | up |
| Matk | 2.6266228 | 4.37E-25 | 1.66E-23 | up |
| Runx1 | 2.194515 | 5.34E-25 | 2.02E-23 | up |
| C5ar2 | 3.0908343 | 6.52E-25 | 2.46E-23 | up |
| C1qtnf1 | 1.8721607 | 9.10E-25 | 3.38E-23 | up |
| Cd14 | 2.1805882 | 1.07E-24 | 3.97E-23 | up |
| Slc15a3 | 2.7518546 | 1.15E-24 | 4.26E-23 | up |
| Kif26b | 3.4524174 | 1.23E-24 | 4.52E-23 | up |
| Sh3bgrl | 1.0048251 | 1.27E-24 | 4.64E-23 | up |
| Arhgap9 | 2.7041549 | 1.40E-24 | 5.09E-23 | up |
| Sema4a | 2.1656396 | 1.56E-24 | 5.65E-23 | up |
| Rxrg | 5.3959572 | 1.59E-24 | 5.74E-23 | up |
| Gadd45b | 1.864751 | 1.66E-24 | 5.97E-23 | up |
| Tspan4 | 1.564039 | 1.96E-24 | 6.98E-23 | up |
| Wfdc8 | 4.2354797 | 3.29E-24 | 1.16E-22 | up |
| Stom | 1.6253875 | 4.06E-24 | 1.42E-22 | up |
| Scara3 | 2.0911187 | 6.89E-24 | 2.36E-22 | up |
| Sh3pxd2b | 1.7768638 | 7.30E-24 | 2.49E-22 | up |
| Nudt16 | 1.8974636 | 7.86E-24 | 2.67E-22 | up |
| Slc7a2 | 2.632048 | 8.13E-24 | 2.75E-22 | up |
| Mpp1 | 1.1700574 | 1.05E-23 | 3.53E-22 | up |
| Rassf4 | 1.4927626 | 1.37E-23 | 4.59E-22 | up |
| Dbndd2 | 1.2834168 | 1.38E-23 | 4.61E-22 | up |
| Nupr1 | 2.21914 | 1.53E-23 | 5.09E-22 | up |
| Rnf145 | 1.2655172 | 1.64E-23 | 5.44E-22 | up |
| Ikbke | 1.6159835 | 1.72E-23 | 5.70E-22 | up |
| Glipr1 | 3.0883778 | 1.88E-23 | 6.19E-22 | up |
| Samhd1 | 1.0304998 | 1.88E-23 | 6.19E-22 | up |
| Rnase4 | 1.5953793 | 2.38E-23 | 7.81E-22 | up |
| Serpinb9 | 2.3744988 | 2.81E-23 | 9.15E-22 | up |
| Pilra | 3.4856346 | 3.19E-23 | 1.04E-21 | up |
| Itga7 | 1.874326 | 3.23E-23 | 1.05E-21 | up |
| Gstm1 | 1.5134674 | 3.42E-23 | 1.10E-21 | up |
| H2-DMa | 2.2448594 | 3.78E-23 | 1.21E-21 | up |
| Traf1 | 2.5724745 | 4.02E-23 | 1.28E-21 | up |
| Lrrc52 | 5.7801649 | 4.75E-23 | 1.51E-21 | up |
| Cd37 | 2.3134416 | 5.40E-23 | 1.71E-21 | up |
| Tnfsf13 | 2.5996602 | 5.93E-23 | 1.87E-21 | up |
| Wipf1 | 1.3514911 | 5.96E-23 | 1.87E-21 | up |
| Cd200r1 | 3.9501246 | 6.07E-23 | 1.90E-21 | up |
| Vat1l | 4.2930296 | 6.27E-23 | 1.95E-21 | up |
| Hmga2-ps1 | 2.4943012 | 6.26E-23 | 1.95E-21 | up |
| Tgm2 | 3.0423021 | 7.01E-23 | 2.17E-21 | up |
| Plekho1 | 1.4590988 | 7.43E-23 | 2.30E-21 | up |
| Car9 | 3.5137042 | 7.58E-23 | 2.34E-21 | up |
| Trpv2 | 2.6555064 | 7.98E-23 | 2.46E-21 | up |
| Prcp | 1.554516 | 8.79E-23 | 2.70E-21 | up |
| Tmem106a | 1.6947033 | 1.00E-22 | 3.07E-21 | up |
| Acot11 | 2.0736544 | 1.08E-22 | 3.30E-21 | up |
| Fads3 | 1.381476 | 1.10E-22 | 3.35E-21 | up |
| Cyba | 1.6951795 | 1.15E-22 | 3.48E-21 | up |
| Dock10 | 2.2812821 | 1.19E-22 | 3.59E-21 | up |
| Cd300ld | 2.4458174 | 1.46E-22 | 4.40E-21 | up |
| Ear1 | 8.6778479 | 1.56E-22 | 4.67E-21 | up |
| Hkdc1 | 4.3630199 | 1.88E-22 | 5.62E-21 | up |
| Clec4a2 | 3.1863282 | 1.96E-22 | 5.87E-21 | up |
| Ccl9 | 2.7611007 | 2.51E-22 | 7.45E-21 | up |
| Evi2b | 2.5738153 | 2.62E-22 | 7.77E-21 | up |
| Ncf4 | 2.0189772 | 2.65E-22 | 7.84E-21 | up |
| St3gal4 | 1.325151 | 2.79E-22 | 8.23E-21 | up |
| Mmp14 | 1.8932848 | 2.87E-22 | 8.45E-21 | up |
| Snx20 | 2.8143416 | 3.08E-22 | 9.00E-21 | up |
| Ly86 | 2.3305388 | 3.62E-22 | 1.05E-20 | up |
| AU022793 | 4.9007691 | 3.86E-22 | 1.12E-20 | up |
| Lum | 2.3898177 | 3.91E-22 | 1.13E-20 | up |
| C5ar1 | 2.5970916 | 4.30E-22 | 1.24E-20 | up |
| Gpsm3 | 2.2784465 | 4.34E-22 | 1.25E-20 | up |
| Fzd1 | 1.3809107 | 4.62E-22 | 1.33E-20 | up |
| Metrnl | 1.6808776 | 7.16E-22 | 2.03E-20 | up |
| Ppp1r14a | 2.4492935 | 7.77E-22 | 2.20E-20 | up |
| Ltc4s | 1.9677315 | 8.40E-22 | 2.37E-20 | up |
| H2-DMb2 | 2.1567925 | 8.72E-22 | 2.46E-20 | up |
| Mrc2 | 1.8112882 | 1.37E-21 | 3.82E-20 | up |
| Nfkb2 | 1.0720991 | 1.44E-21 | 4.01E-20 | up |
| Lsp1 | 1.8317383 | 1.56E-21 | 4.31E-20 | up |
| Dpp7 | 2.0173748 | 1.79E-21 | 4.91E-20 | up |
| Cd36 | 3.2319533 | 1.83E-21 | 5.00E-20 | up |
| Pik3cd | 1.6914719 | 1.89E-21 | 5.16E-20 | up |
| Vcam1 | 2.4268221 | 2.03E-21 | 5.54E-20 | up |
| Gpr20 | 2.5861817 | 2.07E-21 | 5.62E-20 | up |
| Naip5 | 3.1640579 | 2.47E-21 | 6.66E-20 | up |
| Adcy7 | 1.5276351 | 2.46E-21 | 6.66E-20 | up |
| Itgad | 4.4426663 | 2.90E-21 | 7.81E-20 | up |
| Irx3 | 2.2256852 | 2.92E-21 | 7.86E-20 | up |
| Rac2 | 2.5571737 | 3.03E-21 | 8.15E-20 | up |
| Stac2 | 2.8440604 | 3.43E-21 | 9.20E-20 | up |
| Kcnn4 | 2.7510785 | 3.99E-21 | 1.06E-19 | up |
| 1-Mar | 2.424917 | 4.01E-21 | 1.07E-19 | up |
| Kynu | 6.3414434 | 4.13E-21 | 1.10E-19 | up |
| 1810033B17Rik | 5.5970756 | 4.37E-21 | 1.16E-19 | up |
| Zfp36l2 | 1.1409797 | 4.75E-21 | 1.25E-19 | up |
| Ifngr2 | 1.2306994 | 4.99E-21 | 1.31E-19 | up |
| Emp3 | 1.722773 | 5.30E-21 | 1.39E-19 | up |
| Rab31 | 1.5005228 | 5.58E-21 | 1.46E-19 | up |
| 5430435G22Rik | 2.1461255 | 5.66E-21 | 1.48E-19 | up |
| Ccr5 | 2.4993411 | 5.77E-21 | 1.51E-19 | up |
| Sfxn3 | 1.0503337 | 7.86E-21 | 2.04E-19 | up |
| Ramp1 | 3.6278762 | 9.63E-21 | 2.47E-19 | up |
| Mfap2 | 2.7603132 | 9.92E-21 | 2.54E-19 | up |
| Rab3il1 | 1.5846455 | 1.02E-20 | 2.62E-19 | up |
| Scarf2 | 2.1773975 | 1.03E-20 | 2.63E-19 | up |
| Gp49a | 3.3693011 | 1.05E-20 | 2.69E-19 | up |
| Rasa4 | 1.3686349 | 1.09E-20 | 2.77E-19 | up |
| Ear10 | 7.9851459 | 1.10E-20 | 2.79E-19 | up |
| Mamdc2 | 2.8724327 | 1.12E-20 | 2.84E-19 | up |
| Reep2 | 2.0754231 | 1.16E-20 | 2.94E-19 | up |
| Nfkbie | 1.9142016 | 1.22E-20 | 3.07E-19 | up |
| Bin2 | 2.3838673 | 1.31E-20 | 3.30E-19 | up |
| Eid1 | 1.6367697 | 1.34E-20 | 3.38E-19 | up |
| Tlr8 | 2.8825953 | 1.36E-20 | 3.41E-19 | up |
| 5730559C18Rik | 3.2459586 | 1.46E-20 | 3.65E-19 | up |
| Cmtm3 | 1.4340048 | 1.46E-20 | 3.66E-19 | up |
| Sys1 | 1.1443271 | 1.48E-20 | 3.69E-19 | up |
| Clec2d | 1.8309183 | 1.52E-20 | 3.79E-19 | up |
| Apbb1ip | 1.8986496 | 1.55E-20 | 3.85E-19 | up |
| Ftl1 | 1.6078067 | 1.70E-20 | 4.20E-19 | up |
| 2810474O19Rik | 1.0976349 | 1.72E-20 | 4.25E-19 | up |
| Anpep | 2.8287161 | 1.74E-20 | 4.28E-19 | up |
| Ptpn7 | 3.1247536 | 2.02E-20 | 4.94E-19 | up |
| Card11 | 3.8934504 | 2.27E-20 | 5.52E-19 | up |
| Aif1 | 2.21406 | 2.30E-20 | 5.60E-19 | up |
| Fam20c | 1.7061925 | 2.32E-20 | 5.63E-19 | up |
| Galnt6 | 3.7326258 | 2.40E-20 | 5.82E-19 | up |
| Ccl11 | 2.3267921 | 2.51E-20 | 6.06E-19 | up |
| Mrc1 | 1.9193839 | 2.58E-20 | 6.22E-19 | up |
| Cntln | 1.3656818 | 2.60E-20 | 6.26E-19 | up |
| Ccl22 | 6.1627235 | 2.63E-20 | 6.30E-19 | up |
| Mrgpre | 2.0479484 | 2.68E-20 | 6.42E-19 | up |
| Dok2 | 2.7088657 | 4.70E-20 | 1.12E-18 | up |
| Irx5 | 3.6566843 | 4.81E-20 | 1.14E-18 | up |
| Runx2 | 1.9408336 | 6.04E-20 | 1.42E-18 | up |
| Nt5e | 1.9830319 | 6.83E-20 | 1.60E-18 | up |
| Milr1 | 2.4403058 | 7.33E-20 | 1.71E-18 | up |
| Xdh | 2.0171346 | 9.42E-20 | 2.17E-18 | up |
| AB124611 | 2.5063353 | 9.96E-20 | 2.29E-18 | up |
| H2-Ab1 | 2.5489749 | 1.02E-19 | 2.34E-18 | up |
| Gpr35 | 3.3879607 | 1.12E-19 | 2.56E-18 | up |
| Lgr6 | 4.5198006 | 1.15E-19 | 2.61E-18 | up |
| Fmnl1 | 1.7752862 | 1.29E-19 | 2.93E-18 | up |
| Lair1 | 1.9779011 | 1.31E-19 | 2.97E-18 | up |
| Ppfia4 | 1.7706395 | 1.31E-19 | 2.97E-18 | up |
| Serinc2 | 2.5731257 | 1.42E-19 | 3.22E-18 | up |
| Cmbl | 2.8691075 | 1.54E-19 | 3.48E-18 | up |
| Plau | 2.4720637 | 1.56E-19 | 3.52E-18 | up |
| Gpbar1 | 5.7600885 | 1.61E-19 | 3.62E-18 | up |
| Nhp2 | 1.1309685 | 1.79E-19 | 4.01E-18 | up |
| Gna15 | 2.4176712 | 1.79E-19 | 4.02E-18 | up |
| Crtac1 | 5.0845199 | 1.86E-19 | 4.16E-18 | up |
| Rbm3 | 1.1095363 | 2.00E-19 | 4.44E-18 | up |
| Aqp7 | 4.0964492 | 2.01E-19 | 4.47E-18 | up |
| Pmaip1 | 2.607711 | 2.07E-19 | 4.59E-18 | up |
| Sash3 | 2.9154612 | 2.16E-19 | 4.78E-18 | up |
| Mical1 | 1.338446 | 2.22E-19 | 4.89E-18 | up |
| Abcd2 | 2.974754 | 2.30E-19 | 5.06E-18 | up |
| Lrmp | 2.6016582 | 2.74E-19 | 6.01E-18 | up |
| Prkar2b | 2.326399 | 2.78E-19 | 6.06E-18 | up |
| Cfp | 2.1680845 | 2.79E-19 | 6.06E-18 | up |
| Nr1h3 | 1.3211814 | 2.79E-19 | 6.06E-18 | up |
| Loxl1 | 1.8410402 | 2.98E-19 | 6.48E-18 | up |
| Tgfbr2 | 1.4697438 | 3.11E-19 | 6.74E-18 | up |
| Pde1c | 2.2679384 | 3.49E-19 | 7.55E-18 | up |
| Irs1 | 1.6797613 | 3.53E-19 | 7.61E-18 | up |
| Dnase1l1 | 1.1068359 | 3.82E-19 | 8.23E-18 | up |
| Slc9a9 | 2.2367597 | 4.16E-19 | 8.94E-18 | up |
| Was | 2.3646448 | 4.45E-19 | 9.53E-18 | up |
| Mknk2 | 1.2843069 | 4.99E-19 | 1.07E-17 | up |
| Pmp2 | 5.1993739 | 5.10E-19 | 1.09E-17 | up |
| Fes | 1.3667899 | 5.43E-19 | 1.15E-17 | up |
| C1ra | 2.027624 | 5.49E-19 | 1.17E-17 | up |
| Lrrc25 | 2.3101734 | 5.68E-19 | 1.20E-17 | up |
| Pex5l | 5.3751239 | 6.39E-19 | 1.35E-17 | up |
| H2-Aa | 2.495438 | 6.49E-19 | 1.37E-17 | up |
| Tnfrsf11a | 2.4138062 | 7.24E-19 | 1.52E-17 | up |
| Bcl2a1b | 2.3364654 | 8.38E-19 | 1.75E-17 | up |
| Spp1 | 3.4016058 | 8.90E-19 | 1.86E-17 | up |
| Rhobtb1 | 1.3543439 | 9.55E-19 | 1.99E-17 | up |
| Amdhd2 | 1.1878906 | 9.77E-19 | 2.03E-17 | up |
| Cacnb3 | 1.395416 | 9.82E-19 | 2.04E-17 | up |
| Hs3st1 | 2.2916272 | 1.01E-18 | 2.10E-17 | up |
| Mxra7 | 1.4780529 | 1.05E-18 | 2.17E-17 | up |
| Tlr7 | 2.4002173 | 1.06E-18 | 2.19E-17 | up |
| Cd302 | 1.7336533 | 1.25E-18 | 2.57E-17 | up |
| Rcn1 | 1.6340273 | 1.35E-18 | 2.75E-17 | up |
| Lxn | 2.4691011 | 1.48E-18 | 3.02E-17 | up |
| Eno2 | 1.6590402 | 1.54E-18 | 3.13E-17 | up |
| St6galnac4 | 1.565064 | 1.65E-18 | 3.35E-17 | up |
| Slc35e4 | 1.329614 | 1.91E-18 | 3.85E-17 | up |
| Tap2 | 1.1602967 | 2.22E-18 | 4.46E-17 | up |
| Prkar1b | 1.931055 | 2.29E-18 | 4.58E-17 | up |
| 1190002N15Rik | 1.378875 | 2.34E-18 | 4.68E-17 | up |
| Tmsb10 | 1.4799376 | 2.36E-18 | 4.72E-17 | up |
| Il3ra | 1.6794499 | 2.49E-18 | 4.97E-17 | up |
| Aldh1b1 | 4.510953 | 2.92E-18 | 5.78E-17 | up |
| Siglec5 | 5.5372452 | 2.98E-18 | 5.90E-17 | up |
| Ttyh2 | 1.8211957 | 3.15E-18 | 6.21E-17 | up |
| Lcp2 | 2.4554596 | 3.51E-18 | 6.89E-17 | up |
| Serpine2 | 1.3762412 | 3.97E-18 | 7.74E-17 | up |
| Jak3 | 1.3631153 | 4.04E-18 | 7.86E-17 | up |
| Plcb2 | 2.7680012 | 4.05E-18 | 7.87E-17 | up |
| Gsn | 1.8986897 | 4.53E-18 | 8.77E-17 | up |
| Sftpd | 4.0309543 | 4.72E-18 | 9.10E-17 | up |
| Mmp17 | 2.2768976 | 4.77E-18 | 9.17E-17 | up |
| Adc | 1.81413 | 4.94E-18 | 9.49E-17 | up |
| Msr1 | 2.9073183 | 6.01E-18 | 1.15E-16 | up |
| Ticam2 | 2.7661174 | 6.79E-18 | 1.29E-16 | up |
| Slco2b1 | 1.4931777 | 7.10E-18 | 1.34E-16 | up |
| Sirpb1a | 4.99278 | 7.41E-18 | 1.40E-16 | up |
| Tfec | 4.0212232 | 7.48E-18 | 1.41E-16 | up |
| Pdgfrl | 2.44207 | 8.62E-18 | 1.62E-16 | up |
| Lbh | 1.1207372 | 8.80E-18 | 1.65E-16 | up |
| Agap1 | 1.2168984 | 8.85E-18 | 1.66E-16 | up |
| Sh3bp2 | 1.4868624 | 8.95E-18 | 1.68E-16 | up |
| AU018091 | 4.5500429 | 9.09E-18 | 1.70E-16 | up |
| Inpp5d | 1.4135413 | 9.16E-18 | 1.71E-16 | up |
| 1700017B05Rik | 1.4778015 | 1.02E-17 | 1.89E-16 | up |
| Tnfaip2 | 1.7146281 | 1.07E-17 | 1.99E-16 | up |
| Atp8b4 | 3.8626619 | 1.16E-17 | 2.15E-16 | up |
| Ptafr | 2.6494032 | 1.30E-17 | 2.40E-16 | up |
| Vwa7 | 2.6494797 | 1.43E-17 | 2.61E-16 | up |
| Lipk | 4.6259736 | 1.83E-17 | 3.33E-16 | up |
| Ifi27l2a | 3.5650096 | 2.19E-17 | 3.97E-16 | up |
| Plekha6 | 1.889933 | 2.60E-17 | 4.68E-16 | up |
| C3 | 2.6622207 | 2.65E-17 | 4.76E-16 | up |
| Obsl1 | 2.099875 | 2.89E-17 | 5.17E-16 | up |
| Msra | 1.2209182 | 3.02E-17 | 5.40E-16 | up |
| Kcnd3 | 3.9532794 | 3.13E-17 | 5.59E-16 | up |
| Lilra6 | 3.2035658 | 3.16E-17 | 5.63E-16 | up |
| Pde7a | 1.19339 | 3.41E-17 | 6.04E-16 | up |
| St6galnac6 | 1.829437 | 3.56E-17 | 6.30E-16 | up |
| Fmo1 | 2.2589318 | 3.75E-17 | 6.61E-16 | up |
| Cyp4b1 | 2.7732746 | 3.78E-17 | 6.64E-16 | up |
| Mafb | 1.9709187 | 3.78E-17 | 6.64E-16 | up |
| Ikzf1 | 2.3381101 | 3.79E-17 | 6.66E-16 | up |
| Cldn5 | 2.774606 | 3.89E-17 | 6.83E-16 | up |
| S100a14 | 3.032394 | 4.00E-17 | 7.00E-16 | up |
| Fcgr4 | 3.2679667 | 4.38E-17 | 7.66E-16 | up |
| Lmo2 | 1.5748159 | 5.06E-17 | 8.79E-16 | up |
| Ncf1 | 1.6982362 | 5.30E-17 | 9.18E-16 | up |
| Siglec1 | 2.4249656 | 5.33E-17 | 9.22E-16 | up |
| Pamr1 | 1.7961367 | 5.39E-17 | 9.32E-16 | up |
| Themis2 | 2.1061813 | 5.48E-17 | 9.46E-16 | up |
| Ang4 | 4.7347257 | 5.63E-17 | 9.69E-16 | up |
| Myo5a | 1.2836056 | 5.71E-17 | 9.83E-16 | up |
| C1s | 1.7286066 | 5.94E-17 | 1.02E-15 | up |
| Serping1 | 1.7229016 | 7.20E-17 | 1.23E-15 | up |
| Fbxo32 | 1.6301869 | 7.26E-17 | 1.24E-15 | up |
| Ndnf | 2.9856041 | 7.28E-17 | 1.24E-15 | up |
| Cpne2 | 1.8895069 | 7.40E-17 | 1.26E-15 | up |
| Bcap29 | 1.2213021 | 7.41E-17 | 1.26E-15 | up |
| Apoc2 | 3.5149155 | 7.75E-17 | 1.31E-15 | up |
| Map4k1 | 2.3278421 | 7.81E-17 | 1.32E-15 | up |
| Cd74 | 2.1930554 | 8.92E-17 | 1.50E-15 | up |
| Fam46b | 2.1087489 | 9.89E-17 | 1.66E-15 | up |
| Thbs2 | 2.0587846 | 1.03E-16 | 1.72E-15 | up |
| Sh3kbp1 | 1.5637349 | 1.07E-16 | 1.79E-15 | up |
| Pla2g16 | 1.0867947 | 1.16E-16 | 1.95E-15 | up |
| Sepp1 | 1.6902763 | 1.17E-16 | 1.95E-15 | up |
| Kcnab1 | 3.2945646 | 1.21E-16 | 2.01E-15 | up |
| Athl1 | 1.3400225 | 1.25E-16 | 2.08E-15 | up |
| Tmem119 | 1.6513421 | 1.26E-16 | 2.08E-15 | up |
| Ephb3 | 1.4214202 | 1.26E-16 | 2.09E-15 | up |
| Trp53inp1 | 1.1067743 | 1.28E-16 | 2.11E-15 | up |
| Itgam | 1.9067294 | 1.29E-16 | 2.13E-15 | up |
| Cyp1b1 | 1.9112191 | 1.35E-16 | 2.23E-15 | up |
| Wfdc17 | 1.8971716 | 1.39E-16 | 2.29E-15 | up |
| Negr1 | 3.5145374 | 1.42E-16 | 2.32E-15 | up |
| Ifi202b | 1.7062564 | 1.47E-16 | 2.41E-15 | up |
| Rap1gap2 | 2.6825561 | 1.61E-16 | 2.63E-15 | up |
| Fcgrt | 1.5834575 | 1.66E-16 | 2.70E-15 | up |
| Fam183b | 4.7065727 | 1.68E-16 | 2.73E-15 | up |
| Zfp385a | 1.3881527 | 1.69E-16 | 2.73E-15 | up |
| 1810011H11Rik | 1.8036286 | 1.89E-16 | 3.06E-15 | up |
| Wfikkn2 | 2.3421108 | 1.89E-16 | 3.06E-15 | up |
| Gm13476 | 2.6798945 | 2.07E-16 | 3.34E-15 | up |
| Fabp7 | 6.4616135 | 2.11E-16 | 3.40E-15 | up |
| Phlda3 | 1.0461984 | 2.29E-16 | 3.68E-15 | up |
| Foxc1 | 1.8425207 | 2.30E-16 | 3.69E-15 | up |
| Irs4 | 4.4479927 | 2.45E-16 | 3.92E-15 | up |
| Acox2 | 2.8897138 | 2.79E-16 | 4.45E-15 | up |
| Ddit4l | 1.5301422 | 2.90E-16 | 4.60E-15 | up |
| Arhgef6 | 1.4593787 | 3.09E-16 | 4.90E-15 | up |
| Dhrs1 | 1.0156642 | 3.43E-16 | 5.43E-15 | up |
| Sytl2 | 2.6606661 | 3.53E-16 | 5.57E-15 | up |
| Prl2c5 | 8.3297008 | 3.58E-16 | 5.64E-15 | up |
| Clip3 | 1.5508439 | 3.63E-16 | 5.70E-15 | up |
| Zfp423 | 1.6977323 | 3.67E-16 | 5.76E-15 | up |
| Dhrs9 | 3.8595276 | 3.70E-16 | 5.79E-15 | up |
| Sox10 | 4.0587848 | 3.74E-16 | 5.85E-15 | up |
| Rab27a | 1.6901537 | 3.86E-16 | 6.04E-15 | up |
| Tnip3 | 4.0476395 | 4.10E-16 | 6.37E-15 | up |
| Dio3os | 2.3267114 | 4.39E-16 | 6.82E-15 | up |
| Serpinf1 | 1.8490139 | 4.42E-16 | 6.85E-15 | up |
| Pik3cg | 1.9491172 | 4.92E-16 | 7.60E-15 | up |
| Rarres1 | 3.9127281 | 5.33E-16 | 8.23E-15 | up |
| Anxa8 | 2.1318685 | 5.58E-16 | 8.60E-15 | up |
| Rspo3 | 3.7357565 | 5.83E-16 | 8.96E-15 | up |
| Pde2a | 1.3362931 | 5.93E-16 | 9.11E-15 | up |
| Gstm2 | 1.8072817 | 6.47E-16 | 9.90E-15 | up |
| Scara5 | 1.2198169 | 6.63E-16 | 1.01E-14 | up |
| P2rx7 | 1.6347406 | 6.76E-16 | 1.03E-14 | up |
| Chst12 | 1.3526875 | 7.30E-16 | 1.11E-14 | up |
| Abi3bp | 1.8803434 | 7.56E-16 | 1.15E-14 | up |
| Pion | 2.659437 | 7.99E-16 | 1.21E-14 | up |
| Sele | 3.3531944 | 8.34E-16 | 1.26E-14 | up |
| Arhgap25 | 1.692694 | 9.17E-16 | 1.38E-14 | up |
| Il17rc | 1.3467306 | 9.61E-16 | 1.45E-14 | up |
| Fmo2 | 2.0814325 | 9.92E-16 | 1.49E-14 | up |
| Mctp1 | 2.8596161 | 1.02E-15 | 1.53E-14 | up |
| Osr1 | 2.0006456 | 1.03E-15 | 1.55E-14 | up |
| Hck | 1.6844722 | 1.07E-15 | 1.60E-14 | up |
| Fbln1 | 2.3653657 | 1.09E-15 | 1.63E-14 | up |
| Aim2 | 2.5515992 | 1.26E-15 | 1.87E-14 | up |
| Ccr1 | 2.1769186 | 1.33E-15 | 1.98E-14 | up |
| Dcn | 2.0284973 | 1.44E-15 | 2.14E-14 | up |
| Cplx2 | 1.6443915 | 1.51E-15 | 2.24E-14 | up |
| Ccbe1 | 3.6288369 | 1.80E-15 | 2.65E-14 | up |
| Plin2 | 2.2854243 | 1.81E-15 | 2.66E-14 | up |
| Dpep1 | 1.7895632 | 2.03E-15 | 2.97E-14 | up |
| Hsd3b7 | 1.2702418 | 2.07E-15 | 3.03E-14 | up |
| Rasa3 | 1.323156 | 2.09E-15 | 3.06E-14 | up |
| Sh3rf3 | 1.7250595 | 2.10E-15 | 3.07E-14 | up |
| Matn2 | 2.120692 | 2.20E-15 | 3.21E-14 | up |
| Tm6sf1 | 1.6719393 | 2.30E-15 | 3.34E-14 | up |
| Rarres2 | 1.8694618 | 2.33E-15 | 3.38E-14 | up |
| Gpr34 | 1.7366424 | 2.41E-15 | 3.49E-14 | up |
| Timp2 | 1.4495827 | 2.57E-15 | 3.73E-14 | up |
| Myo7a | 2.4219594 | 2.78E-15 | 4.02E-14 | up |
| Ctsh | 1.5686953 | 2.84E-15 | 4.10E-14 | up |
| Cyb5 | 1.0606162 | 2.96E-15 | 4.27E-14 | up |
| Chi3l1 | 3.2064981 | 3.00E-15 | 4.32E-14 | up |
| Myo1g | 2.3147761 | 3.01E-15 | 4.33E-14 | up |
| Arhgap19 | 1.3995185 | 3.06E-15 | 4.40E-14 | up |
| Homer2 | 1.7755289 | 3.20E-15 | 4.58E-14 | up |
| Cp | 1.3702708 | 3.58E-15 | 5.11E-14 | up |
| Mgst1 | 1.4476002 | 3.70E-15 | 5.27E-14 | up |
| 1810037I17Rik | 1.2459926 | 3.80E-15 | 5.40E-14 | up |
| Naip2 | 1.910265 | 4.00E-15 | 5.67E-14 | up |
| Akna | 1.5058702 | 4.15E-15 | 5.86E-14 | up |
| Cygb | 1.4609982 | 4.58E-15 | 6.48E-14 | up |
| Spsb1 | 1.6198527 | 4.94E-15 | 6.96E-14 | up |
| Lrg1 | 2.0789917 | 5.20E-15 | 7.31E-14 | up |
| Def6 | 1.7259327 | 5.34E-15 | 7.50E-14 | up |
| Mfap4 | 2.2661573 | 5.48E-15 | 7.69E-14 | up |
| Lilra5 | 1.9623354 | 5.56E-15 | 7.80E-14 | up |
| Pid1 | 1.4127632 | 5.84E-15 | 8.17E-14 | up |
| Ccl17 | 4.0068948 | 6.19E-15 | 8.63E-14 | up |
| Ada | 2.4658189 | 6.40E-15 | 8.91E-14 | up |
| Efemp2 | 1.081875 | 6.47E-15 | 8.99E-14 | up |
| Slc22a18 | 1.9525752 | 6.67E-15 | 9.26E-14 | up |
| AI413582 | 1.4798893 | 6.80E-15 | 9.43E-14 | up |
| Acsl4 | 1.4652364 | 6.89E-15 | 9.54E-14 | up |
| Epsti1 | 2.1616003 | 7.58E-15 | 1.05E-13 | up |
| Ccl6 | 2.3400967 | 7.81E-15 | 1.08E-13 | up |
| Cd33 | 1.8611084 | 8.53E-15 | 1.17E-13 | up |
| Lpxn | 2.0402113 | 8.64E-15 | 1.19E-13 | up |
| Spata20 | 4.219744 | 9.26E-15 | 1.27E-13 | up |
| Scn1b | 1.7533184 | 9.69E-15 | 1.32E-13 | up |
| Vim | 1.6678733 | 9.79E-15 | 1.34E-13 | up |
| Fam19a5 | 1.8396901 | 1.03E-14 | 1.40E-13 | up |
| Plin4 | 2.1673968 | 1.04E-14 | 1.42E-13 | up |
| Postn | 1.7909843 | 1.12E-14 | 1.52E-13 | up |
| Vill | 2.9334857 | 1.15E-14 | 1.55E-13 | up |
| Pam | 1.301176 | 1.21E-14 | 1.63E-13 | up |
| Kirrel | 1.4771544 | 1.31E-14 | 1.76E-13 | up |
| Clec4a3 | 2.2688533 | 1.33E-14 | 1.79E-13 | up |
| Fxyd3 | 3.6659068 | 1.34E-14 | 1.81E-13 | up |
| Nox4 | 1.9277874 | 1.45E-14 | 1.95E-13 | up |
| Eda2r | 1.4716595 | 1.49E-14 | 2.00E-13 | up |
| Lpcat4 | 1.5885692 | 1.59E-14 | 2.12E-13 | up |
| Txnip | 1.1426866 | 1.72E-14 | 2.30E-13 | up |
| Ogn | 2.09287 | 1.73E-14 | 2.30E-13 | up |
| Unc5c | 2.2010591 | 1.77E-14 | 2.36E-13 | up |
| Rasal3 | 2.5853947 | 1.86E-14 | 2.47E-13 | up |
| Casp8 | 1.0021108 | 1.87E-14 | 2.47E-13 | up |
| Cdkn2a | 2.157143 | 1.89E-14 | 2.50E-13 | up |
| Hcls1 | 1.3662346 | 2.09E-14 | 2.76E-13 | up |
| Cnr2 | 2.0346396 | 2.15E-14 | 2.84E-13 | up |
| Aff3 | 2.238917 | 2.18E-14 | 2.87E-13 | up |
| Anxa2 | 1.5419851 | 2.53E-14 | 3.33E-13 | up |
| Bak1 | 1.0301133 | 2.67E-14 | 3.51E-13 | up |
| Nqo1 | 1.836444 | 2.72E-14 | 3.57E-13 | up |
| Man1a | 1.6557357 | 2.78E-14 | 3.64E-13 | up |
| Itgal | 2.2781732 | 2.82E-14 | 3.70E-13 | up |
| Clec3b | 1.9409125 | 2.89E-14 | 3.78E-13 | up |
| Dock11 | 1.8209766 | 2.94E-14 | 3.84E-13 | up |
| Cln6 | 1.0252184 | 3.01E-14 | 3.93E-13 | up |
| A530013C23Rik | 3.7407342 | 3.09E-14 | 4.03E-13 | up |
| Abca8a | 1.7481407 | 3.14E-14 | 4.09E-13 | up |
| Aatk | 1.3660829 | 3.22E-14 | 4.19E-13 | up |
| Gngt2 | 1.5072237 | 3.58E-14 | 4.65E-13 | up |
| Cyp27a1 | 2.0774383 | 3.65E-14 | 4.73E-13 | up |
| Serpina3h | 2.5714255 | 3.68E-14 | 4.76E-13 | up |
| Cd209a | 2.5845094 | 3.70E-14 | 4.79E-13 | up |
| Psmb8 | 1.5765107 | 4.25E-14 | 5.49E-13 | up |
| Akr1b8 | 1.6485896 | 4.41E-14 | 5.68E-13 | up |
| B4galt2 | 1.3115129 | 4.46E-14 | 5.74E-13 | up |
| Zfp462 | 1.1372712 | 4.47E-14 | 5.74E-13 | up |
| Ccdc80 | 1.5659091 | 4.64E-14 | 5.95E-13 | up |
| Clu | 1.9624325 | 4.70E-14 | 6.02E-13 | up |
| D1Ertd622e | 1.0387336 | 4.72E-14 | 6.04E-13 | up |
| Ppp1r18 | 1.1525295 | 4.82E-14 | 6.15E-13 | up |
| Gusb | 1.0808865 | 4.83E-14 | 6.16E-13 | up |
| Npffr2 | 2.9546923 | 5.49E-14 | 6.95E-13 | up |
| Plac1 | 4.0396975 | 5.61E-14 | 7.10E-13 | up |
| Il6ra | 2.068283 | 5.62E-14 | 7.11E-13 | up |
| Gm5150 | 3.6535099 | 5.64E-14 | 7.13E-13 | up |
| Phyhd1 | 1.4917559 | 5.67E-14 | 7.15E-13 | up |
| Lancl3 | 2.629679 | 5.70E-14 | 7.20E-13 | up |
| Rassf2 | 1.679204 | 5.98E-14 | 7.51E-13 | up |
| Coro2a | 2.3486989 | 6.03E-14 | 7.57E-13 | up |
| Fam3c | 1.277722 | 6.15E-14 | 7.72E-13 | up |
| Jdp2 | 1.1579634 | 6.22E-14 | 7.79E-13 | up |
| Tmem109 | 1.0277438 | 6.56E-14 | 8.19E-13 | up |
| Evc2 | 1.6098697 | 6.60E-14 | 8.23E-13 | up |
| Olfml3 | 1.6000395 | 7.08E-14 | 8.80E-13 | up |
| Spr | 1.044659 | 7.26E-14 | 9.02E-13 | up |
| Plekhf1 | 1.4189225 | 7.31E-14 | 9.08E-13 | up |
| Rrad | 1.9026496 | 7.83E-14 | 9.71E-13 | up |
| Slamf7 | 4.1571706 | 8.59E-14 | 1.06E-12 | up |
| Gsdmd | 1.5100907 | 8.96E-14 | 1.11E-12 | up |
| Myl9 | 1.3176507 | 9.19E-14 | 1.13E-12 | up |
| Lrrc39 | 2.4685145 | 9.22E-14 | 1.14E-12 | up |
| Srgap1 | 1.7746311 | 1.05E-13 | 1.28E-12 | up |
| Tmem154 | 2.2674472 | 1.05E-13 | 1.29E-12 | up |
| Tifa | 1.48904 | 1.05E-13 | 1.29E-12 | up |
| Lst1 | 2.2036515 | 1.10E-13 | 1.34E-12 | up |
| Prkcb | 2.2963344 | 1.14E-13 | 1.39E-12 | up |
| Tpsab1 | 3.902927 | 1.16E-13 | 1.41E-12 | up |
| Mdfic | 1.0472018 | 1.20E-13 | 1.46E-12 | up |
| Wdfy4 | 1.8472613 | 1.28E-13 | 1.55E-12 | up |
| Cacna2d1 | 2.5165664 | 1.34E-13 | 1.63E-12 | up |
| Samd4 | 1.4294333 | 1.41E-13 | 1.70E-12 | up |
| Gmfg | 1.584377 | 1.43E-13 | 1.73E-12 | up |
| Irak3 | 1.5759221 | 1.45E-13 | 1.75E-12 | up |
| Pira6 | 3.845042 | 1.48E-13 | 1.78E-12 | up |
| Selp | 2.7306059 | 1.48E-13 | 1.78E-12 | up |
| 9130017N09Rik | 3.0344596 | 1.52E-13 | 1.83E-12 | up |
| Cdkn1a | 1.1465014 | 1.56E-13 | 1.87E-12 | up |
| Lrp8 | 2.265381 | 1.69E-13 | 2.02E-12 | up |
| Abcg3 | 3.0327871 | 1.83E-13 | 2.19E-12 | up |
| C2 | 2.1827465 | 1.98E-13 | 2.36E-12 | up |
| Mpp6 | 1.0105796 | 2.12E-13 | 2.53E-12 | up |
| Glipr2 | 1.569562 | 2.17E-13 | 2.58E-12 | up |
| Dse | 1.5981388 | 2.19E-13 | 2.61E-12 | up |
| Hmha1 | 1.3274836 | 2.27E-13 | 2.70E-12 | up |
| Ctsb | 1.1782129 | 2.32E-13 | 2.75E-12 | up |
| B3gnt8 | 1.8461279 | 2.50E-13 | 2.96E-12 | up |
| Mmp23 | 1.4930525 | 2.51E-13 | 2.97E-12 | up |
| Slc6a7 | 3.7722301 | 2.54E-13 | 3.00E-12 | up |
| Rhbdl3 | 1.8359881 | 2.63E-13 | 3.11E-12 | up |
| Ciita | 1.6732718 | 2.65E-13 | 3.13E-12 | up |
| Mmp8 | 4.8254254 | 2.85E-13 | 3.36E-12 | up |
| Vps37d | 2.0756691 | 2.86E-13 | 3.37E-12 | up |
| Nat8l | 3.5371379 | 3.46E-13 | 4.03E-12 | up |
| Lgals1 | 1.2517356 | 3.48E-13 | 4.06E-12 | up |
| Hoxb8 | 3.6919698 | 3.53E-13 | 4.11E-12 | up |
| BC048679 | 4.1282856 | 3.59E-13 | 4.18E-12 | up |
| Zcchc24 | 1.2643941 | 3.67E-13 | 4.26E-12 | up |
| Cdkn1c | 2.9667045 | 3.89E-13 | 4.48E-12 | up |
| Klrb1b | 3.1330398 | 4.09E-13 | 4.71E-12 | up |
| Cidec | 3.695218 | 4.12E-13 | 4.74E-12 | up |
| Cd86 | 1.8337621 | 4.23E-13 | 4.86E-12 | up |
| Serpinb8 | 1.7889701 | 4.58E-13 | 5.25E-12 | up |
| Ass1 | 1.6236016 | 4.85E-13 | 5.55E-12 | up |
| Dpt | 1.8496335 | 4.97E-13 | 5.67E-12 | up |
| Tcea3 | 2.5006869 | 5.27E-13 | 6.00E-12 | up |
| Pyhin1 | 2.6560536 | 5.34E-13 | 6.07E-12 | up |
| Cyp2e1 | 4.0067307 | 5.39E-13 | 6.12E-12 | up |
| Slamf8 | 2.6358309 | 5.61E-13 | 6.35E-12 | up |
| Slc2a9 | 1.9890956 | 5.76E-13 | 6.52E-12 | up |
| Gng8 | 2.815458 | 5.77E-13 | 6.52E-12 | up |
| Scp2 | 1.0833817 | 6.00E-13 | 6.76E-12 | up |
| Mcoln2 | 2.6307751 | 6.10E-13 | 6.86E-12 | up |
| Casp1 | 1.888631 | 6.14E-13 | 6.90E-12 | up |
| Car3 | 3.7339965 | 6.29E-13 | 7.06E-12 | up |
| Retnla | 2.8641895 | 6.49E-13 | 7.26E-12 | up |
| Tnfaip3 | 1.270272 | 6.65E-13 | 7.44E-12 | up |
| Ptges | 1.3249814 | 7.18E-13 | 7.99E-12 | up |
| Cxcl11 | 2.7218364 | 7.25E-13 | 8.05E-12 | up |
| Krt79 | 4.1653027 | 7.48E-13 | 8.30E-12 | up |
| Nfatc2 | 1.5946805 | 7.85E-13 | 8.70E-12 | up |
| BC064078 | 2.1659685 | 8.52E-13 | 9.42E-12 | up |
| Rgs1 | 3.2549125 | 9.06E-13 | 9.99E-12 | up |
| Casp4 | 1.7892377 | 9.45E-13 | 1.04E-11 | up |
| P2ry14 | 1.7988512 | 9.68E-13 | 1.07E-11 | up |
| Pla1a | 1.3321875 | 9.80E-13 | 1.08E-11 | up |
| Afp | 3.782103 | 1.03E-12 | 1.13E-11 | up |
| Slpi | 5.1810606 | 1.06E-12 | 1.16E-11 | up |
| Arhgap15 | 2.3826095 | 1.12E-12 | 1.23E-11 | up |
| Ttc39a | 1.2174121 | 1.14E-12 | 1.25E-11 | up |
| Trerf1 | 2.1051946 | 1.14E-12 | 1.25E-11 | up |
| Cadps | 3.7638521 | 1.19E-12 | 1.30E-11 | up |
| Dlg4 | 1.3561278 | 1.20E-12 | 1.31E-11 | up |
| 6330416G13Rik | 1.2261215 | 1.20E-12 | 1.31E-11 | up |
| Emilin1 | 1.2818929 | 1.23E-12 | 1.34E-11 | up |
| AI607873 | 1.4609454 | 1.24E-12 | 1.35E-11 | up |
| Serpina3g | 2.8129283 | 1.25E-12 | 1.36E-11 | up |
| Pla2g2d | 2.7983964 | 1.25E-12 | 1.36E-11 | up |
| B3galt2 | 3.5267858 | 1.27E-12 | 1.38E-11 | up |
| Cxcr6 | 3.0447174 | 1.30E-12 | 1.41E-11 | up |
| Doc2b | 3.1596092 | 1.32E-12 | 1.42E-11 | up |
| Car2 | 1.6242067 | 1.34E-12 | 1.45E-11 | up |
| Trim47 | 1.1452897 | 1.36E-12 | 1.47E-11 | up |
| H2-Q4 | 1.2581968 | 1.39E-12 | 1.50E-11 | up |
| Pstpip1 | 2.4462938 | 1.47E-12 | 1.57E-11 | up |
| Abcd4 | 1.2251227 | 1.58E-12 | 1.69E-11 | up |
| Gpr132 | 3.71156 | 1.64E-12 | 1.74E-11 | up |
| Orm2 | 4.5900214 | 1.68E-12 | 1.79E-11 | up |
| Scimp | 2.2419778 | 1.71E-12 | 1.82E-11 | up |
| Nradd | 1.5783193 | 1.84E-12 | 1.95E-11 | up |
| Crispld2 | 1.2410736 | 1.85E-12 | 1.96E-11 | up |
| Lix1l | 1.0368154 | 2.02E-12 | 2.13E-11 | up |
| Scd1 | 4.2880403 | 2.10E-12 | 2.21E-11 | up |
| Col6a3 | 1.0832064 | 2.32E-12 | 2.44E-11 | up |
| Gja1 | 1.2371011 | 2.34E-12 | 2.46E-11 | up |
| Dact3 | 1.9421817 | 2.43E-12 | 2.56E-11 | up |
| Aldh3b1 | 1.5611749 | 2.56E-12 | 2.69E-11 | up |
| Kcnj10 | 1.7430825 | 2.62E-12 | 2.74E-11 | up |
| Mgst3 | 1.3009853 | 2.74E-12 | 2.85E-11 | up |
| Sla | 1.5209889 | 2.76E-12 | 2.87E-11 | up |
| Axin2 | 1.7652004 | 2.78E-12 | 2.88E-11 | up |
| Ugcg | 1.1748011 | 2.78E-12 | 2.89E-11 | up |
| Tlr9 | 2.3234485 | 3.17E-12 | 3.28E-11 | up |
| Il1f6 | 4.2995106 | 3.18E-12 | 3.28E-11 | up |
| Rcn3 | 1.3890973 | 3.29E-12 | 3.39E-11 | up |
| Ephb6 | 2.4721767 | 3.33E-12 | 3.43E-11 | up |
| Selm | 1.4437024 | 3.37E-12 | 3.47E-11 | up |
| Cidea | 4.6883895 | 3.41E-12 | 3.51E-11 | up |
| Dnase2a | 1.0738644 | 3.71E-12 | 3.80E-11 | up |
| Arl4c | 1.3426412 | 3.92E-12 | 4.01E-11 | up |
| Ehbp1l1 | 1.2904097 | 3.95E-12 | 4.03E-11 | up |
| Plcd3 | 1.4093857 | 4.10E-12 | 4.19E-11 | up |
| Pvt1 | 1.9021818 | 4.60E-12 | 4.68E-11 | up |
| Olfml1 | 2.2654614 | 4.64E-12 | 4.72E-11 | up |
| Hmga2 | 1.566323 | 4.85E-12 | 4.92E-11 | up |
| Gbp2 | 1.4980742 | 4.86E-12 | 4.93E-11 | up |
| Efemp1 | 1.5900858 | 5.00E-12 | 5.06E-11 | up |
| Mgp | 1.7897074 | 5.07E-12 | 5.13E-11 | up |
| Fyb | 1.90599 | 5.53E-12 | 5.58E-11 | up |
| S100a6 | 1.7602177 | 5.56E-12 | 5.61E-11 | up |
| Gpr176 | 2.069672 | 5.57E-12 | 5.62E-11 | up |
| Tpcn2 | 1.2357688 | 5.60E-12 | 5.64E-11 | up |
| Crlf2 | 1.5016789 | 5.62E-12 | 5.66E-11 | up |
| Padi1 | 3.1987791 | 5.69E-12 | 5.72E-11 | up |
| Nbl1 | 1.4060162 | 5.73E-12 | 5.75E-11 | up |
| Csf1 | 1.4394133 | 5.81E-12 | 5.83E-11 | up |
| A630001G21Rik | 2.0110988 | 5.84E-12 | 5.86E-11 | up |
| Susd5 | 3.0429713 | 5.91E-12 | 5.92E-11 | up |
| Cpa4 | 2.929847 | 5.99E-12 | 6.00E-11 | up |
| Fkbp1b | 1.8476028 | 6.01E-12 | 6.01E-11 | up |
| Adamtsl4 | 1.512584 | 6.03E-12 | 6.03E-11 | up |
| 2310069B03Rik | 4.883735 | 6.10E-12 | 6.09E-11 | up |
| Cela1 | 2.4229022 | 6.83E-12 | 6.80E-11 | up |
| H2-Eb1 | 2.011771 | 6.91E-12 | 6.87E-11 | up |
| Bcl11a | 3.2917609 | 7.02E-12 | 6.98E-11 | up |
| Tspan32 | 2.4055665 | 7.16E-12 | 7.12E-11 | up |
| Twist1 | 2.0229704 | 7.23E-12 | 7.18E-11 | up |
| Aspa | 2.1966976 | 7.25E-12 | 7.20E-11 | up |
| Ifi30 | 1.0971041 | 7.27E-12 | 7.21E-11 | up |
| Amotl1 | 1.3074653 | 7.37E-12 | 7.31E-11 | up |
| Igf2bp2 | 1.052004 | 8.05E-12 | 7.95E-11 | up |
| Ebi3 | 2.249045 | 8.11E-12 | 8.00E-11 | up |
| Fut9 | 2.7369475 | 8.18E-12 | 8.06E-11 | up |
| P2ry13 | 1.6970558 | 8.55E-12 | 8.42E-11 | up |
| Col1a2 | 1.6876092 | 8.73E-12 | 8.58E-11 | up |
| Gstt1 | 1.3736075 | 8.88E-12 | 8.73E-11 | up |
| H2-D1 | 1.2603411 | 9.58E-12 | 9.39E-11 | up |
| Pkib | 2.4922584 | 1.02E-11 | 9.94E-11 | up |
| Psmb10 | 1.0386281 | 1.07E-11 | 1.05E-10 | up |
| AI467606 | 1.5877416 | 1.09E-11 | 1.06E-10 | up |
| Fut4 | 1.6931162 | 1.10E-11 | 1.07E-10 | up |
| Slc36a2 | 3.4886024 | 1.12E-11 | 1.09E-10 | up |
| Clec5a | 1.9035659 | 1.14E-11 | 1.11E-10 | up |
| Ppic | 1.0061495 | 1.15E-11 | 1.12E-10 | up |
| Fam26e | 1.9326536 | 1.17E-11 | 1.13E-10 | up |
| Haus8 | 1.1957795 | 1.25E-11 | 1.21E-10 | up |
| Pilrb2 | 3.1006418 | 1.27E-11 | 1.23E-10 | up |
| Cfh | 1.4740296 | 1.32E-11 | 1.27E-10 | up |
| Il18 | 2.1737834 | 1.35E-11 | 1.30E-10 | up |
| Nlrc4 | 2.4941645 | 1.36E-11 | 1.31E-10 | up |
| Gpx4 | 1.1164529 | 1.39E-11 | 1.34E-10 | up |
| Gpd1 | 1.1425539 | 1.44E-11 | 1.39E-10 | up |
| Fkbp7 | 1.3324678 | 1.52E-11 | 1.46E-10 | up |
| Sh2d1b1 | 2.6629429 | 1.54E-11 | 1.47E-10 | up |
| Tmem54 | 2.8729162 | 1.54E-11 | 1.48E-10 | up |
| Pdgfra | 1.4309844 | 1.58E-11 | 1.52E-10 | up |
| Inmt | 2.9400495 | 1.59E-11 | 1.52E-10 | up |
| Darc | 2.1622795 | 1.62E-11 | 1.55E-10 | up |
| Aldh3a1 | 3.713059 | 1.63E-11 | 1.55E-10 | up |
| Cox6b2 | 1.47035 | 1.65E-11 | 1.57E-10 | up |
| Pros1 | 1.0790445 | 1.66E-11 | 1.58E-10 | up |
| Entpd2 | 1.4475633 | 1.68E-11 | 1.60E-10 | up |
| Fam129b | 1.0054265 | 1.71E-11 | 1.62E-10 | up |
| Otop1 | 4.9290275 | 1.76E-11 | 1.67E-10 | up |
| Rnasel | 1.8661638 | 2.11E-11 | 1.99E-10 | up |
| Liph | 2.6298913 | 2.22E-11 | 2.09E-10 | up |
| Prrx1 | 1.4648369 | 2.26E-11 | 2.13E-10 | up |
| Runx3 | 1.7812788 | 2.33E-11 | 2.19E-10 | up |
| Slc7a8 | 1.5267815 | 2.33E-11 | 2.19E-10 | up |
| Fam196b | 2.42532 | 2.44E-11 | 2.28E-10 | up |
| Flt3 | 3.6479339 | 2.66E-11 | 2.48E-10 | up |
| Ugt1a7c | 2.5251677 | 2.70E-11 | 2.51E-10 | up |
| Pvrl1 | 1.2224595 | 2.86E-11 | 2.66E-10 | up |
| Adh7 | 3.1518339 | 3.03E-11 | 2.81E-10 | up |
| Ebpl | 1.445398 | 3.04E-11 | 2.81E-10 | up |
| Creg2 | 2.5045411 | 3.31E-11 | 3.05E-10 | up |
| Tpst1 | 1.3781428 | 3.53E-11 | 3.24E-10 | up |
| Lgals3bp | 1.171197 | 3.58E-11 | 3.28E-10 | up |
| H2afy2 | 1.5333521 | 3.77E-11 | 3.45E-10 | up |
| Serpinb9b | 3.8197675 | 3.77E-11 | 3.45E-10 | up |
| Amica1 | 1.5091672 | 3.82E-11 | 3.49E-10 | up |
| Creb3l3 | 3.4982775 | 3.83E-11 | 3.49E-10 | up |
| Sorcs1 | 3.7085533 | 4.00E-11 | 3.64E-10 | up |
| Cd9 | 1.0447708 | 4.12E-11 | 3.76E-10 | up |
| Efs | 1.6514915 | 4.29E-11 | 3.90E-10 | up |
| Synpo2 | 1.8381999 | 4.31E-11 | 3.91E-10 | up |
| Ccdc109b | 1.9583432 | 4.46E-11 | 4.05E-10 | up |
| Slc22a3 | 3.5549879 | 4.52E-11 | 4.10E-10 | up |
| Vwf | 1.738569 | 4.76E-11 | 4.30E-10 | up |
| Fblim1 | 1.2347166 | 4.89E-11 | 4.41E-10 | up |
| Sh2b2 | 1.8567977 | 4.98E-11 | 4.49E-10 | up |
| Slc9a4 | 3.5988902 | 5.10E-11 | 4.59E-10 | up |
| Il17ra | 1.0242856 | 5.16E-11 | 4.64E-10 | up |
| Pth1r | 1.8934793 | 5.20E-11 | 4.67E-10 | up |
| Rnf144a | 1.1298089 | 5.29E-11 | 4.74E-10 | up |
| Cldn1 | 1.2660146 | 5.32E-11 | 4.77E-10 | up |
| Clec11a | 1.5148003 | 5.45E-11 | 4.87E-10 | up |
| Ptprn | 2.8601072 | 5.67E-11 | 5.05E-10 | up |
| Prkcdbp | 1.1095078 | 5.77E-11 | 5.12E-10 | up |
| Ptpn5 | 5.3823572 | 5.89E-11 | 5.23E-10 | up |
| Ace | 1.2508828 | 6.06E-11 | 5.37E-10 | up |
| Sp110 | 1.6151444 | 6.41E-11 | 5.68E-10 | up |
| Nrxn2 | 2.4781368 | 6.82E-11 | 6.02E-10 | up |
| H2-Ea-ps | 2.0121364 | 6.87E-11 | 6.07E-10 | up |
| Zfp808 | 1.6540016 | 7.55E-11 | 6.65E-10 | up |
| Spib | 2.3812314 | 7.87E-11 | 6.92E-10 | up |
| Megf10 | 2.4605698 | 7.89E-11 | 6.94E-10 | up |
| Mboat1 | 2.0753617 | 8.26E-11 | 7.25E-10 | up |
| Susd2 | 1.5676616 | 8.43E-11 | 7.39E-10 | up |
| F630028O10Rik | 2.0908398 | 8.65E-11 | 7.55E-10 | up |
| Naip6 | 2.7756322 | 8.91E-11 | 7.77E-10 | up |
| Ypel2 | 1.0125888 | 9.07E-11 | 7.89E-10 | up |
| Sdcbp2 | 2.1249291 | 9.08E-11 | 7.90E-10 | up |
| Islr | 1.3675711 | 9.35E-11 | 8.12E-10 | up |
| Fam65c | 1.9139138 | 9.47E-11 | 8.21E-10 | up |
| 5430427O19Rik | 2.4920258 | 9.76E-11 | 8.45E-10 | up |
| Vcan | 1.2220447 | 1.08E-10 | 9.28E-10 | up |
| C1rb | 2.306799 | 1.08E-10 | 9.35E-10 | up |
| Colec12 | 1.2964531 | 1.10E-10 | 9.51E-10 | up |
| Card9 | 1.8910495 | 1.11E-10 | 9.59E-10 | up |
| Bok | 1.6080609 | 1.25E-10 | 1.07E-09 | up |
| Adamdec1 | 4.0947834 | 1.35E-10 | 1.15E-09 | up |
| S100a4 | 1.5705378 | 1.36E-10 | 1.16E-09 | up |
| Lpar1 | 1.5414809 | 1.48E-10 | 1.26E-09 | up |
| Osm | 3.169982 | 1.54E-10 | 1.31E-09 | up |
| 2410006H16Rik | 1.0478024 | 1.61E-10 | 1.36E-09 | up |
| Elf4 | 1.3134684 | 1.63E-10 | 1.38E-09 | up |
| Map3k8 | 1.5686267 | 1.64E-10 | 1.39E-09 | up |
| Apod | 2.21246 | 1.67E-10 | 1.41E-09 | up |
| G0s2 | 1.2274768 | 1.67E-10 | 1.41E-09 | up |
| Sec1 | 3.2878784 | 1.71E-10 | 1.44E-09 | up |
| Tmcc3 | 1.0332903 | 1.73E-10 | 1.46E-09 | up |
| Gpr65 | 1.5382471 | 1.75E-10 | 1.47E-09 | up |
| Sprr2g | 5.1156913 | 1.77E-10 | 1.49E-09 | up |
| Itgb7 | 2.2328689 | 1.79E-10 | 1.50E-09 | up |
| Ltf | 5.0180198 | 1.86E-10 | 1.55E-09 | up |
| Rasl11a | 1.9039588 | 1.94E-10 | 1.62E-09 | up |
| Cubn | 2.7137525 | 1.96E-10 | 1.63E-09 | up |
| Arl5c | 1.9012133 | 2.04E-10 | 1.69E-09 | up |
| Grik5 | 1.2617365 | 2.07E-10 | 1.71E-09 | up |
| Pdlim4 | 1.6171149 | 2.07E-10 | 1.72E-09 | up |
| Col8a2 | 1.7270806 | 2.14E-10 | 1.77E-09 | up |
| Angpt1 | 2.1178272 | 2.17E-10 | 1.79E-09 | up |
| Dcstamp | 1.8982794 | 2.18E-10 | 1.80E-09 | up |
| Atoh8 | 2.0231553 | 2.26E-10 | 1.86E-09 | up |
| Gcnt4 | 1.8220926 | 2.27E-10 | 1.87E-09 | up |
| Gm5105 | 3.3662966 | 2.29E-10 | 1.89E-09 | up |
| Hcst | 2.3359053 | 2.37E-10 | 1.95E-09 | up |
| Tk1 | 1.2135971 | 2.42E-10 | 1.99E-09 | up |
| Rgs14 | 1.9327765 | 2.65E-10 | 2.16E-09 | up |
| AI427809 | 1.9391882 | 2.66E-10 | 2.18E-09 | up |
| Sdc3 | 1.2332665 | 2.98E-10 | 2.42E-09 | up |
| Gpr141 | 2.982946 | 3.03E-10 | 2.46E-09 | up |
| Slitrk6 | 4.0148213 | 3.04E-10 | 2.47E-09 | up |
| Col14a1 | 1.2973834 | 3.38E-10 | 2.73E-09 | up |
| Plscr4 | 1.62135 | 3.39E-10 | 2.74E-09 | up |
| Adora3 | 2.5388058 | 3.42E-10 | 2.76E-09 | up |
| Bgn | 1.1566791 | 3.46E-10 | 2.79E-09 | up |
| Spink5 | 5.6135611 | 3.56E-10 | 2.86E-09 | up |
| Ptger2 | 2.009877 | 3.79E-10 | 3.03E-09 | up |
| Chrd | 1.8958403 | 3.84E-10 | 3.08E-09 | up |
| 6-Sep | 2.0375038 | 3.87E-10 | 3.09E-09 | up |
| ORF63 | 2.1036794 | 3.89E-10 | 3.11E-09 | up |
| Fbln5 | 1.0117047 | 4.07E-10 | 3.25E-09 | up |
| Cd180 | 1.9659014 | 4.17E-10 | 3.33E-09 | up |
| A230050P20Rik | 1.1980277 | 4.21E-10 | 3.36E-09 | up |
| Krt14 | 5.5044556 | 4.36E-10 | 3.47E-09 | up |
| C430049B03Rik | 1.4490479 | 4.42E-10 | 3.52E-09 | up |
| Col27a1 | 1.8020088 | 4.46E-10 | 3.54E-09 | up |
| Kcnh2 | 2.7194039 | 4.51E-10 | 3.59E-09 | up |
| Clec4a1 | 1.9369817 | 4.63E-10 | 3.67E-09 | up |
| Gpr88 | 3.2407371 | 4.97E-10 | 3.92E-09 | up |
| Bicc1 | 1.7138485 | 5.10E-10 | 4.02E-09 | up |
| Adamts2 | 1.4197872 | 5.14E-10 | 4.05E-09 | up |
| Defb1 | 5.1742282 | 5.23E-10 | 4.12E-09 | up |
| Shc2 | 1.7672466 | 5.25E-10 | 4.13E-09 | up |
| Prss12 | 2.3347397 | 5.27E-10 | 4.14E-09 | up |
| Col17a1 | 4.2602264 | 5.35E-10 | 4.20E-09 | up |
| Dram1 | 1.1875367 | 5.70E-10 | 4.46E-09 | up |
| Sema3c | 1.4728468 | 6.21E-10 | 4.85E-09 | up |
| Psmb9 | 1.5775892 | 6.22E-10 | 4.86E-09 | up |
| Srxn1 | 1.1591775 | 6.31E-10 | 4.92E-09 | up |
| Ifi204 | 1.6224212 | 6.40E-10 | 4.99E-09 | up |
| Cyfip2 | 2.698768 | 6.59E-10 | 5.13E-09 | up |
| Inha | 2.0000871 | 6.72E-10 | 5.23E-09 | up |
| Zfyve28 | 1.9495592 | 7.01E-10 | 5.44E-09 | up |
| Ifitm10 | 2.4281199 | 7.15E-10 | 5.55E-09 | up |
| Tgm1 | 2.5579509 | 7.25E-10 | 5.61E-09 | up |
| Leprel2 | 1.2131958 | 7.40E-10 | 5.72E-09 | up |
| Igsf10 | 1.7658942 | 7.63E-10 | 5.90E-09 | up |
| Susd3 | 1.5912376 | 7.67E-10 | 5.92E-09 | up |
| Calr3 | 1.5195672 | 7.89E-10 | 6.07E-09 | up |
| Srpx2 | 1.3897058 | 7.96E-10 | 6.12E-09 | up |
| Atp6v1c2 | 2.4529574 | 7.97E-10 | 6.13E-09 | up |
| Sema3e | 2.5970069 | 8.07E-10 | 6.20E-09 | up |
| Fgf7 | 1.6849061 | 8.30E-10 | 6.37E-09 | up |
| Faxc | 1.5319906 | 8.32E-10 | 6.39E-09 | up |
| Ssu2 | 2.4841964 | 8.38E-10 | 6.43E-09 | up |
| Slamf6 | 2.9936436 | 8.60E-10 | 6.58E-09 | up |
| Agbl2 | 1.7821279 | 8.72E-10 | 6.66E-09 | up |
| Adh1 | 2.4427544 | 9.07E-10 | 6.91E-09 | up |
| C1qtnf2 | 2.1418126 | 9.89E-10 | 7.49E-09 | up |
| 2310007B03Rik | 1.7740413 | 9.96E-10 | 7.54E-09 | up |
| Mmp3 | 2.3879443 | 1.01E-09 | 7.67E-09 | up |
| Pln | 4.7182248 | 1.04E-09 | 7.84E-09 | up |
| Hgf | 2.3430583 | 1.05E-09 | 7.89E-09 | up |
| Niacr1 | 3.1918503 | 1.05E-09 | 7.89E-09 | up |
| Ablim1 | 1.0980407 | 1.13E-09 | 8.47E-09 | up |
| Uba7 | 1.0918272 | 1.18E-09 | 8.84E-09 | up |
| Ltb4r1 | 2.1771825 | 1.18E-09 | 8.89E-09 | up |
| Fgd2 | 1.4260239 | 1.23E-09 | 9.21E-09 | up |
| St6galnac2 | 1.6387306 | 1.27E-09 | 9.51E-09 | up |
| Ggh | 1.2620596 | 1.32E-09 | 9.81E-09 | up |
| Slfn2 | 1.4164539 | 1.33E-09 | 9.91E-09 | up |
| Figf | 1.6684986 | 1.35E-09 | 1.00E-08 | up |
| Pcdh8 | 3.6489991 | 1.36E-09 | 1.01E-08 | up |
| Itpripl2 | 1.2336046 | 1.37E-09 | 1.02E-08 | up |
| A4galt | 1.6573854 | 1.42E-09 | 1.05E-08 | up |
| Kctd15 | 1.6138474 | 1.45E-09 | 1.07E-08 | up |
| Slc6a14 | 1.6997335 | 1.50E-09 | 1.11E-08 | up |
| Tesc | 1.4544301 | 1.51E-09 | 1.12E-08 | up |
| Aqp4 | 2.6648917 | 1.58E-09 | 1.17E-08 | up |
| Ier3 | 1.4987319 | 1.60E-09 | 1.18E-08 | up |
| Morc4 | 1.0134617 | 1.61E-09 | 1.19E-08 | up |
| Ppt2 | 1.179094 | 1.62E-09 | 1.19E-08 | up |
| Tagln | 1.0038228 | 1.68E-09 | 1.24E-08 | up |
| Vipr2 | 2.4438498 | 1.68E-09 | 1.24E-08 | up |
| Pfkfb4 | 1.3459417 | 1.69E-09 | 1.24E-08 | up |
| Alpk1 | 1.0257651 | 1.70E-09 | 1.25E-08 | up |
| Trim29 | 4.0357673 | 1.75E-09 | 1.28E-08 | up |
| Gpc6 | 1.3612054 | 1.76E-09 | 1.29E-08 | up |
| Fut10 | 1.6801837 | 1.82E-09 | 1.33E-08 | up |
| Chst1 | 1.6676995 | 1.89E-09 | 1.38E-08 | up |
| Kcnk13 | 1.7783171 | 1.98E-09 | 1.44E-08 | up |
| Sox21 | 3.8111623 | 2.04E-09 | 1.48E-08 | up |
| Abca9 | 1.374928 | 2.08E-09 | 1.51E-08 | up |
| Rdh1 | 2.8367068 | 2.27E-09 | 1.64E-08 | up |
| Pex11a | 1.0353029 | 2.30E-09 | 1.66E-08 | up |
| Ldoc1 | 8.6519505 | 2.30E-09 | 1.66E-08 | up |
| Krt15 | 4.5622207 | 2.31E-09 | 1.66E-08 | up |
| Mfsd12 | 1.1217472 | 2.31E-09 | 1.66E-08 | up |
| Meox2 | 1.5152929 | 2.38E-09 | 1.71E-08 | up |
| Gpm6b | 1.3459742 | 2.42E-09 | 1.74E-08 | up |
| Numbl | 1.1883352 | 2.48E-09 | 1.78E-08 | up |
| Gbp8 | 2.8747943 | 2.65E-09 | 1.90E-08 | up |
| Igj | 3.6312053 | 2.66E-09 | 1.90E-08 | up |
| Fasn | 1.2858813 | 2.73E-09 | 1.95E-08 | up |
| Evc | 1.5212767 | 2.76E-09 | 1.97E-08 | up |
| Mfap5 | 1.2492196 | 2.78E-09 | 1.98E-08 | up |
| Gpr68 | 2.2491959 | 2.78E-09 | 1.98E-08 | up |
| Tmem177 | 1.1877762 | 2.78E-09 | 1.98E-08 | up |
| Asic1 | 1.1734993 | 2.79E-09 | 1.98E-08 | up |
| Acaa1b | 2.3581637 | 2.85E-09 | 2.02E-08 | up |
| Glt8d2 | 2.2527861 | 2.86E-09 | 2.03E-08 | up |
| Slc2a6 | 1.5520671 | 2.89E-09 | 2.05E-08 | up |
| Col3a1 | 1.5526877 | 2.92E-09 | 2.07E-08 | up |
| Stk17b | 1.1819768 | 2.97E-09 | 2.10E-08 | up |
| Clec4b1 | 2.799893 | 2.98E-09 | 2.11E-08 | up |
| Prkca | 1.0848556 | 3.06E-09 | 2.16E-08 | up |
| Dnajb13 | 2.5519038 | 3.27E-09 | 2.30E-08 | up |
| Ecm1 | 1.1344556 | 3.27E-09 | 2.30E-08 | up |
| Krt20 | 4.6771233 | 3.31E-09 | 2.33E-08 | up |
| Hist1h4h | 1.6465077 | 3.37E-09 | 2.37E-08 | up |
| Nova1 | 1.4400889 | 3.56E-09 | 2.49E-08 | up |
| Hopx | 1.3591203 | 3.56E-09 | 2.49E-08 | up |
| Matn3 | 3.4493003 | 3.59E-09 | 2.51E-08 | up |
| Gm5086 | 2.3354652 | 3.62E-09 | 2.53E-08 | up |
| Tmem158 | 1.3901535 | 3.68E-09 | 2.57E-08 | up |
| Klhl6 | 1.3227808 | 3.74E-09 | 2.61E-08 | up |
| Msln | 4.4881248 | 3.75E-09 | 2.62E-08 | up |
| Cdc45 | 1.1195462 | 3.79E-09 | 2.64E-08 | up |
| Upk3bl | 1.8082464 | 3.83E-09 | 2.67E-08 | up |
| Dnm3os | 1.8052231 | 3.84E-09 | 2.67E-08 | up |
| Svep1 | 1.2415669 | 3.84E-09 | 2.67E-08 | up |
| Upk1b | 4.126679 | 3.99E-09 | 2.77E-08 | up |
| Hepacam2 | 2.8842743 | 4.06E-09 | 2.82E-08 | up |
| Eppin | 3.2018058 | 4.12E-09 | 2.86E-08 | up |
| Col16a1 | 1.2545306 | 4.21E-09 | 2.92E-08 | up |
| Ntn3 | 2.1947442 | 4.33E-09 | 2.99E-08 | up |
| Sprr1a | 4.1899474 | 4.43E-09 | 3.06E-08 | up |
| Uck2 | 1.3635636 | 4.43E-09 | 3.06E-08 | up |
| Serpina3i | 2.4293926 | 4.46E-09 | 3.08E-08 | up |
| Syt15 | 1.4713082 | 4.91E-09 | 3.38E-08 | up |
| 9530077C05Rik | 1.4350538 | 4.97E-09 | 3.42E-08 | up |
| Tmem173 | 1.0561278 | 5.01E-09 | 3.45E-08 | up |
| 4930404N11Rik | 1.4229657 | 5.06E-09 | 3.48E-08 | up |
| Il20rb | 1.8159661 | 5.08E-09 | 3.48E-08 | up |
| Slc41a3 | 1.4663026 | 5.14E-09 | 3.52E-08 | up |
| Fscn1 | 1.0320956 | 5.27E-09 | 3.61E-08 | up |
| Ak1 | 1.7732972 | 5.31E-09 | 3.63E-08 | up |
| Frzb | 2.2967695 | 5.32E-09 | 3.64E-08 | up |
| Gypc | 1.5386369 | 5.36E-09 | 3.66E-08 | up |
| Gpm6a | 2.8239897 | 5.41E-09 | 3.70E-08 | up |
| Il21r | 1.8627597 | 5.49E-09 | 3.75E-08 | up |
| Kif21b | 1.4904984 | 5.50E-09 | 3.75E-08 | up |
| Gm11545 | 2.8563298 | 5.51E-09 | 3.76E-08 | up |
| Zcchc5 | 3.2122536 | 5.63E-09 | 3.83E-08 | up |
| Acta2 | 1.025306 | 5.78E-09 | 3.94E-08 | up |
| Metrn | 1.567917 | 5.88E-09 | 4.00E-08 | up |
| Gm13710 | 2.6227265 | 5.92E-09 | 4.02E-08 | up |
| Il1a | 3.1558608 | 6.22E-09 | 4.22E-08 | up |
| Adamts10 | 1.0472784 | 6.41E-09 | 4.34E-08 | up |
| Abcb1b | 1.8998654 | 6.87E-09 | 4.64E-08 | up |
| Fat3 | 2.9989217 | 6.98E-09 | 4.71E-08 | up |
| Vnn1 | 2.1915901 | 7.02E-09 | 4.73E-08 | up |
| Bcl3 | 1.4183394 | 7.03E-09 | 4.74E-08 | up |
| Sh3bp1 | 1.1223476 | 7.18E-09 | 4.83E-08 | up |
| Itgbl1 | 1.5650556 | 7.20E-09 | 4.85E-08 | up |
| AA414768 | 1.5121367 | 7.39E-09 | 4.96E-08 | up |
| Il18bp | 1.4058867 | 7.40E-09 | 4.97E-08 | up |
| 6030419C18Rik | 1.5537873 | 7.50E-09 | 5.03E-08 | up |
| Ooep | 1.2221126 | 7.73E-09 | 5.19E-08 | up |
| Gpr81 | 1.8248829 | 7.91E-09 | 5.30E-08 | up |
| Sox6 | 1.9726621 | 7.99E-09 | 5.35E-08 | up |
| Fcrls | 1.1799591 | 8.03E-09 | 5.37E-08 | up |
| Crip1 | 1.0747161 | 8.34E-09 | 5.57E-08 | up |
| Plk3 | 1.3366081 | 8.54E-09 | 5.70E-08 | up |
| Ces1f | 2.8230797 | 8.63E-09 | 5.75E-08 | up |
| Kmo | 2.1539116 | 8.79E-09 | 5.85E-08 | up |
| Jazf1 | 1.3333467 | 9.10E-09 | 6.04E-08 | up |
| Acsm1 | 4.1280827 | 9.46E-09 | 6.27E-08 | up |
| Fap | 1.7087071 | 9.83E-09 | 6.49E-08 | up |
| Trpv6 | 2.6012216 | 9.99E-09 | 6.59E-08 | up |
| Eif4e3 | 1.0230332 | 1.09E-08 | 7.18E-08 | up |
| Ebf2 | 1.1962107 | 1.10E-08 | 7.20E-08 | up |
| Klhl38 | 3.2986423 | 1.13E-08 | 7.41E-08 | up |
| Islr2 | 1.5275007 | 1.17E-08 | 7.65E-08 | up |
| Thbd | 1.0137417 | 1.19E-08 | 7.77E-08 | up |
| Pcsk6 | 1.3561208 | 1.21E-08 | 7.89E-08 | up |
| Rapsn | 2.3707166 | 1.29E-08 | 8.40E-08 | up |
| Rspo2 | 2.1821572 | 1.36E-08 | 8.83E-08 | up |
| Phlda2 | 2.3911603 | 1.37E-08 | 8.89E-08 | up |
| Orm1 | 3.3850753 | 1.40E-08 | 9.07E-08 | up |
| Cxcl2 | 3.9923901 | 1.43E-08 | 9.25E-08 | up |
| Fam26f | 2.8187837 | 1.44E-08 | 9.29E-08 | up |
| Batf3 | 2.0463521 | 1.48E-08 | 9.52E-08 | up |
| Colq | 4.3158908 | 1.54E-08 | 9.91E-08 | up |
| Dixdc1 | 1.0285024 | 1.61E-08 | 1.03E-07 | up |
| Zmynd15 | 1.3570638 | 1.70E-08 | 1.09E-07 | up |
| Galnt16 | 1.306594 | 1.71E-08 | 1.10E-07 | up |
| Ptplad2 | 1.3144128 | 1.74E-08 | 1.11E-07 | up |
| Ebf3 | 1.1887129 | 1.77E-08 | 1.13E-07 | up |
| Hfe | 1.0390631 | 1.81E-08 | 1.15E-07 | up |
| Ednra | 1.2150301 | 1.86E-08 | 1.18E-07 | up |
| 6720489N17Rik | 1.5348083 | 1.86E-08 | 1.18E-07 | up |
| Aoah | 2.0090777 | 1.88E-08 | 1.20E-07 | up |
| Gm6377 | 2.5804887 | 1.90E-08 | 1.21E-07 | up |
| Olfml2b | 1.1320019 | 1.93E-08 | 1.23E-07 | up |
| Cpz | 3.1147349 | 1.93E-08 | 1.23E-07 | up |
| Grip2 | 2.6063985 | 1.94E-08 | 1.23E-07 | up |
| Ube2c | 1.1247183 | 1.96E-08 | 1.24E-07 | up |
| Gas6 | 1.0921178 | 1.97E-08 | 1.25E-07 | up |
| Scd4 | 2.0498976 | 2.06E-08 | 1.30E-07 | up |
| Igf1 | 1.3476689 | 2.07E-08 | 1.31E-07 | up |
| Gas2l3 | 1.2789677 | 2.08E-08 | 1.31E-07 | up |
| Slc35d2 | 1.1169815 | 2.08E-08 | 1.32E-07 | up |
| Rps4y2 | 1.3578632 | 2.10E-08 | 1.33E-07 | up |
| Col1a1 | 1.4313574 | 2.11E-08 | 1.33E-07 | up |
| Fpr2 | 3.4152094 | 2.14E-08 | 1.35E-07 | up |
| Shc3 | 2.2008252 | 2.14E-08 | 1.35E-07 | up |
| Plaur | 1.4151295 | 2.21E-08 | 1.39E-07 | up |
| Dok3 | 1.3744014 | 2.33E-08 | 1.46E-07 | up |
| C920025E04Rik | 1.0748559 | 2.45E-08 | 1.53E-07 | up |
| Ethe1 | 1.109995 | 2.51E-08 | 1.57E-07 | up |
| Hspb8 | 1.6968364 | 2.63E-08 | 1.64E-07 | up |
| Abhd15 | 1.5290307 | 2.68E-08 | 1.67E-07 | up |
| Ssc5d | 1.6452626 | 2.69E-08 | 1.68E-07 | up |
| Scn7a | 1.3058033 | 2.81E-08 | 1.75E-07 | up |
| Epb4.1l3 | 1.2744534 | 2.95E-08 | 1.83E-07 | up |
| Aspn | 1.4585221 | 2.98E-08 | 1.85E-07 | up |
| Btla | 2.1748854 | 3.12E-08 | 1.93E-07 | up |
| Kctd8 | 1.332019 | 3.14E-08 | 1.94E-07 | up |
| Lbp | 1.3546556 | 3.22E-08 | 1.99E-07 | up |
| Paqr8 | 1.3149872 | 3.38E-08 | 2.08E-07 | up |
| Cebpd | 1.49897 | 3.51E-08 | 2.16E-07 | up |
| Mypop | 1.2043458 | 3.52E-08 | 2.16E-07 | up |
| Gm5077 | 1.5611111 | 3.54E-08 | 2.17E-07 | up |
| Slc5a1 | 2.7420513 | 3.59E-08 | 2.20E-07 | up |
| Fxyd6 | 2.2457962 | 3.66E-08 | 2.24E-07 | up |
| Nfatc4 | 1.2407783 | 3.67E-08 | 2.25E-07 | up |
| Entpd1 | 1.1228608 | 3.75E-08 | 2.29E-07 | up |
| Srpx | 1.5411767 | 3.87E-08 | 2.36E-07 | up |
| Ccl5 | 2.3237731 | 3.91E-08 | 2.38E-07 | up |
| Ptger4 | 1.2460853 | 3.98E-08 | 2.42E-07 | up |
| Cox8b | 4.0032445 | 4.07E-08 | 2.47E-07 | up |
| Arhgap4 | 1.2450763 | 4.11E-08 | 2.49E-07 | up |
| Lag3 | 1.8996671 | 4.34E-08 | 2.62E-07 | up |
| Hpx | 2.6312807 | 4.37E-08 | 2.64E-07 | up |
| Pcolce2 | 1.5869874 | 4.56E-08 | 2.74E-07 | up |
| Adcy3 | 1.2693553 | 4.88E-08 | 2.93E-07 | up |
| Rinl | 1.2671721 | 4.89E-08 | 2.93E-07 | up |
| Dnph1 | 1.6007264 | 4.96E-08 | 2.97E-07 | up |
| Lrrc4 | 2.3008071 | 4.98E-08 | 2.99E-07 | up |
| Gpr171 | 3.0580869 | 5.08E-08 | 3.04E-07 | up |
| Fxyd1 | 1.9137492 | 5.13E-08 | 3.07E-07 | up |
| Krt17 | 3.2312597 | 5.16E-08 | 3.08E-07 | up |
| P2ry10 | 3.0318069 | 5.57E-08 | 3.31E-07 | up |
| Gm13251 | 1.129181 | 5.58E-08 | 3.32E-07 | up |
| Kcnq5 | 3.2645406 | 5.83E-08 | 3.46E-07 | up |
| Ndn | 1.3596573 | 5.89E-08 | 3.49E-07 | up |
| Il2rg | 1.1876702 | 6.03E-08 | 3.57E-07 | up |
| Serpinb2 | 2.7535443 | 6.03E-08 | 3.57E-07 | up |
| Fabp3 | 3.6645014 | 6.19E-08 | 3.66E-07 | up |
| Ghr | 1.1236122 | 6.36E-08 | 3.75E-07 | up |
| Bdh2 | 2.2665573 | 6.37E-08 | 3.76E-07 | up |
| Npr3 | 1.2003097 | 6.67E-08 | 3.92E-07 | up |
| Sytl1 | 1.1890523 | 6.99E-08 | 4.10E-07 | up |
| Adipoq | 2.93772 | 7.18E-08 | 4.20E-07 | up |
| Palm | 1.1340601 | 7.22E-08 | 4.23E-07 | up |
| Gdf6 | 1.3280722 | 7.27E-08 | 4.25E-07 | up |
| Stmn2 | 1.4825314 | 7.43E-08 | 4.34E-07 | up |
| Ankrd55 | 2.6712116 | 7.69E-08 | 4.49E-07 | up |
| Clca4 | 2.8746741 | 8.20E-08 | 4.77E-07 | up |
| Rprm | 1.7301622 | 8.26E-08 | 4.81E-07 | up |
| H2-M3 | 1.2013998 | 8.34E-08 | 4.85E-07 | up |
| Slc22a4 | 2.8449437 | 8.54E-08 | 4.96E-07 | up |
| Ccdc33 | 3.5520522 | 8.63E-08 | 5.01E-07 | up |
| Fetub | 1.8751599 | 8.89E-08 | 5.15E-07 | up |
| Sult4a1 | 2.6565964 | 9.16E-08 | 5.29E-07 | up |
| Kcp | 1.5753067 | 9.26E-08 | 5.34E-07 | up |
| Olfr78 | 2.2880955 | 9.54E-08 | 5.49E-07 | up |
| Tspyl3 | 1.2336907 | 9.76E-08 | 5.61E-07 | up |
| Serpina3n | 1.930722 | 9.94E-08 | 5.70E-07 | up |
| Cys1 | 1.5919316 | 9.96E-08 | 5.71E-07 | up |
| Ccl3 | 2.8266096 | 1.00E-07 | 5.74E-07 | up |
| Irf4 | 1.8250213 | 1.00E-07 | 5.75E-07 | up |
| Spsb4 | 1.9800732 | 1.03E-07 | 5.88E-07 | up |
| Bmx | 1.7245952 | 1.07E-07 | 6.08E-07 | up |
| Lrrc10b | 2.1609403 | 1.07E-07 | 6.09E-07 | up |
| Siglece | 1.5093639 | 1.11E-07 | 6.34E-07 | up |
| Ltb | 2.3983328 | 1.12E-07 | 6.37E-07 | up |
| Apol6 | 2.324044 | 1.15E-07 | 6.52E-07 | up |
| Comp | 1.2538661 | 1.15E-07 | 6.54E-07 | up |
| Sox11 | 2.3439559 | 1.17E-07 | 6.61E-07 | up |
| Pglyrp2 | 3.1836179 | 1.17E-07 | 6.64E-07 | up |
| Gamt | 1.3069065 | 1.17E-07 | 6.64E-07 | up |
| Fpr1 | 3.515362 | 1.20E-07 | 6.77E-07 | up |
| Itga11 | 1.5216176 | 1.22E-07 | 6.90E-07 | up |
| Cxcl10 | 2.1239098 | 1.23E-07 | 6.94E-07 | up |
| Wdr72 | 2.9224766 | 1.23E-07 | 6.96E-07 | up |
| C130050O18Rik | 1.7167183 | 1.27E-07 | 7.18E-07 | up |
| Cyp2f2 | 4.1692484 | 1.28E-07 | 7.19E-07 | up |
| Ptgir | 1.2992459 | 1.29E-07 | 7.24E-07 | up |
| Slc39a2 | 2.5126437 | 1.31E-07 | 7.38E-07 | up |
| Cbr2 | 2.2947737 | 1.34E-07 | 7.55E-07 | up |
| D630003M21Rik | 1.0527191 | 1.35E-07 | 7.58E-07 | up |
| Kcna6 | 2.9833597 | 1.37E-07 | 7.68E-07 | up |
| Vipr1 | 1.3316866 | 1.39E-07 | 7.81E-07 | up |
| Kcnk3 | 1.878198 | 1.41E-07 | 7.89E-07 | up |
| Lmo1 | 1.9207191 | 1.52E-07 | 8.45E-07 | up |
| Clmp | 1.2977907 | 1.57E-07 | 8.73E-07 | up |
| Rrm2 | 1.2534386 | 1.60E-07 | 8.86E-07 | up |
| Gm13889 | 1.180254 | 1.60E-07 | 8.87E-07 | up |
| Dapl1 | 3.3252619 | 1.64E-07 | 9.07E-07 | up |
| Icos | 2.9841659 | 1.65E-07 | 9.13E-07 | up |
| Snx30 | 1.1066078 | 1.69E-07 | 9.36E-07 | up |
| Sox2 | 4.1491862 | 1.75E-07 | 9.65E-07 | up |
| Lhfpl2 | 1.0722044 | 1.77E-07 | 9.80E-07 | up |
| Lrrc16b | 1.5235153 | 1.78E-07 | 9.81E-07 | up |
| Mt1 | 1.1141665 | 1.80E-07 | 9.95E-07 | up |
| Pcolce | 1.0195933 | 1.92E-07 | 1.06E-06 | up |
| Radil | 1.3486956 | 1.94E-07 | 1.07E-06 | up |
| Fndc5 | 2.9908154 | 1.94E-07 | 1.07E-06 | up |
| Omd | 1.67603 | 1.96E-07 | 1.08E-06 | up |
| Hoxa2 | 1.9194366 | 2.01E-07 | 1.10E-06 | up |
| 2010001M06Rik | 1.4982226 | 2.09E-07 | 1.14E-06 | up |
| Apitd1 | 1.50535 | 2.12E-07 | 1.16E-06 | up |
| Fam198a | 1.9832044 | 2.14E-07 | 1.17E-06 | up |
| Penk | 1.5425861 | 2.20E-07 | 1.20E-06 | up |
| Il27ra | 1.7189842 | 2.22E-07 | 1.21E-06 | up |
| Rhoh | 2.0051963 | 2.34E-07 | 1.28E-06 | up |
| Clstn3 | 3.1157002 | 2.42E-07 | 1.31E-06 | up |
| Syt7 | 1.4072748 | 2.44E-07 | 1.32E-06 | up |
| Pglyrp1 | 2.6814605 | 2.46E-07 | 1.33E-06 | up |
| Slc13a3 | 1.706921 | 2.57E-07 | 1.39E-06 | up |
| Mcm6 | 1.0653168 | 2.61E-07 | 1.41E-06 | up |
| Ecm2 | 1.3140383 | 2.61E-07 | 1.41E-06 | up |
| Fndc1 | 1.1875445 | 2.64E-07 | 1.43E-06 | up |
| Col5a2 | 1.0517233 | 2.77E-07 | 1.49E-06 | up |
| Ms4a6b | 1.3080046 | 2.77E-07 | 1.49E-06 | up |
| Gli3 | 1.5879442 | 2.79E-07 | 1.50E-06 | up |
| Ntng2 | 1.4704804 | 2.82E-07 | 1.51E-06 | up |
| Prrg4 | 2.0560482 | 2.90E-07 | 1.55E-06 | up |
| C1qtnf9 | 1.7423006 | 2.92E-07 | 1.56E-06 | up |
| Sult1a1 | 1.0704784 | 2.93E-07 | 1.57E-06 | up |
| Samsn1 | 1.6540265 | 2.96E-07 | 1.58E-06 | up |
| Ccrl2 | 1.2800641 | 2.96E-07 | 1.58E-06 | up |
| Cdh11 | 1.3706532 | 3.10E-07 | 1.65E-06 | up |
| Ifitm1 | 1.5691598 | 3.18E-07 | 1.69E-06 | up |
| 8430408G22Rik | 1.5584707 | 3.31E-07 | 1.76E-06 | up |
| Selenbp1 | 1.0261444 | 3.33E-07 | 1.77E-06 | up |
| Lefty1 | 1.5341406 | 3.53E-07 | 1.87E-06 | up |
| Cmklr1 | 1.08911 | 3.55E-07 | 1.87E-06 | up |
| Chd5 | 1.8963298 | 3.59E-07 | 1.89E-06 | up |
| Epyc | 1.8449043 | 3.59E-07 | 1.89E-06 | up |
| Slc16a7 | 2.231452 | 3.62E-07 | 1.90E-06 | up |
| Tekt4 | 1.159669 | 3.63E-07 | 1.91E-06 | up |
| Gadd45g | 1.1408026 | 3.63E-07 | 1.91E-06 | up |
| Lhx6 | 1.3768262 | 3.75E-07 | 1.97E-06 | up |
| Egr2 | 1.6496627 | 3.90E-07 | 2.04E-06 | up |
| Igfals | 2.4725572 | 3.95E-07 | 2.07E-06 | up |
| Rcsd1 | 1.2406208 | 3.97E-07 | 2.08E-06 | up |
| Adrb1 | 1.8257951 | 3.98E-07 | 2.08E-06 | up |
| Chrnd | 3.9182317 | 4.06E-07 | 2.12E-06 | up |
| 2010300C02Rik | 1.4162514 | 4.07E-07 | 2.12E-06 | up |
| Epha2 | 1.1416218 | 4.09E-07 | 2.13E-06 | up |
| Nyap1 | 1.3444895 | 4.13E-07 | 2.15E-06 | up |
| Neurl1a | 2.5333189 | 4.21E-07 | 2.20E-06 | up |
| Camk4 | 2.8061022 | 4.28E-07 | 2.23E-06 | up |
| Ifit3 | 1.2653728 | 4.33E-07 | 2.25E-06 | up |
| Lrrn4cl | 1.2690791 | 4.41E-07 | 2.29E-06 | up |
| Pdgfc | 1.0295922 | 4.46E-07 | 2.31E-06 | up |
| Tacr1 | 1.4563775 | 4.54E-07 | 2.36E-06 | up |
| Tnf | 2.4460835 | 4.58E-07 | 2.37E-06 | up |
| Hal | 1.9268043 | 4.66E-07 | 2.41E-06 | up |
| Gria3 | 1.2239183 | 4.68E-07 | 2.42E-06 | up |
| H2-K2 | 1.3036266 | 4.74E-07 | 2.44E-06 | up |
| Iffo1 | 1.0728505 | 4.85E-07 | 2.50E-06 | up |
| Slc22a1 | 2.278437 | 5.08E-07 | 2.61E-06 | up |
| Pi15 | 1.7349512 | 5.08E-07 | 2.61E-06 | up |
| Ppm1j | 1.2420917 | 5.28E-07 | 2.70E-06 | up |
| Ms4a4d | 1.5171162 | 5.42E-07 | 2.77E-06 | up |
| Ubxn10 | 1.2078157 | 5.44E-07 | 2.77E-06 | up |
| Snai2 | 1.2324759 | 5.50E-07 | 2.81E-06 | up |
| Bst1 | 2.5563887 | 5.56E-07 | 2.84E-06 | up |
| Trim30d | 4.6194069 | 5.58E-07 | 2.84E-06 | up |
| Lrrc26 | 2.066094 | 5.85E-07 | 2.97E-06 | up |
| Oxtr | 2.1844902 | 5.96E-07 | 3.03E-06 | up |
| Fbxo17 | 1.233168 | 6.05E-07 | 3.07E-06 | up |
| Cnrip1 | 1.2276101 | 6.13E-07 | 3.11E-06 | up |
| Frem1 | 1.7643995 | 6.15E-07 | 3.12E-06 | up |
| Prkg2 | 1.6538204 | 6.34E-07 | 3.21E-06 | up |
| Il16 | 1.4658549 | 6.35E-07 | 3.21E-06 | up |
| Bmp4 | 1.2793971 | 6.36E-07 | 3.21E-06 | up |
| Acnat1 | 1.2583444 | 6.41E-07 | 3.24E-06 | up |
| C1rl | 1.4847652 | 6.49E-07 | 3.27E-06 | up |
| C1qtnf7 | 1.6648039 | 6.52E-07 | 3.29E-06 | up |
| Lcn2 | 3.0223822 | 6.76E-07 | 3.40E-06 | up |
| Cxcl1 | 2.0912202 | 7.02E-07 | 3.52E-06 | up |
| Sh3rf2 | 2.9277369 | 7.05E-07 | 3.53E-06 | up |
| Arid5b | 1.2704311 | 7.09E-07 | 3.56E-06 | up |
| Aqp5 | 1.3387303 | 7.23E-07 | 3.62E-06 | up |
| Duoxa1 | 1.4441115 | 7.32E-07 | 3.66E-06 | up |
| Pi16 | 1.2634571 | 7.54E-07 | 3.76E-06 | up |
| Unc13a | 1.8173601 | 7.69E-07 | 3.83E-06 | up |
| Slfn8 | 1.3850808 | 7.72E-07 | 3.84E-06 | up |
| Eomes | 2.2230962 | 7.84E-07 | 3.91E-06 | up |
| Smyd1 | 2.8510081 | 7.98E-07 | 3.97E-06 | up |
| Dzip1l | 1.1411218 | 8.16E-07 | 4.05E-06 | up |
| Gys2 | 3.6292178 | 8.21E-07 | 4.08E-06 | up |
| Pde1b | 1.0976261 | 8.21E-07 | 4.08E-06 | up |
| 9230105E05Rik | 1.8330413 | 8.22E-07 | 4.08E-06 | up |
| Rasgrp1 | 2.8752762 | 8.38E-07 | 4.15E-06 | up |
| Runx1t1 | 1.5407761 | 8.44E-07 | 4.18E-06 | up |
| Slc39a4 | 2.7153822 | 8.45E-07 | 4.18E-06 | up |
| Pla2g4f | 1.9568375 | 8.45E-07 | 4.18E-06 | up |
| Plb1 | 1.7158789 | 8.58E-07 | 4.24E-06 | up |
| Dennd1c | 1.088888 | 8.66E-07 | 4.28E-06 | up |
| Ang | 1.6453497 | 9.28E-07 | 4.57E-06 | up |
| Prom1 | 2.3157642 | 9.30E-07 | 4.57E-06 | up |
| P2rx5 | 2.4479163 | 9.66E-07 | 4.73E-06 | up |
| Gpr162 | 1.7266322 | 9.78E-07 | 4.79E-06 | up |
| Xcr1 | 2.3008384 | 1.01E-06 | 4.92E-06 | up |
| Il18r1 | 1.2505215 | 1.03E-06 | 5.00E-06 | up |
| B3gnt9 | 1.1426742 | 1.03E-06 | 5.00E-06 | up |
| Lrfn3 | 1.1647246 | 1.06E-06 | 5.17E-06 | up |
| Acpp | 1.555776 | 1.10E-06 | 5.32E-06 | up |
| Wnk3 | 1.9504347 | 1.12E-06 | 5.44E-06 | up |
| C130026I21Rik | 2.057615 | 1.15E-06 | 5.55E-06 | up |
| Stmnd1 | 3.0566993 | 1.18E-06 | 5.69E-06 | up |
| Ccr7 | 2.2931429 | 1.18E-06 | 5.72E-06 | up |
| Pxdc1 | 1.3533399 | 1.19E-06 | 5.72E-06 | up |
| Myb | 2.2250186 | 1.21E-06 | 5.84E-06 | up |
| Pf4 | 1.5903385 | 1.23E-06 | 5.94E-06 | up |
| Mgam | 2.1151938 | 1.25E-06 | 6.02E-06 | up |
| Gm5431 | 1.7322553 | 1.28E-06 | 6.16E-06 | up |
| Il1rl2 | 1.0982601 | 1.29E-06 | 6.21E-06 | up |
| Gpr126 | 1.5965466 | 1.31E-06 | 6.27E-06 | up |
| Krt4 | 3.5017961 | 1.32E-06 | 6.31E-06 | up |
| Scgb3a2 | 4.4132061 | 1.39E-06 | 6.63E-06 | up |
| Ppp1r3c | 1.0876656 | 1.40E-06 | 6.66E-06 | up |
| Entpd3 | 1.6355215 | 1.42E-06 | 6.78E-06 | up |
| Kcnk6 | 1.1485893 | 1.43E-06 | 6.80E-06 | up |
| 6530402F18Rik | 1.2091888 | 1.49E-06 | 7.07E-06 | up |
| Fbp2 | 2.0490142 | 1.49E-06 | 7.07E-06 | up |
| Fam84a | 1.4996281 | 1.49E-06 | 7.07E-06 | up |
| Dnahc6 | 2.4706348 | 1.50E-06 | 7.12E-06 | up |
| Itgae | 2.0747545 | 1.53E-06 | 7.24E-06 | up |
| Clic6 | 3.3246786 | 1.53E-06 | 7.25E-06 | up |
| Cacna1a | 1.1448061 | 1.53E-06 | 7.25E-06 | up |
| Rerg | 1.7409893 | 1.54E-06 | 7.30E-06 | up |
| Qpct | 1.5265832 | 1.61E-06 | 7.61E-06 | up |
| Rac3 | 1.1414292 | 1.62E-06 | 7.61E-06 | up |
| Bdnf | 1.4145906 | 1.62E-06 | 7.63E-06 | up |
| Dmpk | 1.2552204 | 1.63E-06 | 7.68E-06 | up |
| 2310007L24Rik | 2.6631886 | 1.68E-06 | 7.89E-06 | up |
| Styk1 | 2.5619654 | 1.69E-06 | 7.94E-06 | up |
| Gm3604 | 1.2901698 | 1.74E-06 | 8.17E-06 | up |
| Cysltr1 | 1.3463404 | 1.76E-06 | 8.26E-06 | up |
| Htr2b | 2.3994096 | 1.80E-06 | 8.41E-06 | up |
| Rassf9 | 1.5091066 | 1.83E-06 | 8.57E-06 | up |
| Casp12 | 1.2250437 | 1.84E-06 | 8.62E-06 | up |
| Mb21d1 | 1.2123364 | 1.86E-06 | 8.70E-06 | up |
| Igf2 | 2.1389813 | 1.92E-06 | 8.94E-06 | up |
| Mmp28 | 1.1534905 | 1.96E-06 | 9.11E-06 | up |
| Prune2 | 1.1258527 | 1.96E-06 | 9.11E-06 | up |
| Myom1 | 1.9685554 | 1.98E-06 | 9.22E-06 | up |
| S100g | 3.1332625 | 2.08E-06 | 9.66E-06 | up |
| Tmem45a | 1.3101869 | 2.09E-06 | 9.71E-06 | up |
| Nnmt | 1.6299951 | 2.09E-06 | 9.72E-06 | up |
| Robo1 | 1.344085 | 2.10E-06 | 9.73E-06 | up |
| Mustn1 | 1.6755409 | 2.10E-06 | 9.74E-06 | up |
| Rnf207 | 2.1100355 | 2.14E-06 | 9.90E-06 | up |
| Vtcn1 | 2.2435778 | 2.23E-06 | 1.03E-05 | up |
| Gm1332 | 3.50478 | 2.24E-06 | 1.03E-05 | up |
| Bmp3 | 2.1249422 | 2.25E-06 | 1.04E-05 | up |
| Paqr5 | 1.3501089 | 2.27E-06 | 1.05E-05 | up |
| Ppapdc3 | 2.1126934 | 2.29E-06 | 1.06E-05 | up |
| Lgr5 | 2.2441143 | 2.30E-06 | 1.06E-05 | up |
| Sfrp1 | 1.2996192 | 2.33E-06 | 1.07E-05 | up |
| Adamtsl3 | 1.272629 | 2.33E-06 | 1.07E-05 | up |
| Pag1 | 1.1049548 | 2.35E-06 | 1.08E-05 | up |
| 9830107B12Rik | 1.9319033 | 2.39E-06 | 1.10E-05 | up |
| Myrip | 1.6919963 | 2.44E-06 | 1.12E-05 | up |
| Rspo1 | 2.098239 | 2.46E-06 | 1.13E-05 | up |
| Fat4 | 1.0286543 | 2.49E-06 | 1.14E-05 | up |
| C7 | 3.0470333 | 2.55E-06 | 1.17E-05 | up |
| Meis1 | 1.0673318 | 2.59E-06 | 1.19E-05 | up |
| Mfsd2a | 2.5392157 | 2.61E-06 | 1.19E-05 | up |
| Myh7 | 9.8829927 | 2.63E-06 | 1.20E-05 | up |
| Lingo1 | 1.8206158 | 2.65E-06 | 1.21E-05 | up |
| Chadl | 1.5226442 | 2.72E-06 | 1.24E-05 | up |
| Pcsk1n | 3.7322001 | 2.76E-06 | 1.26E-05 | up |
| Elovl3 | 4.2856838 | 2.87E-06 | 1.30E-05 | up |
| Cd274 | 1.1806965 | 2.89E-06 | 1.31E-05 | up |
| Itih1 | 2.4504157 | 2.90E-06 | 1.31E-05 | up |
| Kcnd1 | 1.8638848 | 3.02E-06 | 1.36E-05 | up |
| Fbln7 | 1.2213425 | 3.06E-06 | 1.38E-05 | up |
| Wbscr17 | 1.6644899 | 3.13E-06 | 1.41E-05 | up |
| Kif19a | 1.9741624 | 3.16E-06 | 1.42E-05 | up |
| Crispld1 | 1.5902937 | 3.17E-06 | 1.43E-05 | up |
| Tmsb15a | 2.7739557 | 3.17E-06 | 1.43E-05 | up |
| Gm5141 | 1.2264792 | 3.18E-06 | 1.43E-05 | up |
| Lax1 | 2.2984724 | 3.24E-06 | 1.46E-05 | up |
| Plekha4 | 1.2694898 | 3.27E-06 | 1.47E-05 | up |
| Rhov | 1.5449206 | 3.38E-06 | 1.51E-05 | up |
| Upk2 | 2.3824493 | 3.51E-06 | 1.57E-05 | up |
| Prrx2 | 1.2108483 | 3.53E-06 | 1.58E-05 | up |
| Fam129a | 1.071494 | 3.55E-06 | 1.59E-05 | up |
| Klra2 | 2.0981997 | 3.62E-06 | 1.61E-05 | up |
| I830012O16Rik | 1.1603 | 3.77E-06 | 1.68E-05 | up |
| Col24a1 | 2.5649547 | 3.83E-06 | 1.70E-05 | up |
| Cyp26b1 | 2.0394802 | 3.85E-06 | 1.71E-05 | up |
| Mybpc2 | 2.4102808 | 3.89E-06 | 1.73E-05 | up |
| Cyp4f18 | 2.6234827 | 3.92E-06 | 1.74E-05 | up |
| H2-Q2 | 1.510264 | 3.98E-06 | 1.77E-05 | up |
| Gas1 | 1.0509214 | 4.03E-06 | 1.79E-05 | up |
| Six1 | 1.9420722 | 4.04E-06 | 1.79E-05 | up |
| Dnahc10 | 1.3041534 | 4.13E-06 | 1.83E-05 | up |
| Vnn3 | 3.0687951 | 4.18E-06 | 1.85E-05 | up |
| Melk | 1.0463494 | 4.29E-06 | 1.89E-05 | up |
| Zbp1 | 1.9123562 | 4.50E-06 | 1.98E-05 | up |
| Scg2 | 2.8911591 | 4.52E-06 | 1.99E-05 | up |
| Tns4 | 1.6751289 | 4.60E-06 | 2.02E-05 | up |
| Il2rb | 2.2159686 | 4.61E-06 | 2.03E-05 | up |
| 4833403I15Rik | 2.0927194 | 4.63E-06 | 2.03E-05 | up |
| Msx1 | 1.404002 | 4.79E-06 | 2.10E-05 | up |
| Cenpa | 1.017305 | 4.85E-06 | 2.12E-05 | up |
| Ly96 | 1.0974238 | 4.95E-06 | 2.16E-05 | up |
| Ly6g6c | 2.7680969 | 5.11E-06 | 2.23E-05 | up |
| Muc5b | 3.9863538 | 5.12E-06 | 2.23E-05 | up |
| Mybl1 | 1.2682963 | 5.20E-06 | 2.26E-05 | up |
| Xlr | 1.2628076 | 5.30E-06 | 2.31E-05 | up |
| Igsf21 | 2.4133285 | 5.35E-06 | 2.33E-05 | up |
| Filip1l | 1.0208991 | 5.38E-06 | 2.34E-05 | up |
| Tdrkh | 1.6932566 | 5.42E-06 | 2.35E-05 | up |
| Dok7 | 2.7445799 | 5.53E-06 | 2.40E-05 | up |
| Slc38a4 | 2.037697 | 5.59E-06 | 2.42E-05 | up |
| Prdm5 | 1.1384921 | 5.63E-06 | 2.44E-05 | up |
| Fgf13 | 2.6949801 | 5.65E-06 | 2.44E-05 | up |
| Igdcc4 | 1.9599465 | 5.65E-06 | 2.45E-05 | up |
| Bst2 | 1.0049192 | 5.65E-06 | 2.45E-05 | up |
| Socs3 | 1.0288457 | 5.67E-06 | 2.45E-05 | up |
| Cited1 | 1.7002635 | 5.73E-06 | 2.47E-05 | up |
| Npy | 2.2341972 | 5.84E-06 | 2.52E-05 | up |
| Cyb5r2 | 2.1260162 | 6.02E-06 | 2.59E-05 | up |
| Slc4a11 | 1.9288301 | 6.24E-06 | 2.67E-05 | up |
| Tpm2 | 2.9705316 | 6.44E-06 | 2.75E-05 | up |
| Wnt11 | 1.4361717 | 6.53E-06 | 2.79E-05 | up |
| Olr1 | 2.7915109 | 6.55E-06 | 2.80E-05 | up |
| Rdh9 | 1.7205369 | 6.66E-06 | 2.84E-05 | up |
| Nav3 | 1.6152007 | 6.67E-06 | 2.84E-05 | up |
| Art4 | 2.0718412 | 7.08E-06 | 3.01E-05 | up |
| Dkk3 | 1.0075483 | 7.25E-06 | 3.07E-05 | up |
| Arg1 | 2.976758 | 7.44E-06 | 3.15E-05 | up |
| Ank1 | 2.5182273 | 7.49E-06 | 3.17E-05 | up |
| Rhebl1 | 1.0973195 | 7.59E-06 | 3.21E-05 | up |
| Cd2 | 2.5132454 | 7.62E-06 | 3.22E-05 | up |
| Cenpw | 1.0494925 | 7.69E-06 | 3.24E-05 | up |
| 4931408D14Rik | 1.1750257 | 7.71E-06 | 3.25E-05 | up |
| Adamtsl5 | 1.0846617 | 7.74E-06 | 3.26E-05 | up |
| Zfp783 | 1.9738636 | 7.85E-06 | 3.31E-05 | up |
| Arhgef39 | 1.148102 | 7.91E-06 | 3.33E-05 | up |
| Slc43a1 | 1.8362155 | 7.92E-06 | 3.33E-05 | up |
| Tmod2 | 1.104159 | 7.99E-06 | 3.36E-05 | up |
| Ptgfr | 1.8648077 | 8.01E-06 | 3.37E-05 | up |
| Tstd3 | 1.0109365 | 8.11E-06 | 3.41E-05 | up |
| Scg3 | 2.1156087 | 8.11E-06 | 3.41E-05 | up |
| Tmem140 | 1.0191286 | 8.22E-06 | 3.45E-05 | up |
| Amy1 | 1.632912 | 8.62E-06 | 3.61E-05 | up |
| Nsg1 | 1.0365573 | 8.65E-06 | 3.62E-05 | up |
| Mreg | 2.0813444 | 8.68E-06 | 3.63E-05 | up |
| Scrn1 | 1.5639258 | 8.68E-06 | 3.63E-05 | up |
| Cd209d | 2.1710858 | 8.76E-06 | 3.66E-05 | up |
| Gbp11 | 2.3596964 | 8.96E-06 | 3.74E-05 | up |
| Btbd19 | 1.0375838 | 9.07E-06 | 3.78E-05 | up |
| Gtse1 | 1.0009404 | 9.33E-06 | 3.89E-05 | up |
| Omp | 1.4234872 | 9.58E-06 | 3.99E-05 | up |
| Cftr | 1.9842773 | 9.98E-06 | 4.14E-05 | up |
| Flrt2 | 1.0668676 | 1.00E-05 | 4.15E-05 | up |
| Nsg2 | 3.4471949 | 1.01E-05 | 4.19E-05 | up |
| C4b | 1.079846 | 1.03E-05 | 4.27E-05 | up |
| Myl2 | 6.1063296 | 1.03E-05 | 4.28E-05 | up |
| Ppp1r3b | 1.8707756 | 1.05E-05 | 4.34E-05 | up |
| Cnn1 | 1.730177 | 1.06E-05 | 4.36E-05 | up |
| B3gnt3 | 1.4924593 | 1.06E-05 | 4.38E-05 | up |
| Nmnat2 | 2.0172012 | 1.06E-05 | 4.38E-05 | up |
| Gxylt2 | 1.713051 | 1.07E-05 | 4.41E-05 | up |
| Cyr61 | 1.1708949 | 1.07E-05 | 4.42E-05 | up |
| Gpr113 | 2.4325152 | 1.08E-05 | 4.43E-05 | up |
| Scn3b | 1.1199077 | 1.09E-05 | 4.47E-05 | up |
| Ccrl1 | 1.1943542 | 1.10E-05 | 4.54E-05 | up |
| 1110046J04Rik | 1.1825252 | 1.11E-05 | 4.56E-05 | up |
| Klhl30 | 2.6161022 | 1.11E-05 | 4.57E-05 | up |
| Ror2 | 1.4207952 | 1.13E-05 | 4.63E-05 | up |
| Osr2 | 1.4896319 | 1.13E-05 | 4.63E-05 | up |
| Tmem202 | 1.3707969 | 1.14E-05 | 4.67E-05 | up |
| Bhlhe22 | 1.8651902 | 1.14E-05 | 4.68E-05 | up |
| Calml4 | 1.2920322 | 1.15E-05 | 4.69E-05 | up |
| Actg2 | 1.8533373 | 1.17E-05 | 4.78E-05 | up |
| B3gnt5 | 1.2120472 | 1.17E-05 | 4.80E-05 | up |
| Pck1 | 2.4640391 | 1.21E-05 | 4.95E-05 | up |
| Kazald1 | 1.2525787 | 1.27E-05 | 5.15E-05 | up |
| Sostdc1 | 1.380215 | 1.29E-05 | 5.24E-05 | up |
| Btg2 | 1.0163931 | 1.30E-05 | 5.25E-05 | up |
| Barx1 | 2.1812228 | 1.31E-05 | 5.32E-05 | up |
| Tmem220 | 1.1960749 | 1.32E-05 | 5.34E-05 | up |
| Adap2 | 1.0295937 | 1.33E-05 | 5.39E-05 | up |
| Tmprss4 | 2.6449186 | 1.35E-05 | 5.47E-05 | up |
| Grrp1 | 1.0799337 | 1.41E-05 | 5.67E-05 | up |
| Gdf10 | 1.3839414 | 1.42E-05 | 5.70E-05 | up |
| Fam78a | 1.6254758 | 1.43E-05 | 5.76E-05 | up |
| Bbox1 | 2.1731816 | 1.43E-05 | 5.76E-05 | up |
| Dsc3 | 3.2562866 | 1.44E-05 | 5.77E-05 | up |
| Abca6 | 1.8624242 | 1.44E-05 | 5.77E-05 | up |
| Fbxo27 | 1.3010736 | 1.49E-05 | 5.96E-05 | up |
| Trim67 | 1.3192272 | 1.49E-05 | 5.99E-05 | up |
| D10Bwg1379e | 2.2007013 | 1.51E-05 | 6.05E-05 | up |
| Map6 | 1.0977335 | 1.52E-05 | 6.07E-05 | up |
| Rasd2 | 1.6007026 | 1.52E-05 | 6.08E-05 | up |
| Cxxc4 | 1.4093145 | 1.53E-05 | 6.13E-05 | up |
| Fgf10 | 1.6238118 | 1.55E-05 | 6.20E-05 | up |
| Hrh1 | 1.8916732 | 1.57E-05 | 6.26E-05 | up |
| Mgat3 | 1.0883034 | 1.61E-05 | 6.40E-05 | up |
| Cyp7b1 | 1.1933251 | 1.62E-05 | 6.45E-05 | up |
| Hmga1 | 1.2708306 | 1.64E-05 | 6.52E-05 | up |
| Il1rl1 | 1.9483419 | 1.65E-05 | 6.54E-05 | up |
| Sbk2 | 4.5840845 | 1.66E-05 | 6.57E-05 | up |
| Gata6 | 1.1891719 | 1.68E-05 | 6.65E-05 | up |
| Nlrc5 | 1.25401 | 1.71E-05 | 6.76E-05 | up |
| P2ry12 | 1.0877275 | 1.82E-05 | 7.13E-05 | up |
| Cfd | 2.0384418 | 1.84E-05 | 7.22E-05 | up |
| Gpr114 | 1.2987265 | 1.84E-05 | 7.23E-05 | up |
| Gm5424 | 1.5747019 | 1.88E-05 | 7.37E-05 | up |
| Tnfsf13b | 1.2052365 | 1.90E-05 | 7.42E-05 | up |
| Tmem245 | 1.1316757 | 1.90E-05 | 7.44E-05 | up |
| Fmo3 | 3.2614642 | 1.90E-05 | 7.44E-05 | up |
| Car8 | 1.2686929 | 1.92E-05 | 7.51E-05 | up |
| Rtn2 | 1.7405801 | 1.94E-05 | 7.58E-05 | up |
| Diras2 | 2.3379822 | 1.97E-05 | 7.68E-05 | up |
| Stx19 | 1.9206813 | 1.99E-05 | 7.76E-05 | up |
| Birc5 | 1.0231284 | 2.00E-05 | 7.79E-05 | up |
| Adamts12 | 1.1443492 | 2.02E-05 | 7.85E-05 | up |
| Slc16a3 | 1.6148381 | 2.04E-05 | 7.91E-05 | up |
| Pfkfb1 | 1.4208045 | 2.04E-05 | 7.91E-05 | up |
| Bend6 | 1.4703302 | 2.04E-05 | 7.92E-05 | up |
| H2-Q10 | 1.6518659 | 2.15E-05 | 8.33E-05 | up |
| Acot5 | 2.4193039 | 2.19E-05 | 8.47E-05 | up |
| Ifi47 | 1.0890314 | 2.22E-05 | 8.57E-05 | up |
| Pdzd4 | 1.23772 | 2.25E-05 | 8.67E-05 | up |
| Cdsn | 1.4415594 | 2.29E-05 | 8.80E-05 | up |
| B430212C06Rik | 1.7292608 | 2.31E-05 | 8.87E-05 | up |
| Piwil4 | 1.9032703 | 2.39E-05 | 9.17E-05 | up |
| Sgcd | 1.9482521 | 2.44E-05 | 9.36E-05 | up |
| Tlcd2 | 1.1997843 | 2.55E-05 | 9.74E-05 | up |
| Rnase1 | 1.9074378 | 2.59E-05 | 9.89E-05 | up |
| Fam132b | 1.3377093 | 2.63E-05 | 0.0001 | up |
| Ndrg2 | 1.9942852 | 2.67E-05 | 0.0001014 | up |
| Hoxb7 | 2.5354032 | 2.67E-05 | 0.0001015 | up |
| Cd207 | 2.7186322 | 2.69E-05 | 0.0001024 | up |
| Abcc3 | 1.1375996 | 2.72E-05 | 0.0001034 | up |
| Sprr2b | 4.6236936 | 2.74E-05 | 0.0001039 | up |
| Zfp934 | 1.1134782 | 2.77E-05 | 0.000105 | up |
| Hs6st2 | 1.4109267 | 2.85E-05 | 0.0001077 | up |
| Fut2 | 1.7966562 | 2.85E-05 | 0.0001079 | up |
| Atp1a2 | 1.8093078 | 2.86E-05 | 0.0001079 | up |
| Asphd2 | 1.7480882 | 2.86E-05 | 0.000108 | up |
| Akap6 | 1.7405963 | 2.87E-05 | 0.0001083 | up |
| Ifi27l2b | 2.6577023 | 2.92E-05 | 0.0001101 | up |
| Lep | 2.7762286 | 2.94E-05 | 0.0001106 | up |
| AA986860 | 1.0717484 | 2.98E-05 | 0.0001123 | up |
| Fam20a | 1.0640364 | 2.99E-05 | 0.0001124 | up |
| Fam171b | 1.9534075 | 3.00E-05 | 0.0001129 | up |
| Abcb1a | 1.285954 | 3.17E-05 | 0.0001191 | up |
| Rbm24 | 3.795114 | 3.24E-05 | 0.0001212 | up |
| Serpinb12 | 3.3860616 | 3.31E-05 | 0.0001237 | up |
| 5031414D18Rik | 1.5330276 | 3.34E-05 | 0.0001248 | up |
| 1810041L15Rik | 1.0871871 | 3.40E-05 | 0.000127 | up |
| Gulp1 | 1.1160934 | 3.56E-05 | 0.0001321 | up |
| Sec16b | 1.0942205 | 3.63E-05 | 0.0001346 | up |
| Ccl12 | 1.3797973 | 3.63E-05 | 0.0001347 | up |
| Ereg | 2.2108067 | 3.82E-05 | 0.0001411 | up |
| Grap2 | 1.9122138 | 3.85E-05 | 0.000142 | up |
| Chp2 | 1.6461009 | 3.86E-05 | 0.0001425 | up |
| Cytl1 | 2.4533513 | 3.88E-05 | 0.0001432 | up |
| Cxcl9 | 2.4447736 | 3.91E-05 | 0.0001441 | up |
| Serpina3f | 1.6384223 | 3.96E-05 | 0.0001457 | up |
| Spint4 | 1.5674432 | 3.99E-05 | 0.0001469 | up |
| Fam109b | 1.2977883 | 4.06E-05 | 0.0001494 | up |
| Cd1d1 | 1.2482931 | 4.07E-05 | 0.0001496 | up |
| Slc9a2 | 1.8381196 | 4.11E-05 | 0.0001512 | up |
| Serpinb10 | 3.0425921 | 4.21E-05 | 0.0001544 | up |
| Siglech | 1.663116 | 4.39E-05 | 0.0001601 | up |
| Adam28 | 2.4611404 | 4.65E-05 | 0.000169 | up |
| Srrm4 | 2.4111353 | 4.66E-05 | 0.0001693 | up |
| Mrap | 2.1120663 | 4.70E-05 | 0.0001708 | up |
| Nlrp3 | 1.1347494 | 4.77E-05 | 0.0001731 | up |
| Dnase1l3 | 2.1826785 | 4.82E-05 | 0.0001747 | up |
| Gpr123 | 1.9079014 | 4.99E-05 | 0.0001807 | up |
| Flt3l | 1.0716082 | 5.11E-05 | 0.0001847 | up |
| BC096441 | 1.3245362 | 5.18E-05 | 0.0001869 | up |
| Dleu2 | 1.0284977 | 5.40E-05 | 0.0001939 | up |
| Ascl1 | 2.9512107 | 5.41E-05 | 0.0001942 | up |
| 4833422C13Rik | 1.4144202 | 5.42E-05 | 0.0001946 | up |
| Abcc8 | 2.4810131 | 5.49E-05 | 0.0001968 | up |
| 4833423E24Rik | 4.0974811 | 5.51E-05 | 0.0001973 | up |
| Ctse | 1.6379011 | 5.55E-05 | 0.0001986 | up |
| Stat4 | 2.1298119 | 5.76E-05 | 0.0002056 | up |
| C1qtnf3 | 1.7228009 | 5.98E-05 | 0.0002129 | up |
| Cd4 | 2.141399 | 6.00E-05 | 0.0002134 | up |
| Spn | 1.4146276 | 6.19E-05 | 0.0002193 | up |
| Enox1 | 1.4237127 | 6.27E-05 | 0.000222 | up |
| Gsta3 | 1.2919668 | 6.28E-05 | 0.0002222 | up |
| Gpr161 | 1.6083635 | 6.48E-05 | 0.0002289 | up |
| Msc | 1.5509643 | 6.48E-05 | 0.000229 | up |
| Acap1 | 2.3546374 | 6.52E-05 | 0.0002301 | up |
| Padi4 | 2.2876741 | 6.54E-05 | 0.0002308 | up |
| Lrrc17 | 1.3403259 | 6.65E-05 | 0.0002343 | up |
| Gab3 | 1.4662075 | 6.98E-05 | 0.0002452 | up |
| Srpk3 | 1.9148861 | 7.01E-05 | 0.000246 | up |
| Lgi3 | 1.1227806 | 7.04E-05 | 0.0002471 | up |
| Pard6g | 1.2435368 | 7.22E-05 | 0.0002531 | up |
| 2310001H17Rik | 1.541542 | 7.31E-05 | 0.0002559 | up |
| Sp140 | 1.0030323 | 7.62E-05 | 0.0002659 | up |
| Nkx6-2 | 3.0798969 | 7.65E-05 | 0.0002668 | up |
| Bmper | 1.0227173 | 7.74E-05 | 0.0002696 | up |
| Hdac9 | 1.753789 | 7.85E-05 | 0.000273 | up |
| Slc28a3 | 1.2852083 | 7.96E-05 | 0.0002761 | up |
| Shc4 | 1.1523544 | 8.27E-05 | 0.0002861 | up |
| Muc4 | 2.4652811 | 8.29E-05 | 0.0002867 | up |
| Slc39a8 | 1.3850417 | 8.34E-05 | 0.0002882 | up |
| Zfp820 | 1.0736022 | 8.41E-05 | 0.0002903 | up |
| Slc25a43 | 1.2738353 | 8.84E-05 | 0.0003042 | up |
| Tnni1 | 4.6109622 | 8.97E-05 | 0.0003085 | up |
| Tcp11 | 1.506932 | 9.14E-05 | 0.0003137 | up |
| Rimkla | 1.662381 | 9.21E-05 | 0.000316 | up |
| Slc30a2 | 2.7194153 | 9.35E-05 | 0.0003202 | up |
| Angptl7 | 1.8246519 | 9.42E-05 | 0.0003223 | up |
| Camk2b | 2.6562194 | 9.61E-05 | 0.0003283 | up |
| Mcoln3 | 3.7165804 | 9.62E-05 | 0.0003286 | up |
| Wnk4 | 1.1475697 | 0.0001018 | 0.0003462 | up |
| Il1b | 2.1838232 | 0.0001025 | 0.0003484 | up |
| Kcnma1 | 1.8496836 | 0.0001053 | 0.0003571 | up |
| Fmod | 1.2331729 | 0.0001058 | 0.0003585 | up |
| Trim12a | 4.5516012 | 0.0001078 | 0.0003648 | up |
| Extl1 | 1.8484713 | 0.000109 | 0.0003686 | up |
| Klf8 | 1.3693127 | 0.0001101 | 0.0003722 | up |
| Creb3l1 | 1.1896462 | 0.0001135 | 0.0003825 | up |
| 9330159F19Rik | 2.4926396 | 0.0001139 | 0.0003834 | up |
| Nfkbid | 1.0349955 | 0.0001153 | 0.0003881 | up |
| S100a8 | 2.3245946 | 0.0001192 | 0.0004006 | up |
| Fhl3 | 1.4333636 | 0.0001194 | 0.0004009 | up |
| Il2ra | 1.2902251 | 0.0001213 | 0.0004068 | up |
| Epha3 | 1.6084538 | 0.0001224 | 0.0004101 | up |
| Shcbp1 | 1.0271869 | 0.0001234 | 0.0004131 | up |
| Has2 | 1.5877819 | 0.0001258 | 0.0004203 | up |
| Rbp1 | 1.1480109 | 0.000126 | 0.0004207 | up |
| Pvalb | 4.3156472 | 0.0001262 | 0.0004214 | up |
| Nexn | 1.4991726 | 0.0001269 | 0.0004238 | up |
| Chst10 | 1.8698774 | 0.0001284 | 0.000428 | up |
| Stap1 | 1.713955 | 0.0001299 | 0.0004324 | up |
| Speg | 1.5010412 | 0.00013 | 0.0004326 | up |
| Pgam2 | 2.8926542 | 0.0001345 | 0.0004464 | up |
| Junb | 1.004965 | 0.0001353 | 0.0004487 | up |
| Scn2a1 | 1.4124818 | 0.0001365 | 0.0004523 | up |
| Mtl5 | 1.4658145 | 0.0001411 | 0.0004662 | up |
| Htra4 | 2.0811209 | 0.0001435 | 0.0004737 | up |
| Orc1 | 1.0662654 | 0.0001438 | 0.0004744 | up |
| Dnaic2 | 2.8514177 | 0.0001466 | 0.0004831 | up |
| Thsd7a | 1.311504 | 0.000147 | 0.0004843 | up |
| Hoxa3 | 1.2933872 | 0.0001496 | 0.0004922 | up |
| Xpnpep2 | 1.5448453 | 0.0001543 | 0.0005061 | up |
| Cntfr | 2.2424599 | 0.0001552 | 0.0005088 | up |
| Car11 | 1.2052631 | 0.0001577 | 0.0005166 | up |
| Slc2a4 | 1.9639334 | 0.0001601 | 0.0005237 | up |
| Gimap3 | 1.6160025 | 0.0001603 | 0.0005241 | up |
| Col23a1 | 1.8789925 | 0.0001625 | 0.000531 | up |
| Wdr52 | 2.6164313 | 0.0001633 | 0.0005334 | up |
| Cym | 3.1635343 | 0.0001642 | 0.0005358 | up |
| Htr1d | 3.2527956 | 0.0001717 | 0.0005576 | up |
| Fez1 | 1.5382528 | 0.0001749 | 0.000567 | up |
| Ky | 3.1767908 | 0.0001765 | 0.0005719 | up |
| Hspb6 | 1.3435186 | 0.0001772 | 0.0005736 | up |
| Chgb | 2.5783651 | 0.0001802 | 0.0005828 | up |
| Itgb1bp2 | 3.7535103 | 0.0001833 | 0.0005917 | up |
| Rad54b | 1.0068503 | 0.0001848 | 0.0005959 | up |
| Kcnj11 | 3.6363961 | 0.0001851 | 0.0005966 | up |
| Dok5 | 1.6599686 | 0.0001912 | 0.0006144 | up |
| Fancd2 | 1.0204583 | 0.0001917 | 0.0006157 | up |
| Gm14492 | 1.345386 | 0.0001927 | 0.0006188 | up |
| Gbgt1 | 1.4331213 | 0.0001961 | 0.0006287 | up |
| Tox | 2.578754 | 0.0002015 | 0.0006446 | up |
| Pcp4l1 | 1.195089 | 0.000204 | 0.0006518 | up |
| Mylk4 | 3.8608335 | 0.000207 | 0.0006602 | up |
| Bpifa1 | 3.9343155 | 0.0002088 | 0.0006652 | up |
| 0610040B10Rik | 1.3120801 | 0.0002103 | 0.0006695 | up |
| Prkg1 | 1.038449 | 0.0002167 | 0.000688 | up |
| Tmem212 | 3.577162 | 0.0002183 | 0.000692 | up |
| Scn4b | 2.3713607 | 0.0002201 | 0.0006973 | up |
| Gpc3 | 1.1852502 | 0.0002203 | 0.0006975 | up |
| Muc20 | 1.0680592 | 0.0002225 | 0.0007039 | up |
| Cilp | 1.2037201 | 0.0002233 | 0.0007061 | up |
| Kcne4 | 1.1927373 | 0.0002238 | 0.0007075 | up |
| Gcgr | 1.3071536 | 0.0002307 | 0.0007273 | up |
| Fam217a | 1.7084858 | 0.0002317 | 0.00073 | up |
| Hsd11b1 | 1.015583 | 0.000233 | 0.0007336 | up |
| Fcer2a | 1.2843863 | 0.0002348 | 0.0007387 | up |
| Atf3 | 1.2575181 | 0.0002348 | 0.0007387 | up |
| Jph2 | 1.8859563 | 0.0002361 | 0.0007414 | up |
| Slc23a3 | 1.3598818 | 0.0002392 | 0.0007502 | up |
| Tcap | 3.8857303 | 0.0002454 | 0.0007681 | up |
| Bcl2a1a | 1.4919317 | 0.0002533 | 0.0007918 | up |
| Zfp382 | 1.3176291 | 0.0002555 | 0.0007982 | up |
| Fibin | 1.091368 | 0.0002584 | 0.0008066 | up |
| Fam180a | 1.3577433 | 0.000262 | 0.0008164 | up |
| Uts2 | 1.9277584 | 0.000263 | 0.000819 | up |
| Hoxb3 | 1.0523948 | 0.0002653 | 0.0008256 | up |
| Mst1r | 1.1231238 | 0.0002707 | 0.0008417 | up |
| Stra6 | 1.6831229 | 0.0002771 | 0.0008607 | up |
| Cd3d | 2.274264 | 0.0002994 | 0.0009245 | up |
| Prr7 | 1.112524 | 0.0003001 | 0.0009263 | up |
| Slco5a1 | 2.3520712 | 0.0003099 | 0.0009534 | up |
| Trim55 | 3.4095478 | 0.000313 | 0.0009623 | up |
| AI593442 | 2.0757173 | 0.0003132 | 0.0009629 | up |
| Mlf1 | 2.0193983 | 0.0003188 | 0.000978 | up |
| Dusp27 | 2.098213 | 0.0003279 | 0.0010028 | up |
| Cyp2a5 | 3.1451604 | 0.0003453 | 0.001052 | up |
| Trpm2 | 1.111374 | 0.0003531 | 0.0010736 | up |
| Cyp2b10 | 3.2563582 | 0.0003577 | 0.0010862 | up |
| Chsy3 | 1.3774614 | 0.0003647 | 0.0011048 | up |
| Adrb3 | 2.2295498 | 0.0003668 | 0.0011105 | up |
| Akap5 | 1.0273396 | 0.0003669 | 0.0011105 | up |
| Gm5480 | 1.1286309 | 0.0003699 | 0.001119 | up |
| Pygo1 | 1.6250932 | 0.0003724 | 0.0011248 | up |
| Ptprcap | 2.3578782 | 0.0003765 | 0.001136 | up |
| Sh2d2a | 2.4312806 | 0.0003772 | 0.0011375 | up |
| Cd72 | 1.0163815 | 0.0003797 | 0.0011445 | up |
| Snap25 | 2.5733676 | 0.0003833 | 0.0011549 | up |
| Myl3 | 5.6630325 | 0.000391 | 0.0011745 | up |
| Gpr182 | 1.1567168 | 0.000399 | 0.001196 | up |
| Bfsp1 | 1.2855477 | 0.0004038 | 0.0012084 | up |
| Emilin3 | 1.8918952 | 0.0004059 | 0.0012137 | up |
| Gm684 | 1.2405535 | 0.0004084 | 0.0012203 | up |
| Sgca | 3.8987923 | 0.0004137 | 0.0012349 | up |
| Tmeff2 | 1.2043512 | 0.0004255 | 0.0012683 | up |
| 2310042D19Rik | 1.4364981 | 0.0004286 | 0.0012771 | up |
| Snord17 | 3.979065 | 0.0004328 | 0.0012881 | up |
| Gsc | 1.4938019 | 0.0004362 | 0.0012968 | up |
| Nrip3 | 1.2848279 | 0.0004373 | 0.0012993 | up |
| Gna14 | 1.5088711 | 0.0004461 | 0.0013231 | up |
| Upk1a | 1.3597832 | 0.0004469 | 0.0013253 | up |
| Zfhx4 | 1.3526571 | 0.0004594 | 0.0013588 | up |
| Ttn | 3.421231 | 0.0004618 | 0.0013651 | up |
| Ndrg4 | 1.2356099 | 0.0004737 | 0.0013985 | up |
| Rpl3l | 2.7766156 | 0.00048 | 0.0014156 | up |
| Pkp1 | 1.6092955 | 0.0004845 | 0.0014284 | up |
| Dnahc7b | 1.3381487 | 0.0005032 | 0.0014799 | up |
| Coro6 | 2.176753 | 0.0005044 | 0.001483 | up |
| Cdh3 | 1.8339367 | 0.0005174 | 0.0015198 | up |
| Bcl2a1d | 1.4269674 | 0.0005291 | 0.0015508 | up |
| Folh1 | 1.4710731 | 0.0005347 | 0.0015665 | up |
| Dhrs7c | 3.6315758 | 0.000564 | 0.0016437 | up |
| 3-Sep | 1.4900086 | 0.0005685 | 0.0016554 | up |
| Prdm8 | 1.4450705 | 0.0005693 | 0.0016573 | up |
| Angptl3 | 1.1927027 | 0.0005833 | 0.0016955 | up |
| Avil | 1.0633454 | 0.0005842 | 0.0016976 | up |
| Hk2 | 1.0780646 | 0.0005942 | 0.0017243 | up |
| Kndc1 | 1.4539981 | 0.0005965 | 0.0017301 | up |
| Skap1 | 2.1509709 | 0.0006007 | 0.001741 | up |
| Ptprz1 | 2.4331165 | 0.0006057 | 0.0017542 | up |
| Tfpi2 | 1.1922723 | 0.0006131 | 0.0017741 | up |
| 1-Mar | 1.2078982 | 0.0006169 | 0.0017845 | up |
| Serpinb5 | 2.0227158 | 0.0006236 | 0.0018017 | up |
| Pcdhb16 | 1.1891193 | 0.0006323 | 0.0018254 | up |
| Lypd2 | 1.3311683 | 0.0006409 | 0.0018484 | up |
| Hspb3 | 3.1287564 | 0.0006487 | 0.0018686 | up |
| Vgll2 | 3.9140047 | 0.0006577 | 0.0018917 | up |
| Ankrd35 | 1.4695096 | 0.000661 | 0.0018999 | up |
| Npy1r | 1.3956503 | 0.0006868 | 0.0019681 | up |
| Slc6a4 | 1.1241355 | 0.0006897 | 0.0019759 | up |
| Cyp4a12b | 3.6743256 | 0.0006953 | 0.0019894 | up |
| Prkag3 | 2.4617245 | 0.0006981 | 0.0019967 | up |
| Pcdhb7 | 1.0551762 | 0.000702 | 0.002007 | up |
| Resp18 | 2.4676126 | 0.0007094 | 0.0020244 | up |
| Csmd1 | 1.4083723 | 0.0007094 | 0.0020244 | up |
| Mill2 | 1.2956262 | 0.00071 | 0.0020256 | up |
| Lgi2 | 1.6900809 | 0.000715 | 0.0020382 | up |
| 1700020L24Rik | 1.0908029 | 0.0007185 | 0.002047 | up |
| Gal3st3 | 1.2904658 | 0.0007366 | 0.0020964 | up |
| Dnahc2 | 2.0091457 | 0.0007368 | 0.0020967 | up |
| Mtmr7 | 1.3743499 | 0.0007414 | 0.0021081 | up |
| Cd80 | 1.0961277 | 0.0007436 | 0.0021139 | up |
| Tc2n | 1.1384104 | 0.0007439 | 0.0021145 | up |
| 2810029C07Rik | 1.1570591 | 0.0007451 | 0.002117 | up |
| 6330403A02Rik | 1.1823066 | 0.0007528 | 0.0021356 | up |
| Ces2c | 1.7566597 | 0.0007724 | 0.0021856 | up |
| Angptl1 | 1.3290747 | 0.0007888 | 0.0022299 | up |
| Lef1 | 1.8109585 | 0.0007893 | 0.0022306 | up |
| Klhl33 | 3.04885 | 0.0008099 | 0.0022854 | up |
| Ldb3 | 3.0727398 | 0.0008117 | 0.0022902 | up |
| Tbx1 | 2.2168483 | 0.0008204 | 0.0023122 | up |
| H2-Ob | 1.1518059 | 0.0008387 | 0.0023602 | up |
| Mxd3 | 1.0042101 | 0.0008459 | 0.0023759 | up |
| P2rx6 | 1.0728153 | 0.0008672 | 0.0024282 | up |
| Scube2 | 1.2883196 | 0.0008762 | 0.0024502 | up |
| Casq1 | 3.1291918 | 0.0008772 | 0.002452 | up |
| Itga2 | 1.3983573 | 0.0008897 | 0.0024821 | up |
| Hrc | 2.9844006 | 0.0009042 | 0.0025177 | up |
| Hesx1 | 1.2544187 | 0.0009113 | 0.002536 | up |
| Art5 | 2.9853638 | 0.0009117 | 0.0025366 | up |
| Syn1 | 1.3118495 | 0.000934 | 0.0025921 | up |
| Jsrp1 | 3.0502061 | 0.0009416 | 0.0026117 | up |
| Twist2 | 1.2123156 | 0.0009455 | 0.0026217 | up |
| Gbp6 | 1.2167573 | 0.0009546 | 0.0026434 | up |
| 2310065F04Rik | 4.0731218 | 0.0009552 | 0.0026444 | up |
| Actn3 | 3.1999292 | 0.0009598 | 0.0026566 | up |
| Nxpe3 | 1.0503405 | 0.0010096 | 0.0027808 | up |
| Slc8a3 | 2.2643118 | 0.0010221 | 0.002812 | up |
| Ifi205 | 1.1604558 | 0.001039 | 0.0028535 | up |
| Pcsk2 | 2.7054743 | 0.0010414 | 0.0028596 | up |
| Gareml | 1.4742566 | 0.0010429 | 0.0028625 | up |
| Dtna | 1.1884435 | 0.0010678 | 0.0029277 | up |
| Sync | 1.5181092 | 0.0010805 | 0.0029587 | up |
| Wt1 | 1.3153355 | 0.0010928 | 0.0029895 | up |
| Reep1 | 1.2716305 | 0.0011006 | 0.0030066 | up |
| Kcnn3 | 1.8249009 | 0.0011029 | 0.003011 | up |
| Calca | 2.9615728 | 0.0011284 | 0.0030743 | up |
| Kcna7 | 2.2903013 | 0.0011356 | 0.0030908 | up |
| Dusp13 | 2.303892 | 0.0011656 | 0.0031679 | up |
| Tnni2 | 3.2716282 | 0.0011717 | 0.0031826 | up |
| Gpr27 | 1.3944507 | 0.0011887 | 0.0032251 | up |
| Cap2 | 1.9130225 | 0.0012387 | 0.0033427 | up |
| Acsm3 | 1.8388842 | 0.0012722 | 0.0034227 | up |
| Six2 | 1.4691818 | 0.0013049 | 0.0035031 | up |
| Ephx3 | 1.1071856 | 0.001309 | 0.003512 | up |
| Dgkb | 1.9147745 | 0.0013239 | 0.0035476 | up |
| Tbx6 | 1.1787552 | 0.0013269 | 0.0035549 | up |
| Etv4 | 1.0339144 | 0.0014167 | 0.0037668 | up |
| Cilp2 | 1.379325 | 0.0014175 | 0.0037679 | up |
| Sgsm1 | 1.1365862 | 0.0014843 | 0.003923 | up |
| Cd6 | 2.561947 | 0.0015164 | 0.0039996 | up |
| Cmya5 | 2.6886007 | 0.0015606 | 0.0040999 | up |
| Cd3e | 2.6118451 | 0.0015805 | 0.0041455 | up |
| Mylpf | 3.221161 | 0.0015852 | 0.0041539 | up |
| Ckmt2 | 3.8504285 | 0.0015933 | 0.0041729 | up |
| Fitm1 | 3.201878 | 0.0015931 | 0.0041729 | up |
| Capn3 | 2.0991783 | 0.0016409 | 0.0042859 | up |
| Mb | 3.0101849 | 0.0016482 | 0.0043036 | up |
| Fgf14 | 1.5166837 | 0.0016832 | 0.0043855 | up |
| Dnali1 | 2.9292494 | 0.0017026 | 0.0044305 | up |
| Slc22a12 | 1.2752594 | 0.0017394 | 0.0045189 | up |
| Xylt1 | 1.3004783 | 0.0017606 | 0.0045674 | up |
| 2310002L09Rik | 3.4734161 | 0.0017862 | 0.0046214 | up |
| Dnahc5 | 2.9166965 | 0.0017916 | 0.0046331 | up |
| Il12rb2 | 1.1155238 | 0.0018083 | 0.0046729 | up |
| Adamts14 | 1.0970825 | 0.0018232 | 0.0047087 | up |
| Atp2b2 | 1.3581124 | 0.0018431 | 0.0047541 | up |
| Cav3 | 2.4000287 | 0.0018728 | 0.0048239 | up |
| Mettl21e | 3.1235263 | 0.0019212 | 0.0049321 | up |
| 9330182L06Rik | 1.11305 | 0.0019237 | 0.0049375 | up |
| Tceal3 | 1.401095 | 0.00195 | 0.0049957 | up |
| Clcn1 | 2.3110441 | 0.0019782 | 0.0050552 | up |
| Il13ra2 | 1.4226981 | 0.0019829 | 0.0050643 | up |
| Dynlrb2 | 3.1647189 | 0.0019885 | 0.0050751 | up |
| AU040972 | 3.5670888 | 0.001995 | 0.0050908 | up |
| Mypn | 3.5187964 | 0.0019997 | 0.005102 | up |
| Drp2 | 1.4675703 | 0.002049 | 0.0052204 | up |
| Popdc2 | 1.8310443 | 0.0021342 | 0.0054119 | up |
| Maats1 | 1.3509709 | 0.0021567 | 0.0054601 | up |
| Mycl1 | 1.248605 | 0.0022022 | 0.0055648 | up |
| Crhr2 | 2.5104977 | 0.0022045 | 0.0055697 | up |
| Aox3 | 2.1625853 | 0.0022061 | 0.0055727 | up |
| Cldn23 | 1.3466824 | 0.0022276 | 0.0056212 | up |
| 2410004P03Rik | 2.224425 | 0.0022454 | 0.0056602 | up |
| Rpl26 | 1.0356322 | 0.0022462 | 0.0056612 | up |
| Wdr63 | 2.5321134 | 0.0022928 | 0.0057626 | up |
| Nlrc3 | 1.2013699 | 0.0022938 | 0.0057641 | up |
| Hoxa4 | 1.3692654 | 0.002313 | 0.0058084 | up |
| Kcnk7 | 1.084291 | 0.0023228 | 0.0058253 | up |
| 4930486L24Rik | 1.0144182 | 0.0023229 | 0.0058253 | up |
| Bai2 | 1.0552082 | 0.0023576 | 0.0059051 | up |
| Ptger3 | 1.2410408 | 0.0023717 | 0.0059374 | up |
| Pdlim3 | 1.9682413 | 0.0024153 | 0.0060369 | up |
| Tbx15 | 2.2656844 | 0.0024453 | 0.0060993 | up |
| Krt5 | 2.951552 | 0.0024573 | 0.0061241 | up |
| Rgs18 | 1.2991089 | 0.0024793 | 0.0061747 | up |
| Gdpd2 | 1.6819822 | 0.0024872 | 0.0061932 | up |
| Dach1 | 1.1619003 | 0.0025093 | 0.006243 | up |
| Dhh | 1.3332929 | 0.0025568 | 0.0063535 | up |
| Gm19522 | 1.217055 | 0.0025816 | 0.0064031 | up |
| Slc16a5 | 2.7160095 | 0.0026946 | 0.0066559 | up |
| Luzp2 | 1.4817262 | 0.0027322 | 0.0067373 | up |
| Col10a1 | 1.3138365 | 0.0027842 | 0.0068505 | up |
| Oasl1 | 1.0612089 | 0.0028689 | 0.0070433 | up |
| Myoz1 | 3.2182297 | 0.0029346 | 0.0071913 | up |
| H19 | 2.724019 | 0.0029951 | 0.0073272 | up |
| Lipf | 3.0866733 | 0.0030197 | 0.0073774 | up |
| Tnfrsf19 | 1.7723583 | 0.0030317 | 0.0074041 | up |
| Zmynd10 | 1.8760386 | 0.0030518 | 0.0074444 | up |
| Foxg1 | 1.5701486 | 0.0030919 | 0.0075283 | up |
| 3425401B19Rik | 2.7709036 | 0.0031089 | 0.0075672 | up |
| Myh8 | 4.505005 | 0.0031123 | 0.0075742 | up |
| Tmem45b | 1.6623303 | 0.0031173 | 0.0075851 | up |
| Lmod3 | 3.110836 | 0.0031522 | 0.0076597 | up |
| Trim72 | 1.8212377 | 0.0031667 | 0.0076899 | up |
| Myl1 | 3.5152469 | 0.0032499 | 0.0078668 | up |
| Itk | 2.2478815 | 0.003264 | 0.0078972 | up |
| Eno3 | 1.8066207 | 0.0033697 | 0.0081271 | up |
| Ch25h | 1.0144866 | 0.0033778 | 0.0081439 | up |
| Pde3a | 1.1262004 | 0.0033844 | 0.0081558 | up |
| Acss3 | 2.1084308 | 0.0034335 | 0.0082617 | up |
| Thbs4 | 1.4862525 | 0.0034906 | 0.0083823 | up |
| Pcdhb9 | 1.0519788 | 0.0035245 | 0.0084569 | up |
| Fam169b | 1.5823568 | 0.0036991 | 0.0088261 | up |
| 9930013L23Rik | 1.2884451 | 0.0037322 | 0.0088934 | up |
| H2-Q1 | 1.1468315 | 0.0037511 | 0.0089327 | up |
| Ankrd1 | 2.6620008 | 0.0037568 | 0.0089433 | up |
| Yipf7 | 3.2053466 | 0.0037659 | 0.0089633 | up |
| Grpr | 1.4118442 | 0.0037702 | 0.0089692 | up |
| Ppp1r3a | 3.3114787 | 0.0037726 | 0.0089734 | up |
| Traf3ip3 | 1.2343352 | 0.0037805 | 0.0089894 | up |
| Il1r2 | 1.0904974 | 0.0038363 | 0.009107 | up |
| Klhl31 | 2.8939012 | 0.003859 | 0.0091535 | up |
| Kcnj12 | 2.3715745 | 0.0039349 | 0.0093091 | up |
| Mstn | 2.8340584 | 0.0040551 | 0.0095654 | up |
| Hs3st4 | 2.0656889 | 0.0041456 | 0.0097566 | up |
| Sec14l3 | 3.1359111 | 0.0041662 | 0.0098005 | up |
| Itm2a | 1.0781787 | 0.0042582 | 0.0099872 | up |
| Begain | 1.085776 | 0.0043252 | 0.0101203 | up |
| Cd209b | 1.4021327 | 0.0043944 | 0.010259 | up |
| Mylk2 | 2.3974558 | 0.0044734 | 0.0104251 | up |
| Adig | 1.5247477 | 0.0045809 | 0.0106397 | up |
| Adcy1 | 1.1210538 | 0.0046135 | 0.0107017 | up |
| Klk8 | 1.2190213 | 0.0046935 | 0.0108682 | up |
| Des | 1.9933263 | 0.0047116 | 0.0109031 | up |
| Mybpc1 | 3.1121328 | 0.0047215 | 0.0109207 | up |
| Trim63 | 2.9708298 | 0.0048582 | 0.0112083 | up |
| Atp2a1 | 3.2607773 | 0.0048863 | 0.0112624 | up |
| Xlr4b | 1.9311255 | 0.0049618 | 0.0114201 | up |
| Mlxipl | 1.2675004 | 0.0051058 | 0.0117031 | up |
| Usp13 | 2.1011445 | 0.0051114 | 0.0117122 | up |
| Sgcg | 3.0475218 | 0.0051164 | 0.0117183 | up |
| Klhl41 | 2.2364839 | 0.0051277 | 0.0117387 | up |
| Nrn1 | 1.5306216 | 0.0051577 | 0.0117963 | up |
| Trdn | 3.1380185 | 0.0052203 | 0.0119198 | up |
| Cdhr3 | 2.5238909 | 0.0052741 | 0.0120206 | up |
| BC051019 | 2.4360116 | 0.0052884 | 0.0120437 | up |
| Tcf21 | 1.0426107 | 0.0053121 | 0.0120863 | up |
| Kcnrg | 1.7254842 | 0.0053265 | 0.0121171 | up |
| Wscd2 | 1.1055813 | 0.0053721 | 0.0122094 | up |
| Myom2 | 3.1216159 | 0.0054003 | 0.012264 | up |
| Abcb4 | 1.740272 | 0.0055259 | 0.0125218 | up |
| Eef1a2 | 2.8913752 | 0.0055337 | 0.0125336 | up |
| Myh2 | 3.2363883 | 0.005562 | 0.0125838 | up |
| Sypl2 | 2.9797478 | 0.0056003 | 0.0126647 | up |
| Olfr1396 | 1.2680185 | 0.0057144 | 0.0129025 | up |
| Ryr1 | 2.9276233 | 0.0057449 | 0.0129674 | up |
| Ampd1 | 3.049262 | 0.0058234 | 0.0131324 | up |
| Smpx | 3.0429192 | 0.0061335 | 0.0137291 | up |
| Ccl24 | 1.4369233 | 0.0061329 | 0.0137291 | up |
| Myom3 | 1.0913373 | 0.006231 | 0.0139302 | up |
| Stac3 | 1.7673172 | 0.0062972 | 0.0140566 | up |
| Pon1 | 1.450278 | 0.006361 | 0.0141858 | up |
| Cox6a2 | 2.1190573 | 0.0063977 | 0.0142611 | up |
| Pcsk1 | 1.9081159 | 0.0064196 | 0.0143055 | up |
| Foxj1 | 1.7131394 | 0.0065308 | 0.0145221 | up |
| Myh4 | 3.2996736 | 0.0066294 | 0.0147233 | up |
| 2010107G12Rik | 1.5121471 | 0.0066361 | 0.014736 | up |
| Bnc2 | 1.1599614 | 0.006649 | 0.0147579 | up |
| Sln | 3.1705935 | 0.0067353 | 0.0149152 | up |
| Tmem182 | 2.2340455 | 0.006753 | 0.014952 | up |
| Masp1 | 1.110024 | 0.0067622 | 0.0149701 | up |
| Nctc1 | 2.9590281 | 0.0070111 | 0.0154598 | up |
| Ckm | 3.0504944 | 0.0071378 | 0.0157082 | up |
| Olfm4 | 1.1132431 | 0.007307 | 0.0160453 | up |
| Adcy2 | 1.4182614 | 0.0077762 | 0.0169893 | up |
| Sdk2 | 1.2676177 | 0.0078689 | 0.0171765 | up |
| Myot | 2.9368298 | 0.0078862 | 0.017209 | up |
| Tnnt1 | 2.252444 | 0.0080374 | 0.0174916 | up |
| Cd247 | 2.062121 | 0.0081009 | 0.0176112 | up |
| Gpr30 | 1.0544516 | 0.0081486 | 0.0176937 | up |
| Adam33 | 1.0136264 | 0.0083277 | 0.018061 | up |
| Nrap | 2.6977759 | 0.0084736 | 0.0183609 | up |
| Myadml2 | 2.1029044 | 0.0088595 | 0.0191115 | up |
| Pax1 | 2.5757449 | 0.0088777 | 0.019148 | up |
| Ppp1r1a | 1.4066262 | 0.0090084 | 0.0193838 | up |
| Art1 | 3.0332197 | 0.009274 | 0.0198903 | up |
| Ccl2 | 1.0095313 | 0.0097275 | 0.0207556 | up |
| Tnnc2 | 2.9855733 | 0.0097542 | 0.0208033 | up |
| Gdf5 | 1.351964 | 0.0097975 | 0.0208894 | up |
| Tnnt3 | 2.9147107 | 0.0098346 | 0.0209409 | up |
| Asb4 | 1.5907789 | 0.0100201 | 0.0212761 | up |
| Treml2 | 1.2941917 | 0.0101068 | 0.0214325 | up |
| Rbp4 | 1.2623809 | 0.010671 | 0.0224679 | up |
| Sdr16c6 | 1.317365 | 0.0112367 | 0.0235293 | up |
| Fxyd2 | 1.2328321 | 0.0113773 | 0.0237894 | up |
| Acan | 2.5842819 | 0.0117265 | 0.024449 | up |
| Krt9 | 1.9869674 | 0.0117828 | 0.0245452 | up |
| Klhl40 | 2.8158397 | 0.0118853 | 0.0247278 | up |
| Dusp4 | 1.0914742 | 0.0119349 | 0.0248193 | up |
| H2-Bl | 1.0893806 | 0.0123089 | 0.0254947 | up |
| Synpo2l | 1.971 | 0.012459 | 0.0257725 | up |
| Nog | 1.5477079 | 0.0128464 | 0.0264795 | up |
| Sytl3 | 1.2447055 | 0.0135142 | 0.0277103 | up |
| Prkcq | 1.6272229 | 0.013611 | 0.0278774 | up |
| Il8 | 1.8916895 | 0.0136799 | 0.0279829 | up |
| Ccdc108 | 2.027443 | 0.0137028 | 0.0280257 | up |
| Acta1 | 2.5960167 | 0.013811 | 0.0282152 | up |
| Abp1 | 1.8668946 | 0.0141946 | 0.0288809 | up |
| H2-T3 | 1.7676698 | 0.0144121 | 0.029262 | up |
| Lrtm2 | 1.1014383 | 0.0152167 | 0.0306894 | up |
| Hfe2 | 3.0272644 | 0.0152419 | 0.0307359 | up |
| Isl1 | 1.0664184 | 0.0152939 | 0.0308323 | up |
| Bcl11b | 1.7207809 | 0.0153377 | 0.0309049 | up |
| Hepacam | 1.4521838 | 0.01536 | 0.0309354 | up |
| Serpina1b | 1.0067358 | 0.0153698 | 0.0309422 | up |
| Mmrn1 | 1.5964534 | 0.0170844 | 0.0339791 | up |
| Dcaf12l1 | 1.0356448 | 0.0171963 | 0.0341876 | up |
| Atp6v1b1 | 1.2319044 | 0.0172796 | 0.034325 | up |
| Dner | 1.6573046 | 0.0177038 | 0.0350444 | up |
| Pla2g4e | 2.4946168 | 0.0178154 | 0.0352158 | up |
| Retn | 1.4246165 | 0.0181145 | 0.0357484 | up |
| Myf6 | 2.5294694 | 0.0181449 | 0.0357938 | up |
| Fhad1 | 1.056922 | 0.0181804 | 0.0358541 | up |
| Asb15 | 2.6380917 | 0.018215 | 0.0359077 | up |
| Dnajc6 | 1.202883 | 0.0185527 | 0.0364743 | up |
| Smtnl1 | 1.8965241 | 0.02024 | 0.0393753 | up |
| Popdc3 | 2.8795916 | 0.0202771 | 0.0394423 | up |
| Cacna1s | 2.4040478 | 0.0206098 | 0.0400196 | up |
| Zcchc12 | 1.147909 | 0.0209224 | 0.0405308 | up |
| Rhcg | 1.8762786 | 0.0209268 | 0.040532 | up |
| Cd28 | 1.3447978 | 0.0209349 | 0.0405423 | up |
| Neb | 1.9161063 | 0.0215522 | 0.04161 | up |
| Myoc | 1.4935837 | 0.0219081 | 0.0422128 | up |
| Lrrc15 | 1.1330052 | 0.0219214 | 0.0422216 | up |
| Alpk3 | 2.1210648 | 0.0227994 | 0.0437096 | up |
| Actc1 | 2.3936803 | 0.0231534 | 0.0442712 | up |
| Prokr2 | 1.2672216 | 0.023374 | 0.0446696 | up |
| Tmod4 | 1.1058816 | 0.0237098 | 0.0452279 | up |
| Myoz2 | 2.6136957 | 0.0245441 | 0.046611 | up |
| 2010003K11Rik | 1.302071 | 0.0249907 | 0.0473661 | up |
| Actn2 | 2.3209619 | 0.0258921 | 0.048794 | up |
| Rmrp | 1.6278448 | 0.026265 | 0.0494262 | up |
| Cadm4 | -2.8586164 | 6.02E-136 | 8.73E-132 | Down |
| Abat | -2.6926571 | 6.32E-118 | 4.58E-114 | Down |
| Otub2 | -3.7830056 | 2.39E-116 | 1.16E-112 | Down |
| Snx31 | -5.560183 | 1.74E-114 | 5.04E-111 | Down |
| Kank4 | -3.6186576 | 8.10E-99 | 1.68E-95 | Down |
| Rbm20 | -3.3303441 | 2.00E-90 | 3.64E-87 | Down |
| Slc41a2 | -2.9875667 | 6.09E-89 | 9.81E-86 | Down |
| Il17rb | -2.8890576 | 2.03E-87 | 2.94E-84 | Down |
| Rdh11 | -2.8530034 | 1.41E-85 | 1.86E-82 | Down |
| Cldn6 | -4.2728368 | 3.43E-85 | 4.14E-82 | Down |
| Sptbn4 | -3.7563271 | 3.39E-83 | 3.51E-80 | Down |
| Fmn2 | -3.560095 | 3.88E-83 | 3.75E-80 | Down |
| Clec2l | -5.5007584 | 1.23E-79 | 1.12E-76 | Down |
| Ceacam1 | -2.6406461 | 4.85E-77 | 3.91E-74 | Down |
| Cnr1 | -2.9345615 | 4.13E-73 | 3.15E-70 | Down |
| Celsr1 | -2.2273358 | 6.02E-71 | 4.16E-68 | Down |
| Xylb | -2.4187688 | 2.53E-69 | 1.67E-66 | Down |
| E130309F12Rik | -4.0004162 | 2.35E-66 | 1.42E-63 | Down |
| Nlrp1a | -5.305431 | 4.50E-62 | 2.51E-59 | Down |
| Zfyve26 | -2.053789 | 7.38E-62 | 3.82E-59 | Down |
| Spag1 | -2.3978591 | 5.09E-61 | 2.38E-58 | Down |
| Cryz | -2.4323378 | 1.38E-59 | 6.07E-57 | Down |
| Wnk2 | -2.3546196 | 1.96E-58 | 7.47E-56 | Down |
| Wdr16 | -3.0005687 | 1.28E-57 | 4.77E-55 | Down |
| Kcnh3 | -3.6183763 | 7.05E-57 | 2.49E-54 | Down |
| BC021891 | -2.5361131 | 2.53E-55 | 8.14E-53 | Down |
| Sowaha | -3.5251374 | 1.68E-54 | 5.31E-52 | Down |
| Tepp | -3.8032579 | 2.16E-53 | 6.68E-51 | Down |
| Rps6ka2 | -1.9396843 | 1.02E-52 | 3.02E-50 | Down |
| Hgsnat | -1.7943729 | 3.32E-51 | 9.09E-49 | Down |
| Slc46a1 | -1.8597895 | 5.49E-51 | 1.48E-48 | Down |
| Cd79b | -3.2558955 | 1.14E-50 | 2.95E-48 | Down |
| Bspry | -1.7651447 | 1.05E-49 | 2.59E-47 | Down |
| Hsd3b6 | -6.7911393 | 8.80E-49 | 2.09E-46 | Down |
| Cldn10 | -2.7225249 | 2.07E-48 | 4.85E-46 | Down |
| Fgfr2 | -2.4185165 | 3.72E-47 | 7.94E-45 | Down |
| Nr5a1 | -4.1471058 | 1.07E-46 | 2.19E-44 | Down |
| Raver2 | -2.8057238 | 1.12E-46 | 2.25E-44 | Down |
| Hcn2 | -2.7631384 | 1.46E-46 | 2.90E-44 | Down |
| Tpr | -2.4473676 | 3.31E-46 | 6.40E-44 | Down |
| Gpr137c | -3.6126259 | 3.27E-46 | 6.40E-44 | Down |
| Kalrn | -1.643149 | 4.54E-46 | 8.67E-44 | Down |
| 1810020O05Rik | -3.9724483 | 3.06E-45 | 5.70E-43 | Down |
| Arhgef16 | -1.6097128 | 5.92E-45 | 1.09E-42 | Down |
| Fstl4 | -3.1049226 | 1.41E-44 | 2.52E-42 | Down |
| Srcin1 | -2.165315 | 4.53E-44 | 7.83E-42 | Down |
| Gm3002 | -3.1859511 | 1.88E-43 | 3.18E-41 | Down |
| Park2 | -2.6784525 | 2.87E-43 | 4.79E-41 | Down |
| Espn | -2.4820672 | 4.05E-43 | 6.68E-41 | Down |
| Phyh | -2.1087663 | 2.08E-42 | 3.31E-40 | Down |
| Gck | -3.0305702 | 2.61E-42 | 4.11E-40 | Down |
| Gjb1 | -4.0360197 | 2.74E-42 | 4.23E-40 | Down |
| Gyltl1b | -2.0082353 | 7.57E-42 | 1.12E-39 | Down |
| Gldc | -4.2605659 | 1.43E-41 | 2.08E-39 | Down |
| Arsi | -3.4689631 | 2.28E-41 | 3.27E-39 | Down |
| Clstn2 | -3.4601983 | 4.11E-41 | 5.58E-39 | Down |
| Aldob | -7.6160842 | 4.82E-41 | 6.48E-39 | Down |
| Snx22 | -2.4934414 | 7.20E-41 | 9.50E-39 | Down |
| Gng2 | -2.1002691 | 1.11E-40 | 1.45E-38 | Down |
| Cngb1 | -3.1127369 | 3.45E-40 | 4.39E-38 | Down |
| Cables2 | -1.2423397 | 5.28E-40 | 6.60E-38 | Down |
| Iqgap2 | -1.8379861 | 8.61E-40 | 1.06E-37 | Down |
| Amt | -2.0807636 | 1.61E-39 | 1.96E-37 | Down |
| Suox | -1.502364 | 1.80E-39 | 2.18E-37 | Down |
| Acsl3 | -1.4959438 | 2.80E-39 | 3.30E-37 | Down |
| Pax8 | -2.2120205 | 6.72E-39 | 7.62E-37 | Down |
| Txndc16 | -1.8031292 | 1.35E-38 | 1.52E-36 | Down |
| Tmprss3 | -4.928599 | 2.76E-38 | 3.02E-36 | Down |
| Fam92b | -4.0893981 | 2.97E-38 | 3.22E-36 | Down |
| Sall1 | -2.271016 | 4.24E-38 | 4.52E-36 | Down |
| Rdh12 | -3.4511437 | 4.47E-38 | 4.73E-36 | Down |
| 1700003M02Rik | -2.5580609 | 4.54E-38 | 4.77E-36 | Down |
| Stxbp1 | -2.3982767 | 6.44E-38 | 6.67E-36 | Down |
| Myo5c | -2.0104482 | 1.18E-37 | 1.21E-35 | Down |
| Rasgef1b | -2.5970318 | 2.58E-37 | 2.64E-35 | Down |
| 2810011L19Rik | -5.5931891 | 5.01E-37 | 5.05E-35 | Down |
| Fbxo21 | -1.6002093 | 1.04E-36 | 1.02E-34 | Down |
| Slc35f3 | -2.3816186 | 1.19E-36 | 1.16E-34 | Down |
| Acrv1 | -4.1287378 | 1.76E-36 | 1.69E-34 | Down |
| Esm1 | -2.2970571 | 2.85E-36 | 2.70E-34 | Down |
| Foxo6 | -2.7494167 | 8.17E-36 | 7.46E-34 | Down |
| AI118078 | -2.6835878 | 1.58E-35 | 1.41E-33 | Down |
| Capn6 | -2.3642277 | 1.78E-35 | 1.57E-33 | Down |
| Arhgap44 | -1.4906318 | 1.92E-35 | 1.69E-33 | Down |
| Mgat5b | -4.8882668 | 2.15E-35 | 1.88E-33 | Down |
| Lect1 | -3.431467 | 1.04E-34 | 8.76E-33 | Down |
| Plekhb1 | -1.8698226 | 1.10E-34 | 9.24E-33 | Down |
| Gm1082 | -3.459255 | 1.57E-34 | 1.31E-32 | Down |
| Cpq | -2.0812474 | 3.87E-34 | 3.21E-32 | Down |
| Tln2 | -1.3953942 | 4.00E-34 | 3.28E-32 | Down |
| Fam115c | -1.7836809 | 4.56E-34 | 3.71E-32 | Down |
| Wscd1 | -1.9793784 | 4.80E-34 | 3.89E-32 | Down |
| Ism1 | -1.6753181 | 5.20E-34 | 4.16E-32 | Down |
| Fam69a | -1.3413772 | 5.32E-34 | 4.22E-32 | Down |
| Dgka | -1.1967175 | 1.52E-33 | 1.19E-31 | Down |
| Mapk8ip1 | -1.2825045 | 1.54E-33 | 1.19E-31 | Down |
| Fam46c | -2.5173807 | 2.20E-33 | 1.68E-31 | Down |
| Plekhd1 | -1.9268005 | 3.38E-33 | 2.54E-31 | Down |
| Pm20d1 | -1.6632788 | 3.81E-33 | 2.85E-31 | Down |
| Lrrc3 | -2.5525881 | 4.48E-33 | 3.32E-31 | Down |
| Spg20 | -1.1725701 | 8.77E-33 | 6.39E-31 | Down |
| Rhpn1 | -3.289057 | 1.10E-32 | 7.92E-31 | Down |
| Ppp1r13b | -1.1659062 | 1.31E-32 | 9.36E-31 | Down |
| Sned1 | -2.503196 | 2.04E-32 | 1.44E-30 | Down |
| Mbnl3 | -2.3779157 | 2.49E-32 | 1.73E-30 | Down |
| Prodh | -1.6565189 | 3.88E-32 | 2.67E-30 | Down |
| Elovl7 | -1.9932387 | 4.57E-32 | 3.12E-30 | Down |
| Prima1 | -3.2865614 | 9.34E-32 | 6.21E-30 | Down |
| Chrdl1 | -2.6909581 | 1.23E-31 | 8.10E-30 | Down |
| Fam98a | -1.0738697 | 1.43E-31 | 9.32E-30 | Down |
| Slc51a | -4.8844588 | 1.42E-31 | 9.32E-30 | Down |
| Dcaf11 | -1.665991 | 1.62E-31 | 1.05E-29 | Down |
| Artn | -2.3341921 | 7.48E-31 | 4.59E-29 | Down |
| Cited4 | -2.0309275 | 8.16E-31 | 4.97E-29 | Down |
| Cst12 | -6.5897662 | 8.90E-31 | 5.40E-29 | Down |
| Mtor | -1.6412899 | 9.26E-31 | 5.60E-29 | Down |
| Gprasp1 | -1.6508756 | 1.71E-30 | 1.01E-28 | Down |
| Tmem213 | -3.1183438 | 1.73E-30 | 1.02E-28 | Down |
| Slc2a12 | -2.3410413 | 1.91E-30 | 1.12E-28 | Down |
| Napepld | -1.4346438 | 2.30E-30 | 1.33E-28 | Down |
| Plcd1 | -1.3253119 | 2.42E-30 | 1.40E-28 | Down |
| Ces1e | -1.5686451 | 2.93E-30 | 1.69E-28 | Down |
| Lrat | -2.9268225 | 3.60E-30 | 2.06E-28 | Down |
| Als2 | -1.3257462 | 4.18E-30 | 2.37E-28 | Down |
| Sema6c | -2.2677273 | 4.24E-30 | 2.39E-28 | Down |
| Armc2 | -2.1908586 | 5.07E-30 | 2.84E-28 | Down |
| Klhl14 | -1.4893392 | 6.07E-30 | 3.38E-28 | Down |
| Dpysl5 | -4.923918 | 1.58E-29 | 8.62E-28 | Down |
| Zfp319 | -1.3085187 | 2.63E-29 | 1.42E-27 | Down |
| Lmf1 | -1.3493868 | 2.64E-29 | 1.42E-27 | Down |
| Smarca1 | -2.3469306 | 5.07E-29 | 2.70E-27 | Down |
| Hap1 | -1.6218373 | 5.32E-29 | 2.83E-27 | Down |
| Lamc3 | -3.7524895 | 5.39E-29 | 2.86E-27 | Down |
| Fam83f | -2.3389082 | 5.53E-29 | 2.92E-27 | Down |
| Papss2 | -2.1927256 | 5.63E-29 | 2.96E-27 | Down |
| Tert | -2.0359371 | 6.28E-29 | 3.29E-27 | Down |
| Inpp5j | -2.5346523 | 7.08E-29 | 3.69E-27 | Down |
| Casc4 | -1.2989098 | 1.57E-28 | 8.10E-27 | Down |
| Ehf | -1.9792096 | 1.62E-28 | 8.32E-27 | Down |
| Ncs1 | -1.2609734 | 1.65E-28 | 8.41E-27 | Down |
| Thsd4 | -2.8282019 | 2.24E-28 | 1.13E-26 | Down |
| Mdm1 | -1.8628958 | 2.42E-28 | 1.22E-26 | Down |
| Arhgap33 | -2.3998008 | 2.96E-28 | 1.48E-26 | Down |
| Usp43 | -1.7984098 | 3.48E-28 | 1.72E-26 | Down |
| 1700024P16Rik | -2.2068833 | 3.85E-28 | 1.89E-26 | Down |
| Tfcp2l1 | -2.4694764 | 3.88E-28 | 1.90E-26 | Down |
| Mccc2 | -1.2995851 | 4.03E-28 | 1.96E-26 | Down |
| D630045J12Rik | -2.0185198 | 6.97E-28 | 3.35E-26 | Down |
| Pls1 | -1.6758736 | 1.00E-27 | 4.79E-26 | Down |
| Lrp3 | -1.3802153 | 1.06E-27 | 5.01E-26 | Down |
| Cbx7 | -1.2888108 | 1.46E-27 | 6.77E-26 | Down |
| Slc5a8 | -2.1528012 | 1.46E-27 | 6.77E-26 | Down |
| Col26a1 | -2.6110788 | 1.51E-27 | 7.00E-26 | Down |
| Efcab4b | -2.9692882 | 1.52E-27 | 7.01E-26 | Down |
| 1700030J22Rik | -1.7244732 | 1.78E-27 | 8.14E-26 | Down |
| Lrrc42 | -1.290691 | 3.00E-27 | 1.34E-25 | Down |
| Bcat1 | -1.6584033 | 3.01E-27 | 1.34E-25 | Down |
| Ero1lb | -2.8398435 | 3.16E-27 | 1.40E-25 | Down |
| Gprasp2 | -2.428463 | 3.60E-27 | 1.59E-25 | Down |
| Cldn7 | -1.3679749 | 3.88E-27 | 1.70E-25 | Down |
| Plekhg6 | -1.538149 | 6.25E-27 | 2.71E-25 | Down |
| Mctp2 | -2.2369407 | 7.49E-27 | 3.23E-25 | Down |
| Prkcz | -1.1606161 | 7.77E-27 | 3.34E-25 | Down |
| Trim36 | -2.655056 | 8.94E-27 | 3.80E-25 | Down |
| Pla2g7 | -1.5563866 | 1.11E-26 | 4.72E-25 | Down |
| Tnfrsf4 | -3.1247231 | 1.16E-26 | 4.90E-25 | Down |
| Aqp6 | -3.2712162 | 1.61E-26 | 6.72E-25 | Down |
| Gramd3 | -1.2091123 | 2.09E-26 | 8.65E-25 | Down |
| Slc2a10 | -1.4194342 | 2.89E-26 | 1.19E-24 | Down |
| Chdh | -1.7074597 | 7.60E-26 | 3.07E-24 | Down |
| Esrp1 | -1.2673009 | 9.67E-26 | 3.88E-24 | Down |
| Slc6a19 | -2.5676436 | 9.98E-26 | 3.99E-24 | Down |
| Cenpt | -1.1670855 | 1.02E-25 | 4.05E-24 | Down |
| Actr3b | -2.1937273 | 1.39E-25 | 5.48E-24 | Down |
| Appl2 | -1.2744794 | 1.45E-25 | 5.70E-24 | Down |
| Ppp1r9a | -1.2253498 | 1.86E-25 | 7.24E-24 | Down |
| C530008M17Rik | -2.1260577 | 2.80E-25 | 1.09E-23 | Down |
| Ncam1 | -1.586944 | 2.96E-25 | 1.14E-23 | Down |
| Rhpn2 | -1.8667841 | 3.47E-25 | 1.33E-23 | Down |
| Ctnnal1 | -1.1056954 | 3.61E-25 | 1.38E-23 | Down |
| Zfp385b | -2.725302 | 3.98E-25 | 1.52E-23 | Down |
| Plxdc1 | -1.9912114 | 6.68E-25 | 2.52E-23 | Down |
| Trim2 | -1.3282612 | 7.25E-25 | 2.72E-23 | Down |
| Oxnad1 | -1.4047731 | 7.26E-25 | 2.72E-23 | Down |
| L3mbtl4 | -3.5407283 | 7.54E-25 | 2.82E-23 | Down |
| Slc22a21 | -2.6402275 | 7.59E-25 | 2.83E-23 | Down |
| Rnf208 | -2.4390426 | 8.80E-25 | 3.27E-23 | Down |
| Sdc2 | -1.1202749 | 1.18E-24 | 4.36E-23 | Down |
| 2610035D17Rik | -2.3363866 | 1.34E-24 | 4.90E-23 | Down |
| Hspa1a | -1.7140942 | 1.39E-24 | 5.08E-23 | Down |
| Aldh7a1 | -1.0343494 | 1.42E-24 | 5.15E-23 | Down |
| Nmnat3 | -2.1076992 | 1.67E-24 | 6.00E-23 | Down |
| Fbxo44 | -1.4914966 | 1.83E-24 | 6.56E-23 | Down |
| Rai2 | -2.5103643 | 1.95E-24 | 6.97E-23 | Down |
| Fam184a | -3.1097207 | 2.02E-24 | 7.20E-23 | Down |
| Bcl7a | -1.3710249 | 2.27E-24 | 8.03E-23 | Down |
| 5430425J12Rik | -4.9932932 | 2.31E-24 | 8.16E-23 | Down |
| Arhgef33 | -2.4801827 | 3.06E-24 | 1.08E-22 | Down |
| 6430548M08Rik | -1.6484176 | 3.21E-24 | 1.13E-22 | Down |
| Map6d1 | -2.9805063 | 3.68E-24 | 1.29E-22 | Down |
| Cgn | -1.2437561 | 4.19E-24 | 1.46E-22 | Down |
| Lcn8 | -3.2269284 | 4.84E-24 | 1.68E-22 | Down |
| Arhgef37 | -3.6307782 | 4.86E-24 | 1.68E-22 | Down |
| Gm15413 | -3.2814323 | 4.97E-24 | 1.72E-22 | Down |
| Nipal3 | -1.0652267 | 5.04E-24 | 1.74E-22 | Down |
| Slc6a12 | -3.1449033 | 5.11E-24 | 1.76E-22 | Down |
| Dll4 | -2.034464 | 5.84E-24 | 2.00E-22 | Down |
| Atp6v0a4 | -1.7951596 | 7.30E-24 | 2.49E-22 | Down |
| Bnc1 | -4.8519171 | 8.11E-24 | 2.75E-22 | Down |
| Dgkg | -2.2240027 | 9.22E-24 | 3.11E-22 | Down |
| Vegfa | -2.1494405 | 1.02E-23 | 3.44E-22 | Down |
| Sez6l | -1.8254037 | 1.58E-23 | 5.25E-22 | Down |
| Epb4.9 | -1.441212 | 2.19E-23 | 7.20E-22 | Down |
| Lrrc66 | -4.4061327 | 2.46E-23 | 8.05E-22 | Down |
| Rbms3 | -1.3629491 | 2.60E-23 | 8.49E-22 | Down |
| Kcnj16 | -2.7239432 | 3.05E-23 | 9.93E-22 | Down |
| Themis3 | -4.0872632 | 3.40E-23 | 1.10E-21 | Down |
| Cd320 | -1.1710465 | 3.48E-23 | 1.12E-21 | Down |
| Sox9 | -1.8866858 | 3.66E-23 | 1.17E-21 | Down |
| AI661453 | -1.4447631 | 3.83E-23 | 1.22E-21 | Down |
| Cnbd2 | -1.8177139 | 4.59E-23 | 1.46E-21 | Down |
| Myh10 | -1.7517219 | 6.19E-23 | 1.94E-21 | Down |
| Cacna1d | -1.5229058 | 6.64E-23 | 2.06E-21 | Down |
| D3Bwg0562e | -2.0889749 | 1.11E-22 | 3.37E-21 | Down |
| Car5b | -1.6556936 | 2.48E-22 | 7.41E-21 | Down |
| Gramd1c | -1.4928252 | 2.49E-22 | 7.41E-21 | Down |
| Zfp804b | -5.3987654 | 2.86E-22 | 8.45E-21 | Down |
| Rimklb | -3.6465506 | 2.96E-22 | 8.69E-21 | Down |
| Sarm1 | -3.317776 | 3.06E-22 | 8.97E-21 | Down |
| Slc22a17 | -1.7320571 | 3.46E-22 | 1.01E-20 | Down |
| Btc | -2.3701154 | 3.53E-22 | 1.03E-20 | Down |
| Sowahb | -1.6425124 | 3.74E-22 | 1.08E-20 | Down |
| Efcab6 | -3.9508378 | 5.47E-22 | 1.57E-20 | Down |
| Acoxl | -4.0453557 | 5.86E-22 | 1.68E-20 | Down |
| Arg2 | -1.4496293 | 7.03E-22 | 2.00E-20 | Down |
| Gng7 | -1.8691024 | 7.11E-22 | 2.02E-20 | Down |
| Cd276 | -1.377663 | 8.86E-22 | 2.49E-20 | Down |
| Cd164l2 | -2.5859167 | 8.84E-22 | 2.49E-20 | Down |
| Sh3gl2 | -1.6928928 | 1.02E-21 | 2.85E-20 | Down |
| Mon1b | -1.1271888 | 1.12E-21 | 3.14E-20 | Down |
| Zbtb8b | -2.7408387 | 1.17E-21 | 3.26E-20 | Down |
| Hspa1b | -1.4647129 | 1.53E-21 | 4.24E-20 | Down |
| Tjp3 | -1.1527598 | 1.61E-21 | 4.44E-20 | Down |
| Csrnp3 | -3.8470126 | 1.64E-21 | 4.52E-20 | Down |
| Snph | -1.6461319 | 1.67E-21 | 4.58E-20 | Down |
| Csrp1 | -1.5343546 | 1.72E-21 | 4.73E-20 | Down |
| Arhgef19 | -1.332979 | 2.07E-21 | 5.62E-20 | Down |
| Gstt3 | -1.185253 | 3.44E-21 | 9.21E-20 | Down |
| Wasf3 | -1.8062872 | 3.47E-21 | 9.28E-20 | Down |
| 5830411N06Rik | -5.1240785 | 4.10E-21 | 1.09E-19 | Down |
| Slc23a1 | -1.3854309 | 4.64E-21 | 1.23E-19 | Down |
| Aspg | -2.419072 | 5.16E-21 | 1.35E-19 | Down |
| Mfsd7c | -2.6650877 | 6.50E-21 | 1.69E-19 | Down |
| Derl3 | -2.4459042 | 6.85E-21 | 1.78E-19 | Down |
| Apoc1 | -2.2242353 | 8.04E-21 | 2.08E-19 | Down |
| Rassf10 | -1.4157435 | 9.00E-21 | 2.33E-19 | Down |
| Nsun7 | -3.0058349 | 9.18E-21 | 2.37E-19 | Down |
| Kcne3 | -2.6740579 | 9.20E-21 | 2.37E-19 | Down |
| A330041J22Rik | -2.1824194 | 9.46E-21 | 2.43E-19 | Down |
| Mcc | -1.410207 | 1.39E-20 | 3.49E-19 | Down |
| Tspan1 | -1.9837297 | 1.59E-20 | 3.95E-19 | Down |
| Cacna1b | -3.9538895 | 1.65E-20 | 4.08E-19 | Down |
| St3gal5 | -1.1929128 | 1.70E-20 | 4.20E-19 | Down |
| Pdzrn3 | -1.0089915 | 1.82E-20 | 4.48E-19 | Down |
| Ces3a | -5.1254512 | 1.89E-20 | 4.65E-19 | Down |
| Ldlrad3 | -1.0833714 | 2.06E-20 | 5.03E-19 | Down |
| Optn | -1.0387587 | 2.36E-20 | 5.73E-19 | Down |
| Chst3 | -2.0309478 | 2.44E-20 | 5.90E-19 | Down |
| Ptdss2 | -1.1320597 | 2.45E-20 | 5.92E-19 | Down |
| Lpar2 | -1.6818832 | 2.63E-20 | 6.30E-19 | Down |
| Agr3 | -2.8570586 | 3.20E-20 | 7.63E-19 | Down |
| Tmem108 | -2.3527614 | 4.37E-20 | 1.04E-18 | Down |
| Epcam | -1.2528503 | 4.89E-20 | 1.16E-18 | Down |
| Mfsd4 | -1.7714374 | 5.33E-20 | 1.26E-18 | Down |
| Mapk8ip3 | -1.1590748 | 5.43E-20 | 1.28E-18 | Down |
| Epb4.1l4a | -1.6427493 | 5.80E-20 | 1.37E-18 | Down |
| Mylk | -1.209827 | 5.99E-20 | 1.41E-18 | Down |
| Trim16 | -1.2318635 | 6.43E-20 | 1.51E-18 | Down |
| Slc12a5 | -2.378969 | 6.48E-20 | 1.52E-18 | Down |
| Ss18l1 | -1.1984041 | 7.25E-20 | 1.69E-18 | Down |
| Dlgap1 | -1.7735764 | 7.86E-20 | 1.83E-18 | Down |
| Rap1gap | -2.0425033 | 8.02E-20 | 1.86E-18 | Down |
| Kifc2 | -1.549421 | 9.13E-20 | 2.11E-18 | Down |
| Chek1 | -2.1404889 | 9.13E-20 | 2.11E-18 | Down |
| Cmtm8 | -1.3074077 | 9.53E-20 | 2.19E-18 | Down |
| Spon1 | -1.305147 | 1.05E-19 | 2.40E-18 | Down |
| Hsbp1l1 | -3.6910047 | 1.06E-19 | 2.43E-18 | Down |
| Dusp7 | -1.1331762 | 1.08E-19 | 2.47E-18 | Down |
| Hunk | -1.9275899 | 1.53E-19 | 3.45E-18 | Down |
| Ccdc160 | -1.8642387 | 1.88E-19 | 4.21E-18 | Down |
| Gm19990 | -3.2955475 | 1.91E-19 | 4.27E-18 | Down |
| Sfrp5 | -2.7099881 | 1.93E-19 | 4.31E-18 | Down |
| Ckmt1 | -2.0493267 | 2.03E-19 | 4.49E-18 | Down |
| Ica1 | -1.134386 | 2.13E-19 | 4.72E-18 | Down |
| Gm7694 | -1.6735349 | 2.51E-19 | 5.51E-18 | Down |
| Elmo3 | -1.0995168 | 2.76E-19 | 6.04E-18 | Down |
| Hlf | -1.8883325 | 2.77E-19 | 6.06E-18 | Down |
| Alox5 | -2.1877298 | 3.11E-19 | 6.74E-18 | Down |
| Tnfsf18 | -3.7567264 | 4.13E-19 | 8.88E-18 | Down |
| Gnao1 | -2.7625636 | 4.33E-19 | 9.29E-18 | Down |
| Qsox1 | -1.0519252 | 5.03E-19 | 1.07E-17 | Down |
| Chd1l | -1.3447461 | 6.21E-19 | 1.32E-17 | Down |
| Ptprn2 | -4.4338502 | 6.32E-19 | 1.34E-17 | Down |
| Sdf4 | -1.1189534 | 7.91E-19 | 1.66E-17 | Down |
| Pald1 | -1.0893145 | 8.15E-19 | 1.71E-17 | Down |
| Epb4.1l1 | -1.0801481 | 8.33E-19 | 1.75E-17 | Down |
| Rrbp1 | -1.4461531 | 9.32E-19 | 1.94E-17 | Down |
| Tbl2 | -1.0187411 | 9.68E-19 | 2.01E-17 | Down |
| Ankhd1 | -1.2339517 | 1.03E-18 | 2.12E-17 | Down |
| Kif17 | -2.5615383 | 1.14E-18 | 2.35E-17 | Down |
| Ttc39c | -1.8012492 | 1.15E-18 | 2.37E-17 | Down |
| Magi1 | -1.2258075 | 1.16E-18 | 2.38E-17 | Down |
| Tmem116 | -1.2936522 | 1.35E-18 | 2.75E-17 | Down |
| Kit | -1.5332538 | 1.60E-18 | 3.26E-17 | Down |
| Bmp7 | -2.1607423 | 1.65E-18 | 3.34E-17 | Down |
| Gm13003 | -2.955785 | 1.78E-18 | 3.61E-17 | Down |
| Klrg2 | -1.8048813 | 1.79E-18 | 3.63E-17 | Down |
| Dapk1 | -1.3513887 | 1.83E-18 | 3.70E-17 | Down |
| Tox3 | -1.6073659 | 1.99E-18 | 4.00E-17 | Down |
| Klhl13 | -1.4151173 | 2.14E-18 | 4.31E-17 | Down |
| Hs3st6 | -1.3246206 | 2.58E-18 | 5.14E-17 | Down |
| Kcns3 | -2.1260981 | 2.62E-18 | 5.21E-17 | Down |
| Hid1 | -1.4040061 | 2.88E-18 | 5.72E-17 | Down |
| Sprn | -2.7064165 | 3.03E-18 | 5.99E-17 | Down |
| Irf6 | -1.2199246 | 3.42E-18 | 6.74E-17 | Down |
| Syt13 | -2.1920484 | 3.45E-18 | 6.79E-17 | Down |
| Rab15 | -1.0913102 | 3.51E-18 | 6.89E-17 | Down |
| Marveld2 | -1.312713 | 3.74E-18 | 7.34E-17 | Down |
| Prrg3 | -1.6967074 | 3.75E-18 | 7.35E-17 | Down |
| Me2 | -1.2224529 | 3.77E-18 | 7.37E-17 | Down |
| Otof | -3.95233 | 3.85E-18 | 7.51E-17 | Down |
| Ap1m2 | -1.1230586 | 3.87E-18 | 7.55E-17 | Down |
| Dcaf17 | -1.0270914 | 4.19E-18 | 8.13E-17 | Down |
| Fras1 | -1.784518 | 4.39E-18 | 8.50E-17 | Down |
| Celf4 | -2.1688209 | 4.57E-18 | 8.83E-17 | Down |
| 1500009L16Rik | -2.6446005 | 4.61E-18 | 8.90E-17 | Down |
| Clvs1 | -4.0215598 | 5.49E-18 | 1.05E-16 | Down |
| Mchr1 | -2.3822112 | 5.58E-18 | 1.07E-16 | Down |
| Morn1 | -1.6175317 | 5.86E-18 | 1.12E-16 | Down |
| Tmem184c | -1.0220513 | 5.98E-18 | 1.14E-16 | Down |
| Nipal2 | -1.0869036 | 6.28E-18 | 1.20E-16 | Down |
| 1500015L24Rik | -3.2796139 | 6.61E-18 | 1.26E-16 | Down |
| Zxda | -1.2151854 | 6.78E-18 | 1.29E-16 | Down |
| AK129341 | -1.4041841 | 8.28E-18 | 1.56E-16 | Down |
| Them4 | -1.5564053 | 8.93E-18 | 1.67E-16 | Down |
| Grhpr | -1.108242 | 9.24E-18 | 1.72E-16 | Down |
| Igfn1 | -2.6267227 | 9.81E-18 | 1.82E-16 | Down |
| Slc6a18 | -3.2399577 | 1.07E-17 | 1.98E-16 | Down |
| Exph5 | -2.0584274 | 1.12E-17 | 2.08E-16 | Down |
| Kif21a | -1.0542547 | 1.16E-17 | 2.15E-16 | Down |
| Rgs9 | -2.2425117 | 1.23E-17 | 2.27E-16 | Down |
| Car12 | -3.393811 | 1.31E-17 | 2.40E-16 | Down |
| Tiam1 | -1.1702258 | 1.60E-17 | 2.92E-16 | Down |
| Cdca7l | -1.2806449 | 1.66E-17 | 3.02E-16 | Down |
| Grik3 | -1.8856471 | 1.87E-17 | 3.40E-16 | Down |
| Slc12a8 | -1.4903821 | 2.38E-17 | 4.32E-16 | Down |
| Fam174b | -2.1221838 | 2.39E-17 | 4.33E-16 | Down |
| Shh | -7.9672715 | 2.42E-17 | 4.38E-16 | Down |
| Clcnka | -2.4202132 | 2.52E-17 | 4.55E-16 | Down |
| Ppargc1b | -2.8145257 | 2.80E-17 | 5.02E-16 | Down |
| Stk36 | -1.4109723 | 2.86E-17 | 5.12E-16 | Down |
| Nav2 | -1.0580675 | 3.24E-17 | 5.76E-16 | Down |
| Ccdc74a | -3.0930867 | 3.37E-17 | 5.99E-16 | Down |
| Ccdc148 | -2.0066091 | 3.57E-17 | 6.32E-16 | Down |
| Tbx3 | -1.4246005 | 3.98E-17 | 6.97E-16 | Down |
| Aif1l | -1.7283159 | 4.40E-17 | 7.67E-16 | Down |
| Gpihbp1 | -2.2144982 | 5.06E-17 | 8.79E-16 | Down |
| Peli2 | -1.3388315 | 5.08E-17 | 8.81E-16 | Down |
| Dcun1d4 | -1.0649429 | 5.91E-17 | 1.02E-15 | Down |
| Enah | -1.1836864 | 5.95E-17 | 1.02E-15 | Down |
| Hs3st3b1 | -1.8374371 | 6.52E-17 | 1.12E-15 | Down |
| Ccdc8 | -1.35307 | 6.67E-17 | 1.14E-15 | Down |
| 9230112J17Rik | -3.8858088 | 7.36E-17 | 1.25E-15 | Down |
| Nrtn | -2.3243899 | 7.93E-17 | 1.34E-15 | Down |
| Vav3 | -1.1009 | 8.31E-17 | 1.40E-15 | Down |
| Aifm3 | -1.7523422 | 8.74E-17 | 1.48E-15 | Down |
| Bbs2 | -1.0941279 | 9.01E-17 | 1.52E-15 | Down |
| Fam169a | -3.2189226 | 1.00E-16 | 1.69E-15 | Down |
| Scnn1a | -1.0120062 | 1.07E-16 | 1.79E-15 | Down |
| Aph1b | -1.5915694 | 1.17E-16 | 1.95E-15 | Down |
| Pdxk | -1.2426207 | 1.19E-16 | 1.98E-15 | Down |
| Tmem42 | -1.2651631 | 1.22E-16 | 2.02E-15 | Down |
| Notch1 | -1.271502 | 1.36E-16 | 2.24E-15 | Down |
| Nek1 | -1.4166339 | 1.42E-16 | 2.32E-15 | Down |
| Gch1 | -1.4458946 | 1.53E-16 | 2.51E-15 | Down |
| Hist3h2ba | -3.0634071 | 1.55E-16 | 2.53E-15 | Down |
| Tmem117 | -1.0822255 | 1.56E-16 | 2.55E-15 | Down |
| 8-Mar | -1.045943 | 1.64E-16 | 2.66E-15 | Down |
| Mmp16 | -2.5831652 | 1.64E-16 | 2.67E-15 | Down |
| Slc25a23 | -1.2058119 | 2.08E-16 | 3.35E-15 | Down |
| Npc2 | -1.6412668 | 2.16E-16 | 3.47E-15 | Down |
| Spin2 | -1.9361285 | 2.42E-16 | 3.89E-15 | Down |
| Nudt10 | -1.3040942 | 2.52E-16 | 4.04E-15 | Down |
| Tdrd9 | -2.2507671 | 2.57E-16 | 4.10E-15 | Down |
| Zfp612 | -1.0859953 | 2.84E-16 | 4.53E-15 | Down |
| Smpdl3b | -1.7932318 | 2.87E-16 | 4.56E-15 | Down |
| Tbc1d30 | -2.5913023 | 3.29E-16 | 5.22E-15 | Down |
| Scgb1c1 | -3.0034578 | 3.39E-16 | 5.37E-15 | Down |
| Tg | -2.1932532 | 3.51E-16 | 5.54E-15 | Down |
| Mt3 | -2.0547263 | 3.62E-16 | 5.70E-15 | Down |
| Kif27 | -1.8864571 | 3.94E-16 | 6.15E-15 | Down |
| Smoc1 | -2.5071713 | 4.03E-16 | 6.29E-15 | Down |
| Mroh2a | -3.999026 | 4.50E-16 | 6.97E-15 | Down |
| Bmp2k | -1.2377344 | 5.30E-16 | 8.19E-15 | Down |
| Cacna1h | -1.8161128 | 5.62E-16 | 8.66E-15 | Down |
| Stc1 | -2.2034183 | 5.78E-16 | 8.89E-15 | Down |
| Slc44a3 | -1.3466651 | 6.61E-16 | 1.01E-14 | Down |
| Mro | -2.2945648 | 6.61E-16 | 1.01E-14 | Down |
| Ppl | -1.218222 | 6.78E-16 | 1.03E-14 | Down |
| Zfr2 | -1.3212428 | 7.75E-16 | 1.18E-14 | Down |
| Ccdc64b | -1.3692662 | 7.83E-16 | 1.19E-14 | Down |
| Ednrb | -1.5457257 | 9.42E-16 | 1.42E-14 | Down |
| 4930426D05Rik | -1.7924756 | 9.72E-16 | 1.46E-14 | Down |
| Trabd2b | -1.3591633 | 1.05E-15 | 1.57E-14 | Down |
| Agpat4 | -1.1143537 | 1.11E-15 | 1.65E-14 | Down |
| Slc4a5 | -1.6171324 | 1.21E-15 | 1.81E-14 | Down |
| Ripk4 | -1.1671211 | 1.27E-15 | 1.89E-14 | Down |
| Cxcr7 | -1.5731379 | 1.28E-15 | 1.90E-14 | Down |
| Mpp2 | -1.2226798 | 1.53E-15 | 2.26E-14 | Down |
| Rhbg | -2.5742429 | 1.72E-15 | 2.53E-14 | Down |
| Smad9 | -1.4683939 | 1.91E-15 | 2.81E-14 | Down |
| Adm2 | -2.3001919 | 1.98E-15 | 2.90E-14 | Down |
| Sptbn2 | -1.4992645 | 2.05E-15 | 3.00E-14 | Down |
| Chrna2 | -2.877689 | 2.47E-15 | 3.59E-14 | Down |
| Prosapip1 | -1.210937 | 2.82E-15 | 4.08E-14 | Down |
| Grb14 | -1.7571922 | 2.93E-15 | 4.23E-14 | Down |
| 4930502A04Rik | -2.4244918 | 3.09E-15 | 4.44E-14 | Down |
| Bcas1 | -2.1666188 | 3.13E-15 | 4.49E-14 | Down |
| Cyp2j13 | -3.7413328 | 3.20E-15 | 4.58E-14 | Down |
| Gucy2f | -4.0246664 | 3.42E-15 | 4.88E-14 | Down |
| Mansc1 | -1.0237075 | 3.53E-15 | 5.04E-14 | Down |
| Phyhipl | -2.3073986 | 3.64E-15 | 5.18E-14 | Down |
| Rab11fip4 | -2.0451011 | 3.85E-15 | 5.46E-14 | Down |
| Dbx2 | -4.0322385 | 4.06E-15 | 5.76E-14 | Down |
| Tmem52 | -2.1427013 | 4.14E-15 | 5.86E-14 | Down |
| Tspyl4 | -1.1241447 | 4.77E-15 | 6.74E-14 | Down |
| Smpd3 | -1.5858553 | 5.10E-15 | 7.19E-14 | Down |
| Cep128 | -1.586593 | 5.67E-15 | 7.93E-14 | Down |
| Armcx6 | -1.1574968 | 6.03E-15 | 8.43E-14 | Down |
| Fam57b | -2.1079838 | 6.15E-15 | 8.58E-14 | Down |
| Ldhd | -1.2112722 | 6.46E-15 | 8.99E-14 | Down |
| Abhd3 | -1.3581084 | 7.01E-15 | 9.70E-14 | Down |
| Cntnap5a | -4.5412451 | 7.48E-15 | 1.03E-13 | Down |
| Dach2 | -2.7641963 | 7.92E-15 | 1.09E-13 | Down |
| Ccdc162 | -1.8914137 | 8.51E-15 | 1.17E-13 | Down |
| Dlgap2 | -3.0554653 | 8.93E-15 | 1.23E-13 | Down |
| Mlph | -1.2331693 | 9.24E-15 | 1.26E-13 | Down |
| Prob1 | -1.396677 | 9.24E-15 | 1.26E-13 | Down |
| Tmem200b | -1.9391304 | 1.01E-14 | 1.38E-13 | Down |
| Cntnap2 | -4.005785 | 1.02E-14 | 1.38E-13 | Down |
| Kcnn2 | -2.0592522 | 1.07E-14 | 1.45E-13 | Down |
| Cachd1 | -1.1011486 | 1.18E-14 | 1.60E-13 | Down |
| BC068157 | -1.8414678 | 1.22E-14 | 1.65E-13 | Down |
| Gcdh | -1.3939779 | 1.50E-14 | 2.01E-13 | Down |
| Mei1 | -6.8905064 | 1.51E-14 | 2.03E-13 | Down |
| Tes | -1.1573458 | 1.58E-14 | 2.12E-13 | Down |
| 1700018A04Rik | -3.7018803 | 1.76E-14 | 2.35E-13 | Down |
| Vash2 | -1.7014699 | 1.79E-14 | 2.38E-13 | Down |
| Cdon | -1.4383407 | 1.81E-14 | 2.40E-13 | Down |
| Add3 | -1.1157024 | 2.04E-14 | 2.70E-13 | Down |
| Acpl2 | -1.0054495 | 2.07E-14 | 2.74E-13 | Down |
| 2410131K14Rik | -1.2185096 | 2.10E-14 | 2.78E-13 | Down |
| Prdm14 | -7.8752164 | 2.81E-14 | 3.68E-13 | Down |
| Epha1 | -1.3115578 | 3.35E-14 | 4.35E-13 | Down |
| Alas2 | -3.75451 | 4.33E-14 | 5.57E-13 | Down |
| Clcn2 | -1.1004584 | 4.73E-14 | 6.04E-13 | Down |
| Eps8l2 | -1.2178862 | 4.73E-14 | 6.04E-13 | Down |
| Bsn | -1.9923037 | 5.16E-14 | 6.57E-13 | Down |
| Ankrd46 | -1.0609584 | 5.21E-14 | 6.63E-13 | Down |
| Lmo3 | -2.9026424 | 5.37E-14 | 6.82E-13 | Down |
| Ano1 | -1.2816679 | 5.74E-14 | 7.23E-13 | Down |
| Fzd3 | -1.0322482 | 5.80E-14 | 7.29E-13 | Down |
| Mmp15 | -1.0389108 | 6.00E-14 | 7.54E-13 | Down |
| Tmem8c | -5.6213606 | 6.39E-14 | 8.00E-13 | Down |
| Fkbp4 | -1.1239756 | 6.45E-14 | 8.06E-13 | Down |
| Sec14l5 | -2.341882 | 6.80E-14 | 8.47E-13 | Down |
| Slc6a3 | -2.6709372 | 7.06E-14 | 8.79E-13 | Down |
| Shroom1 | -1.441673 | 8.01E-14 | 9.91E-13 | Down |
| Pabpn1l | -1.4850026 | 8.05E-14 | 9.96E-13 | Down |
| Lonrf3 | -1.3021889 | 9.85E-14 | 1.21E-12 | Down |
| Susd4 | -2.1716809 | 1.03E-13 | 1.26E-12 | Down |
| Cux2 | -1.406914 | 1.07E-13 | 1.32E-12 | Down |
| Nptx1 | -1.773013 | 1.20E-13 | 1.46E-12 | Down |
| Egf | -1.462875 | 1.27E-13 | 1.54E-12 | Down |
| B230312C02Rik | -6.0590914 | 1.27E-13 | 1.55E-12 | Down |
| Fam189a2 | -1.2052768 | 1.64E-13 | 1.97E-12 | Down |
| Cspg5 | -2.3393256 | 1.68E-13 | 2.02E-12 | Down |
| Aph1c | -1.3366548 | 1.83E-13 | 2.20E-12 | Down |
| Arl4d | -1.7687185 | 1.85E-13 | 2.22E-12 | Down |
| LOC100861615 | -2.5200882 | 1.95E-13 | 2.33E-12 | Down |
| Ppp3cc | -1.0206001 | 2.92E-13 | 3.44E-12 | Down |
| Sox5 | -1.1128835 | 2.93E-13 | 3.44E-12 | Down |
| 4932438H23Rik | -1.3366652 | 3.18E-13 | 3.73E-12 | Down |
| Macrod2 | -1.3105331 | 3.23E-13 | 3.79E-12 | Down |
| Ncrna00086 | -1.316593 | 3.31E-13 | 3.88E-12 | Down |
| Cecr2 | -1.2162106 | 3.40E-13 | 3.98E-12 | Down |
| Gm3558 | -2.5163504 | 3.42E-13 | 3.99E-12 | Down |
| Mpped2 | -1.4631062 | 3.56E-13 | 4.14E-12 | Down |
| Calml3 | -1.5131613 | 3.69E-13 | 4.28E-12 | Down |
| Spns2 | -1.0113491 | 3.74E-13 | 4.33E-12 | Down |
| Nt5dc3 | -1.2049982 | 3.76E-13 | 4.35E-12 | Down |
| Nkd1 | -1.8494624 | 3.85E-13 | 4.45E-12 | Down |
| Slc6a17 | -1.2367315 | 4.40E-13 | 5.06E-12 | Down |
| Ctxn1 | -1.0239414 | 4.55E-13 | 5.22E-12 | Down |
| Amph | -1.5701463 | 4.73E-13 | 5.41E-12 | Down |
| Marveld3 | -1.0250469 | 5.31E-13 | 6.04E-12 | Down |
| Elfn1 | -1.5364567 | 5.36E-13 | 6.10E-12 | Down |
| Itpka | -2.2710929 | 5.84E-13 | 6.59E-12 | Down |
| Ccdc68 | -1.2480582 | 5.86E-13 | 6.61E-12 | Down |
| Tmem37 | -1.1543864 | 6.05E-13 | 6.81E-12 | Down |
| Esrrb | -2.149121 | 6.26E-13 | 7.03E-12 | Down |
| Kcnj5 | -2.3520789 | 6.46E-13 | 7.23E-12 | Down |
| Hmcn1 | -1.2057603 | 6.83E-13 | 7.62E-12 | Down |
| Rnf223 | -2.3218315 | 6.89E-13 | 7.68E-12 | Down |
| Ldhb | -1.4572949 | 7.19E-13 | 7.99E-12 | Down |
| Svopl | -1.8873603 | 7.41E-13 | 8.23E-12 | Down |
| Dio1 | -2.0123414 | 7.99E-13 | 8.85E-12 | Down |
| Slco4a1 | -3.2221217 | 8.35E-13 | 9.24E-12 | Down |
| Gas7 | -1.1279408 | 8.60E-13 | 9.50E-12 | Down |
| Elovl2 | -1.6509091 | 9.04E-13 | 9.98E-12 | Down |
| Edar | -1.8096776 | 1.03E-12 | 1.13E-11 | Down |
| Apcdd1 | -1.2868041 | 1.03E-12 | 1.13E-11 | Down |
| Lrig3 | -1.0731178 | 1.05E-12 | 1.15E-11 | Down |
| Fads6 | -2.2852442 | 1.15E-12 | 1.26E-11 | Down |
| Obp2b | -4.0794223 | 1.17E-12 | 1.28E-11 | Down |
| Gpr160 | -1.2301848 | 1.30E-12 | 1.41E-11 | Down |
| Tns3 | -1.094024 | 1.43E-12 | 1.54E-11 | Down |
| Beta-s | -4.1215232 | 1.44E-12 | 1.54E-11 | Down |
| Slc1a3 | -1.3476038 | 1.46E-12 | 1.57E-11 | Down |
| Stard8 | -1.2134669 | 1.49E-12 | 1.60E-11 | Down |
| Bace2 | -1.9910012 | 1.72E-12 | 1.83E-11 | Down |
| Tle2 | -1.2437897 | 1.80E-12 | 1.91E-11 | Down |
| Lrfn1 | -1.3829271 | 1.91E-12 | 2.03E-11 | Down |
| Gramd2 | -2.0330983 | 1.93E-12 | 2.04E-11 | Down |
| Fam110c | -1.1717741 | 1.99E-12 | 2.10E-11 | Down |
| Orai2 | -1.1532997 | 2.54E-12 | 2.66E-11 | Down |
| Dmrtc1b | -2.8009207 | 2.57E-12 | 2.69E-11 | Down |
| Larp6 | -1.6381424 | 2.57E-12 | 2.69E-11 | Down |
| Bex4 | -1.7326558 | 2.76E-12 | 2.87E-11 | Down |
| Pllp | -2.0252576 | 2.76E-12 | 2.87E-11 | Down |
| Camkk1 | -1.2227177 | 2.77E-12 | 2.88E-11 | Down |
| Agt | -1.7994202 | 2.79E-12 | 2.89E-11 | Down |
| Scn9a | -5.0791879 | 2.82E-12 | 2.93E-11 | Down |
| 1810019J16Rik | -1.0073996 | 3.00E-12 | 3.11E-11 | Down |
| Slc7a4 | -1.1936486 | 3.10E-12 | 3.21E-11 | Down |
| Ndufa1 | -1.1993575 | 3.46E-12 | 3.56E-11 | Down |
| Ubash3b | -1.0726098 | 3.61E-12 | 3.69E-11 | Down |
| Adora2b | -1.1540654 | 5.17E-12 | 5.23E-11 | Down |
| Syt3 | -1.6660156 | 7.57E-12 | 7.48E-11 | Down |
| Adra1a | -1.7958782 | 7.96E-12 | 7.86E-11 | Down |
| Aak1 | -1.0782117 | 8.47E-12 | 8.34E-11 | Down |
| Tmc7 | -1.4754459 | 1.01E-11 | 9.86E-11 | Down |
| BC006965 | -2.4484875 | 1.03E-11 | 1.00E-10 | Down |
| Sphk1 | -1.1541958 | 1.15E-11 | 1.12E-10 | Down |
| Ppp1r1b | -1.7703036 | 1.18E-11 | 1.14E-10 | Down |
| Fam178b | -1.6675562 | 1.34E-11 | 1.30E-10 | Down |
| Spock2 | -2.1195772 | 1.62E-11 | 1.55E-10 | Down |
| Glis2 | -1.0279357 | 1.74E-11 | 1.65E-10 | Down |
| Slc6a13 | -2.8264196 | 1.86E-11 | 1.76E-10 | Down |
| Opcml | -1.6422404 | 1.92E-11 | 1.82E-10 | Down |
| Usp11 | -1.0534058 | 1.93E-11 | 1.83E-10 | Down |
| Egfr | -1.0893795 | 1.94E-11 | 1.83E-10 | Down |
| Slc14a2 | -2.7477758 | 2.14E-11 | 2.01E-10 | Down |
| 0610040J01Rik | -1.0750717 | 2.75E-11 | 2.57E-10 | Down |
| Gm5796 | -2.8480982 | 2.98E-11 | 2.77E-10 | Down |
| Stc2 | -1.3616441 | 3.01E-11 | 2.80E-10 | Down |
| Tnfrsf18 | -1.5505363 | 3.02E-11 | 2.80E-10 | Down |
| Rln1 | -3.9753827 | 3.09E-11 | 2.86E-10 | Down |
| 2700054A10Rik | -1.506645 | 3.12E-11 | 2.88E-10 | Down |
| Prr5 | -1.1306985 | 3.31E-11 | 3.05E-10 | Down |
| Zscan18 | -1.4020311 | 3.33E-11 | 3.07E-10 | Down |
| Pitpnm3 | -1.1239958 | 3.39E-11 | 3.13E-10 | Down |
| Avpr1a | -2.4641444 | 3.49E-11 | 3.21E-10 | Down |
| Gls | -1.0563679 | 3.75E-11 | 3.43E-10 | Down |
| Ppif | -1.0238359 | 4.17E-11 | 3.79E-10 | Down |
| Epb4.2 | -3.7137884 | 4.49E-11 | 4.07E-10 | Down |
| Ankrd13d | -1.0797397 | 4.61E-11 | 4.18E-10 | Down |
| Shisa2 | -1.5185585 | 4.99E-11 | 4.49E-10 | Down |
| 9130227L01Rik | -3.4957708 | 4.99E-11 | 4.49E-10 | Down |
| Ppm1e | -1.6897196 | 5.24E-11 | 4.70E-10 | Down |
| Adamtsl2 | -1.7527745 | 5.30E-11 | 4.75E-10 | Down |
| Wfs1 | -1.029279 | 5.37E-11 | 4.81E-10 | Down |
| Mterfd2 | -1.1503655 | 5.40E-11 | 4.83E-10 | Down |
| Syt12 | -1.3555172 | 5.41E-11 | 4.84E-10 | Down |
| Trpc3 | -1.5700019 | 5.47E-11 | 4.89E-10 | Down |
| Ubxn11 | -1.2208495 | 5.51E-11 | 4.91E-10 | Down |
| Lrrn2 | -1.6566389 | 5.55E-11 | 4.95E-10 | Down |
| Slc16a2 | -1.5998475 | 5.75E-11 | 5.11E-10 | Down |
| Pabpc4l | -1.8356316 | 6.22E-11 | 5.51E-10 | Down |
| 9230104L09Rik | -5.876326 | 7.21E-11 | 6.37E-10 | Down |
| Fam222a | -1.6017187 | 7.33E-11 | 6.46E-10 | Down |
| Ccdc121 | -2.4558307 | 7.83E-11 | 6.89E-10 | Down |
| Fbxo16 | -1.6651204 | 7.98E-11 | 7.01E-10 | Down |
| Ccdc141 | -1.300689 | 8.65E-11 | 7.55E-10 | Down |
| Icam5 | -2.1884912 | 8.83E-11 | 7.70E-10 | Down |
| Muc6 | -3.0445428 | 8.98E-11 | 7.82E-10 | Down |
| Fam211b | -1.1744955 | 9.02E-11 | 7.85E-10 | Down |
| 1810010H24Rik | -1.7051034 | 9.24E-11 | 8.03E-10 | Down |
| Snhg4 | -1.185901 | 9.44E-11 | 8.19E-10 | Down |
| Clcn6 | -1.1178639 | 9.71E-11 | 8.41E-10 | Down |
| Camk1d | -1.6546983 | 1.04E-10 | 9.01E-10 | Down |
| Hspa12a | -1.2410582 | 1.05E-10 | 9.06E-10 | Down |
| Phgdh | -1.0100179 | 1.14E-10 | 9.80E-10 | Down |
| Iyd | -1.1330794 | 1.14E-10 | 9.81E-10 | Down |
| Caskin1 | -1.4022364 | 1.18E-10 | 1.02E-09 | Down |
| 1700003D09Rik | -2.3693303 | 1.23E-10 | 1.06E-09 | Down |
| Pyroxd2 | -1.3046091 | 1.24E-10 | 1.06E-09 | Down |
| Gpam | -1.2719934 | 1.25E-10 | 1.07E-09 | Down |
| Pcp4 | -2.2663738 | 1.37E-10 | 1.17E-09 | Down |
| Cyp26a1 | -2.195363 | 1.39E-10 | 1.18E-09 | Down |
| Fn3k | -1.8283977 | 1.47E-10 | 1.25E-09 | Down |
| Cyp1a1 | -5.501103 | 1.54E-10 | 1.31E-09 | Down |
| Map1b | -1.2513919 | 1.75E-10 | 1.47E-09 | Down |
| Smtnl2 | -1.1726874 | 1.76E-10 | 1.48E-09 | Down |
| Dlx3 | -3.0007998 | 1.77E-10 | 1.49E-09 | Down |
| Gm10406 | -1.9074429 | 1.78E-10 | 1.50E-09 | Down |
| Nynrin | -1.074931 | 1.80E-10 | 1.51E-09 | Down |
| 2610203C20Rik | -1.6041395 | 1.87E-10 | 1.56E-09 | Down |
| Best3 | -2.2615382 | 1.89E-10 | 1.58E-09 | Down |
| Slc35f2 | -1.6863597 | 1.90E-10 | 1.58E-09 | Down |
| F5 | -1.8240674 | 1.93E-10 | 1.61E-09 | Down |
| Sdf2l1 | -1.3199812 | 1.95E-10 | 1.62E-09 | Down |
| Folr1 | -1.6875349 | 2.06E-10 | 1.71E-09 | Down |
| Gm16432 | -1.8105471 | 2.30E-10 | 1.89E-09 | Down |
| Crygn | -1.0495614 | 2.33E-10 | 1.92E-09 | Down |
| Tmem56 | -1.1219404 | 2.43E-10 | 1.99E-09 | Down |
| Gm10516 | -1.315209 | 2.45E-10 | 2.01E-09 | Down |
| Piga | -1.1609849 | 2.48E-10 | 2.03E-09 | Down |
| Pde7b | -1.3970662 | 2.51E-10 | 2.06E-09 | Down |
| Rec8 | -1.47827 | 2.64E-10 | 2.16E-09 | Down |
| Hpgds | -1.2749652 | 2.74E-10 | 2.23E-09 | Down |
| Cst8 | -2.7927792 | 2.78E-10 | 2.27E-09 | Down |
| Plag1 | -1.5683881 | 3.06E-10 | 2.48E-09 | Down |
| Tgif2 | -1.0748702 | 3.11E-10 | 2.52E-09 | Down |
| Edaradd | -1.8980393 | 3.36E-10 | 2.72E-09 | Down |
| Tmprss2 | -1.0162258 | 3.42E-10 | 2.77E-09 | Down |
| Iqcg | -1.5955188 | 3.47E-10 | 2.80E-09 | Down |
| Nrarp | -1.1275536 | 3.58E-10 | 2.88E-09 | Down |
| Mfsd6l | -1.3621969 | 3.71E-10 | 2.98E-09 | Down |
| Agphd1 | -1.0307224 | 3.72E-10 | 2.99E-09 | Down |
| 5033411D12Rik | -1.4732704 | 3.75E-10 | 3.01E-09 | Down |
| Slc22a19 | -2.3823581 | 3.86E-10 | 3.09E-09 | Down |
| Ikzf4 | -1.9407228 | 3.94E-10 | 3.15E-09 | Down |
| Ddc | -2.1829407 | 4.20E-10 | 3.35E-09 | Down |
| Id4 | -1.0679491 | 4.25E-10 | 3.39E-09 | Down |
| Zfp941 | -1.5835928 | 4.26E-10 | 3.39E-09 | Down |
| 9030404E10Rik | -1.7700624 | 4.66E-10 | 3.69E-09 | Down |
| Tenm3 | -1.0353706 | 4.68E-10 | 3.70E-09 | Down |
| Scin | -1.1230086 | 4.87E-10 | 3.85E-09 | Down |
| Asb2 | -2.2102318 | 4.89E-10 | 3.87E-09 | Down |
| Arhgap24 | -1.0266101 | 4.94E-10 | 3.90E-09 | Down |
| Map3k9 | -1.0877968 | 6.38E-10 | 4.98E-09 | Down |
| Grik4 | -1.3164006 | 6.84E-10 | 5.31E-09 | Down |
| Ccdc112 | -1.0316312 | 7.79E-10 | 6.01E-09 | Down |
| Ppm1l | -1.3820905 | 8.85E-10 | 6.76E-09 | Down |
| Kcnj2 | -1.6951381 | 8.93E-10 | 6.81E-09 | Down |
| Nr3c2 | -1.2981986 | 9.61E-10 | 7.29E-09 | Down |
| Wnt4 | -1.0359766 | 1.02E-09 | 7.69E-09 | Down |
| Syt2 | -2.4266665 | 1.05E-09 | 7.89E-09 | Down |
| S100a1 | -1.0891473 | 1.12E-09 | 8.41E-09 | Down |
| Prlr | -1.4619673 | 1.21E-09 | 9.04E-09 | Down |
| Pkd1l2 | -2.942966 | 1.23E-09 | 9.17E-09 | Down |
| Gm2897 | -2.447183 | 1.36E-09 | 1.01E-08 | Down |
| Rbbp8nl | -1.2052809 | 1.38E-09 | 1.02E-08 | Down |
| Rmst | -1.8110089 | 1.38E-09 | 1.02E-08 | Down |
| Fam163b | -2.859581 | 1.44E-09 | 1.06E-08 | Down |
| Zxdb | -1.0011671 | 1.62E-09 | 1.19E-08 | Down |
| Ube2ql1 | -2.9590215 | 1.72E-09 | 1.26E-08 | Down |
| Zdhhc23 | -1.954269 | 1.75E-09 | 1.28E-08 | Down |
| Scn4a | -1.9487233 | 1.75E-09 | 1.28E-08 | Down |
| Ccdc113 | -2.9425755 | 1.79E-09 | 1.31E-08 | Down |
| Nmu | -3.5323287 | 2.01E-09 | 1.46E-08 | Down |
| Fahd2a | -1.0240014 | 2.16E-09 | 1.57E-08 | Down |
| Glis1 | -2.1769224 | 2.30E-09 | 1.66E-08 | Down |
| Tmem171 | -1.4055417 | 2.31E-09 | 1.66E-08 | Down |
| Garem | -1.1107023 | 2.43E-09 | 1.75E-08 | Down |
| Reln | -1.3421247 | 2.65E-09 | 1.90E-08 | Down |
| Mdga1 | -1.1877797 | 3.01E-09 | 2.13E-08 | Down |
| Vwa2 | -2.2971532 | 3.12E-09 | 2.20E-08 | Down |
| Map3k13 | -1.297323 | 3.65E-09 | 2.55E-08 | Down |
| Plch1 | -1.4389199 | 3.95E-09 | 2.75E-08 | Down |
| Hsp90b1 | -1.0508484 | 4.19E-09 | 2.91E-08 | Down |
| Cdkl1 | -1.1563749 | 4.24E-09 | 2.94E-08 | Down |
| Plac8 | -1.4436383 | 4.41E-09 | 3.05E-08 | Down |
| Rab27b | -1.0686013 | 4.94E-09 | 3.40E-08 | Down |
| Glcci1 | -1.1459136 | 5.06E-09 | 3.48E-08 | Down |
| Ggct | -1.060836 | 5.37E-09 | 3.67E-08 | Down |
| 1190003J15Rik | -1.6951168 | 5.44E-09 | 3.71E-08 | Down |
| Glb1l2 | -1.8686973 | 5.63E-09 | 3.84E-08 | Down |
| Krt7 | -1.5751515 | 6.34E-09 | 4.30E-08 | Down |
| Vstm2b | -2.5613457 | 6.85E-09 | 4.63E-08 | Down |
| Slc26a7 | -1.7643981 | 7.35E-09 | 4.94E-08 | Down |
| 4931428F04Rik | -1.0414572 | 7.77E-09 | 5.20E-08 | Down |
| Sema6d | -1.0080564 | 8.02E-09 | 5.37E-08 | Down |
| Vdr | -1.2523014 | 8.08E-09 | 5.40E-08 | Down |
| Phf21b | -1.5998781 | 8.54E-09 | 5.69E-08 | Down |
| Dclk3 | -1.2275199 | 9.65E-09 | 6.38E-08 | Down |
| Fam83g | -1.085074 | 9.65E-09 | 6.38E-08 | Down |
| Cybrd1 | -1.5071668 | 9.93E-09 | 6.55E-08 | Down |
| Kcnq1 | -1.4997974 | 1.02E-08 | 6.71E-08 | Down |
| Iqsec3 | -1.8656009 | 1.09E-08 | 7.17E-08 | Down |
| Slc20a1 | -1.1200908 | 1.11E-08 | 7.28E-08 | Down |
| Cnksr1 | -1.0316176 | 1.13E-08 | 7.41E-08 | Down |
| Tenm4 | -1.8064347 | 1.14E-08 | 7.46E-08 | Down |
| Sult5a1 | -3.62359 | 1.17E-08 | 7.66E-08 | Down |
| Acy1 | -1.1501459 | 1.22E-08 | 7.98E-08 | Down |
| Sh3bgr | -1.6010311 | 1.24E-08 | 8.08E-08 | Down |
| Cpne7 | -2.2651122 | 1.26E-08 | 8.20E-08 | Down |
| St3gal6 | -1.0224202 | 1.36E-08 | 8.80E-08 | Down |
| 6030466F02Rik | -2.1796179 | 1.36E-08 | 8.84E-08 | Down |
| A330048O09Rik | -1.9351645 | 1.37E-08 | 8.88E-08 | Down |
| Tacr3 | -3.7951583 | 1.39E-08 | 8.98E-08 | Down |
| Grip1 | -2.9181229 | 1.46E-08 | 9.39E-08 | Down |
| S100a5 | -1.7515971 | 1.47E-08 | 9.46E-08 | Down |
| Nos2 | -1.9458688 | 1.50E-08 | 9.64E-08 | Down |
| Tfap2c | -1.2154049 | 1.54E-08 | 9.91E-08 | Down |
| Hpdl | -1.6537464 | 1.67E-08 | 1.07E-07 | Down |
| Vamp1 | -1.020156 | 1.72E-08 | 1.10E-07 | Down |
| Tmem44 | -2.5229552 | 1.78E-08 | 1.14E-07 | Down |
| Ccdc64 | -1.083581 | 1.81E-08 | 1.15E-07 | Down |
| Gm16065 | -1.6242596 | 1.85E-08 | 1.18E-07 | Down |
| Spata2l | -1.1104226 | 1.96E-08 | 1.24E-07 | Down |
| Ltbp1 | -1.2004959 | 2.12E-08 | 1.34E-07 | Down |
| Pdk1 | -1.0042021 | 2.21E-08 | 1.39E-07 | Down |
| Fgf4 | -2.6958041 | 2.24E-08 | 1.41E-07 | Down |
| 1700011M02Rik | -1.1613664 | 2.30E-08 | 1.45E-07 | Down |
| Mzb1 | -2.0824311 | 2.41E-08 | 1.51E-07 | Down |
| Fbxo2 | -1.3251797 | 2.47E-08 | 1.54E-07 | Down |
| Kiss1r | -1.0698104 | 2.49E-08 | 1.56E-07 | Down |
| Gm8579 | -2.0266799 | 2.62E-08 | 1.64E-07 | Down |
| Rnf32 | -1.329501 | 2.67E-08 | 1.66E-07 | Down |
| 0610009B14Rik | -1.5296062 | 2.75E-08 | 1.71E-07 | Down |
| Camk1g | -1.4568293 | 2.92E-08 | 1.81E-07 | Down |
| Ank3 | -1.3021922 | 3.04E-08 | 1.88E-07 | Down |
| Ocln | -1.2243237 | 3.14E-08 | 1.94E-07 | Down |
| Gm9112 | -2.2359311 | 3.30E-08 | 2.04E-07 | Down |
| Dpysl3 | -1.4243306 | 3.37E-08 | 2.07E-07 | Down |
| Khk | -1.1873405 | 3.85E-08 | 2.34E-07 | Down |
| Slc8a1 | -1.2345524 | 4.29E-08 | 2.60E-07 | Down |
| Ankrd24 | -1.21602 | 4.67E-08 | 2.81E-07 | Down |
| Ttc22 | -2.0631645 | 4.76E-08 | 2.86E-07 | Down |
| Gstm7 | -1.2002305 | 5.38E-08 | 3.20E-07 | Down |
| Fbxl21 | -1.4495553 | 5.60E-08 | 3.33E-07 | Down |
| Egln3 | -1.0496653 | 5.67E-08 | 3.36E-07 | Down |
| Agr2 | -1.2933399 | 6.11E-08 | 3.62E-07 | Down |
| Slc2a3 | -1.2667999 | 6.26E-08 | 3.70E-07 | Down |
| Ttll10 | -1.2656693 | 6.49E-08 | 3.82E-07 | Down |
| 1600016N20Rik | -1.1483106 | 6.83E-08 | 4.01E-07 | Down |
| 4632428C04Rik | -1.1085299 | 7.27E-08 | 4.25E-07 | Down |
| Pla2g4b | -1.0640277 | 7.38E-08 | 4.31E-07 | Down |
| Fam189a1 | -1.6304415 | 8.73E-08 | 5.06E-07 | Down |
| B3galt5 | -1.4293111 | 9.00E-08 | 5.21E-07 | Down |
| Dusp8 | -1.5744189 | 9.81E-08 | 5.64E-07 | Down |
| Med12l | -1.145365 | 9.91E-08 | 5.69E-07 | Down |
| Dpp4 | -1.0304793 | 1.05E-07 | 6.01E-07 | Down |
| Adamts13 | -4.4165468 | 1.09E-07 | 6.19E-07 | Down |
| Dnahc17 | -3.1090284 | 1.10E-07 | 6.26E-07 | Down |
| Gfra1 | -1.7114095 | 1.10E-07 | 6.28E-07 | Down |
| Dzank1 | -1.0296161 | 1.14E-07 | 6.51E-07 | Down |
| Klhl32 | -1.6534224 | 1.16E-07 | 6.59E-07 | Down |
| B3galt1 | -2.5924034 | 1.20E-07 | 6.77E-07 | Down |
| Creb3l2 | -1.0670702 | 1.23E-07 | 6.93E-07 | Down |
| Pcdh17 | -1.3844488 | 1.25E-07 | 7.04E-07 | Down |
| F3 | -1.0189862 | 1.35E-07 | 7.56E-07 | Down |
| Corin | -1.5937452 | 1.35E-07 | 7.56E-07 | Down |
| 4921507P07Rik | -1.4796952 | 1.41E-07 | 7.89E-07 | Down |
| Morn4 | -1.0456215 | 1.43E-07 | 7.96E-07 | Down |
| Slc29a2 | -1.328532 | 1.67E-07 | 9.23E-07 | Down |
| Arntl | -1.0103845 | 1.73E-07 | 9.57E-07 | Down |
| Emid1 | -1.3778152 | 1.87E-07 | 1.03E-06 | Down |
| Cd59b | -1.2346192 | 1.94E-07 | 1.07E-06 | Down |
| Lipg | -2.3928916 | 2.00E-07 | 1.10E-06 | Down |
| Ces1g | -2.0918627 | 2.00E-07 | 1.10E-06 | Down |
| Pcnxl2 | -1.7333515 | 2.18E-07 | 1.19E-06 | Down |
| Faim2 | -2.5614732 | 2.39E-07 | 1.30E-06 | Down |
| 1700040L02Rik | -2.0300351 | 2.41E-07 | 1.31E-06 | Down |
| Garnl3 | -1.0014185 | 2.65E-07 | 1.43E-06 | Down |
| Lrrtm1 | -3.8500194 | 2.67E-07 | 1.44E-06 | Down |
| Syn3 | -1.0404365 | 2.73E-07 | 1.47E-06 | Down |
| Abcb9 | -1.0670573 | 2.78E-07 | 1.49E-06 | Down |
| Bmp2 | -1.6157811 | 2.81E-07 | 1.51E-06 | Down |
| Clca5 | -2.6242668 | 3.26E-07 | 1.73E-06 | Down |
| Nos1ap | -1.6900579 | 3.31E-07 | 1.76E-06 | Down |
| Efcab12 | -1.7078199 | 3.41E-07 | 1.81E-06 | Down |
| Fxyd4 | -1.2155199 | 3.44E-07 | 1.82E-06 | Down |
| Dmrtc1a | -1.0129641 | 3.56E-07 | 1.88E-06 | Down |
| Plcb4 | -1.0483663 | 3.99E-07 | 2.09E-06 | Down |
| Clcnkb | -2.1113446 | 4.40E-07 | 2.29E-06 | Down |
| Zfp184 | -1.0731001 | 4.43E-07 | 2.30E-06 | Down |
| Rbm11 | -1.5849934 | 4.49E-07 | 2.33E-06 | Down |
| BC021767 | -1.3085034 | 4.53E-07 | 2.35E-06 | Down |
| Peg3 | -1.5019022 | 4.80E-07 | 2.47E-06 | Down |
| Eddm3b | -1.3619911 | 4.90E-07 | 2.52E-06 | Down |
| Mia | -1.3422313 | 5.13E-07 | 2.63E-06 | Down |
| Tex11 | -1.3249884 | 5.37E-07 | 2.75E-06 | Down |
| Ramp3 | -1.1777567 | 5.44E-07 | 2.78E-06 | Down |
| Tppp | -1.1150036 | 5.72E-07 | 2.91E-06 | Down |
| Fam83b | -1.3913588 | 6.19E-07 | 3.13E-06 | Down |
| 4931403E22Rik | -1.9817846 | 6.38E-07 | 3.22E-06 | Down |
| Ceacam19 | -1.7938525 | 6.63E-07 | 3.33E-06 | Down |
| Ralgps1 | -1.1217101 | 6.82E-07 | 3.42E-06 | Down |
| Dpf3 | -1.1382789 | 6.85E-07 | 3.44E-06 | Down |
| Ggnbp1 | -1.5687601 | 7.19E-07 | 3.60E-06 | Down |
| Creld2 | -1.0421423 | 7.23E-07 | 3.62E-06 | Down |
| Bche | -1.5803011 | 7.65E-07 | 3.81E-06 | Down |
| Tnc | -1.341141 | 8.27E-07 | 4.11E-06 | Down |
| Lrrc29 | -1.2325832 | 8.51E-07 | 4.21E-06 | Down |
| Dnase1 | -2.7332737 | 8.61E-07 | 4.25E-06 | Down |
| Rab17 | -1.2449036 | 8.84E-07 | 4.36E-06 | Down |
| Rnf180 | -1.4260997 | 9.28E-07 | 4.57E-06 | Down |
| Ttc18 | -1.7190113 | 9.28E-07 | 4.57E-06 | Down |
| Lrrc6 | -1.9848549 | 9.30E-07 | 4.57E-06 | Down |
| Apold1 | -1.0742787 | 1.01E-06 | 4.93E-06 | Down |
| 4930451G09Rik | -1.0353806 | 1.02E-06 | 4.96E-06 | Down |
| Cldn15 | -1.4602411 | 1.05E-06 | 5.11E-06 | Down |
| A330023F24Rik | -1.0480215 | 1.17E-06 | 5.66E-06 | Down |
| Tbx22 | -1.0153821 | 1.23E-06 | 5.90E-06 | Down |
| Rnft2 | -1.2179763 | 1.28E-06 | 6.14E-06 | Down |
| Pemt | -1.2304017 | 1.31E-06 | 6.28E-06 | Down |
| Ablim2 | -1.2356489 | 1.41E-06 | 6.73E-06 | Down |
| Ankrd45 | -2.4386765 | 1.42E-06 | 6.77E-06 | Down |
| Igsf1 | -2.4838101 | 1.44E-06 | 6.87E-06 | Down |
| Rab40b | -1.0566875 | 1.50E-06 | 7.11E-06 | Down |
| Tmem82 | -1.2190131 | 1.51E-06 | 7.18E-06 | Down |
| Timp3 | -1.1691255 | 1.61E-06 | 7.61E-06 | Down |
| Zfp72 | -1.1473313 | 1.68E-06 | 7.89E-06 | Down |
| Ccne1 | -1.006338 | 1.70E-06 | 7.98E-06 | Down |
| Apln | -1.6767018 | 1.75E-06 | 8.20E-06 | Down |
| Dao | -1.1588082 | 1.76E-06 | 8.26E-06 | Down |
| Pde4c | -1.607804 | 1.83E-06 | 8.55E-06 | Down |
| Gm5577 | -1.7584217 | 1.88E-06 | 8.79E-06 | Down |
| Sez6l2 | -1.1969965 | 2.20E-06 | 1.02E-05 | Down |
| Ahsg | -2.7401643 | 2.33E-06 | 1.07E-05 | Down |
| 1700001C02Rik | -2.8029686 | 2.33E-06 | 1.07E-05 | Down |
| Cers6 | -1.1762585 | 2.44E-06 | 1.12E-05 | Down |
| D630029K05Rik | -2.1283653 | 2.61E-06 | 1.19E-05 | Down |
| Atp8a2 | -1.9468718 | 2.75E-06 | 1.25E-05 | Down |
| Wif1 | -2.6559397 | 2.87E-06 | 1.30E-05 | Down |
| Eno4 | -1.5263398 | 2.93E-06 | 1.33E-05 | Down |
| Rsph4a | -2.0025566 | 3.37E-06 | 1.51E-05 | Down |
| Slc26a2 | -1.0998625 | 3.47E-06 | 1.56E-05 | Down |
| Wnt2 | -1.9994634 | 3.50E-06 | 1.57E-05 | Down |
| Kcnj4 | -3.7093096 | 3.57E-06 | 1.60E-05 | Down |
| Gm5113 | -1.0545922 | 3.60E-06 | 1.61E-05 | Down |
| C2cd4b | -2.4464147 | 4.07E-06 | 1.80E-05 | Down |
| Gm16576 | -1.0562057 | 4.13E-06 | 1.83E-05 | Down |
| Efcc1 | -1.7324654 | 4.50E-06 | 1.98E-05 | Down |
| Spef2 | -1.59717 | 4.54E-06 | 2.00E-05 | Down |
| Pak6 | -1.2982094 | 4.75E-06 | 2.08E-05 | Down |
| Ttll11 | -1.0988349 | 5.01E-06 | 2.18E-05 | Down |
| B230206H07Rik | -1.819632 | 5.45E-06 | 2.36E-05 | Down |
| Frmpd4 | -1.4093692 | 5.89E-06 | 2.54E-05 | Down |
| Syt17 | -1.81572 | 5.93E-06 | 2.55E-05 | Down |
| Snca | -2.8378377 | 6.28E-06 | 2.69E-05 | Down |
| Angpt2 | -1.3555913 | 6.68E-06 | 2.84E-05 | Down |
| Gjb4 | -1.1765506 | 7.09E-06 | 3.01E-05 | Down |
| Unc5d | -5.1965967 | 7.82E-06 | 3.30E-05 | Down |
| Eif4ebp3 | -1.0784566 | 9.74E-06 | 4.05E-05 | Down |
| Spdef | -1.6148756 | 1.01E-05 | 4.19E-05 | Down |
| Ell3 | -1.1179552 | 1.05E-05 | 4.34E-05 | Down |
| Vstm5 | -1.8146542 | 1.09E-05 | 4.49E-05 | Down |
| Tnfrsf11b | -1.3869967 | 1.15E-05 | 4.72E-05 | Down |
| Pcdhgb8 | -1.3318839 | 1.20E-05 | 4.90E-05 | Down |
| Vit | -1.3068003 | 1.23E-05 | 5.01E-05 | Down |
| Rasgrp2 | -1.0112359 | 1.29E-05 | 5.25E-05 | Down |
| Cldn9 | -1.1306878 | 1.34E-05 | 5.41E-05 | Down |
| Ano9 | -1.0040959 | 1.43E-05 | 5.75E-05 | Down |
| Tiam2 | -1.0004525 | 1.58E-05 | 6.29E-05 | Down |
| Gm973 | -2.0839821 | 1.59E-05 | 6.34E-05 | Down |
| 2310014L17Rik | -1.4679158 | 1.65E-05 | 6.55E-05 | Down |
| Shisa7 | -2.7778866 | 1.70E-05 | 6.73E-05 | Down |
| Veph1 | -1.541817 | 1.71E-05 | 6.75E-05 | Down |
| Vsig4 | -1.564344 | 1.71E-05 | 6.77E-05 | Down |
| Lnx1 | -1.1077845 | 1.71E-05 | 6.77E-05 | Down |
| Gng4 | -2.7110366 | 1.87E-05 | 7.34E-05 | Down |
| Zfp457 | -1.5875674 | 1.93E-05 | 7.55E-05 | Down |
| Calr4 | -1.908917 | 2.00E-05 | 7.77E-05 | Down |
| Acat3 | -1.8741683 | 2.11E-05 | 8.17E-05 | Down |
| Gm5126 | -1.7098502 | 2.19E-05 | 8.47E-05 | Down |
| Kitl | -1.144125 | 2.30E-05 | 8.84E-05 | Down |
| Mpped1 | -2.333777 | 2.33E-05 | 8.97E-05 | Down |
| A930013F10Rik | -1.4430419 | 2.41E-05 | 9.23E-05 | Down |
| Tmem255a | -1.0475291 | 2.49E-05 | 9.53E-05 | Down |
| Cldn8 | -1.1121644 | 2.61E-05 | 9.96E-05 | Down |
| Spag6 | -1.878797 | 2.66E-05 | 0.0001013 | Down |
| Prtg | -1.8004302 | 2.86E-05 | 0.0001079 | Down |
| Gpr135 | -1.5364599 | 2.99E-05 | 0.0001126 | Down |
| Nphp4 | -1.0952509 | 3.14E-05 | 0.0001179 | Down |
| Hey2 | -1.0220881 | 3.33E-05 | 0.0001244 | Down |
| Katnal2 | -1.3263087 | 3.34E-05 | 0.0001248 | Down |
| Panx2 | -1.7705213 | 3.34E-05 | 0.0001248 | Down |
| Trpc1 | -1.0202604 | 3.45E-05 | 0.0001286 | Down |
| Slc1a1 | -1.0294047 | 3.53E-05 | 0.0001312 | Down |
| Prss53 | -1.3805954 | 3.55E-05 | 0.0001317 | Down |
| Flrt1 | -1.0015837 | 3.68E-05 | 0.0001365 | Down |
| Muc1 | -1.4770623 | 3.74E-05 | 0.0001384 | Down |
| Has1 | -1.9785128 | 4.30E-05 | 0.0001573 | Down |
| Robo3 | -1.8300082 | 4.33E-05 | 0.0001583 | Down |
| Dpyd | -1.3830382 | 4.49E-05 | 0.0001634 | Down |
| Gm14207 | -1.4368764 | 4.59E-05 | 0.0001669 | Down |
| Myh11 | -1.0858736 | 4.69E-05 | 0.0001703 | Down |
| Gm13807 | -1.1703323 | 4.98E-05 | 0.0001802 | Down |
| D830030K20Rik | -3.932926 | 5.02E-05 | 0.0001817 | Down |
| Zfp711 | -1.5037608 | 5.07E-05 | 0.0001834 | Down |
| Chrm3 | -1.6673472 | 5.10E-05 | 0.0001843 | Down |
| Pcdhga11 | -1.0065448 | 5.24E-05 | 0.000189 | Down |
| F420014N23Rik | -1.1263151 | 5.29E-05 | 0.0001904 | Down |
| Zfp9 | -1.0023891 | 5.61E-05 | 0.0002008 | Down |
| 4933406C10Rik | -1.401524 | 5.62E-05 | 0.0002012 | Down |
| Gli1 | -1.6168072 | 5.65E-05 | 0.0002022 | Down |
| Tsnaxip1 | -1.6971536 | 5.82E-05 | 0.0002076 | Down |
| Stab2 | -1.9354369 | 6.01E-05 | 0.0002136 | Down |
| Nuggc | -1.4424694 | 6.61E-05 | 0.0002329 | Down |
| Cpne5 | -1.3113492 | 7.06E-05 | 0.0002477 | Down |
| Slc9a7 | -1.0247917 | 7.32E-05 | 0.0002561 | Down |
| Slc26a10 | -1.4687367 | 7.41E-05 | 0.0002591 | Down |
| Ccdc67 | -1.32574 | 7.71E-05 | 0.0002687 | Down |
| Astn2 | -1.2076574 | 8.42E-05 | 0.0002909 | Down |
| Fcgbp | -1.6418135 | 8.77E-05 | 0.0003019 | Down |
| Cntn2 | -1.8964449 | 8.82E-05 | 0.0003036 | Down |
| Cd163l1 | -1.5445137 | 0.0001018 | 0.0003461 | Down |
| Bcl6b | -1.095821 | 0.0001073 | 0.0003632 | Down |
| Zbtb32 | -1.0432533 | 0.0001078 | 0.0003648 | Down |
| Acot3 | -2.0567278 | 0.0001187 | 0.0003989 | Down |
| Snx29 | -1.0403473 | 0.0001205 | 0.0004045 | Down |
| St8sia1 | -2.0451561 | 0.0001229 | 0.0004117 | Down |
| A730090H04Rik | -2.405648 | 0.0001243 | 0.0004159 | Down |
| Hba-a2 | -3.0409293 | 0.0001287 | 0.0004288 | Down |
| BC065397 | -1.1498117 | 0.0001308 | 0.000435 | Down |
| D030025P21Rik | -1.543867 | 0.0001346 | 0.0004464 | Down |
| 1700003E16Rik | -1.0514609 | 0.000135 | 0.0004478 | Down |
| Rundc3b | -1.2504814 | 0.0001367 | 0.0004529 | Down |
| Murc | -1.3559362 | 0.0001373 | 0.0004544 | Down |
| Samd12 | -1.6517915 | 0.0001477 | 0.0004861 | Down |
| Grik2 | -1.9261558 | 0.0001518 | 0.0004988 | Down |
| Nme5 | -1.1862107 | 0.0001654 | 0.0005391 | Down |
| Otogl | -1.435473 | 0.0001692 | 0.0005505 | Down |
| Cml2 | -1.51161 | 0.0001801 | 0.0005825 | Down |
| Dbndd1 | -1.0122444 | 0.0002035 | 0.0006505 | Down |
| Slc7a5 | -1.0936461 | 0.0002072 | 0.0006608 | Down |
| Atp10b | -1.0873936 | 0.0002074 | 0.0006612 | Down |
| 9530026P05Rik | -1.2612306 | 0.000222 | 0.0007025 | Down |
| Ccdc158 | -1.2924028 | 0.0002233 | 0.0007061 | Down |
| 2610027K06Rik | -1.0268826 | 0.0002297 | 0.0007246 | Down |
| Map3k15 | -1.2264141 | 0.0002556 | 0.0007985 | Down |
| 2810408I11Rik | -1.1457338 | 0.0002596 | 0.0008099 | Down |
| Cdkl5 | -1.3582667 | 0.0002831 | 0.0008778 | Down |
| BC030307 | -1.0042397 | 0.0002862 | 0.0008865 | Down |
| Eif2s3y | -6.7591636 | 0.0002938 | 0.0009083 | Down |
| Smoc2 | -1.0215509 | 0.0003006 | 0.0009274 | Down |
| Dusp10 | -1.0370067 | 0.0003135 | 0.0009637 | Down |
| Cacng7 | -1.2255103 | 0.0003176 | 0.0009749 | Down |
| Ryr3 | -1.304953 | 0.0003186 | 0.0009776 | Down |
| Epha5 | -1.4557986 | 0.0003225 | 0.000988 | Down |
| Tspan33 | -1.3512003 | 0.0003254 | 0.0009959 | Down |
| Scgb1a1 | -1.9743527 | 0.0003837 | 0.0011555 | Down |
| Tsix | -1.7125506 | 0.0004093 | 0.0012226 | Down |
| Ly6k | -1.0931079 | 0.000454 | 0.0013445 | Down |
| Casr | -2.0100682 | 0.0004574 | 0.001354 | Down |
| Necab1 | -1.2825742 | 0.000529 | 0.0015508 | Down |
| Dnahc9 | -2.6815088 | 0.0005453 | 0.0015944 | Down |
| 9130206I24Rik | -1.0110198 | 0.0005823 | 0.0016928 | Down |
| Ccr6 | -1.3834009 | 0.0006201 | 0.0017924 | Down |
| 1700071M16Rik | -1.5408395 | 0.0006278 | 0.0018132 | Down |
| AW549542 | -1.3367195 | 0.0007588 | 0.0021521 | Down |
| Uroc1 | -1.4698597 | 0.0007815 | 0.0022104 | Down |
| Kcnh1 | -1.1957835 | 0.0008044 | 0.0022703 | Down |
| Cacna2d2 | -1.4548681 | 0.0008889 | 0.0024804 | Down |
| Asb11 | -1.802623 | 0.0008973 | 0.0025005 | Down |
| Pcdhga8 | -1.2728821 | 0.0009051 | 0.0025199 | Down |
| Aadat | -1.3781278 | 0.0009499 | 0.0026322 | Down |
| Nlgn3 | -1.3597774 | 0.0010141 | 0.0027911 | Down |
| Pcdhgb4 | -1.0750642 | 0.0010233 | 0.0028147 | Down |
| Ddx3y | -6.1272521 | 0.0011219 | 0.0030582 | Down |
| Mpz | -1.1136792 | 0.0011628 | 0.0031613 | Down |
| Cabyr | -1.5706187 | 0.0011887 | 0.0032251 | Down |
| 9130230L23Rik | -1.1049846 | 0.0012138 | 0.0032864 | Down |
| Kcna2 | -1.1655112 | 0.0013288 | 0.0035594 | Down |
| Lefty2 | -1.1606319 | 0.0013303 | 0.0035626 | Down |
| Ppifos | -1.1730672 | 0.001331 | 0.0035639 | Down |
| Npm2 | -1.4167201 | 0.0014071 | 0.0037454 | Down |
| Ptgds | -1.0877912 | 0.0014216 | 0.0037765 | Down |
| 4930452B06Rik | -1.6258589 | 0.0014662 | 0.0038787 | Down |
| Grtp1 | -1.1153719 | 0.0015137 | 0.0039934 | Down |
| Sntg1 | -1.5512288 | 0.001552 | 0.004081 | Down |
| E330009J07Rik | -1.0084589 | 0.0016138 | 0.0042229 | Down |
| Car7 | -1.0292499 | 0.0016225 | 0.0042419 | Down |
| Tnfsf11 | -1.790888 | 0.0016734 | 0.0043616 | Down |
| 1700031F05Rik | -1.921194 | 0.0017812 | 0.0046111 | Down |
| Adamts17 | -1.1481194 | 0.0019387 | 0.0049743 | Down |
| Steap1 | -1.0946573 | 0.0022718 | 0.0057157 | Down |
| Chga | -3.8686756 | 0.0022843 | 0.0057443 | Down |
| Atp1a4 | -1.0954233 | 0.0023349 | 0.0058513 | Down |
| Gm10635 | -1.0975923 | 0.0024495 | 0.0061066 | Down |
| Gm11992 | -1.1346278 | 0.0026178 | 0.0064818 | Down |
| Ar | -1.0487244 | 0.0026488 | 0.0065496 | Down |
| Ostn | -2.2690199 | 0.0026908 | 0.0066476 | Down |
| Spata21 | -2.1976209 | 0.0027086 | 0.0066859 | Down |
| Satb1 | -1.0652194 | 0.0028743 | 0.0070506 | Down |
| Fam47e | -1.5021072 | 0.0031617 | 0.007679 | Down |
| Awat2 | -1.2378813 | 0.0035225 | 0.0084534 | Down |
[truncated: 188,456 more chars]
